# Cognitive function in different motor subtypes of Parkinson’s disease: A systematic review and multilevel meta-analysis

**DOI:** 10.3758/s13415-025-01343-8

**Published:** 2025-12-17

**Authors:** Brittany Child, Nathan Beu, Isaac Saywell, Robyn da Silva, Lyndsey Collins-Praino, Irina Baetu

**Affiliations:** 1 School of Psychology, University of Adelaide, Hughes Building, North Terrace, Adelaide, South Australia 5005 Australia; 2 College of Education, Psychology, and Social Work, Flinders University, Adelaide, Australia; 3 School of Biomedicine, University of Adelaide, Adelaide, Australia

**Keywords:** Parkinson’s disease, Motor disorders, Classification, Motor function, Cognition, CRD42022362290

## Abstract

Although defined as a movement disorder, Parkinson’s disease is characterised by a diverse range of motor and nonmotor symptoms, including cognitive impairment. Use of motor subtypes to characterise symptom heterogeneity is common, but it remains unclear whether these subtypes capture meaningful differences in nonmotor symptoms. Our systematic review and meta-analysis sought to investigate differences in cognitive function between motor subtypes of Parkinson’s disease. A total of 123 eligible studies were identified from database searches (PubMed, PsycINFO, CINAHL, Scopus, Web of Science; last searched 23/01/2024) and forward and backward snowballing. All included studies used at least one objective or clinician-rated motor measure to classify patients into motor subtypes and administered one or more objective or clinician-rated cognitive assessments. Data were analysed using a combination of multilevel meta-analysis, traditional meta-analysis, and narrative synthesis. Several study characteristics, including subtyping method, cognitive domain, and disease duration, were evaluated as possible moderators, and risk of bias was assessed using an adapted version of the Quality in Prognosis Studies tool. We found robust evidence for better preserved cognition in tremor-dominant motor subtypes and for poorer cognition in freezing of gait, postural instability gait disorder, and akinetic-rigid subtypes. Our moderator analyses indicated that motor subtype provides important information about cognitive profile that cannot be inferred from other patient characteristics. Unfortunately, reporting of motor subtyping procedures often lacked clarity, compromising subtype reproducibility. Our findings demonstrate the utility of motor subtyping for identifying patients most at risk of cognitive decline and dementia, and highlight opportunities for future motor subtyping research.

**Supplementary Information:**

The online version contains supplementary material available at 10.3758/s13415-025-01343-8.

## Introduction

### Rationale

Parkinson’s disease (PD) is the fastest growing neurological disorder globally (Feigin et al., 2017), outpacing Alzheimer’s disease, with its prevalence projected to double between 2014 and 2040 (Dorsey & Bloem, 2018). Predominantly driven by the death of dopaminergic neurons in the substantia nigra pars compacta, PD is defined as a movement disorder characterised by four cardinal motor symptoms: resting tremor, bradykinesia (slowness of movement), muscle rigidity, and impaired gait (Erro & Stamelou, 2017; Kalia & Lang, 2015). People with PD also experience a wide range of nonmotor symptoms that affect processes such as olfaction, digestion, mood, and cognition (Zis et al., 2015). These nonmotor symptoms have repeatedly been found to be stronger predictors of patients’ quality of life than motor symptoms (Martinez‐Martin et al., 2011b; Müller et al., 2013; Prakash et al., 2016), highlighting the importance of understanding both the motor and nonmotor aspects of PD. Critically, however, bradykinesia (accompanied by at least one other cardinal motor symptom) is the only motor symptom required for diagnosis, and diagnosis does not require the presence of any nonmotor symptoms (Gibb & Lees, 1988; Postuma et al., 2015). As a consequence, PD patients vary considerably in symptom profile, as well as in symptom severity and rate of decline. This substantial heterogeneity presents challenges for providing accurate prognoses regarding disease progression and for offering treatment tailored to each patient’s unique symptom profile (Greenland et al., 2019).

Over the past decade, research efforts have been increasingly directed towards the use of subtyping methods to characterise symptom heterogeneity among PD patients. Broadly, these approaches seek to identify demographic and disease features that tend to co-occur, giving rise to distinct clusters (subtypes) of patients with similar symptom profiles (Marras & Lang, 2013). It is theorised that any disease features that cluster together may be reflective of shared underlying pathophysiology and that patients belonging to the same subtype will have similar disease trajectories and treatment responses distinct from those of patients belonging to other subtype groups (Marras, 2015; Marras & Lang, 2013). Having a taxonomy of subtypes into which patients can be classified has considerable potential to improve prognostic accuracy, reducing uncertainty for patients and their loved ones. In addition, it also provides opportunities for early intervention and prevention strategies tailored to an individual’s projected disease course (van Rooden et al., 2010).

Overwhelmingly, subtyping research in PD has focused on identifying *motor* subtypes, wherein subtype groups are defined on the basis of motor symptom profile alone. Of these motor subtyping frameworks, the most frequently used classifies patients as belonging to tremor-dominant (TD), postural instability gait disorder (PIGD), and indeterminate (ID) subtypes (Jankovic et al., 1990; Stebbins et al., 2013). In another, similar motor subtyping framework, patients are classified into TD, akinetic-rigid (AR), and mixed (MX) subtype groups (Kang et al., 2005; Rajput et al., 2009). Other motor subtyping frameworks are less elaborate, classifying patients based on the presence or absence of a single motor symptom (e.g., freezing of gait [FOG] vs. no freezing of gait (nFOG; Ortelli et al., 2019); tremor [TR-Y] vs. no-tremor (TR-N; Poletti et al., 2012)).

Using motor subtyping frameworks such as these, researchers have sought to compare motor subtype groups on nonmotor symptoms to establish whether motor subtype classification can be used to predict the type and severity of a patient’s nonmotor symptoms. One of the most common yet underrecognised nonmotor symptoms in PD is cognitive impairment (for review, see Aarsland et al., 2021). Approximately half of PD patients experience some degree of cognitive impairment within five to six years from diagnosis (Pigott et al., 2015; Williams-Gray et al., 2007). This impairment may begin as subjective cognitive impairment and progress to formally diagnosed mild cognitive impairment (PD-MCI) or PD dementia (PDD; Aarsland et al., 2017; Kehagia et al., 2010). Cognitive impairment and dementia are associated with reduced quality of life for both patients (Lawson et al., 2014) and their carers (Lawson et al., 2017; Morley et al., 2012), as well as increased healthcare service utilisation (Chandler et al., 2021; Vossius et al., 2011) and increased mortality (Backstrom et al., 2018; Willis et al., 2012).

Understanding the relationship between motor subtype and cognitive impairment in PD has potential clinical utility for identifying patients most at risk of PD-MCI and PDD, providing opportunities for more effective prevention and early intervention strategies tailored to those most at risk. However, there is no consensus on the strength of the relationship between motor and cognitive function, limiting confidence in such risk predictions. When comparing cognitive function across motor subtype groups, findings tend to support a motor-cognitive relationship. Several studies have, for example, documented a higher incidence of PD-MCI (Poletti et al., 2012) and PDD (Alves et al., 2006) in nontremor subtypes (PIGD, AR) compared with the TD subtype. Other studies, however, have failed to replicate these results, instead reporting no differences in cognition between motor subtype groups (Ren et al., 2020; Urso et al., 2022) or results trending towards higher rates of PD-MCI and dementia among TD patients (Paulus & Jellinger, 1991; Stojkovic et al., 2018).

Methodological heterogeneity between motor subtyping studies may, at least in part, account for these inconsistent results. The proliferation of different subtyping frameworks (e.g., TD/PIGD/ID, TD/AR/MX, FOG/nFOG) presents challenges for synthesising study results, and additional complexity arises from the use of *different* subtyping procedures to classify patients under the *same* subtyping framework. For example, under the TD/PIGD/ID framework, patients are usually classified into subtype groups based on their ratio of tremor to PIGD symptoms. The original classification method, developed by Jankovic and colleagues (1990), uses tremor and PIGD scores calculated from the Unified Parkinson’s Disease Rating Scale (UPDRS; Fahn et al., 1987). In response to revisions to the UPDRS, resulting in the Movement Disorder Society UPDRS (MDS-UPDRS; Goetz et al., 2008), Stebbins and colleagues (2013) published an updated version of Jankovic et al.’s (1990) method using scores calculated from this revised scale. Although developed with the intent of replicating Jankovic et al.’s (1990) classification, Stebbins et al. (2013) reported positive predictive values of 96% and 97% for the TD and PIGD groups, respectively, suggesting an excellent, but imperfect, equivalence. Other subtyping methods seem less consistent. In another study (von Coelln et al., 2015), Jankovic et al.’s (1990) subtyping method for classifying participants as TD, PIGD, or ID was compared with two different methods for classifying participants as TD, AR, or MX (Eggers et al., 2011; Schiess et al., 2000). Marked inconsistencies between all three methods were noted in the percentage of patients classified as TD (7–57%), PIGD/AR (33–70%), and ID/MX (9–37%). Moreover, most subtyping methods rely upon clinician assessment (e.g., rating of UPDRS-III items, clinical observation of FOG); as a result, differences in clinicians’ judgements may lead to differences in a patient’s subtype classification, even when the same procedure is followed.

Among studies specifically investigating cognitive differences between motor subtypes, methodological heterogeneity is also present in the measure(s) used to assess cognitive function. Global measures of cognition (e.g., Mini-Mental State Examination [MMSE], Folstein et al., 1975; Montreal Cognitive Assessment [MoCA], Nasreddine et al., 2005), for example, have been shown to suffer from ceiling and floor effects (Federico et al., 2018) and may not be suitable for detecting subtle, domain-specific differences in cognition between motor subtype groups (Hendershott et al., 2017). Moreover, clinician-rated assessments (such as the MMSE and MoCA) may be affected by rater bias and expertise (Riello et al., 2021), leading to unwanted variability between raters that could mask genuine cognitive differences between subtypes. Continuous measures of cognition (e.g., measures derived from the stop-signal task, Logan & Cowan, 1984) can offer greater sensitivity than discrete measures (e.g., prevalence rates of PD-MCI or PDD) and may therefore be more suitable for capturing subtype differences in the earlier stages of cognitive decline. Given this, the choice of cognitive assessment tool(s) could have a marked influence on the magnitude of subtype differences observed depending on their specificity and sensitivity.

### Research aims

This systematic review and meta-analysis aims to quantify the relationship between motor subtype and cognitive impairment in PD by synthesising the results of studies that have performed motor subtyping and assessed cognitive function in a PD cohort. Whilst there have been several recent reviews on subtyping in PD (Fereshtehnejad & Postuma, 2017; Marras, 2015; Marras & Lang, 2013; Mestre et al., 2021; Qian & Huang, 2019; van Rooden et al., 2010; von Coelln & Shulman, 2016), many of these are expert reviews that were not performed systematically (Fereshtehnejad & Postuma, 2017; Marras, 2015; Marras & Lang, 2013). Only one previous systematic review specifically investigated the relationship between motor subtype and cognition (Monaghan et al., 2023); this review focused exclusively on one motor subtyping framework (FOG vs. nFOG) and, to our knowledge, is the only review to apply meta-analytic techniques to the PD motor subtyping literature. Our review and multilevel meta-analysis seeks to build upon previous work by evaluating cognitive differences across *multiple* motor subtyping frameworks. Moreover, we aim to resolve inconsistent findings regarding the motor-cognitive relationship in PD by evaluating several methodological considerations (e.g., subtype method, cognitive assessment tool) and sample characteristics (e.g., disease duration, medication status) as potential effect size moderators. Our research questions are as follows:

1. Which subtyping methods are most commonly used?
2. What is the nature and strength of the relationship observed between motor and cognitive function in PD, as investigated by studies using motor subtyping methods?
3. Does the subtyping method used influence the motor-cognitive relationship observed?
4. Does the type of motor and/or cognitive assessment(s) used influence the motor-cognitive relationship observed?

## Methods

This review was conducted according to the Preferred Reporting Items for Systematic Reviews and Meta-Analyses (PRISMA; Page et al., 2021; for checklist, see Supplementary Material 1). This review is registered in the International Prospective Register of Systematic Reviews (PROSPERO; registration number CRD42022362290) and a review protocol was published *a priori *(Child et al., 2024).

### Eligibility criteria

#### Participants

To be eligible for inclusion, studies had to report on a sample of patients who had been formally diagnosed with PD by a neurologist according to established criteria (e.g., UK Brain Bank criteria (Gibb & Lees, 1988), Movement Disorder Society [MDS] clinical diagnostic criteria (Postuma et al., 2015). Studies including other cohorts (e.g., healthy controls, Dementia with Lewy Bodies [DLB]) were only eligible if they reported data for their PD sample separately. No limits were placed on participants’ demographics (age, sex, gender, ethnicity, education) or disease characteristics (e.g., disease duration, age at onset, medication status).

#### Assessments

##### Motor function

All included studies had to use at least one motor assessment tool that had been validated for use with people with PD and which measured one or more of the cardinal symptoms of PD (tremor, bradykinesia, muscle rigidity, impaired gait/postural stability). This assessment tool had to be objective and/or clinician-rated; studies that only measured motor symptoms via patient or caregiver report (e.g., Freezing of Gait Questionnaire; Giladi et al., 2000) were excluded.

##### Cognitive function

Similarly, all included studies had to use at least one objective or clinician-completed assessment of cognition. Both global and domain-specific measures of cognition were eligible for inclusion (for examples, see Table 2 of our review protocol; Child et al., 2024). In addition, studies that categorised participants according to their cognitive status (i.e., normal cognition [NC], PD-MDI, PDD) using cutoff scores and/or diagnostic criteria validated for use with people with PD were eligible for inclusion.

#### Study design

##### Subtyping methods

All included studies had to apply one or more motor subtyping methods to their PD sample. We adopted the definition used by Mestre et al., (2021, p. 397), who described a PD subtyping study as “any research study conducted with the purpose of dividing PD patients into subtypes, as stated by its authors, or identified by distinct groups of PD patients that were discussed as possible subtypes.” Studies were not eligible if they performed subtyping among multiple cohorts to differentiate PD patients—considered as a single group—from other groups (e.g., healthy controls). Because we were interested in evaluating differences in cognitive function between motor subtype groups, only studies that performed subtyping on the basis of *motor* symptoms—using objective and/or clinician-rated assessment tools—were eligible. Studies were permitted to use more than one motor assessment tool to derive their subtype groups but were excluded if their subtyping methods relied on a combination of motor and nonmotor measures.

In our published protocol (Child et al., 2024), we reported that we would exclude studies where participants were subtyped solely based on motor symptom asymmetry or side of motor symptom onset. We should clarify that we retained studies where both predominant motor symptom(s) *and* asymmetry/side of onset were used to derive subtype groups; for these studies, we collapsed groups with the same predominant motor symptom(s) prior to analysis to form subtype groups based on predominant motor symptom(s) only. For example, Chen et al. (2022) report on four motor subtype groups: left-side tremor, right-side tremor, left-side AR, and right-side AR. For the purposes of our review and meta-analysis, data for the left-side and right-side groups were pooled (weighted by sample size) to produce a tremor group and an AR group.

#### Study types

Only peer-reviewed original research studies published in English were included in our review. The following publication types were excluded: books, opinions/editorials, replies/commentaries, conference abstracts, posters, reviews, protocols, case studies, theses/dissertations, and unpublished/grey literature. All study design types were included (e.g., cohort, cross-sectional, longitudinal); however, longitudinal studies were only included if they reported cognitive data collected at baseline; no follow-up cognitive data were included in our meta-analyses.^1^ No limits were placed on publication year. Studies where the full text was unavailable were excluded.

#### Studies reporting on same or overlapping samples

**Fig. 1:**
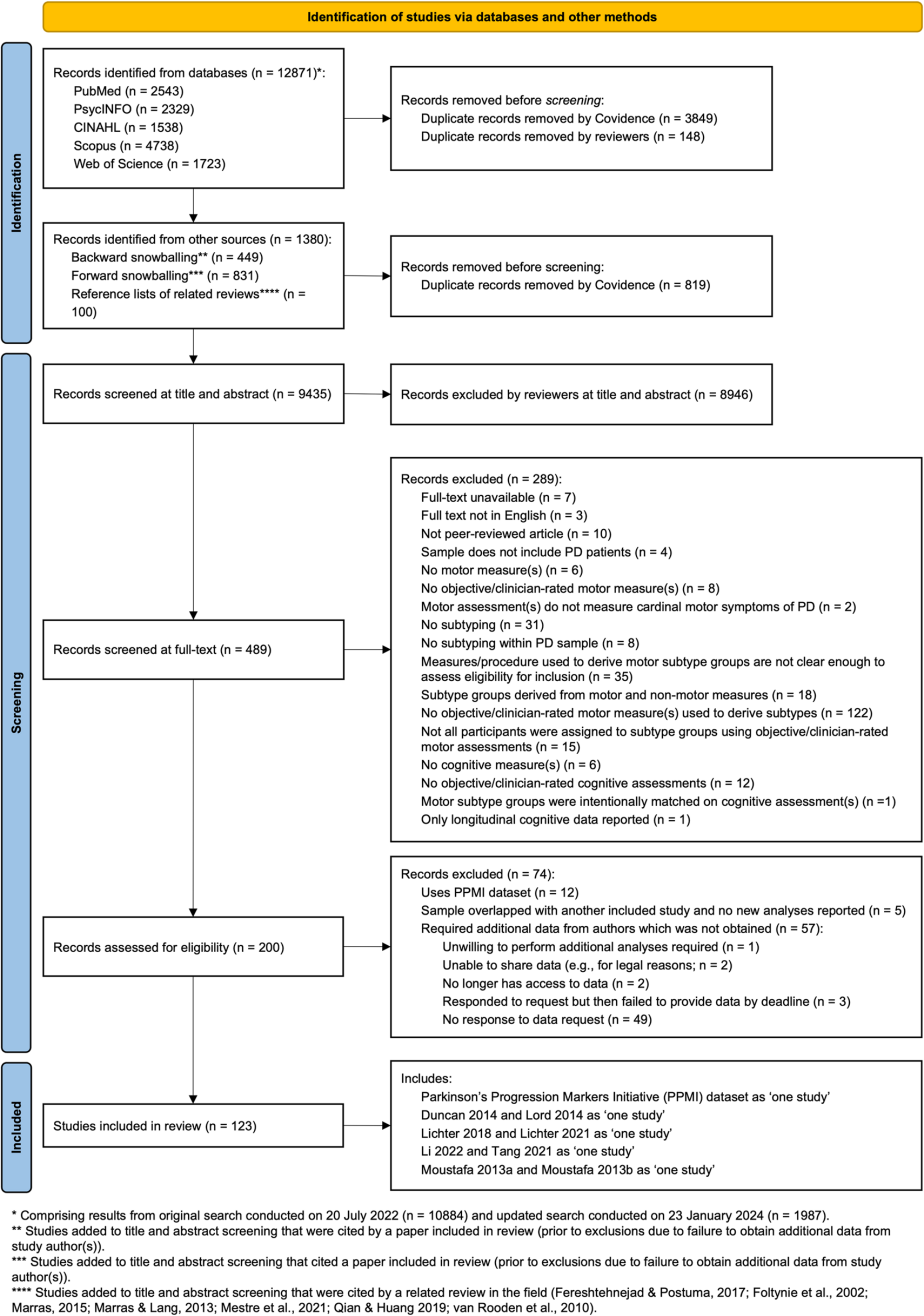
PRISMA flow diagram of the study selection process. *Note.* * Comprising results from original search conducted on 20 July 2022 (n = 10,884) and updated search conducted on 23 January 2024 (n = 1,987). ** Studies added to title and abstract screening that were cited by a paper included in review (prior to exclusions due to failure to obtain additional data from study author(s)). *** Studies added to title and abstract screening that cited a paper included in review (prior to exclusions due to failure to obtain additional data from study author(s)). **** Studies added to title and abstract screening that were cited by a related review in the field (Fereshtehnejad & Postuma, 2017; Foltynie et al., 2002; Marras, 2015; Marras & Lang, 2013; Mestre et al., 2021; Qian & Huang, 2019; van Rooden et al., 2010)

In our published protocol (Child et al., 2024), we reported that if two or more studies used data from the same or overlapping samples, we would retain the higher quality study (according to our quality assessment tool). After commencing data extraction and quality assessment, we determined that this decision rule was not appropriate as it would, in some cases, result in unnecessary data loss. Some studies, for example, reported on the same (or overlapping) sample but applied different subtyping methods, and so would never be included in the same meta-analysis (i.e., nonindependence of effect sizes would not be violated); in these cases, we retained both studies in the review.^2^ Other studies applied the same subtyping method to the same (or overlapping) sample but reported data from different cognitive assessments. Our proposed method for handling these cases would have again resulted in data being excluded unnecessarily. In these cases, we chose to collapse studies into a single study and included *all* cognitive assessments reported in the original studies (Fig. 1). For cases where two or more studies applied the same subtyping method(s) to the same (or overlapping) sample and reported on the same cognitive assessment(s), we applied our original decision rule of only retaining the study of highest quality.^3^ In cases where we mildly suspected that two or more studies were reporting on the same or overlapping samples, but this was not reported explicitly, the corresponding author(s) were contacted for clarification; if no response was received, we assumed no overlap in samples and retained all studies in our review.

Notably, we also excluded all studies eligible for inclusion in our review that analysed data from the openly available Parkinson’s Progression Markers Initiative (PPMI) dataset. To maximise the number of participants and number of cognitive assessment(s) from the PPMI dataset that could be used in our meta-analyses, we opted to analyse this dataset ourselves, applying custom data filtering methods (Supplementary Material 2) consistent with our review’s inclusion criteria.

### Search strategy

We searched the following online databases from inception: PubMed, PsycINFO (Ovid), CINAHL (EBSCOhost), Scopus, and Web of Science. Our search strategy for each database was developed in consultation with a research librarian with expertise in medicine and psychology. Key search terms were variations on PD, subtyping, motor function, and cognition. We included search terms and/or applied search filters to exclude animal-only studies and non-English studies, consistent with our eligibility criteria. We intentionally kept our search terms broad to increase the likelihood of capturing eligible studies (see Supplementary Material 2 for full search strategy for each database). In addition to database searches, we performed title screening on all studies that had been cited by one or more eligible studies (backward snowballing) and on all studies that had themselves cited one or more eligible studies (forward snowballing). We also searched the reference lists of key reviews in the field (Fereshtehnejad & Postuma, 2017; Foltynie et al., 2002; Marras, 2015; Marras & Lang, 2013; Mestre et al., 2021; Qian & Huang, 2019; van Rooden et al., 2010) for eligible studies.

### Data management

All search results were exported as .ris or .txt files and imported into Covidence (Veritas Health Innovation, Melbourne, Australia), which performs automatic de-duplication. Title and abstract screening, full-text screening, data extraction, and quality assessment were all completed in Covidence.

### Study selection

At both title and abstract screening and full-text screening, each record was screened by two independent, nonblinded reviewers. BC screened all records during each phase along with one of five other reviewers (AM, BE, IB, IS, RDS). Disagreements were resolved via discussion and, where necessary, arbitration by a third reviewer. For title and abstract screening, proportionate agreement between reviewers ranged from 93–95%, while Cohen’s kappa was weak (Supplementary Material 2, Table S1); for full-text screening, proportionate agreement ranged from 84–100% and Cohen’s kappa varied from weak to strong (Supplementary Material 2, Table S1; McHugh, 2012).

A detailed PRISMA flow diagram of the study selection process is provided in Fig. 1, and a list of references for all included studies is provided in Supplementary Material 2. Initial databases searches were conducted on 20 July 2022 and returned 7,696 results after de-duplication by Covidence. Database searches were re-run on 23 January 2024, prior to the commencement of data synthesis, and were returned an additional 1,197 results after de-duplication. A further 561 studies were identified from backward and forward snowballing methods and searching the reference lists of relevant reviews. After title and abstract screening, 489 of 9,435 studies proceeded to full-text screening; of these, 289 studies were excluded. Of the 200 studies deemed eligible for inclusion in our review, a further 74 were excluded. Most of these studies (*n* = 57 studies) were excluded because they did not report the critical data required (cognitive assessments reported for each motor subtype group) in their main text or supplementary material and were either unable to provide these data or did not respond to our data requests. Five studies were excluded due to the same sample or overlapping samples being reported on by more than one study, and 12 studies were excluded for analysing the PPMI dataset.

### Data extraction

A detailed summary of our approach to data extraction is provided in Supplementary Material 2, and our data extraction template can be found in Supplementary Material 3. One reviewer (BC) extracted data for all studies, and a second reviewer (IS) independently extracted data for 20% of all included studies (*n* = 24, selected at random). According to the threshold of ≥ 85% specified in our protocol, raw agreement between reviewers on critical fields was deemed satisfactory (85%). Any inconsistencies were resolved through discussion between reviewers. Data requests were sent to 139 corresponding authors for 155 studies missing critical cognitive outcome data. We received either some or all requested data for 31 studies.

### Quality assessment

Study quality was appraised using a quality assessment tool (Supplementary Material 4) that was developed specifically for this review. This tool was used to assess study quality in four domains: recruitment and sample characteristics; motor function measurement; cognitive function measurement; and statistical analysis and reporting. Scores on each domain were used to classify studies a study’s risk of bias as low, moderate, or high for each domain and overall (see Supplementary Material 2 for more information).

Quality assessment was conducted by one reviewer (BC) for all studies, and a second reviewer (IS) independently appraised the quality of 20% of all included studies (*n* = 24, selected at random). Intraclass correlations (ICC) were used to assess interrater agreement (see Supplementary Material 2, Table S2), and disagreements were resolved via discussion. Individual domain ICCs ranged from .57 (Domain 1: Recruitment and sample characteristics) to .87 (Domain 3: Cognitive function measurement), indicating moderate-to-good agreement according to Koo and Li’s (2016) guidelines. Overall interrater agreement for risk of bias across all domains was also moderate (ICC = .73).

### Data synthesis and analysis

#### Software

All effect size calculations and meta-analyses were performed with R Statistical Software (v4.3.2; R Core Team, 2023) using the metafor (v4.6–0; Viechtbauer, 2010), dmetar (v0.1.0, Harrer et al., 2019), clubSandwich (v0.5.11; Pustejovsky, 2024), orchard (v2.0; Nakagawa et al., 2023), metaviz (v0.3.1; Kossmeier et al., 2020), and robvis (V0.3.0.900; McGuinness & Higgins, 2021) packages. R Markdown files containing the R code used to conduct all analyses, along with data files and a detailed data dictionary, are available on OSF (https://osf.io/6ckwe/).

#### Effect size calculation

All studies included in our review used subtyping frameworks with either two (e.g., FOG vs. nFOG) or three (e.g., TD vs. PIGD vs. ID) motor subtype groups. Any effect size measure capturing differences between three groups would be difficult to interpret with respect to the *direction* of the effect; therefore, we chose to perform pairwise comparisons between motor subtype groups, regardless of whether the subtyping method used generated two or three groups. All subtype pairs are presented in Table 1. We calculated Hedges’ *g* as the effect size measure for continuous variables and risk ratios (RRs) for categorical variables.

**Table 1:** Motor subtype pairs included in review

Motor subtype pair	No. studies (*n*)	No. studies with continuous cognitive data	No. studies with categorical cognitive data
TD/PIGD	55	50	17
TD/ID	22	19	11
PIGD/ID	22	19	11
TD/AR	22	20	3
TD/MX	6	5	1
AR/MX	6	5	1
non-PIGD/PIGD	8	7	3
TD/NTD	10	10	1
p-TD/p-PIGD	2	2	-
FOG/nFOG	20	20	12
TR-Y/TR-N	2	1	2
TR/BR	5	5	-
Fallers/nonfallers	1	1	-
Bradykinesia/no-bradykinesia	1	1	-
Poor gait/good gait	5	4	1
With facial tremor/without facial tremor	1	1	-

*Note.* AR = akinetic-rigid; BR = bradykinesia; FOG = freezing of gait; nFOG = no freezing of gait; non-PIGD = nonpostural instability gait disorder; NTD = nontremor-dominant; PIGD = postural instability and gait disorder; p-PIGD = predominantly postural instability and gait disorder; p-TD = predominantly tremor-dominant; TD = tremor-dominant; TR = tremor; TR-N = no tremor; TR-Y = tremor. The sum of number of studies with continuous cognitive data and number of studies with categorical cognitive data sometimes exceeds the total number of studies (*n*) as some studies reported both continuous and categorical cognitive data

##### Hedges’ *g*

Hedges’ *g* captures the standardised mean difference (SMD) between two groups and weights each group’s standard deviation by its sample size, allowing for correction of a potential upward bias that can occur when Cohen’s *d* is calculated for small samples (Borenstein et al., 2009; Ellis, 2010).

For all continuous cognitive outcome measures, Hedges’ *g* was calculated using the escalc function in R (from the metafor package, Viechtbauer, 2010), which requires the means, standard deviations, and sample sizes for each group. Where author contact was unsuccessful and these values were not reported in the main text or supplementary material, every effort was made to estimate these values from the available data. Where only medians and inter-quartile ranges were available, Wan et al.’s (2014) formula was used to estimate means and standard deviations from these values; where standard error was reported in place of standard deviation, values were converted using the formula $SD=SE \times \surd n$ (Higgins et al., 2024); and where means and standard deviations were only reported visually, the WebPlotDigitizer 5.2 web tool (Rohatgi, 2024) was used to estimate exact values. As aforementioned, where one or more motor subtype groups had to be collapsed for inclusion in our review (e.g., combining left-side tremor and right-side tremor groups to form a tremor group), the means and standard deviations of the constituent groups were pooled, weighted by the sample size of each group. For one study (Tolleson et al., 2017), only Cohen’s *d* values were reported for some cognitive measures; these values were converted to Hedges’ *g*. A note was made in all instances where these estimation methods were used (Supplementary Material 5).

For reverse-scored cognitive measures (where lower scores reflected better performance, e.g., reaction time), Hedges’ *g* values were multiplied by − 1 to ensure that the directionality of effect was consistent across all effect sizes.

##### Risk ratio

We calculated the RR as an effect size measure of motor subtype differences in cognitive status.^4^ Specifically, we calculated RRs capturing the association between motor subtype group and MCI status (relative to NC) and/or RRs capturing the association between motor subtype group and dementia status (relative to NC). We did not compute an effect size estimate capturing a 2× 3 comparison between motor subtype group and cognitive status (i.e., NC vs. MCI vs. dementia), as RRs cannot be computed for 2 × 3 comparisons.

Two studies (Lichter et al., 2018, 2021 as one study; Santos-García et al., 2020) reported the number of dementia cases in their sample but also reported dementia as an exclusion criterion. For example, Santos-García et al. (2020) reported dementia, operationalised as an MMSE score < 26, as an exclusion criterion but subsequently reported the number of dementia cases in their sample, operationalised as PD Cognitive Rating Scale scores ≤ 64. Given that this seemed contradictory, dementia count data from these two studies were excluded. Both studies also reported the number of MCI cases in their samples; these data were retained.

Where our data request attempts were unsuccessful and we did not have sufficient data available to calculate either Hedges’ *g* or a RR for at least one cognitive outcome measure, the study was excluded (Fig. 1; Supplementary Material 5).

#### Traditional (two-level) meta-analyses

##### Demographics and disease characteristics

Given the large number of studies included in our review, we were able to produce a rich dataset capturing not only differences in cognitive performance between motor subtype groups, but also differences in demographics and disease characteristics, such as age, sex, disease duration, and levodopa equivalent daily dose (LEDD). Whilst we had intended to investigate whether these sample characteristics moderated the difference in cognition between motor subtype groups, it became apparent that meaningful insights could be gained from evaluating these subtype differences in their own right. We therefore ran a series of traditional (two-level) random effects meta-analyses in which motor subtype pairs were compared on the following demographics and disease characteristics: age, gender (proportion of men), years of education, disease duration, age at disease onset, and LEDD. We also compared subtype pairs on UPDRS-III and MDS-UPDRS-III total scores (considered both separately and together), to evaluate subtype differences in *overall* motor impairment. Hedges’ *g* was calculated as the effect size measure for all variables except for proportion of males, for which RRs were calculated.^5^ Given that only one effect size could be computed per study for each of these variables, a multilevel meta-analytic approach was not required. Meta-analyses were only performed when data were available for at least three studies, and studies were excluded if they reported having intentionally matched motor subtype groups on the characteristic being meta-analysed.

Outliers were identified by using the find.outliers function in R (from the dmetar package; Harrer et al., 2019), where studies are defined as outliers when the 95% confidence interval around their effect size estimate lies outside the 95% confidence interval of the pooled effect size. This function automatically re-runs the meta-analysis model with outliers removed to ascertain any differences in pooled effect size estimates following outlier removal. Publication bias was investigated via inspection of funnel plots and Pustejovsky and Rodgers’ (2019) method for performing Egger's test on SMDs (Egger et al., 1997). This method uses a different formula to calculate effect size standard error, as Pustejovsky and Rodgers’ (2019) found that using Egger’s test with SMDs can inflate the false positive rate. Egger’s test was not performed for meta-analyses with fewer than ten studies.

##### Cognitive status (categorical data)

We meta-analysed categorical cognitive outcome data where data were available for at least three studies; given the small number of studies included in each meta-analysis (*n* range = 6–15 studies), no subgroup analyses or meta-regressions were conducted. Where data were available for fewer than three studies, results were narratively synthesised (see Narrative Synthesis). For all studies included in our meta-analyses of categorical cognitive data, no more than one effect size was computed per study for each categorical cognitive comparison of interest (NC vs. MCI, NC vs. dementia). Given this, traditional (two-level) random effects meta-analyses were performed. Outlier detection and publication bias followed the same procedure as outlined in the previous section (see Demographics and disease characteristics).

#### Multilevel (three-level) meta-analyses

Of 123 studies, 81 studies reported more than one effect size (for the same subtype pair) from continuous measures of cognitive function, resulting in nonindependent effect sizes. Effect size dependency in meta-analysis is often addressed by (1) choosing one effect size per study based on some criterion; (2) meta-analysing the average effect size of each study; or (3) running multiple meta-analyses wherein within-study effect sizes are separated across analyses (Gucciardi et al., 2022). Each of these strategies are suboptimal, given that they result in information loss and restrict the exploration of sources of within-study heterogeneity (López‐López et al., 2018). Given this, we chose to analyse our continuous cognitive data using multilevel meta-analytic techniques, which allow for nonindependent effect sizes (i.e., multiple effect sizes from the same study) to be included in the same model (Assink & Wibbelink, 2016; Gucciardi et al., 2022).

In a traditional random effects meta-analysis, random effects are assigned at the study level, with participants nested within each study. Effect size variance is attributed to sampling error of individual studies and between-study heterogeneity (Harrer et al., 2019).^6^ In multilevel meta-analysis models, a third level is introduced to model within-study (unique effect size) heterogeneity alongside sampling error and between-study (study level) heterogeneity, capturing dependencies between effect sizes (López‐López et al., 2018; Van Den Noortgate et al., 2013). Variance–covariance matrices were imputed to account for within-study effect dependency at an estimated constant correlation of 0.3 (using the impute_covariance_matrix function in R from the clubSandwich package; Pustejovsky, 2017).

Overall heterogeneity was inferred through *I*^*2*^ and *Q* statistics. The amount of variance attributable to within-study heterogeneity and between-study heterogeneity was calculated by using Cheung’s (2014) formula (implemented using the var.comp function in R from the dmetar package; Harrer et al., 2019). Multilevel meta-analyses were only performed where effect sizes were available from at least three studies; where data were available for fewer than three studies, results were narratively synthesised (see Narrative Synthesis).

##### Outcome prioritisation

Notably, as a consequence of implementing multilevel meta-analysis, much of our planned approach to outcome prioritisation (outlined in our published protocol; Child et al., 2024) became redundant. We did not prioritise domain-specific cognitive measures over global measures of cognition, and we did not prioritise objective cognitive measures over clinician-rated measures. We had also planned to prioritise continuous cognitive data over categorical data capturing participants’ cognitive status (e.g., NC, PD-MCI, PDD). Because we ultimately chose to analyse our continuous and categorical cognitive data separately, such prioritisation was not necessary. Thus, where studies reported both continuous and categorical cognitive data, all data were extracted and included in the corresponding meta-analysis.

Where a study reported multiple measures from a single cognitive task, we only retained the most representative measure for each cognitive domain (see Categorical moderators for more information on cognitive domain classification) for meta-analysis. We considered the most representative measure to be the one whose operationalisation most closely aligned with the established conceptual definition of the cognitive domain being measured.^7^ This allowed us to maintain consistency in effect size dependencies within studies (i.e., two effect sizes measuring the same cognitive domain taken from the *same* task would be more highly correlated than two effect sizes measuring the same cognitive domain taken from *different* tasks). For global measures (e.g., MoCA, MMSE), in instances where both the total score and domain-specific subscores were reported, we retained only the total score, given that domain subscores have limited sensitivity due to the few items used per domain. However, if only domain-specific subscores were reported, these were retained. For the NeuroTrax Mindstreams cognitive assessment system (Dwolatzky et al., 2003) and the Seoul Neuropsychological Screening Battery (SNSB; Ryu & Yang, 2023), we prioritised domain composite scores over individual task measures and over a single global composite score. We also took the same approach to one study (Karunanayaka et al., 2016) that calculated domain composite scores from their neuropsychological assessment battery.

##### Outliers and publication bias

We used two methods for identifying outliers in our multilevel meta-analyses, namely inspection of residuals (*z*-scores less than −1.5 and greater than 1.5 were considered outliers) and inspection of Cook’s distance (values greater than 1.5 multiplied by the average Cook’s distance value across individual effects were considered outliers). All multilevel meta-analysis models were re-run with outliers removed to ascertain any differences in pooled effect estimates. Publication bias was investigated using a multilevel extension of Egger’s test (Fernández-Castilla et al., 2021) and inspection of sunset (power-enhanced) funnel plots (Kossmeier et al., 2020). The multilevel Egger’s test re-runs the multilevel meta-analysis with effect size standard error as a moderator; if the moderator analysis is significant, this indicates that publication bias is likely (Fernández-Castilla et al., 2021). Sunset funnel plots are a variation of conventional funnel plots wherein statistical power is also visualised using colour regions (ranging from dark red for highly underpowered studies to dark green for appropriately powered studies; Kossmeier et al., 2020).

##### Dose–effect analyses

If there is a genuine difference in cognitive function between motor subtype groups, it follows that studies where the participants who are assigned to each subtype group more strongly reflect the characteristics of that subtype may report larger effect sizes. In other words, studies where participants tend to reflect the “extremes” of the motor subtype groups (e.g., severe tremor vs. no tremor, severe FOG vs. no FOG) may be more powered to detect an effect. Given this, we sought to conduct dose–effect analyses where we considered whether the magnitude of the group *difference* on a relevant dimension of motor function moderated the effect size for the difference in cognitive function. This would effectively demonstrate a dose effect whereby larger group differences in motor function are associated with larger differences in cognitive function. For pairwise comparisons with subtype groups taken from the TD/PIGD/ID subtyping framework, we conducted dose–effect analyses where the SMD (Hedges’ *g*) in UPDRS (any version) tremor subscore or the SMD in UPDRS (any version) PIGD subscore was entered as a moderator. For pairwise comparisons with subtype groups taken from the TD/AR/MX subtyping framework, we conducted dose–effect analyses where the SMD (Hedges’ *g*) in UPDRS (any version) tremor subscore was entered as a moderator (insufficient data were available to run this analysis using the SMD in UPDRS [any version] AR subscore). This approach was identical to our approach to analysing all other continuous moderators, detailed below (see Continuous moderators).

##### Moderator analyses

According to Hunter and Schmidt (2004), heterogeneity is substantial if less than 75% of total variance can be attributed to sampling variance; in these cases, it may be useful to examine the influence of potential moderators on the overall effect. Several study and sample characteristics were considered as possible moderators; these are listed in Tables 2 and 3. Moderator analyses were not conducted for motor subtype pairs where moderator data were available for fewer than ten studies; for categorical moderators, we excluded moderator levels where data were only available for one study.

**Table 2:** List of categorical moderators

Categorical moderator	Levels
Subtyping method	Jankovic^†^, Stebbins, Kang, other
Cognitive class (global, specific)	global^†^, specific
Cognitive domain	global cognitive function^†^; executive function – cognitive flexibility; executive function – attention; executive function – cognitive inhibition; executive function – response inhibition; executive function – working memory; higher-order fluid abilities – planning; composite IQ; language; LTM/learning – lexical; LTM/learning – semantic; LTM/learning – visuospatial; LTM/learning – other; STM – lexical; STM – numerical; STM – visuospatial; STM – other; processing speed; visuospatial abilities – construction; visuospatial abilities – perception; visuospatial abilities – reasoning;
Medication status for cognitive assessment(s)	ON, OFF, ON or OFF, de novo^†^
Medication status for motor assessment(s)	ON, OFF, ON or OFF, de novo^†^
Cognitive impairment/dementia as exclusion criterion	yes, no^†^
Motor subtype groups compared on cognition in paper^*^	yes, no^†^
Overall study risk of bias	low, moderate, high^†^

*Note.* LTM = long-term memory; STM = short-term memory. ^*^ Reflects whether critical data (motor subtype x cognition comparison) were reported in the main text or supplementary materials (yes) or whether these data had to be requested from corresponding author (no). ^†^ Denotes reference category

**Table 3:** List of continuous moderators

Continuous moderators
Sample size
Year of publication
Pooled mean age
Pooled proportion of males
Pooled mean years of education
Pooled mean disease duration (years)
Pooled mean age at disease onset (years)
Pooled mean LEDD
Pooled mean UPDRS-III score
Pooled mean MDS-UPDRS-III score
Pooled mean UPDRS-III score (any version)

*Note.* LEDD = levodopa equivalent daily dose; MDS-UPDRS-III = Movement Disorder Society Unified Parkinson’s Disease Rating Scale Part III; PIGD = postural instability and gait disorder; UPDRS-III = Unified Parkinson’s Disease Rating Scale Part III. Pooled means and proportions were calculated by calculating the average mean or proportion of the two subtype groups, weighted by the sample size of each group

##### Continuous moderators

We followed Assink and Wibbelink’s (2016) procedure for running multilevel meta-analyses with continuous moderators. The test of moderators was used to determine whether a moderator was statistically significant; if significant, the regression coefficient was then inspected to determine the size and direction of the moderator’s influence on the main effect. Bubble plots were used to visualise results. We also re-ran the original meta-analysis model (without moderator) with only those studies that reported moderator data, thereby producing comparable with- and without-moderator models. This enabled us to examine the change in between-study heterogeneity by subtracting the with-moderator between-study *I*^*2*^ from the without-moderator between-study *I*^*2*^. This change in between-study heterogeneity reflects how much of the effect size variance is captured by the moderator.

##### Categorical moderators

Multilevel meta-analyses with categorical models were performed using an “inner | outer” formula, with random effects specified at the unique effect size level. This formula assumes that different studies (outer factor) are independent, while effects within the same study share correlated random effects with a level of variance corresponding to levels of the moderator (inner factor; Viechtbauer, 2010). The “inner | outer” formula requires that a variance structure corresponding to the inner factor (moderator) be specified. We opted for a heteroscedastic compound symmetric structure that fixes the amount of heterogeneity for the levels of the inner factor to the corresponding inner | outer formula (Viechtbauer, 2010). We considered this variance structure to be most suitable as it strikes a balance between theoretical appeal and feasibility with the extracted dataset.

For categorical moderators, one moderator level was chosen as the reference category (marked with a cross in Table 2), producing beta coefficients for other moderator levels. This allowed us to evaluate whether effects for other moderator levels differed significantly from the reference category. For example, for our TD vs. PIGD comparison, “Jankovic” was the reference category for subtyping method, meaning that beta coefficients were calculated for “Stebbins” and “Other” to quantify the extent to which pooled effect estimates for studies using these subtyping methods varied from the pooled effect size estimate for studies using Jankovic et al.’s (1990) subtyping method. Pooled effect size, 95% confidence intervals, and *p*-values for moderator levels other than the reference category were obtained by re-running the model without the intercept. Orchard plots (Nakagawa et al., 2023) were used to visualise categorical moderator analyses.

Our cognitive domain classification approach aimed to balance empirically validated theoretical frameworks with clinical utility. The classification favoured empirically derived domains established through psychometric and neuroimaging studies (e.g., Diamond's (2013) executive function framework; Miyake and Friedman's (2012) unity/diversity model; Wechsler’s and Cattell's broader cognitive ability structures (see Schneider & McGrew, 2018)). However, we also incorporated elements of clinically-oriented taxonomies (e.g., Lezak's et al. (2012) neuropsychological framework) to enhance practical utility and accessibility for clinicians. This approach allowed us to maintain neurobiological validity, which is particularly important given PD’s neurological basis, while ensuring the classification system remained clinically interpretable. For example, whereas “attention” as a cognitive domain presents definitional challenges in empirical frameworks, we retained it as a distinct category for specific measures that could not be clearly classified within other executive function domains, acknowledging its clinical significance in neuropsychological assessment.

##### Confounds

We also considered how the magnitude of the *difference* in demographics and disease characteristics between motor subtype pairs might account for subtype differences in cognitive performance. For example, larger effect sizes may be observed in studies where the PIGD group is markedly older than the TD group, simply by virtue of age-related cognitive decline contributing to poorer cognitive performance in the PIGD group, rather than genuine motor subtype differences. To explore this possibility, we ran a series of moderator analyses where the moderators were the SMD (Hedges’ *g*) between groups in age, years of education, disease duration, age at disease onset, LEDD, and UPDRS-III total score (both versions, considered separately and together). We also ran a moderator analysis with the raw difference in proportion of men between subtype groups. For these analyses, we were less interested in whether the pooled effect size for cognition varied with the size of the moderator (confound) but rather interested in whether any unexplained between-study variance remained after including the confound in our model. A substantial reduction in between-study variance (*I*^*2*^) following the inclusion of a confound in our model was interpreted as indicating that any observed cognitive difference between motor subtypes was primarily being driven by the confound, not motor subtype. Conversely, if between-study variance (*I*^*2*^) remained high after including the potential confound as a moderator, we concluded that the confounding variable (e.g., SMD in age between TD and PIGD groups) could not explain the observed difference in cognitive function. Dose–effect, moderator, and confound analyses were only conducted when data were available for at least ten studies.

#### Narrative synthesis

We did not perform meta-analyses comparing motor subtype group pairs where the number of included studies with available cognitive data (continuous or categorical, considered separately) was less than three. In these cases, narrative synthesis (Popay et al., 2006) was used to evaluate differences in cognition between motor subtype groups.

#### Risk of bias plots

Risk of bias was visualised using bar plots (McGuinness & Higgins, 2021). These plots were generated for all included studies and for each motor subtype pair.

### Confidence in cumulative evidence

An adapted version of the Grading of Recommendations Assessment, Development and Evaluation (GRADE) framework (Guyatt et al., 2011; Huguet et al., 2013) was used to determine confidence in cumulative evidence for individual motor subtype pairs where continuous cognitive data were available from ten or more studies^8^ (Table 1). Confidence in cumulative evidence for all included studies was not considered, as we did not consider this to be meaningful, nor did we consider it meaningful to establish confidence in cumulative evidence for subtype pairs with few (< 10) included studies. As GRADE was originally designed for reviews and guidelines of interventions, we followed Huguet et al.’s (2013) adapted GRADE framework for prognostic studies.^9^

The GRADE quality of evidence framework has four quality classifications: very low, low, moderate, and high. According to Huguet et al. (2013), the phase of investigation should be used to determine the initial quality of evidence classification; this classification can then be downgraded or upgraded based on six factors. Given the lack of consensus on the strength of the relationship between motor and cognitive function, we considered the study of the relationship between motor subtype and cognition in PD to be in an early, mostly exploratory, phase of investigation; as such, quality of evidence for all subtype pairs was initially classified as moderate.

We subsequently downgraded the quality of evidence by one level for serious limitations in each of the following areas: study limitations (defined as most studies having a moderate or high risk of bias for most domains of our quality assessment tool); inconsistency (defined as sum of between- and within-study *I*^*2*^ ≥ 50% for multilevel meta-analysis); indirectness (defined as limited coverage of cognitive domains and/or most studies only assessing global cognition, and/or most studies reporting on an exclusively de novo [unmedicated] or early onset sample); imprecision (defined as a pooled effect size 95% confidence interval > 0.5 for *g*); and publication bias (defined as a significant multilevel Egger’s test). Quality of evidence was upgraded by one level each for a moderate-to-large pooled effect (*g* ≥ 0.4) and the presence of an exposure-gradient response. For the TD/PIGD/ID and TD/AR/MX subtype frameworks, we considered an exposure-gradient response to be present where sufficient data (i.e., *n* > 10 studies) were available to perform dose–effect moderation analyses using the tremor and/or PIGD subscores typically used to classify patients into subtype groups *and* at least one of these moderation analyses was statistically significant. Given the inconsistent subtyping methods used to classify patients under other subtyping frameworks (e.g., FOG vs. nFOG), no exposure-gradient response was considered.

## Results

### Study characteristics

After exclusions, 123 studies were eligible for inclusion in our review, resulting in a pooled sample of 22,010 participants with PD. Continuous cognitive data were available for 113 studies (*o* = 20,289 participants) and categorical cognitive data were available for 27 studies (*o* = 7,369 participants). Across studies, sample size varied considerably (range = 14–2,224 participants, *M* = 179, *SD* = 314 participants); studies that reported categorical cognitive outcome data tended to recruit larger samples (*M* = 273, *SD* = 323 participants) than studies that reported continuous cognitive outcome data (*M* = 180, *SD* = 325 participants). Studies predominantly originated from China (34 studies), the United States (16 studies), Italy (14 studies), and the United Kingdom (10 studies). Publication dates ranged from 1991 to 2024, with more than half of included studies published after 2018, reflecting increased interest in PD subtyping.

With respect to subtyping methods, Jankovic et al.’s (1990) subtyping method, which produces TD, PIGD, and ID groups, was the most frequently applied method (*n* = 39 studies). Stebbins et al.’s (2013) method, which adapts Jankovic et al.’s (1990) method for use with the MDS-UPDRS, was the next most common subtyping method (*n* = 25 studies). Kang et al.’s (2005) subtyping method, which produces TD, AR, and MX groups, was used in 11 studies. Fifty-four studies used other subtyping methods; of these, 20 produced FOG and nFOG groups. When subtyping methods that produced TD, PIGD, and ID groups were used, it was common for researchers to exclude ID participants from their analyses and only report on differences between the TD and PIGD groups. For example, of 50 studies with continuous cognitive outcome data available for TD and PIGD groups, only 19 reported these same data for the ID group. Similar trends emerged for studies utilising subtyping methods that produced TD, AR, and MX groups; of 20 studies with continuous cognitive data for TD and AR groups, only five reported these same data for the MX group.

Among studies reporting continuous cognitive data, 106 studies reported global measures of cognition, and 56 studies reported domain-specific measures of cognition. Working memory (*n* = 32) was the most commonly assessed cognitive domain, followed by cognitive flexibility (*n* = 23) and language (*n* = 23). Of the global cognitive measures used in studies reporting continuous cognitive outcome data, the MMSE (*n* = 69) and MoCA (*n* = 53) were the most common, followed by the Frontal Assessment Battery (FAB; *n* = 21).

Among studies reporting cognitive status data (i.e., NC, MCI, dementia), there was considerable heterogeneity in the methods used to determine participants’ cognitive state. Use of MoCA cutoff scores (*n* = 7) was the most popular method; however, the cutoff score(s) applied varied between studies. For example, Zhang et al. (2016) used a cutoff score of < 26 for PD-MCI, whereas Baig et al. (2015) applied a more conservative range of 22–23 for PD-MCI (and < 22 for PDD). Other studies used cutoff scores in conjunction with other screening tools (e.g., MMSE, Scales for Outcomes in Parkinson’s Disease – Cognition [SCOPA-COG], Cambridge Cognition Examination [CAMCOG]) or applied formal diagnostic criteria (e.g., Level II MDS criteria for PD-MCI, clinical diagnosis of dementia using Diagnostic and Statistical Manual of Mental Disorders [DSM] criteria). One study (Ortelli et al., 2019) took a domain-specific approach and classified patients as cognitively normal or impaired (based on normative data) on a series of individual, domain-specific tasks (i.e., Wisconsin Card Sorting Test, Rey Auditory Verbal Learning Test).

### Pooled sample characteristics

Mean participant age was 65.3 years (*SD* = 4.0, *n* = 111). Across individual studies, samples tended to include more men (59%, *n* = 100) than women, consistent with PD being more common among men (Zirra et al., 2023). Mean age at disease onset was 59.5 years (*SD* = 4.8; *n* = 107) and participants had a mean disease duration of 5.8 years (*SD* = 3; *n* = 107) at the time of study participation. Other demographics and disease characteristics are presented in Table 4.

**Table 4:** Pooled sample demographics and disease characteristics

Characteristic	*M* (*SD*)	Median	Range	*n*
Age	65.3 (4)	65.2	57.5–77.2	111
Proportion of men	.59 (.1)	.59	.32–.82	100
Education (years)	11.3 (2.7)	10.8	5.7–16.5	64
Disease duration (years)	5.8 (3)	5.6	0.2–13.6	107
Age at onset (years)	59.5 (4.8)	59.5	44.7–70.8	107
LEDD	538.9 (247.2)	517.9	131.9–1,379.4	71
UPDRS-III	24.5 (8.9)	22.7	11.2–56.1	66
MDS-UPDRS-III	30.0 (8.4)	30.8	13.1–52.4	35

*Note.* LEDD = levodopa equivalent daily dose; MDS-UPDRS-III = Movement Disorder Society Unified Parkinson’s Disease Rating Scale Part III; *n* = number of studies; UPDRS-III = Unified Parkinson’s Disease Rating Scale Part III

Forty studies (32.5%) conducted motor subtyping assessment(s) whilst participants were in the ON state; 31 (25.2%) conducted these assessments while participants were OFF medication; 11 studies (8.9%) assessed participants across mixed medication states (e.g., some participants ON medication, others OFF medication or de novo); and 14 studies (11.4%) only recruited de novo (unmedicated) participants. Twenty-seven studies (22%) did not clearly report medication status at the time of motor assessment. Reporting of medication status at the time of cognitive assessment was much poorer, with 52 studies (42.3%) not reporting this information. Among studies for which this information was available, the majority assessed participants ON medication (*n* = 37; 52.1%). Twelve studies (16.9%) assessed participants OFF medication and eight (11.3%) studies assessed participants across mixed medication states.

Sixty-two studies (50.4%) reported one or more exclusion criteria relating to cognitive impairment or dementia (e.g., Hurt et al. (2014) excluded participants who had “severe cognitive impairment”). Unsurprisingly, such exclusion criteria were much more common in studies reporting continuous cognitive outcome data (*n* = 60; 53.1%) than in studies assessing the presence of MCI or dementia (*n* = 9; 33.3%).^10^ Only four studies (Basaia et al., 2022; D’Iorio et al., 2019; Gan et al., 2023; Karunanayaka et al., 2016) reported intentionally matching patients in each motor subtype group on one or more demographics or disease characteristics. All four studies matched participants on age and gender/sex; of these, two additionally matched participants on education years and one matched participants on years of education, disease duration, and overall motor impairment (according to total MDS-UPDRS-III score). No studies matched participants on LEDD or age at disease onset.

### Tremor-dominant vs. postural instability gait disorder

#### Demographics and disease characteristics

The results of traditional meta-analyses comparing tremor-dominant (TD) and postural instability and gait disorder (PIGD) motor subtype groups on demographics and disease characteristics are reported in Supplementary Material 2, Table S3. All pooled effect sizes indicated negligible-to-small differences between groups, except for LEDD and motor symptom severity. Compared with TD patients, PIGD patients were older (*k* (*o*) = 47 (9,232), *g* = 0.2 [0.12–0.29], *p* <.001) and tended towards a longer disease duration (*k* (*o*) = 43 (8,661), *g* = 0.17 [0.08–0.26], *p* <.001). These results are consistent with longitudinal research studies, which have shown that patients tend to transition from a TD to PIGD subtype over time, and rarely vice versa (Alves et al., 2006). As expected, LEDD was higher in PIGD patients compared with TD patients (*k* (*o*) = 30 (6,517), *g* = 0.41 [0.32–0.49], *p* <.001). In line with the common assumption that the PIGD subtype captures greater overall motor symptom severity (Aleksovski et al., 2018), PIGD patients scored significantly higher on the original UPDRS-III (Fahn et al., 1987) than TD patients (*k* (*o*) = 26 (4,580), *g* = 0.34 [0.19–0.49], *p* <.001), indicating more severe motor impairment. Interestingly, this difference was much less pronounced when the MDS-UPDRS-III (Goetz et al., 2008) was used (*k* (*o*) = 20 (4,575), *g* = 0.11 [0.03–0.25], *p* =.118).

#### Cognition

##### Main analyses

**Fig. 2:**
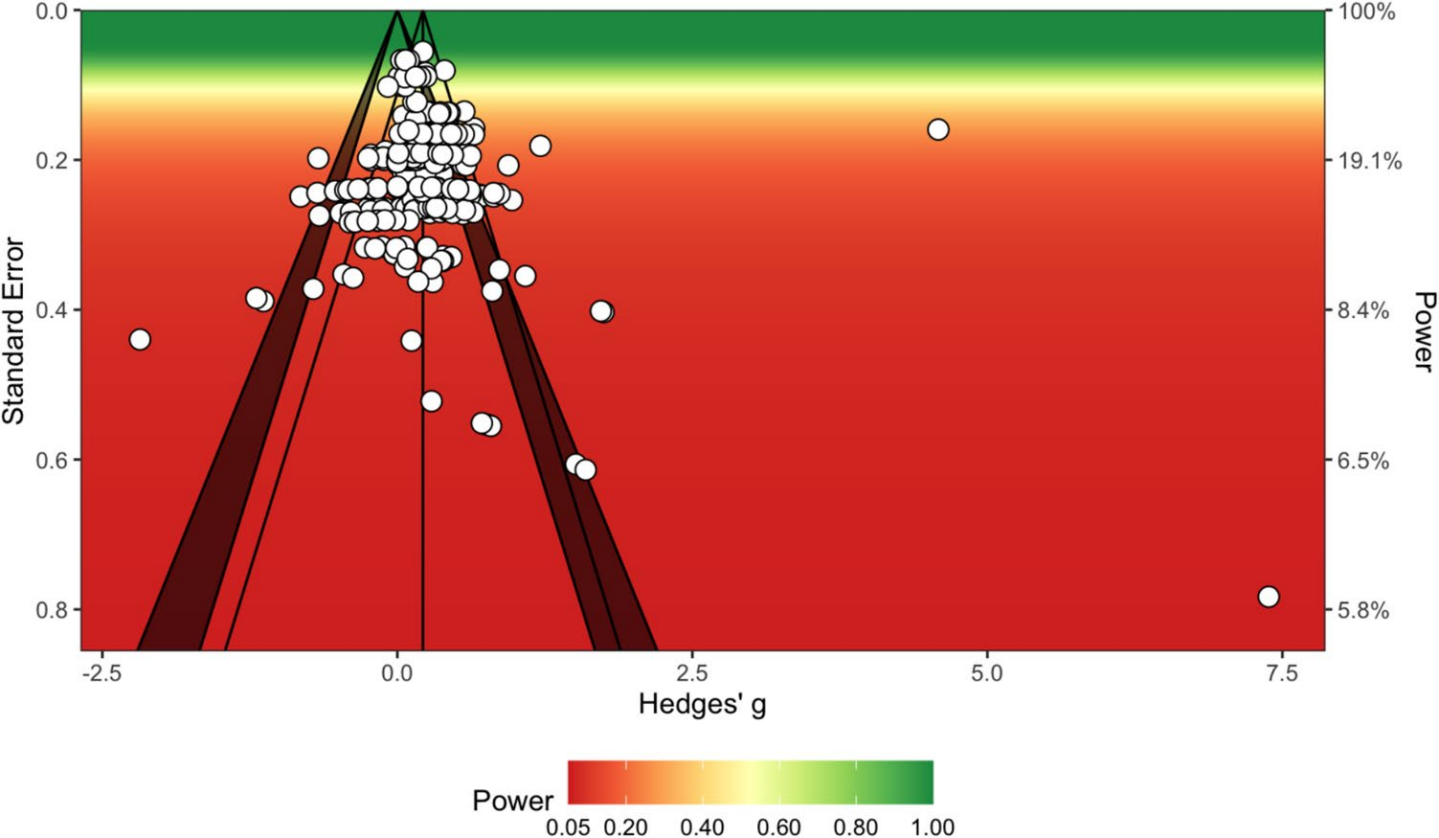
Sunset (power-enhanced) funnel plot for difference in cognitive performance between tremor-dominant (TD) and postural instability gait disorder (PIGD) motor subtype groups (no outliers removed)

A multilevel meta-analysis of 252 effect sizes from 50 studies indicated a small difference in cognitive performance between TD and PIGD patients, favouring TD patients (Table 5). Significant heterogeneity was present in the model, as indicated by *I*^*2*^ and *Q* statistics. Somewhat surprisingly, within-study variance (53.39%) was much greater than between-study variance (34.03%). We speculated that such marked within-study variance could be attributable to the varied cognitive domains assessed by the cognitive test batteries often employed by studies that reported more than one effect size; that is, TD and PIGD groups may display more marked cognitive differences in certain cognitive domains compared with others, magnifying the variance among effect sizes reported *within* individual studies assessing multiple domains (see Moderator analyses). Removal of outliers did not substantially change the pooled effect size. A multilevel Egger’s test indicated evidence of publication bias when all effect sizes were included in our model. Inspection of a sunset (power-enhanced) funnel plot (Fig. 2) suggested a tendency for studies reporting larger cognitive differences favouring TD patients to be published, but this bias appears to have been driven by only a small handful of especially large effect sizes. When these outliers were removed, multilevel Egger’s tests indicated no evidence of publication bias (Table 5).

**Table 5:** Results of multilevel meta-analyses of cognitive performance in tremor-dominant (TD) and postural instability gait disorder (PIGD) motor subtype groups

Subtype pair	Model	*n* (*k*)	Pooled Hedges’ *g* (95% CI)	*p*	Within-study *I*^*2*^ (%) (*p*)	Between-study *I*^*2*^ (%) (*p*)	*Q* (*p*)	Multilevel Egger’s test (*p*)
TD vs. PIGD	All studies	50 (252)	0.26 (0.14–0.38)	** <.001**	53.39 (**<.001**)	34.03 (**<.001**)	1526.18 (**<.001**)	23.74 (**<.001**)
	With outliers removed – residuals approach	50 (242)	0.23 (0.16–0.3)	** <.001**	14.23 (**.001**)	40.83 (**<.001**)	411.38 (**<.001**)	0.04 (.834)
	With outliers removed – Cook’s distance approach	48 (248)	0.21 (0.14–0.27)	** <.001**	36.52 (**<.001**)	18.14 (**.022**)	536.45 (**<.001**)	0.01 (.926)

*Note*. CI = confidence interval; *k* = number of effect sizes;* n* = number of unique studies; PIGD = postural instability gait disorder; TD = tremor-dominant. For Hedges’ *g*, values > 0 indicate poorer cognitive performance among PIGD patients relative to TD patients. Significant *p*-values (<.05) are highlighted in bold

Given the presence of publication bias in our full model, we ran our dose–effect, moderator, and confound analyses with all available effect sizes and then repeated these analyses with outliers (identified using residuals) removed. Results with outliers retained are presented in the main text and results with outliers removed are presented in Supplementary Material 2 (Tables S4–S6; Table S10).

Our traditional meta-analyses evaluating the prevalence of MCI and dementia among TD and PIGD groups (Table 6) found larger group differences compared to our multilevel meta-analyses of continuous cognitive measures. Compared with TD patients, PIGD patients had a 29% greater relative risk of MCI and a 76% greater relative risk of dementia. These findings should be interpreted with caution, however, given the small number of included studies (*k* = 15 and *k* = 7, respectively) and moderate level of between-study heterogeneity (*I*^*2*^ = 50.9% and *I*^*2*^ = 42.4%, respectively). Of note, however, the pooled effect for our MCI model remained stable when outliers were removed, while the amount of between-study heterogeneity reduced by more than half (*I*^*2*^ = 24.1%).

**Table 6:** Results of meta-analyses comparing cognitive status in tremor-dominant and postural instability gait disorder (PIGD) motor subtype groups

Outcome	*k* (*o*)	Pooled risk ratio (95% CI)	*p*	*I*^*2*^ (%)	*Q* (*p*)	Egger’s test (*p*)
**Mild cognitive impairment**						
*All studies*	15 (4,471)	1.29 (1.07–1.56)	**.011**	50.9	28.50 (**.012**)	.305
*With outliers removed*	14 (4,372)	1.38 (1.19–1.6)	** <.001**	24.1	17.13 (.193)	.95
Dementia^*^						
*All studies*	7 (2,224)	1.76 (1.22–2.53)	**.009**	42.4	10.41 (.108)	-

*Note*. CI = confidence interval; *k* = number of studies/effect sizes;* o* = pooled sample size (both groups). For risk ratio, values > 1 indicate that postural instability gait disorder (PIGD) patients have a greater risk of mild cognitive impairment/dementia relative to tremor-dominant patients. Egger’s test was conducted only when *k* ≥ 10 using Pustejovsky and Rodgers’ (2019) method. ^*^ Results for model with outliers removed are not reported as no outliers were detected. Significant *p*-values (<.05) are highlighted in bold

##### Dose–effect analyses

Neither the SMD in UPDRS tremor subscore nor SMD in UPDRS PIGD subscore were significant moderators of differences in cognitive function between TD and PIGD subtypes (Supplementary Material 2, Table S10). This suggests that these motor subtype groups—derived from the *ratio* of tremor over PIGD subscores when using either Jankovic et al. (1990) or Stebbins et al.’s (2013) methods—are capturing distinct variation in cognitive function that is not captured when the severity of tremor or PIGD symptoms are considered independently of one another.

##### Moderator analyses

None of our moderator analyses (outliers retained) were statistically significant (Tables 7 and 8). Of note, subtyping method was not a significant moderator of the difference in cognitive performance between TD and PIGD patients; similar pooled effect sizes were observed for studies using Jankovic et al.’s (1990) subtyping procedure and studies using Stebbins et al.’s (2013) subtyping procedure. This suggests that these two subtyping approaches produce TD and PIGD motor subtype groups with comparable differences in cognitive performance. This is noteworthy given that, as reported (see Demographics and disease characteristics), we found that the magnitude of the difference in motor function between TD and PIGD groups was *not* similar between the UPDRS-III (used in Jankovic et al.’s (1990) subtyping method) and MDS-UPDRS-III (used in Stebbins et al.’s (2013) subtyping method).

**Table 7:** Results of continuous moderator analyses for tremor-dominant (TD) and postural instability gait disorder (PIGD) motor subtype groups (no outliers removed)

Moderator	*n*	*k*	$\beta$(95% CI)	*p*	Change in between-study *I*^*2*^
Sample size	50	252	0.00 (− 0.00, 0.00)	.215	1.51
Publication year	50	252	0.02 (− 0.01, 0.04)	.189	0.76
Pooled mean age	47	228	0.02 (− 0.02, 0.05)	.370	− 0.22
Pooled proportion of men	44	199	− 0.34 (− 1.8, 1.12)	.646	− 0.52
Pooled mean years of education	28	178	− 0.02 (− 0.06, − 0.01)	.209	− 0.59
Pooled mean disease duration	47	217	− 0.01 (− 0.05, 0.03)	.577	− 0.72
Pooled mean age at onset	48	231	0.01 (− 0.01, 0.04)	.277	0.21
Pooled mean LEDD	30	154	− 0.00 (− 0.00, 0.00)	.523	0.98
Pooled mean UPDRS-III (original)	26	132	0.00 (− 0.02, 0.02)	.745	− 1.34
Pooled mean MDS-UPDRS-III	20	98	− 0.00 (− 0.03, 0.02)	.683	− 1.74

*Note*. CI = confidence interval; *k* = number of effect sizes; LEDD = levodopa equivalent daily dose; MDS-UPDRS-III = Movement Disorder Society Unified Parkinson’s Disease Rating Scale Part III;* n* = number of unique studies; UPDRS-III = Unified Parkinson’s Disease Rating Scale Part III; $\beta$ = regression coefficient. For $\beta$, positive values reflect a positive association between the moderator and effect size (i.e., larger values of the moderator are associated with larger effect sizes, reflecting better cognitive performance in the tremor-dominant [TD] group relative to the postural instability gait disorder [PIGD] group). Change in *I*^*2*^ is the difference in between-study *I*^*2*^ for the model with and without the moderator (larger positive values correspond to greater between-study variance being accounted for by the moderator; negative values suggest that model fit worsened as a consequence of including the moderator). Significant *p*-values (<.05) are highlighted in bold. See Supplementary Material 2, Table S4 for results of analyses repeated with outliers removed (residuals approach)

**Table 8:** Results of categorical moderator analyses for tremor-dominant (TD) and postural instability gait disorder (PIGD) motor subtype groups (no outliers removed)

Moderator	*n*	*k*	Pooled Hedges’ *g* (95% CI)	$\beta$(95% CI)	*p*	*Q* (*p*)	ToM (*p*)
**Subtype method**						1522.87 (**<.001**)	0.54 (.582)
Jankovic^*^	26	116	0.25 (0.06, 0.44)		**.009**		
*Stebbins*	21	108	0.24 (0.1, 0.38)	− 0.12 (− 0.25, 0.22)	.918		
*Other*	3	28	0.48 (0.04, 0.91)	0.23 (− 0.24, 0.7)	.342		
**Cognitive class**						1520.07 (**<.001**)	1.36 (.245)
Global^*^	48	69	0.29 (0.15, 0.44)		** <.001**		
*Specific*	24	183	0.21 (0.1, 0.32)	− 0.08 (− 0.23, 0.09)	.245		
**Cognitive domain**						1484.11 (**<.001**)	0.38 (.991)
Global cognitive function^*^	48	69	0.28 (0.14, 0.42)		** <.001**		
*Executive function—attention*	6	11	0.47 (− 0.16, 0.78)	− 0.19 (− 0.15, 0.52)	.281		
*Executive function – cognitive flexibility*	11	15	0.24 (− 0.03, 0.51)	− 0.04 (− 0.3, 0.22)	.751		
*Executive function – cognitive inhibition*	6	6	0.16 (− 0.21, 0.53)	− 0.12 (− 0.49, 0.25)	.524		
*Executive function – response inhibition*	3	3	0.27 (− 0.29, 0.83)	− 0.01 (− 0.56, 0.54)	.962		
*Executive function – working memory*	16	25	0.15 (− 0.11, 0.41)	− 0.13 (− 0.37, 0.11)	.277		
*Higher-order fluid abilities—planning*	5	5	0.29 (− 0.11, 0.68)	0.01 (− 0.39, 0.4)	.979		
*Language*	12	19	0.21 (− 0.03, 0.46)	− 0.07 (− 0.31, 0.17)	.579		
*LTM/learning – lexical*	9	9	0.11 (− 0.17, 0.39)	− 0.17 (− 0.48, 0.14)	.286		
*LTM/learning—semantic*	8	8	0.13 (− 0.24, 0.51)	− 0.15 (− 0.51, 0.21)	.407		
*LTM/learning—visuospatial*	4	4	0.08 (− 0.34, 0.5)	− 0.20 (− 0.63, 0.22)	.346		
*STM – lexical*	9	9	0.17 (− 0.12, 0.49)	− 0.12 (− 0.43, 0.2)	.461		
*STM – numeric*	6	6	0.12 (− 0.3, 0.55)	− 0.16 (− 0.57, 0.25)	.442		
*STM – visuospatial*	5	10	0.19 (− 0.1, 0.49)	− 0.09 (− 0.39, 0.21)	.56		
*STM – other*	3	4	0.31 (− 0.14, 0.76)	− 0.03 (− 0.44, 0.49)	.906		
*Processing speed*	16	25	0.16 (− 0.06, 0.38)	− 0.12 (− 0.34, 0.09)	.265		
*Visuospatial abilities – construction*	3	4	0.10 (− 0.33, 0.52)	− 0.19 (− 0.62, 0.24)	.393		
*Visuospatial abilities – perception*	6	6	0.26 (− 0.15, 0.68)	− 0.02 (− 0.42, 0.39)	.928		
*Visuospatial abilities – reasoning*	3	4	0.33 (− 0.08, 0.74)	0.05 (− 0.38, 0.47)	.835		
**Medication status for cognitive assessment(s)**						442.71 (**<.001**)	1.02 (.387)
De novo^*^	8	53	0.22 (0.1, 0.34)				
*ON*	10	82	0.10 (− 0.144, 0.34)	− 0.12 (− 0.39, 0.15)	.367		
*OFF*	5	35	0.35 (0.18, 0.51)	0.13 (− 0.08, 0.33)	.228		
*ON or OFF*	1	2	0.19 (− 0.37, 0.75)	− 0.03 (− 0.6, 0.54)	.912		
**Medication status for motor assessment(s)**						1418.91 (**<.001**)	0.38 (.767)
De novo^*^	8	53	0.19 (0.05, 0.33)		**.008**		
*ON*	13	73	0.27 (0.09, 0.44)	0.07 (− 0.15, 0.3)	.516		
*OFF*	15	65	0.35 (0.02, 0.68)	0.16 (− 0.2, 0.52)	.389		
*ON or OFF*	4	8	0.16 (− 0.16, 0.48)	− 0.03 (− 0.38, 0.32)	.861		
**Cognitive Impairment/dementia as exclusion criterion**						1523.12 (**<.001**)	0.29 (.594)
No^*^	27	121	0.23 (0.14, 0.33)		** <.001**		
*Yes*	23	131	0.30 (0.07, 0.53)	0.07 (− 0.18, 0.31)	.594		
**Motor subtypes compared on cognition in paper**						1485.10 (**<.001**)	2.26 (.134)
No^*^	15	33	0.44 (0.11, 0.77)		**.009**		
*Yes*	42	206	0.19 (0.1, 0.28)	− 0.25 (− 0.58, 0.08)	.134		
**Overall risk of bias**						1517.14 (**<.001**)	0.812 (.445)
High^*^	20	81	0.37 (0.09, 0.65)		**.009**		
*Moderate*	19	99	0.17 (0.05, 0.30)	− 0.2 (− 0.5, 0.11)	.297		
*Low*	11	72	0.21 (0.08, 0.34)	− 0.16 (− 0.47, 0.14)	.204		

*Note*. CI = confidence interval; *k* = number of effect sizes; LTM = long-term memory; *n* = number of unique studies; STM = short-term memory; ToM = Test of Moderators; $\beta$ = regression coefficient. ^*^Reference category for model. The $\beta$ coefficients and corresponding *p*-values indicate whether the pooled effect size for that level of the moderator differs significantly from the pooled effect size of the reference category; for the reference category, these values indicate whether the pooled effect size differs significantly from zero. For Hedges’ *g*, values > 0 indicate poorer cognitive performance among postural instability gait disorder (PIGD) patients relative to tremor-dominant (TD) patients. Significant *p*-values (<.05) are highlighted in bold. See Supplementary Material 2, Table S5 for results of analyses repeated with outliers removed (residuals approach)

We also found no significant difference in the pooled effect size estimates for global measures versus domain-specific measures of cognition. Cognitive domain did not moderate effect size magnitude, likely owing to poor precision in the pooled effect size estimates for each cognitive domain; with the exception of global cognitive function, the 95% confidence intervals for all cognitive domains ranged from negative, negligible effect sizes to positive, moderate or large effect sizes. Our results did, however, suggest that the performance of TD and PIGD groups differs most strongly for measures of attention and visuospatial reasoning abilities. Patients with TD also showed an advantage on measures of cognitive flexibility and response inhibition. Taken together, these results indicate that cognitive differences between TD and PIGD subtypes may be particularly pronounced for measures of executive function. In contrast, negligible differences between TD and PIGD patients were observed for other visuospatial-based domains, including visuospatial long-term memory/learning, and visuospatial construction abilities. Given the limited number of effect sizes for each domain and the imprecision in pooled effect size estimates, further investigation is needed to corroborate these trends.

When we re-ran our moderator analyses with outliers removed, pooled mean age and pooled mean age at disease onset were statistically significant moderators of effect size magnitude (Supplementary Material 2, Table S4). Older mean age and later age at onset were each associated with a larger difference in cognitive performance between the TD and PIGD groups, favouring better cognition in the TD group. This may indicate that cognitive differences between TD and PIGD groups are more pronounced when patients in both subtype groups are older at the time of disease onset and/or older at the time of cognitive assessment. However, an older mean age (or age at onset) for the whole sample could also result from only *one* motor subtype group being older at the time of cognitive assessment (or time of onset), inflating the overall sample mean; our examination of the SMDs in age and age at onset between TD and PIGD groups as potential confounds allowed us to explore this possibility (see Confound analyses). Between-study *I*^*2*^ remained moderate for both moderators (33.72% for pooled mean age, 37.77% for pooled mean age at onset), indicating that the pooled mean age or age at onset could not fully explain the observed differences in cognition. None of our categorical moderators were statistically significant, even after outlier removal (Supplementary Material 2, Table S5).

##### Confound analyses

The results of our confound analyses (outliers retained) are presented in Table 9. Only the SMD in years of education and SMD in age at disease onset emerged as significant moderators. The pooled effect size for the difference in cognitive performance between TD and PIGD patients increased when the SMD in years of education (favouring *more* years of education in the TD group) increased (Supplementary Material 2, Fig. S1A). This is not unexpected, as more years of education among TD patients relative to PIGD patients would presumably confer further advantage to TD patients on tasks assessing cognition.

**Table 9:** Results of confound analyses for tremor-dominant (TD) and postural instability gait disorder (PIGD) motor subtype groups (no outliers removed)

Confound	*n*	*k*	$\beta$(95% CI)	*p*	Change in Between-Study *I*^*2*^
SMD age	47	228	− 0.19 (− 0.58, 0.2)	.332	0.03
Difference in proportion of men	44	199	− 0.42 (− 1.35, 0.51)	.376	− 0.32
SMD years of education	28	178	0.44 (0.09, 0.79)	**.014**	5.56
SMD disease duration	44	207	− 0.22 (− 0.65, 0.21)	.307	0.16
SMD age at onset	20	92	− 0.42 (− 0.69, − 0.16)	**.002**	2.46
SMD LEDD	30	154	− 0.35 (− 1.02, 0.33)	.311	0.55
SMD UPDRS-III (original)	26	132	− 0.24 (− 0.72, 0.25)	.334	1.02
SMD MDS-UPDRS-III	20	98	− 0.36 (− 0.81, 0.1)	.126	19.32
SMD UPDRS-III (any version)	46	230	− 0.24 (− 0.56, 0.07)	.128	1.95

*Note*. CI = confidence interval; *k* = number of effect sizes; LEDD = levodopa equivalent daily dose; MDS-UPDRS-III = Movement Disorder Society Unified Parkinson’s Disease Rating Scale Part III;* n* = number of unique studies; UPDRS-III = Unified Parkinson’s Disease Rating Scale Part III; SMD = standardised mean difference (Hedges’ *g*); $\beta$ = regression coefficient. For $\beta$, positive values reflect a positive association between the confound (moderator) and effect size (i.e., larger values of the confound [moderator] are associated with larger effect sizes, reflecting better cognitive performance in the tremor-dominant [TD] group relative to the postural instability gait disorder [PIGD] group). Change in *I*^*2*^ is the difference in between-study *I*^*2*^ for the model with and without the confound (moderator; larger positive values correspond to greater between-study variance being accounted for by the confound [moderator]; negative values suggest that model fit worsened as a consequence of including the confound [moderator]). Significant *p*-values (<.05) are highlighted in bold. See Supplementary Material 2, Table S6 for results of analyses repeated with outliers removed (residuals approach)

For age at disease onset, the pooled effect size for the difference in cognitive performance between TD and PIGD patients *decreased* when the SMD in age at onset increased above zero, reflecting *older* age at onset in the TD group. In other words, in studies where TD patients were older at the time of disease onset relative to PIGD patients, these TD patients showed a *less* marked cognitive advantage over their PIGD counterparts. When the SMD in age at onset increased *below* zero, reflecting older age at onset in the PIGD group, the pooled effect size for the difference in cognitive performance *increased*, favouring better cognition in the TD group (Supplementary Material 2, Fig. S2A). Consistent with previous research that has reported greater motor and nonmotor dysfunction among PD patients who are older at the time of disease onset (Pagano et al., 2016; Virameteekul et al., 2021), this result supports a general relationship between older age at onset and poorer cognition in PD, irrespective of motor subtype.

When we re-ran our confound analyses with outliers removed, both the SMD in years of education and the SMD in age at onset remained statistically significant (Supplementary Material 2, Table S6; Figs. S1B and S2B). In addition, both the SMDs in age and UPDRS-III score (original version) emerged as significant confounds. The effect of both confounds on the difference in cognition between TD and PIGD motor subtypes followed the same pattern as observed for the SMD in age at disease onset. Where the SMD in age increased above zero, reflecting older age among TD patients relative to PIGD patients, the pooled effect size estimate for the difference in cognitive performance between TD and PIGD subtypes *decreased*; and where the SMD in age decreased below zero, reflecting older age among PIGD patients relative to TD patients, the pooled effect size for cognition *increased*, reflecting a larger difference in cognitive performance that favoured the TD subtype (Supplementary Material 2, Fig. S3). For UPDRS-III scores (original version), greater motor impairment in the PIGD group relative to the TD group was associated with a larger difference in cognition that favoured the TD group. Greater motor impairment in the TD group relative to the PIGD group was associated with a pooled effect size for cognition that approached—and in some cases exceeded—zero (Supplementary Material 2, Fig. S4). Again, this finding is consistent with a general association between motor symptom severity and cognitive decline, irrespective of motor subtype. Between-study *I*^*2*^ did not reach zero for any of our significant confounds (27.5–31.12% for SMD in years of education; 1.56–42.48% for SMD in age at onset; 20.55% for SMD in age) but did near zero for the SMD in UPDRS-III score (original version; < 0.00%).

### Tremor-dominant vs. indeterminate and postural instability gait disorder vs. indeterminate

Results of our analyses comparing the indeterminate (ID) motor subtype group to both the tremor-dominant (TD) and postural instability gait disorder (PIGD) motor subtype groups can be found in Supplementary Material 2 (Tables S7–S19; Figs. S5–S10).

Our multilevel meta-analysis comparing TD and ID groups found a negligible-to-small difference in cognitive performance, favouring TD patients (*n* (*k*) = 19 (72), *g* = 0.15 [95% CI 0.08–0.22], *p* <.001). Although neither of our traditional meta-analyses were statistically significant, both MCI (*k* (*o*) = 9 (2301), *RR* = 1.18 [95% CI 0.97–1.44], *p* =.082) and dementia (*k* (*o*) = 6 (1383), *RR* = 1.41 [95% CI 0.84–2.39], *p* =.149) were more common in ID patients than TD patients.

Our multilevel meta-analysis comparing PIGD and ID subtypes revealed a negligible-to-small difference in cognitive performance that favoured ID patients (*n* (*k*) = 19 (72), *g* = 0.14 [95% CI 0.06–0.21], *p* =.005). Again, neither of our traditional meta-analyses reached statistical significance; however, consistent with our multilevel meta-analysis, both showed trends of MCI (*k* (*o*) = 9 (2642), *RR* = 1.15 [95% CI 0.92–1.43], *p* =.195) and dementia (*k* (*o*) = 6 (1403), *RR* = 1.53 [95% CI 0.94–2.48], *p* =.073) being more common among PIGD patients relative to ID patients. No moderators or confounds were statistically significant for either comparison (TD vs. ID, PIGD vs. ID).

### Other tremor and postural instability subtypes

#### Predominantly tremor-dominant vs. predominantly postural instability gait disorder

Two studies (Herman et al., 2015; Ren et al., 2020) compared predominantly tremor-dominant (p-TD) and predominantly postural instability gait disorder (p-PIGD) motor subtype groups on continuous measures of cognition. A narrative synthesis of the results of these studies can be found in Supplementary Material 2. Overall, both studies found evidence for better preserved cognition among p-TD patients relative to p-PIGD patients, consistent with the results of our TD vs. PIGD comparison.

#### Tremor-dominant vs. nontremor-dominant

Ten studies compared the cognitive performance of TD and nontremor-dominant (NTD) motor subtype groups; of these, all studies reported continuous cognitive outcome data, and one study compared rates of MCI. There was marked variation in the subtype methods used to classify patients into these groups. The most common method used (*n* = 6) involved applying either Jankovic et al. (1990) or Stebbins et al.’s (2013) method for subtyping participants as TD, PIGD, or ID, and then collapsing the PIGD and ID groups to form a NTD group. The remaining studies all used distinct subtyping methods, often based on clinical judgement (e.g., Si et al., 2018) or cutoff scores on one or more tremor items of the UPDRS or MDS-UPDRS (e.g., Stegemöller et al., 2013; van Nuland et al., 2020).

Traditional meta-analyses revealed negligible-to-small pooled effect size estimates for most demographics and disease characteristics (Supplementary Material 2, Table S20); with the exception of age, LEDD, and UPDRS total score (any version), these effects were not statistically significant. Relative to TD patients, there was a slight tendency for NTD patients to be older (*k* (*o*) = 10 (2655), *g* = 0.12 [0.01–0.22], *p* =.034), and NTD patients reported a significantly higher LEDD compared to TD patients (*k* (*o*) = 5 (1132), *g* = 0.31 [0.13–0.5], *p* =.009). When TD and NTD were compared on overall motor severity, a small, significant pooled effect size was found (*k* (*o*) = 8 (2575), *g* = 0.23 [0.01–0.44], *p* =.041), indicating greater motor symptom severity in the NTD group. It should be noted, however, that this effect only reached statistical significance when data from the UPDRS-III (original version) and MDS-UPDRS were combined, and following the removal of one outlier.

The results of our multilevel meta-analyses evaluating cognitive differences between TD and NTD subtype groups are presented in Table 10. While both of our models (with and without outliers removed) indicated a statistically significant difference between subtypes, in both cases the magnitude of the differences was negligible. Multilevel Egger’s tests for both models were nonsignificant, suggesting no evidence of publication bias. Consistent with these results, our analysis of cognitive status data (*n* = 1) from the PPMI did not reveal a marked difference in the rate of MCI among TD (18.3%) and NTD (14.9%) groups; however, it is interesting to note that the rate of MCI was slightly greater in the TD group.

**Table 10:** Results of multilevel meta-analyses of cognitive performance in other tremor and postural instability motor subtype pairs

Subtype pair	Model	*n* (*k*)	Pooled Hedges’ *g* (95% CI)	*p*	Within-study *I*^*2*^ (%) (*p*)	Between-study *I*^*2*^ (%) (*p*)	*Q* (*p*)	Multilevel Egger’s test (*p*)
TD vs. NTD^*^	All studies	10 (25)	0.08 (0.00, 0.15)	**.039**	2.29 (.5)	0 (.5)	32.42 (.117)	2.73 (.112)
	With outliers removed – residuals approach	9 (23)	0.08 (0.01, 0.16)	**.025**	1.84 (.5)	5.22 (.5)	20.62 (.544)	0.06 (.816)
Non-PIGD vs. PIGD^†^	All studies	7 (31)	0.17 (− 0.16, 0.51)	.297	2.68 (**.031**)	89.22 (**<.001**)	64.13 (**<.001**)	0.03 (.853)
	With outliers removed – residuals approach	6 (27)	0.27 (0.07, 0.47)	**.01**	10.03 (**.031**)	62.31 (**.027**)	42.14 (**.024**)	0.18 (.679)
	With outliers removed – Cook’s distance approach	4 (28)	0.21 (− 0.06, 0.48)	.120	7.46 (**.031**)	70.22 (**.049**)	42.58 (**.029**)	0.03 (.867)

*Note*. CI = confidence interval; FOG = freezing of gait; *k* = number of effect sizes;* n* = number of unique studies; nFOG = no freezing of gait; NTD = nontremor-dominant; non-PIGD = nonpostural instability gait disorder; PIGD = postural instability gait disorder; TD = tremor-dominant. ^*^For Hedges’ *g*, values > 0 indicate poorer cognitive performance among nontremor-dominant (NTD) patients relative to tremor-dominant (TD) patients. ^†^For Hedges’ *g*, values > 0 indicate poorer cognitive performance among postural instability gait disorder (PIGD) patients relative to nonpostural instability gait disorder (non-PIGD) patients. No meta-analysis conducted for TD vs. NTD comparison with outliers removed using Cook’s distance approach as Cook’s distance was computed as NA for all effect sizes (likely due to low between-study variance). Significant *p*-values (<.05) are highlighted in bold

No dose–effect analyses and only a subset of moderator and confound analyses were performed due to insufficient data (*n* < 10 studies); none of the moderators or confounds with sufficient data for analysis were statistically significant (Supplementary Material 2, Tables S21–S23).

#### Postural instability gait disorder vs. nonpostural instability gait disorder

Eight studies compared PIGD and non-PIGD motor subtype groups on cognitive outcomes; of these, continuous cognitive data were available for seven studies and cognitive status data were available for three studies. In all studies, the PIGD and non-PIGD motor subtype groups were produced by applying either Jankovic et al. (1990) or Stebbins et al.’s (2013) method for classifying participants as TD, PIGD, or ID, and then collapsing the TD and ID groups to form a non-PIGD group.^11^

Traditional meta-analyses revealed that, compared to non-PIGD patients, PIGD patients reported longer disease duration (*k* (*o*) = 7 (1,750), *g* = 0.29 [0.06–0.51], *p* =.021) and had greater overall motor symptom severity (*k* (*o*) = 6 (1,686), *g* = 0.22 [0.04–0.4], *p* =.027; see Supplementary Material 2, Table S24). The latter effect was only statistically significant when data from the UPDRS (original version; *k* = 4) and MDS-UPDRS (*k* = 2) were collapsed; when these assessment tools were considered separately, no significant group differences emerged. Given that all pooled effect size estimates had wide confidence intervals, likely owing to the small number of included studies, these results should be interpreted cautiously.

Results of our multilevel meta-analyses examining differences in cognitive performance are presented in Table 10. Our full model was not statistically significant. There was little consistency in the effect sizes reported both within- and between-studies, as indicated by the significant within- and between-study heterogeneity statistics and wide 95% confidence interval, which included zero and ranged from a negligible to moderate effect size. Outlier removal using a residuals approach (resulting in the exclusion of one study contributing four effect sizes) produced a small, significant, pooled effect reflecting marginally better cognition in the non-PIGD group relative to the PIGD group. We have limited confidence in this estimate, however, given the small number of included studies, wide confidence interval that nears zero, and statistically significant heterogeneity statistics. Multilevel Egger’s tests for all three models suggested no evidence of publication bias. No dose–effect, moderator, or confound analyses were conducted as *n* < 10 studies.

With regards to cognitive status, two studies (PPMI as one study; Shkodina et al., 2022) compared rates of MCI (relative to NC) among PIGD and non-PIGD patients and one study (Pelicioni et al., 2021) compared rates of mild and marked executive dysfunction (relative to normal executive function); no studies compared rates of dementia. No clear trends were apparent when data were synthesised across studies. In Shkodina et al. (2022), rates of MCI were high in both motor subtype groups (PIGD = 63.2%; non-PIGD = 53.8%); PIGD patients had a slightly higher risk of impairment. In comparison, our analysis of the PPMI dataset revealed a much smaller difference in MCI rates between groups (PIGD = 15.0%; non-PIGD = 17.8%), which indicated marginally *lower* rates of MCI among PIGD patients; MCI was much less prevalent in both subtype groups within the PPMI cohort, however, presumably owing to this de novo sample having a shorter disease duration. When subtype groups were specifically compared on executive function impairments, more substantial differences emerged. Patients belonging to the PIGD subtype experienced both mild executive dysfunction (62.3%) and marked executive dysfunction (49.5%) at much higher rates than non-PIGD patients (36.9% mild executive dysfunction; 21.2% marked executive dysfunction). These results should be interpreted cautiously, given that they come from a single study in which the presence of executive dysfunction was determined only using cutoff scores on the FAB.

### Tremor-dominant vs. akinetic-rigid

#### Demographics and disease characteristics

The results of traditional meta-analyses comparing tremor-dominant (TD) and akinetic-rigid (AR) motor subtype groups on demographics and disease characteristics are reported in Supplementary Material 2, Table S25. Only UPDRS-III score (original version; Fahn et al., 1987) was found to differ significantly between subtype groups, with AR participants having greater overall motor impairment compared to TD participants (*k* (*o*) = 17 (2,396), *g* = 0.29 [0.2–0.37], *p* <.001). This small pooled effect increased to a large pooled effect when data from both UPDRS-III versions were analysed together (*k* (*o*) = 19 (3,321), *g* = 0.89 [0.11–1.9], *p* =.078), but this estimate was imprecise—as indicated by the wide 95% confidence interval—and both MDS-UPDRS-III effect sizes were identified as outliers^12^; unsurprisingly, this pooled effect was not statistically significant. Following the removal of one outlier, a small pooled effect was also observed for LEDD, indicating higher LEDD in AR patients relative to TD patients (*k* (*o*) = 11 (1,044), *g* = 0.24 [0.01–0.47], *p* =.058); again, however, this estimate lacked precision and should be interpreted with caution.

#### Cognition

##### Main analyses

**Fig. 3:**
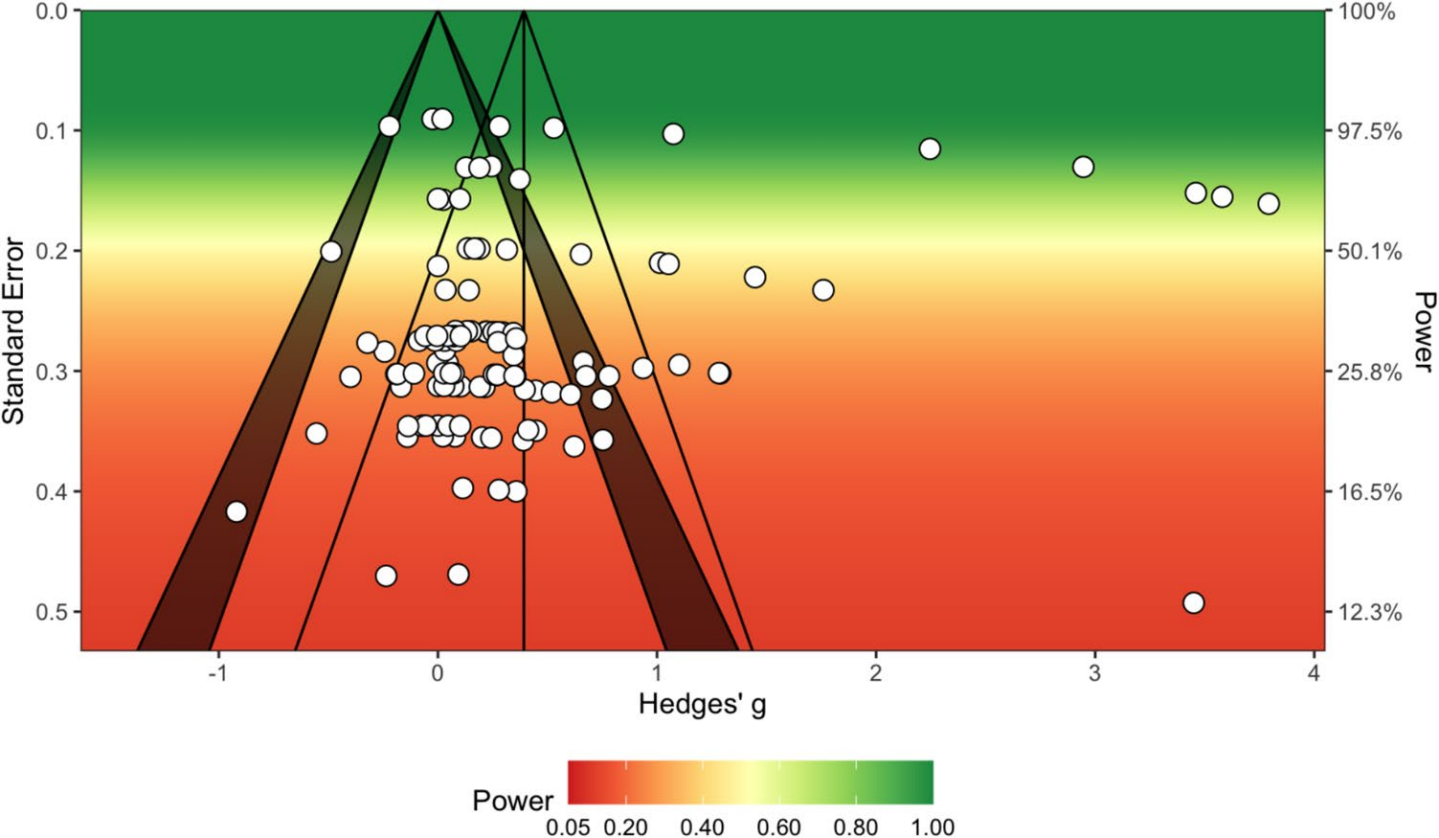
Sunset (power-enhanced) funnel plot for difference in cognitive performance between tremor-dominant (TD) and akinetic-rigid (AR) motor subtype groups (no outliers removed)

As shown in Table 11, our multilevel meta-analysis of 116 effect sizes from 20 studies revealed a small-to-moderate difference in cognitive performance between TD and AR participants, favouring the TD group. Within-study heterogeneity was high (within-study *I*^*2*^ = 72.22%)—possibly due to variation in the cognitive domains assessed by tasks administered within each study—and multilevel Egger’s test suggested the presence of publication bias. Inspection of a sunset (power-enhanced) funnel plot (Fig. 3) indicated several remarkably large effect sizes reflecting better cognitive performance in TD patients relative to AR patients. These effect sizes were identified as outliers using both approaches (residuals and Cook’s distance) and, consequently, both of our models with outliers removed returned nonsignificant multilevel Egger’s tests (Table 11). Although outlier removal resulted in a slight decrease in the pooled effect size estimates for both models, these estimates remained statistically significant.

**Table 11:** Results of multilevel meta-analyses of cognitive performance in tremor-dominant (TD) and akinetic-rigid (AR) motor subtype groups

Subtype Pair	Model	*n* (*k*)	Pooled Hedges’ *g* (95% CI)	*p*	Within-study *I*^*2*^ (%) (*p*)	Between-study *I*^*2*^ (%) (*p*)	*Q* (*p*)	Multilevel Egger’s test (*p*)
TD vs. AR	All studies	20 (116)	0.36 (0.13, 0.59)	**.003**	72.22 (**<.001**)	20.57 (**.002**)	2,590.75 (**<.001**)	** <.001**
	With outliers removed – residuals approach	20 (108)	0.21 (0.12, 0.31)	** <.001**	62.65 (**<.001**)	1.06 (.5)	362.00 (**<.001**)	.825
	With outliers removed – Cook’s distance approach	17 (99)	0.24 (0.14, 0.34)	** <.001**	56.38 (**<.001**)	5.42 (.5)	312.37 (**<.001**)	.525

*Note*. AR = akinetic-rigid; CI = confidence interval; *k* = number of effect sizes; *n* = number of unique studies; TD = tremor-dominant. For Hedges’ *g*, values > 0 indicate poorer cognitive performance among akinetic-rigid (AR) patients relative to tremor-dominant (TD) patients. Significant *p*-values (<.05) are highlighted in bold

Given the presence of publication bias in our full model, we ran our dose–effect, moderator, and confound analyses with all available effect sizes and then repeated these analyses with outliers (identified using the residuals approach) removed. Results with outliers retained are presented in the main text and results with outliers removed are presented in Supplementary Material 2 (Tables S26–29).

With regards to cognitive status, two studies (Karunanayaka et al., 2016; Zhang et al., 2016) compared MCI rates between TD and AR patients and one study (Paulus & Jellinger, 1991) compared dementia rates between TD and AR patients. Although both Karunanayaka et al. (2016) and Zhang et al. (2016) reported higher rates of MCI among AR patients relative to TD patients, the overall rate of MCI within each sample was considerably different. In Karunanayaka et al. (2016), 23.5% of AR patients and no TD patients were classified as having MCI. Within the sample recruited by Zhang et al. (2016), MCI was much more common, and its prevalence much more homogenous between motor subtype groups (TD = 50.0%, AR = 57.4%). While the low rates of MCI reported by Karunanayaka and colleagues (2016) could possibly be attributed to the disease characteristics of their sample—participants were relatively young at time of disease onset (*M* = 56.7 years [SD not reported]) and reported a relatively short disease duration (*M* = 3.6, *SD* = 4.1 years)—similar disease characteristics (age at disease onset: *M* = 56.8, *SD* = 11 years; disease duration: *M* = 4.8, *SD* = 4.2 years) were also reported for Zhang et al.’s (2016) sample.^13^

Somewhat incongruous with these findings regarding greater rates of MCI in the AR subtype, Paulus and Jellinger (1991) reported a higher prevalence of dementia in TD patients (76.5%) relative to AR patients (61.9%; *RR* = 1.235). This finding stands in stark contrast to other subtype comparisons including a TD subtype, where this subtype was associated with *lower* dementia prevalence (e.g., Table 6; Supplementary Material 2, Table S9). It is also noteworthy with respect to the high prevalence of dementia observed across the whole sample relative to other studies, which is likely partly attributable to the sample being much older on average (*M* = 77.2, *SD* = 7.2 years) than those of other studies.

##### Dose–effect analyses

Our dose–effect analysis indicated that the SMD in UPDRS tremor subscore was not a significant moderator of differences in cognitive function between TD and AR subtypes ($\beta$ = − 0.06, *p* =.782; Supplementary Material 2, Table S26). As with our TD vs. PIGD comparison, this nonsignificant result suggests that the TD and AR subtype classification—derived from the ratio of tremor to akinetic-rigid subscores—is capturing some aspect of motor symptom profile not captured when the severity of tremor symptoms is considered alone. Insufficient data (*n* < 10) were available to run a dose–effect analysis on the SMD in UPDRS AR subscore.

##### Moderator analyses

Results of our moderator analyses (outliers retained) are presented in Tables 12 and 13. Only sample size was a significant moderator; as study sample size increased, the difference in cognitive function between the TD and AR subtypes increased, favouring TD patients. However, this effect was driven by several large effect size outliers (Supplementary Material 2, Fig. S11), and sample size was no longer a significant moderator when we repeated this analysis with outliers removed (Supplementary Material 2, Table S27).

**Table 12:** Results of continuous moderator analyses for tremor-dominant (TD) and akinetic-rigid (AR) motor subtype groups (no outliers removed)

Moderator	*n*	*k*	$\beta$(95% CI)	*p*	Change in between-study *I*^*2*^
Sample size	20	116	0.00 (0.00, 0.00)	**.02**	8.93
Publication year	20	116	0.00 (− 0.06, 0.06)	1.000	− 1.54
Pooled mean age	18	104	− 0.01 (− 0.07, 0.05)	.757	− 1.01
Pooled proportion of men	15	77	0.8 (− 2.27, 3.87)	.605	− 1.72
Pooled mean years of education	15	91	− 0.01 (−0.13, 0.11)	.819	− 1.39
Pooled mean disease duration	18	104	0.02 (−0.01, 0.06)	.215	0.00
Pooled mean age at onset	18	104	− 0.00 (− 0.03, 0.02)	.860	− 0.09
Pooled mean LEDD	12	79	0.00 (− 0.00, 0.00)	.476	− 1.77
Pooled mean UPDRS-III (original)	17	94	− 0.00 (− 0.02, 0.01)	.804	0.00

*Note*. CI = confidence interval; *k* = number of effect sizes;* n* = number of unique studies; UPDRS-III = Unified Parkinson’s Disease Rating Scale Part III; $\beta$ = regression coefficient. For $\beta$, positive values reflect a positive association between the moderator and effect size (i.e., larger values of the moderator are associated with larger effect sizes, reflecting better cognitive performance in the tremor-dominant [TD] group relative to the akinetic-rigid [AR] group). Change in *I*^*2*^ is the difference in between-study *I*^*2*^ for the model with and without the moderator (larger positive values correspond to greater between-study variance being accounted for by the moderator; negative values suggest that model fit worsened as a consequence of including the moderator). No moderator analyses performed for pooled mean Movement Disorder Society (MDS)-UPDRS Part III score as *n* < 10 studies. Significant *p*-values (<.05) are highlighted in bold. See Supplementary Material 2, Table S27 for results of analyses repeated with outliers removed (residuals approach)

**Table 13:** Results of categorical moderator analyses for tremor-dominant (TD) and akinetic-rigid (AR) motor subtype groups (no outliers removed)

Moderator	*n*	*k*	Pooled Hedges’ *g* (95% CI)	$\beta$(95% CI)	*p*	*Q* (*p*)	ToM (*p*)
**Subtype method**						2,581.59 (**<.001**)	1.62 (.206)
Kang^*^	11	65	0.2 (− 0.01, 0.41)		.058		
*Other*	9	51	0.51 (0.08, 0.94)	0.31 (− 0.17, 0.78)	.206		
**Cognitive class**						2,511.64 (**<.001**)	0.383 (.537)
Global^*^	20	32	0.32 (0.08, 0.55)		**.01**		
*Specific*	12	84	0.46 (0.07, 0.85)	0.14 (− 0.31, 0.6)	.537		
**Cognitive domain**						1,509.82 (**<.001**)	0.762 (.706)
Global cognitive function^*^	20	32	0.3 (0.1, 0.51)		**.004**		
*Executive function – cognitive flexibility*	4	4	0.28 (− 0.31, 0.86)	− 0.03 (− 0.64, 0.59)	.926		
*Executive function – cognitive inhibition*	6	6	0.84 (− 0.28, 1.95)	0.53 (− 0.58, 1.64)	.344		
*Executive function – working memory*	7	10	0.52 (0.14, 0.91)	0.22 (− 0.21, 0.64)	.316		
*Composite IQ*	2	2	0.32 (− 0.5, 1.15)	0.02 (−0.83, 0.87)	.963		
*Language*	4	4	1.1 (− 0.06, 2.26)	0.8 (− 0.36, 1.95)	.173		
*LTM – lexical*	2	2	0.1 (− 0.76, 0.96)	− 0.21 (− 1.08, 0.67)	.643		
*LTM – semantic*	3	3	0.97 (− 0.43, 2.37)	0.66 (− 0.73, 2.05)	.345		
*LTM – visuospatial*	4	5	0.36 (− 0.16, 0.89)	0.06 (− 0.5, 0.62)	.832		
*STM – lexical*	3	3	0 (− 0.67, 0.68)	− 0.3 (− 1, 0.4)	.391		
*STM – numerical*	2	2	− 0.34 (− 1.12, 0.45)	− 0.64 (− 1.45, 0.16)	.117		
*STM – visuospatial*	4	7	0.08 (− 0.39, 0.55)	− 0.22 (− 0.72, 0.28)	.383		
*Processing speed*	7	14	0.15 (− 0.2, 0.5)	− 0.16 (− 0.54, 0.23)	.421		
*Visuospatial abilities – construction*	2	2	0.19 (− 0.63, 1)	− 0.12 (− 0.95, 0.71)	.777		
*Visuospatial abilities – perception*	2	2	0.55 (− 0.3, 1.39)	0.24 (− 0.62, 1.1)	.579		
**Medication status for cognitive assessment(s)**						1,661.74 (**<.001**)	0.578 (.631)
De novo^*^	4	20	0.59 (− 0.26, 1.44)		.170		
*ON*	5	40	0.31 (0.03, 0.58)	− 0.28 (− 1.17, 0.61)	.530		
*OFF*	4	18	0.06 (− 0.35, 0.46)	− 0.53 (− 1.47, 0.41)	.262		
*ON or OFF*	3	17	0.29 (− 0.12, 0.69)	− 0.31 (− 1.25, 0.63)	.520		
**Medication status for motor assessment(s)**						2,574.04 (**<.001**)	0.246 (.864)
De novo^*^	4	20	0.6 (− 0.26, 1.45)		.169		
*ON*	4	30	0.37 (0.05, 0.69)	− 0.23 (− 1.14, 0.69)	.625		
*OFF*	7	40	0.24 (− 0.1, 0.58)	− 0.35 (− 1.28, 0.57)	.447		
*ON or OFF*	3	17	0.29 (− 0.14, 0.71)	− 0.31 (− 1.26, 0.64)	.520		
**Cognitive impairment/dementia as exclusion criterion**						2,583.54 (**<.001**)	2.32 (.131)
No^*^	10	42	0.54 (0.13, 0.94)		**.011**		
*Yes*	10	74	0.19 (− 0.02, 0.39)	− 0.35 (− 0.81, 0.11)	.131		
**Motor subtypes compared on cognition in paper**						2,581.59 (**<.001**)	0.51 (.476)
No^*^	3	13	0.53 (0.07, 0.99)		**.025**		
*Yes*	19	103	0.34 (0.08, 0.59)	− 0.19 (− 0.73, 0.34)	.476		
**Overall risk of bias**						2,586.55 (**<.001**)	0.5 (.606)
High^*^	9	44	0.39 (0.11, 0.67)		**.007**		
*Moderate*	6	30	0.46 (− 0.16, 1.08)	0.07 (− 0.62, 0.75)	.848		
*Low*	5	42	0.21 (− 0.06, 0.49)	− 0.18 (− 0.57, 0.22)	.379		

*Note*. CI = confidence interval; IQ = intelligence quotient; *k* = number of effect sizes; LTM = long-term memory;* n* = number of unique studies; STM = short-term memory; ToM = Test of Moderators; $\beta$ = regression coefficient. ^*^Reference category for model. The $\beta$ coefficients and corresponding *p*-values indicate whether the pooled effect size for that level of the moderator differs significantly from the pooled effect size of the reference category; for the reference category, these values indicate whether the pooled effect size differs significantly from zero. For Hedges’ *g*, values > 0 indicate poorer cognitive performance among akinetic-rigid (AR) patients relative to tremor-dominant (TD) patients. Significant *p*-values (<.05) are highlighted in bold. See Supplementary Material 2, Table S28 for results of analyses repeated with outliers removed (residuals approach)

Although cognitive domain remained a nonsignificant moderator when our analyses were repeated with outliers removed (Supplementary Material 2, Table S28), the pooled effect sizes for two cognitive domains emerged as significantly different from the pooled effect size for global cognitive function when outliers were removed. The pooled effect size for semantic long-term memory (*g* = 0.86 [95% CI 0.38–1.33]) was significantly larger than the pooled effect size for global cognitive function (*g* = 0.16 [95% CI 0.04–0.28], *p* =.005), suggesting that TD patients show a marked advantage over AR patients in this domain. The absolute pooled effect size for numerical short-term memory (*g* = − 0.38 [95% CI − 0.8–0.05]) was also significantly larger than that for global cognitive function (*p* =.018) but reflected superior performance in the AR group relative to the TD group. This was the only cognitive domain where AR patients showed an advantage over TD patients. These results should be interpreted with caution, however, given the small number of effect sizes available for analysis (*k* = 2 for both semantic long-term memory and numerical short-term memory).

##### Confound analyses

Results of our confound analyses (outliers retained) are presented in Table 14. Only SMD in UPDRS-III score (any version) was a significant confound of the difference in cognitive performance between TD and AR patients; as the SMD in UPDRS-III score (any version) increased below zero, reflecting greater motor impairment in the AR group relative to the TD group, the difference in cognition between subtypes increased, favouring TD patients (Supplementary Material 2, Fig. S12). Given that the SMD in UPDRS-III score (original version) was not statistically significant, it is likely that this significant result was driven by the inclusion of MDS-UPDRS-III data, taken from two papers authored by the same research group (Moretti et al., 2017, 2020). The SMD in MDS-UPDRS-III scores were, in both instances, unreasonably large; as such, this result is likely spurious.^14^

**Table 14:** Results of confound analyses for tremor-dominant (TD) and akinetic-rigid (AR) motor subtype groups (no outliers removed)

Confound	*n*	*k*	$\beta$(95% CI)	*p*	Change in between-study *I*^*2*^
SMD age	18	104	0.23 (− 0.18, 0.64)	.264	0.23
Difference in proportion of men	15	77	− 0.34 (− 2.3, 1.62)	.729	− 1.84
SMD years of education	15	91	0.27 (− 0.7, 1.23)	.58	− 1.36
SMD disease duration	17	94	− 0.18 (− 0.48, 0.13)	.251	0.00
SMD LEDD	12	79	− 0.19 (− 0.48, 0.11)	.212	0.93
SMD UPDRS-III (original)	17	94	0.13 (− 0.39, 0.64)	.63	0.00
SMD UPDRS-III (any version)	19	106	− 0.14 (− 0.23, − 0.05)	**.003**	10.25

*Note*. CI = confidence interval; *k* = number of effect sizes; LEDD = levodopa equivalent daily dose;* n* = number of unique studies; UPDRS-III = Unified Parkinson’s Disease Rating Scale Part III; SMD = standardised mean difference (Hedges’ *g*); $\beta$ = regression coefficient. For $\beta$, positive values reflect a positive association between the confound (moderator) and effect size (i.e., larger values of the confound [moderator] are associated with larger effect sizes, reflecting better cognitive performance in the tremor-dominant [TD] group relative to the akinetic-rigid [AR] group). Change in *I*^*2*^ is the difference in between-study *I*^*2*^ for the model with and without the confound (moderator; larger positive values correspond to greater between-study variance being accounted for by the confound [moderator]; negative values suggest that model fit worsened as a consequence of including the confound [moderator]). No confound (moderator) analyses performed for SMD in age at disease onset or SMD in Movement Disorder Society (MDS)-UPDRS Part III scores as *n* < 10 studies. Significant *p*-values (<.05) are highlighted in bold. See Supplementary Material 2, Table S29 for results of analyses repeated with outliers removed (residuals approach)

When we repeated our confound analyses with outliers removed (Supplementary Material 2, Table S29), none of our confounds were statistically significant, but the SMD in years of education neared significance (*p* =.069). As the difference in years of education between subtypes increased, reflecting more years of education in TD patients relative to AR patients, TD patients’ cognitive advantage increased; as the difference in years of education increased in the opposite direction, reflecting more years of education in AR patients relative to TD patients, TD patients’ cognitive advantage *decreased*. This pattern is broadly consistent with that reported for our TD vs. PIGD comparison and aligns with more years of education being associated with better performance on tasks assessing cognition (Guerra-Carrillo et al., 2017; Schneeweis et al., 2014).

### Tremor-dominant vs. mixed and akinetic-rigid vs. mixed

Results of our analyses comparing the mixed (MX) motor subtype group to both the tremor-dominant (TD) and akinetic-rigid (AR) motor subtype groups can be found in Supplementary Material 2 (Tables S30–S33, Figs. S13–S14). Our multilevel meta-analyses revealed no significant differences in cognitive function between the TD and MX subtypes (*n* (*k*) = 5 (17), *g* = 0.09 [95% CI − 0.14–0.31], *p* =.419) or between the AR and MX subtypes (*n* (*k*) = 5 (17), *g* = 0.04 [95% CI − 0.05–0.13], *p* =.33). Consistent with these results, the one study (Zhang et al., 2016) that compared rates of MCI between subtypes reported comparable rates of MCI among TD and MX patients, and between AR and MX patients. No dose–effect, moderator, or confound analyses were performed due to an insufficient number of studies (*n* < 10).

### Other tremor and bradykinesia subtypes

#### Tremor vs. bradykinesia

Five studies compared the cognitive performance of a tremor (TR) motor subtype to that of a bradykinesia (BR) motor subtype. Results of our analyses comparing the TR and BR motor subtypes can be found in Supplementary Material 2 (Tables S34–S35; Fig. S15).

#### Bradykinesia vs. no-bradykinesia

One study (Tomer et al., 2002) compared the cognitive performance of PD patients with and without bradykinesia on a single task, the Wisconsin Card Sorting Test (WCST). The results of this study are summarised in Supplementary Material 2.

### Other Tremor-only subtypes

Two studies (Poletti et al., 2012; Rana et al., 2012) formed motor subtype groups based on the presence (TR-Y) or absence (TR-N) of tremor, and one study (Ou et al., 2021) subtyped participants according to the presence or absence of facial tremor. A narrative synthesis of the results of these studies can be found in Supplementary Material 2.

### Freezing of Gait vs. Nonfreezing of Gait

Twenty included studies classified patients into freezing of gait (FOG) and nonfreezing of gait (nFOG) motor subtype groups. In the majority of cases (*k* = 14; e.g., Sunwoo et al., 2013; Yu et al., 2022), this classification was performed by first asking patients to self-report FOG, typically using the Freezing of Gait Questionnaire (FOGQ; Giladi et al., 2000) or New Freezing of Gait Questionnaire (nFOGQ; Nieuwboer et al., 2009); following this, the presence of FOG was confirmed via direct observation by a neurologist, movement disorder specialist, or researcher. In some but not all studies, observation of FOG took place whilst participants performed specific tasks known to have a high probability of eliciting FOG episodes (e.g., walking through a narrow doorway). In the remaining studies (*k* = 6), patient classification into FOG and nFOG subtypes was based on clinical observation alone. In three of these studies (Nantel et al., 2012; Pietracupa et al., 2018; Sawada et al., 2019), observation of FOG took place whilst participants performed specific actions known to provoke FOG; in two studies (Bohnen et al., 2014; Pongmala et al., 2023), clinical observation was formally scored using item 3.11 of the MDS-UPDRS (Goetz et al., 2008); and one study (Lord et al., 2020) provided no further detail regarding the conditions under which FOG was observed.

#### Demographics and disease characteristics

Results of our traditional meta-analyses comparing demographics and disease characteristics of FOG and nFOG motor subtype groups are presented in Supplementary Material 2, Table S36. Relative to nFOG patients, FOG patients were slightly older at the time of study participation (*k* (*o*) = 19 (1,632), *g* = 0.14 [0.03–0.25], *p* =.015), had a longer disease duration (*k* (*o*) = 20 (1,706), *g* = 0.52 [0.37–0.68], *p* <.001), and reported higher LEDD (*k* (*o*) = 15 (1,442), *g* = 0.46 [0.24–0.68], *p* = <.001). Consistent with these results, FOG patients also had much greater overall motor impairment regardless of whether the UPDRS (original version; *k* (*o*) = 10 (979), *g* = 0.59 [0.36–0.83], *p* = <.001) or MDS-UPDRS (*k* (*o*) = 10 (727), *g* = 0.73 [0.46–1.00], *p* = <.001) was used. No significant effects were observed for years of education or age at time of disease onset.

#### Cognition

##### Main analyses

As shown in Table 15, our multilevel meta-analysis of 93 effect sizes from 20 studies revealed a moderate effect size for cognitive performance between FOG and nFOG subtypes, favouring the nFOG group. A multilevel Egger’s test indicated evidence of publication bias in both our full model and model with outliers removed using Cook’s distance approach (sunset [power-enhanced] funnel plots can be found in Supplementary Material 2, Fig. S16). This publication bias was not present when a residuals approach was used to remove nine outliers, resulting in a slightly reduced effect size (*g* = 0.3) relative to the full model (*g* = 0.35). Given the presence of publication bias in our full model, we ran our moderator and confound analyses with all available effect sizes (Supplementary Material 2, Tables S37, S39, S41) and then repeated these analyses with outliers (identified using the residuals approach) removed (Supplementary Material 2, Tables S38, S40, S42).

**Table 15:** Results of multilevel meta-analyses of cognitive performance in gait-related motor subtype pairs

Subtype pair	Model	*n* (*k*)	Pooled Hedges’ *g* (95% CI)	*p*	Within-study *I*^*2*^ (%) (*p*)	Between-study *I*^*2*^ (%) (*p*)	*Q* (*p*)	Multilevel Egger’s test (*p*)
FOG vs. nFOG^*^	All studies	20 (93)	− 0.35 (− 0.46, − 0.25)	** <.001**	22.37 (**<.001**)	20.17 (**.025**)	150.33 (**<.001**)	**.039**
	With outliers removed – residuals approach	20 (84)	− 0.3 (− 0.4, − 0.21)	** <.001**	3.52 (.267)	16.65 (.072)	72.93 (.777)	.286
	With outliers removed – Cook’s distance approach	17 (86)	− 0.29 (− 0.39, − 0.19)	** <.001**	13.49 (**.014**)	14.08 (.082)	101.07 (.113)	**.041**
Poor gait vs. good gait^†^	All studies	4 (9)	0.55 (0.22, 0.88)	**.005**	43.82 (.068)	3.05 (.5)	14.83 (**.063**)	.977
	With outliers removed – residuals approach	4 (8)	0.63 (0.35, 0.91)	**.001**	6.41 (.44)	4.29 (.5)	7.05 (.424)	.961
	With outliers removed – Cook’s distance approach	4 (6)	0.53 (0.21, 0.85)	**.008**	5.59 (.5)	9.2 (.5)	3.11 (.683)	.826

*Note*. CI = confidence interval; FOG = freezing of gait; *k* = number of effect sizes;* n* = number of unique studies; nFOG = nonfreezing of gait. ^*^For Hedges’ *g*, values < 0 indicate poorer cognitive performance among freezing of gait (FOG) patients relative to nonfreezing of gait (FOG) patients. ^†^For Hedges’ *g*, values > 0 indicate poorer cognitive performance among poor gait patients relative to good gait patients. Significant *p*-values (<.05) are highlighted in bold

With respect to cognitive status, two studies (de Almeida et al., 2021; Vercruysse et al., 2012) compared rates of MCI in FOG and nFOG subtypes. Consistent with the results of our multilevel meta-analyses of continuous cognitive data, in both studies, MCI was more prevalent among FOG patients relative to nFOG patients (*RR* = 1.18 and 1.8, respectively). However, as per our comparisons of MCI rates in other motor subtype pairs, MCI rates in these two studies differed considerably, despite both samples having similar demographics and disease characteristics (i.e., age, disease duration, age at disease onset). In de Almeida et al. (2021), all FOG patients and 84.6% of nFOG patients were classified as having MCI, whilst Vercruysse and colleagues (2012) reported MCI in only 39.1% of FOG patients and 21.7% of nFOG patients. This difference may be in part attributable to the screening tools used to determine MCI status; de Almeida et al. (2021) used the MoCA, whereas Vercruysse et al. (2012) used the SCOPA-COG.

In addition, one study (Ortelli et al., 2019) examined rates of domain-specific impairment (based on normative data) on ten cognitive tasks. Relative to nFOG patients, greater rates of domain-specific impairment were observed among FOG patients on tasks assessing processing speed (Trail Making Test-A [TMT-A]), working memory (Trail Making Test-B [TMT-B]), cognitive inhibition (Stroop task), visuospatial abilities (Rey-Osterrieth Complex Figure Test – copy), and visuospatial long-term memory/learning (Rey-Osterrieth Complex Figure Test – delayed). Whereas a greater proportion of FOG patients showed impaired cognitive flexibility on the WCST, no meaningful difference in impairment rates between FOG and nFOG patients were observed when cognitive flexibility was evaluated using the difference in time taken to complete TMT-A and TMT-B. Both subtypes also showed similar rates of impairment on tasks assessing semantic long-term memory/learning (semantic fluency) and language (phonemic fluency).

##### Moderator analyses

Results of our moderator analyses are presented in Supplementary Material 2, Tables S37–S42. For our analyses with no outliers removed, only cognitive class emerged as a significant moderator of effect size magnitude (*p* =.007; Supplementary Material 2, Table S39)^15^; compared with domain-specific measures of cognition (*g* = − 0.24 [95% CI − 0.35, − 0.12]), global measures of cognition (*g* = − 0.42 [95% CI − 0.54, − 0.3]) produced a larger difference between subtype groups, favouring the nFOG group.

Our more granular analysis of cognitive domains considered separately was not statistically significant; however, the effect size estimate for lexical short-term memory (*g* = − 0.01 [95% CI − 0.32–0.3]) did differ significantly from that for global cognitive function (*g* = − 0.43 [− 0.55, − 0.3], *p* =.013).^16^ The difference in pooled effect size between global cognitive function and three additional memory-based cognitive domains (lexical long-term memory, visuospatial long-term memory, executive function – working memory) also neared statistical significance (*p* range =.058–.087). In all cases, the effect size estimate was substantially *lower* for the memory domain than for global cognitive function (Supplementary Material 2, Table S39). These results indicate that whilst FOG patients may show poorer overall cognition relative to nFOG patients, the profile of cognitive impairment shown by patients with FOG may not include marked deficits in memory. These results may also explain why we found a significantly smaller pooled effect size for domain-specific measures of cognition relative to global measures of cognition; namely, these domain-specific memory measures may have been responsible for reducing the pooled effect size for domain-specific measures when considered collectively.

Notably, when we re-ran our moderator analyses with outliers removed, a significant difference emerged between the pooled effect size estimates for studies where motor function was assessed in the ON medication state (*g* = − 0.23 [95% CI − 0.34, − 0.12]) and studies where motor function was assessed ON or OFF medication (*g* = − 0.48 [95% CI − 0.69, − 0.26], *p* =.043; Supplementary Material 2, Table S40). Presumably, medication status at time of motor assessment influenced individual patients’ motor subtype classification, which had ramifications for the composition of the resulting motor subtype groups, leading to differences in the relative cognitive performance of these groups. Given that many patients only experience FOG either ON *or* OFF medication (Giladi, 2008; Okuma & Yanagisawa, 2008), it is reasonable to posit that studies where patients were assessed either ON or OFF medication were potentially best equipped to detect the presence of FOG and correctly classify patients into FOG and nFOG groups. More specifically, although not explicitly reported, these studies may have assessed each patient in the medication state under which, based on patient self-report, FOG was most likely to be observed. If these studies are presumed to have produced the most accurate FOG and nFOG subtype groups, the larger pooled effect size for cognitive differences between subtypes found for these studies should be considered a more precise estimate than studies where *all* patients were indiscriminately assessed for FOG either ON or OFF medication.

When outliers were removed, pooled mean years of education also emerged as a significant moderator of effect size magnitude (*p* =.04; Supplementary Material 2, Table S38); in studies with more highly educated samples, the difference in cognitive performance between FOG and nFOG groups was smaller. Based on our visual inspection of these data, however, this moderation effect seems to be primarily driven by a single study where a large difference in cognition between subtypes was observed within a poorly educated sample (Supplementary Material 2, Figure S17).

##### Confound analyses

For our analyses with no outliers removed, none of our confounds were statistically significant (Supplementary Material 2, Table S41). Following removal of outliers, the SMD in UPDRS-III scores (original version, *p* =.021, and any version, *p* =.018) emerged as a significant confound (Supplementary Material 2, Table S42). As the SMD in UPDRS-III score increased, reflecting greater overall motor impairment in the FOG group relative to the nFOG group, the difference in cognitive performance increased, reflecting poorer cognition in the FOG group (Supplementary Material 2, Fig. S18). This finding is consistent with the results of our other motor subtype comparisons and supports a broader relationship between motor and cognitive impairment in PD. Of note, when the SMD in UPDRS-III score (original version; any version) was introduced as a moderator, the remaining between-study *I*^*2*^ neared zero (< 0.00%), suggesting subtype differences in overall motor symptom severity explained most of the variance in effect size magnitude observed between studies.

### Other gait-related subtypes

#### Poor gait vs. good gait

Five studies performed motor subtyping on the basis of impaired gait. There was substantial heterogeneity in the subtyping procedures employed across studies. In two studies (Maidan et al., 2017; Pongmala et al., 2023), walking speed alone was used to determine gait quality; patients with walking speeds < 1 m per second were classified as having poor gait, whilst patients with walking speeds ≥ 1 m per second were classified as having good gait. In another two studies (Ehm et al., 2019; Margolesky et al., 2019), patients were subtyped based on their *tandem* gait performance. Patients were asked to walk heel-to-toe for ten consecutive steps; patients completed two trials, with the trial on which they performed best used to classify them as having either good or poor tandem gait. In Ehm et al. (2019), poor tandem gait was simply defined as one or more side steps being taken, whilst Margolesky et al. (2019) scored patients’ performance using the gait portions of two clinical rating scales (Unified Huntington's Disease Rating Scale (UHDRS; Huntington Study Group, 1996); Scale for the Assessment and Rating of Ataxia (SARA; Schmitz-Hübsch et al., 2006)) and applied cutoff scores to distinguish between “normal” (good) and “abnormal” (poor) tandem gait. Finally, in Allan et al. (2005), the Tinetti Balance Scale (Tinetti, 1986) was used to undertake a more holistic assessment of gait, balance, and fall risk across multiple tasks. Any patients categorised as having at least moderate impairments in either gait or balance according to this clinical rating tool were classified as having a gait disorder (poor gait).

##### Demographics and disease characteristics

Results of our traditional meta-analyses comparing poor gait and good gait motor subtype groups on demographics and disease characteristics are reported in Supplementary Material 2, Table S43. We found a significant, large effect for disease duration, with poor gait patients having a much longer disease duration relative to patients with good gait (*k* (*o*) = 4 (263), *g* = 0.95 [95% CI 0.87–1.04], *p* =.009). The confidence interval around this estimate was narrow, suggesting a high level of precision, but caution should be taken given the small size of the pooled sample. Although there were insufficient data for meta-analysis, inspection of UPDRS (*k* = 1) and MDS-UPDRS (*k* = 1) data also indicated a trend towards patients with poor gait having much greater overall motor impairment relative to patients with good gait (*g* range = 0.75–4.66).

##### Cognition

Our multilevel meta-analysis of nine effect sizes from four studies indicated a moderately sized difference (*g* = 0.55 [95% CI 0.22–0.88], *p* =.005) in cognitive function that favoured patients with good gait (Table 15). Interestingly, removal of outliers either led to a slight increase or decrease in the pooled effect size estimate, depending on whether outliers were identified using residuals or Cook’s distance. Of note, the 95% confidence intervals around these estimates are wide (ranging from a small to large effect), suggesting poor precision. This may be attributable to the small number of included studies, or to the between-study heterogeneity in subtyping methods used; against the latter point, however, our heterogeneity measures (between-study *I*^*2*^) were not significant for any of the three models. Consistent with the large effect size found for studies that used continuous measures of cognitive function, Allan and colleagues (2005) found patients with poor gait to be at much greater risk of dementia (diagnosed based on CAMCOG cutoff scores) compared to patients with good gait (*RR* = 6.6, *o* = 92).

No dose–effect, moderator, or confound analyses were performed due to an insufficient number of studies (*n* < 10). However, considering the large effect size observed for disease duration between poor gait and good gait patients, it is not unreasonable to speculate that the difference in cognitive function between these subtypes could, at least in part, be attributed to longer disease duration among poor gait patients.

#### Fallers vs. nonfallers

One study (Altmann et al., 2023) subtyped patients according to whether they had at least one fall during a 2-week inpatient stay at a PD clinic. This study’s results are summarised in Supplementary Material 2.

### Risk of bias

**Fig. 4:**
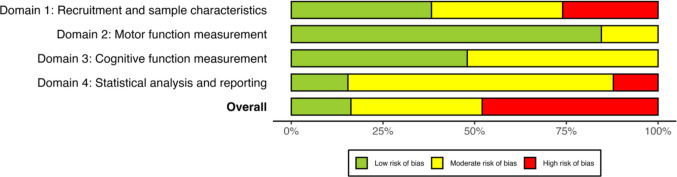
Risk of bias summary plot for all included studies

A risk of bias summary plot for all included studies is presented in Fig. 4. Using our custom quality assessment tool, we found that almost half (*n* = 59, 48%) of included studies had a high risk of bias; 44 studies (35.8%) had a moderate risk of bias; and only 20 studies (16.3%) had a low risk of bias. High risk of bias was generally due to poor reporting of recruitment and sample characteristics (Domain 1) and poor statistical analysis and reporting (Domain 4). We found that many studies did not specify how participants were recruited (e.g., community vs. clinic-based recruitment) or clearly report participant medication status at the time of motor and cognitive assessments (see Pooled sample characteristics). This was somewhat surprising and concerning, given the well-documented effects of medication on both motor symptom severity and cognitive function in PD (Connolly & Lang, 2014; Roy et al., 2018), and limited our ability to test for medication effects at the time of motor and cognitive assessment.

Poor statistical analysis and reporting often resulted from incomplete reporting of cognitive and motor data for each motor subtype group of interest. Of 123 studies, only one study reported effect sizes for all motor and cognitive variables of interest. Although many studies included sufficient descriptive statistics (e.g., means, standard deviations) for effect size calculations to be performed, selective reporting of cognitive outcome data was not uncommon. In several cases, author contact was required to request summary data for cognitive measures that had been described in the Methods section, but then never again mentioned (i.e., no data reported for whole sample, for motor subtype group comparisons, or for any other subgroups).

**Fig. 5:**
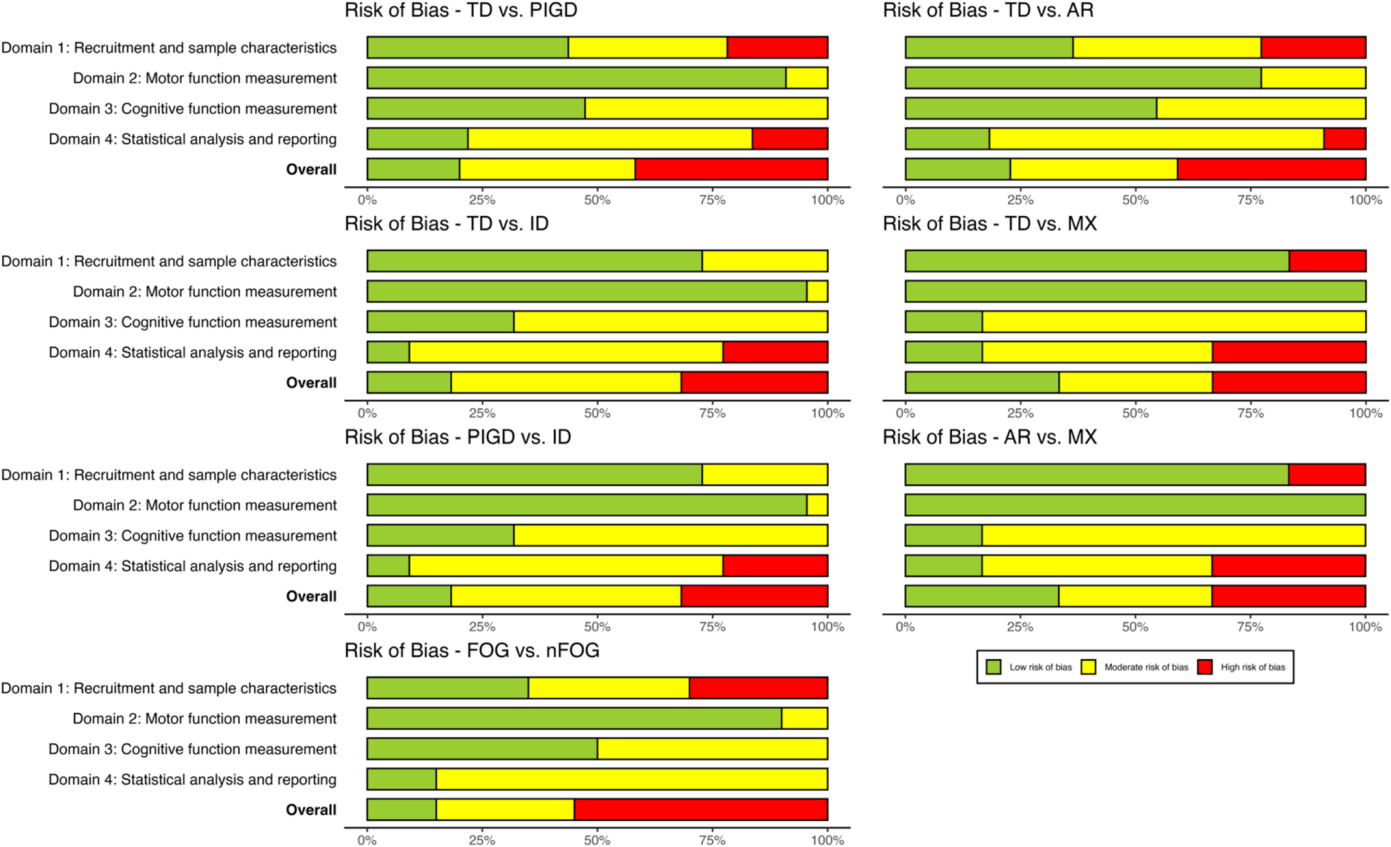
Risk of bias summary plots for most common motor subtype pairs. *Note.* AR = akinetic-rigid; FOG = freezing of gait; ID = indeterminate; MX = mixed; nFOG = nonfreezing of gait; PIGD = postural instability gait disorder; TD = tremor-dominant

**Fig. 6:**
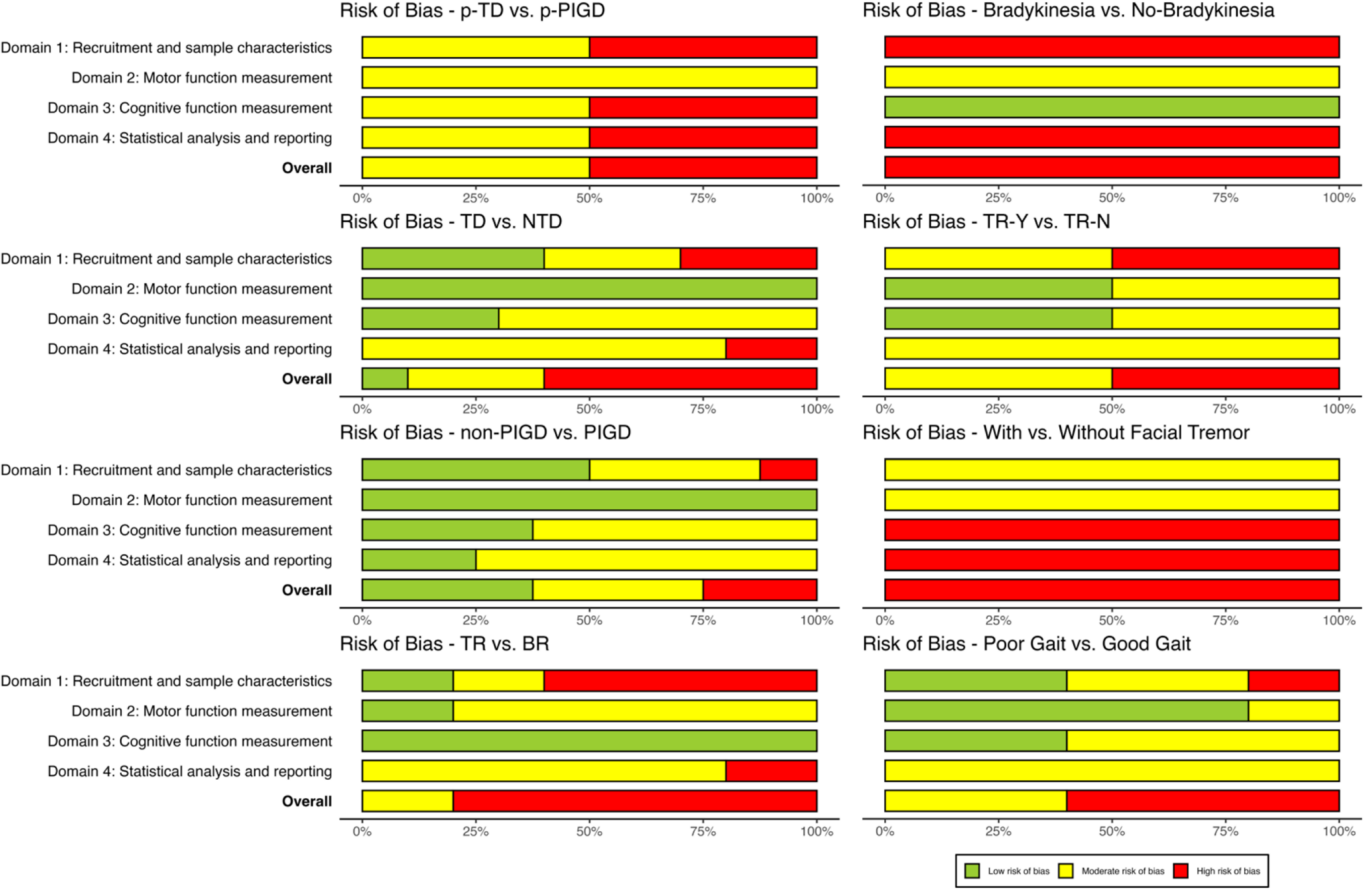
Risk of bias summary plots for other motor subtype pairs. *Note.* BR = bradykinesia; NTD = nontremor-dominant; PIGD = postural instability gait disorder; p-PIGD = predominant postural instability gait disorder; p-TD = predominant tremor-dominant; TD = tremor-dominant; TR = tremor; TR-Y = tremor; TR-N = no tremor

With regards to the reporting of the subtyping method used, where well-established subtyping methods had been used (Jankovic et al., 1990; Stebbins et al., 2013), studies often reported this by citing the corresponding paper. In some cases, however, there was ambiguity regarding *which* subtyping method had been used—either Jankovic et al. (1990) or Stebbins et al. (2013)—owing to unclear or incorrect reporting of *which* UPDRS version had been used, and/or a tendency to cite Stebbins et al. (2013) when Jankovic et al.’s (1990) original procedure, which is reprinted in Stebbins et al. (2013), was used (i.e., there were many instances of citing a method alongside the discordant UPDRS version). Where novel subtyping methods were employed that had not been documented elsewhere, many authors did not provide sufficient information (e.g., exact cutoff scores used) for these methods to be replicated. This can be seen in our risk of bias summary plots for each motor subtype pair, presented in Figs. 5 and 6.^17^ Generally, risk of bias was higher for studies reporting on motor subtype pairs that are less well-established (Fig. 6) than for studies reporting on well-established motor subtype pairs (Fig. 5), especially in the domain of statistical analysis and reporting (Domain 4).

With respect to cognitive function measurement (Domain 2), we also found that more than half of included studies (*k* = 64, 52%) were assessed as having a moderate risk of bias, often due to relying exclusively on one or more coarse, global measures of cognition (e.g., MMSE, MoCA) to assess cognitive function within their sample. Given preliminary evidence for differential impairment across cognitive domains, however, domain-specific assessments of cognition are necessary to elucidate the unique cognitive profile of each motor subtype.

### GRADE

A summary of GRADE framework findings (used to evaluate pooled effects for motor subtype pairs with continuous cognitive outcome data available for at least 10 studies) can be found in Supplementary Material 2, Table S44. Quality of evidence for all motor subtype pairs was deemed low or very low.

### Summary of results

**Fig. 7:**
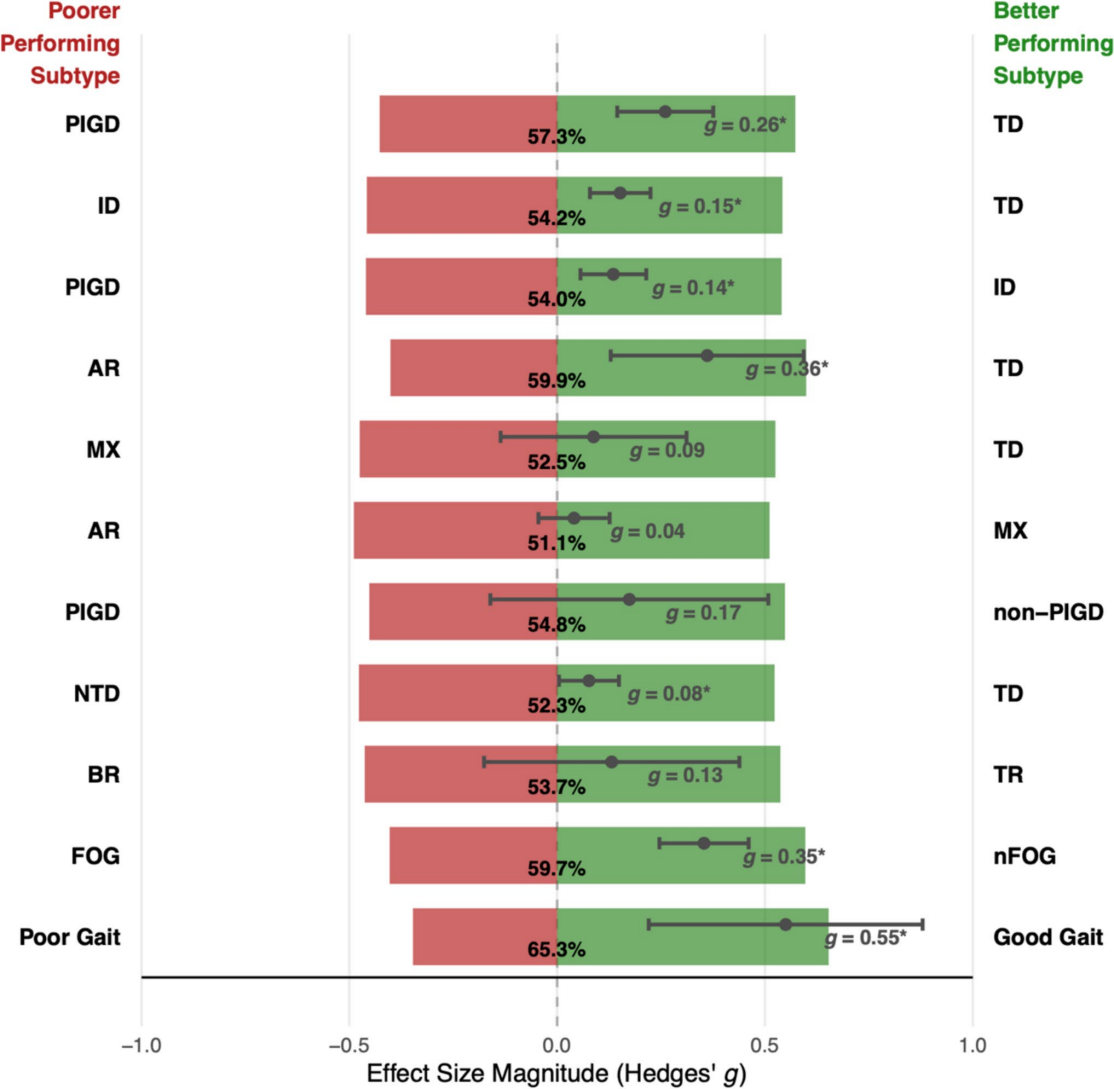
Cognitive performance differences between motor subtypes in Parkinson's disease. *Note.* Bar width represents the probability of superiority (occasionally referred to as the common language effect size), which quantifies the likelihood that a randomly selected individual from one group will have better cognitive performance than a randomly selected individual from the comparison group. This probability is expressed as a percentage. For example, a value of 65% means that in 65 of 100 random pairings, an individual from the better-performing group would show superior cognitive functioning compared to an individual from the poorer-performing group. Red bars represent the proportion where the poorer-performing subtype outperforms the better-performing subtype, while green bars represent the proportion where the better-performing subtype outperforms the poorer-performing subtype*.* Points and error bars show Hedges’ *g* and 95% confidence interval for full model (no outliers removed). *Statistical significance (*p* <.05)

Figure 7 provides a graphical summary of the results of our multilevel meta-analyses. This figure includes all motor subtype pairs for which sufficient continuous cognitive data were available to perform a multilevel meta-analysis.

## Discussion

This is the first systematic review and meta-analysis to investigate the relationship between motor subtype and cognitive impairment in PD across multiple subtyping frameworks. To our knowledge, only one other related systematic review and meta-analysis has previously been published, which specifically examined cognitive differences between FOG and nFOG subtypes (Monaghan et al., 2023). Our review and meta-analysis was much more ambitious in breadth and evaluated differences in cognitive function across *multiple* motor subtyping frameworks, not just FOG/nFOG. This uniquely positioned us to compare differences in cognition between qualitatively similar subtyping pairs, something which would be difficult to achieve if subtyping frameworks were appraised in separate reviews. We found robust evidence for a motor-cognitive relationship in PD. This relationship was observed across several motor subtyping frameworks, with similar patterns of impairment found across subtype pairs with qualitatively similar groups (e.g., TD/PIGD, p-TD/p-PIGD). Overall, we found strong evidence for an association between tremor and better-preserved cognition and between FOG and poorer cognition in PD. Our results underscore the potential for motor subtyping to be used to improve understanding of likely cognitive trajectory in PD, which can inform clinical decision making.

### Tremor-dominant, postural instability gait disorder, and indeterminate motor subtypes

Although many PD motor subtypes have been proposed, the TD and PIGD subtypes remain the most widely recognised and researched (Marras, 2015). These motor subtypes gained popularity as a consequence of their intuitive appeal, straightforward classification procedures (Jankovic et al., 1990; Stebbins et al., 2013), and demonstrated utility for characterising patient heterogeneity along other dimensions such as disease duration, treatment response, and survival (for review, see Fereshtehnejad & Postuma, 2017; Qian & Huang, 2019; Thenganatt & Jankovic, 2014).

Of 123 included studies, 55 compared the cognitive performance of TD and PIGD motor subtype groups. Our multilevel meta-analysis of continuous data found a small-to-moderate difference in cognition that favoured TD patients. We have a high level of confidence in this result for several reasons. First, our meta-analysis was well-powered, synthesising 252 effect sizes from 50 studies. This contributed to a precise pooled effect size estimate with a narrow 95% confidence interval. Second, the magnitude of the pooled effect was maintained following removal of outliers using two different approaches. Third, similar pooled effects were found regardless of the subtyping procedure used to assign patients to TD and PIGD groups. This suggests that the difference in cognition between these motor subtype groups is genuine, and not simply an artefact of a particular classification method. Fourth, our results are coherent with broader research findings, which associate the TD subtype with a more benign disease course (Fereshtehnejad & Postuma, 2017; Jankovic & Kapadia, 2001). Finally, the results of our multilevel meta-analyses were consistent with those of our traditional meta-analyses, which found significantly higher rates of both MCI and dementia among PIGD patients relative to TD patients. Taken together, these findings provide strong evidence for cognitive function being better preserved in TD patients relative to PIGD patients.

Similar, albeit weaker, patterns of cognitive impairment were found when we compared subtype groups qualitatively similar to the TD and PIGD subtypes. In general, better cognitive performance was observed in TD-like subtypes, whilst poorer cognition was observed in PIGD-like subtypes. When the PIGD subtype was compared to a non-PIGD subtype, a small pooled effect size favouring the non-PIGD group was found. We also found a negligible difference in cognition between TD and NTD groups which favoured the TD group. Finally, in one study comparing p-TD and p-PIGD subtypes (Herman et al., 2015), moderate differences in global cognitive function, favouring the p-TD subtype, were reported. Only one study, which compared patients with and without facial tremor, found an association between the presence of tremor and *worse* cognition (Ou et al., 2021).

Compared with the results of our TD vs. PIGD comparison, the differences in cognition between these related subtype pairs lacked precision and were either smaller in magnitude or failed to reach statistical significance. In part, this may be attributable to the relatively few studies investigating these motor subtypes (*n* range = 1–10 studies), and, in some cases, high between-study heterogeneity. For the TD vs. NTD and PIGD vs. non-PIGD comparisons, the attenuated effect is likely a direct consequence of the subtyping procedures used. In most included studies (e.g., Mao et al., 2018; Pelicioni et al., 2019), the NTD and non-PIGD groups were formed by first classifying patients into TD, PIGD, and ID subtypes, and then combining the ID group with either the PIGD (for NTD) or TD (for non-PIGD) group. For our TD vs. ID comparison, our multilevel meta-analysis models found a small but significant pooled effect that favoured TD patients, and our traditional meta-analyses showed reduced rates of MCI and dementia among TD patients. We found a similar pattern of results—namely, a significant and robust small pooled effect—for our PIGD vs. ID comparisons, this time reflecting better cognition among ID patients. Taken together, these results suggest a graded hierarchy of cognitive performance across the TD, ID, and PIGD subtypes, with the ID subtype showing an intermediate level of cognitive function. This may explain why, as a result of combining the ID subtype with either the TD or PIGD group, the differences in cognition observed for our TD vs. NTD and PIGD vs. non-PIGD comparisons were smaller than for our TD vs. PIGD comparison.

In both Jankovic et al. (1990) and Stebbins et al.’s (2013) subtyping procedures, the ID subtype captures patients whose ratio of tremor to PIGD symptoms falls within a midrange between the thresholds for the TD and PIGD subtypes. Given this, it is perhaps unsurprising that some papers (e.g., Kwon et al., 2021) incorrectly identify “ID” as referring to an “intermediate” rather than “indeterminate” subtype. Interestingly, our findings reveal a pattern of cognitive impairment across the TD, PIGD, and ID subtypes that mirrors this spectrum of motor impairment; both motorically *and* cognitively, ID is an intermediary between the TD and PIGD subtypes.

The observation that patients tend to convert from the TD to PIGD subtype over time, but rarely vice versa (Alves et al., 2006), has prompted some to suggest that these motor subtypes simply reflect different *disease stages* rather than distinct “subtypes” of PD (Nutt, 2016). This suggestion is bolstered by research showing that the TD subtype is characterised by a shorter disease duration and less overall motor impairment, relative to the PIGD subtype. Indeed, our own traditional meta-analyses found significant, small pooled effects reflecting longer disease duration and greater overall motor impairment among PIGD patients. On the basis of this, it could be reasoned that the difference in cognitive function between TD and PIGD subtypes is simply a consequence of the PIGD subtype capturing a later disease stage, characterised by greater disease progression. Our moderator and confound analyses provide some evidence against this explanation, however. Neither pooled mean disease duration nor the SMD in disease duration moderated the magnitude of the difference in cognitive performance between TD and PIGD patients. While the SMD in overall motor symptom severity *was* a significant moderator, this effect was not particularly robust; the SMD in motor symptom severity was only a significant confound when assessed using the original UPDRS-III, not the MDS-UPDRS-III. Given this, although the TD and PIGD subtypes undoubtedly differ along the dimensions of disease duration and motor symptom severity, our results contradict the hypothesis that the TD and PIGD subtypes *only* reflect differences in disease stage.

### Tremor-dominant, akinetic-rigid, and mixed motor subtypes

Twenty-two studies included in our review compared a TD subtype to an AR subtype. Half of these studies used Kang et al.’s (2005) subtyping procedure, where, similar to Jankovic et al. (1990) and Stebbins et al.’s (2013) methods, patients are classified into TD, AR, or MX subtype groups according to their ratio of UPDRS tremor to UPDRS AR subscores. Despite being distinct frameworks, there was considerable agreement between our TD/PIGD/ID and TD/AR/MX meta-analyses. First, our multilevel meta-analyses of the latter framework revealed better cognitive function among TD patients relative to AR and MX patients, providing further evidence of TD being associated with better preserved cognition in PD. Second, we saw parallels in the relative cognitive performance of the MX and ID motor subtypes. Our meta-analyses trended towards the MX subtype having better cognitive function relative to the AR group, but not the TD group; across both comparisons (TD vs. MX, AR vs. MX), however, the pooled effect was of a negligible magnitude and was not statistically significant. These results, considered alongside the significant, moderate difference in cognition between TD and AR patients, situates the MX subtype as a motor *and* cognitive intermediary between the TD and AR groups.

With respect to qualitatively similar motor subtype pairs, our review included five studies that compared a TR subtype to a BR subtype. Our multilevel meta-analyses revealed a nonsignificant, negligible-to-small difference in cognitive function between these subtypes, which was not consistent with the results of our TD vs. AR comparisons. This may, in part, be attributable to the limited data available for this comparison, taken from studies that were highly underpowered to detect an effect (median power = 9.6%; Supplementary Material 2, Fig. S7). Alternatively, it may be that bradykinesia, considered independent of the other motor symptoms encapsulated in the broader AR subtype (i.e., akinesia, rigidity), is insufficient for detecting differences in cognition.

### Freezing of gait and other gait-related motor subtypes

Twenty studies compared FOG and nFOG subtypes. Our multilevel meta-analysis found a moderate effect indicating better cognition in nFOG patients relative to FOG patients, which only reduced in magnitude slightly following removal of outliers. Our synthesis of cognitive status data also indicated higher prevalence of MCI among FOG patients compared with nFOG patients. Of note, the pooled effect obtained from our multilevel meta-analysis (*g* = 0.35 [95% CI 0.25–0.46]) overlaps with the effect size estimates (*d* range = 0.21–0.5) reported in Monaghan et al.’s (2023) recent meta-analysis comparing the cognitive performance of FOG and nFOG subtypes across five cognitive domains.

We also found that FOG patients had significantly greater overall motor impairment and longer disease duration relative to nFOG patients. The finding of poorer cognitive function among FOG patients is, therefore, consistent with cognitive symptoms worsening over time alongside worsening motor symptoms, and corresponds with the pattern of worse cognition, greater motor impairment, and longer disease duration observed in PIGD patients relative to TD patients. Importantly, our moderation and confound analyses indicated that the FOG and nFOG subtypes capture variance in cognitive function that cannot be explained by motor symptom severity or disease duration alone. Neither pooled mean disease duration nor the SMD in disease duration significantly moderated the magnitude of the difference in cognition between FOG and nFOG groups. While the SMD in motor symptom severity *was* a significant confound when outliers were removed, this effect was, again, not particularly robust. Similar to the TD/PIGD comparison, the SMD in UPDRS-III score was a significant confound when the original version was analysed alone and when both UPDRS-III versions were analysed together, but not when the MDS-UPDRS was analysed separately. Nevertheless, it is pertinent to highlight that the between-study *I*^*2*^ for models where the SMD in UPDRS-III score was a significant confound neared zero (< 0.00%); thus, we cannot rule out the possibility that differences in overall motor severity do account for a significant portion of the variance in cognitive differences observed between the TD/PIGD and FOG/nFOG subtypes. Given that these results were not robust across different measures of motor function and were highly sensitive to outliers, further investigation is warranted.

Our synthesis of studies comparing other gait-related subtype pairs revealed similar patterns. Only one study compared fallers and nonfallers, reporting poorer cognition among fallers relative to nonfallers. Our multilevel meta-analyses comparing poor gait and good gait subtypes indicated that patients with poor gait had significantly worse cognitive performance than those with good gait. Notably, this comparison produced the largest pooled effect size estimate across *all* motor subtype pairs for which we performed a multilevel meta-analysis; however, data were synthesised across a relatively small sample (*n* = 4, *k* = 9) and the resulting estimate lacked precision. It is thus unlikely that this result reflects a *genuinely* larger difference in cognitive performance between good gait and poor gait subtypes compared to other subtype pairs. However, it is important to note that the pooled effect sizes for our FOG vs. nFOG comparisons also tended to be larger than those obtained for our TD vs. PIGD and TD vs. AR comparisons. Taken together, these results indicate that gait-based subtyping frameworks may be more sensitive to differences in cognition than other frameworks. Compared with other PD motor symptoms (e.g., tremor, rigidity), gait impairments arguably have a stronger cognitive component; attention, response selection, and visuospatial abilities have all been implicated in gait performance and the manifestation of FOG (Giladi & Hausdorff, 2006; for reviews, see Amboni et al., 2013; Heremans et al., 2013). This may explain why gait-based motor subtypes are particularly sensitive to differences in cognition between patients.

#### Generalisability and implementation of freezing of gait vs. nonfreezing of Gait subtypes

##### Generalisability

Despite its utility in capturing differences in cognitive profile, some potential issues regarding the generalisability and implementation of the FOG/nFOG subtyping framework are worth considering. First, it remains uncertain as to whether the FOG/nFOG framework could be applied to newly diagnosed patients. It is well established that the FOG subtype is associated with longer disease duration; indeed, our own traditional meta-analysis found FOG patients to have significantly longer disease duration than nFOG patients. Cognitive impairment is also known to increase with disease duration in PD, irrespective of motor subtype (Baiano et al., 2020). This raises the possibility that the larger difference in cognition seen between FOG and nFOG groups relative to other subtype pairs may simply be a consequence of the larger difference in disease duration between FOG and nFOG patients.

Against this explanation, and as aforementioned, neither pooled mean disease duration nor the SMD in disease duration significantly moderated the difference in cognition between FOG and nFOG subtypes. We are hesitant to conclude from this, however, that the FOG/nFOG framework could be used to detect differences in cognitive performance between patients of any disease duration, particularly in early PD. This is because FOG/nFOG studies tended to recruit samples with a longer disease duration, restricting the range of disease durations present in our pooled data and reducing the power to detect the effect of this potential confound. Studies comparing FOG and nFOG subtypes reported a mean disease duration of 7.5 years (*SD* = 2.2, range = 3.6–10.9 years, *n* = 20), while the mean disease duration for studies reporting on other motor subtyping frameworks was 5.5 years (*SD* = 3, range = 0.2–13.6 years, *n* = 88). No FOG/nFOG studies recruited a de novo sample. Consequently, it is unclear whether these results would generalise to recently diagnosed patients.

##### Implementation

Unlike the TD/PIGD/ID and TD/AR/MX subtype frameworks, where certain classification procedures have been widely adopted, there is little to no consensus regarding the classification of patients into FOG and nFOG groups. Interestingly, despite much greater heterogeneity in the subtyping methods used by FOG/nFOG studies, our multilevel meta-analysis produced a pooled effect of slightly larger magnitude and similar precision to those found for our TD vs. PIGD and TD vs. AR comparisons. Nevertheless, successful application of the FOG/nFOG framework in clinical settings depends on consensus being reached on a clear, reproducible subtyping procedure.

### Moderators and confounds

One of the strengths of our meta-analysis was our thorough exploration of potential moderators and confounds, including demographics, disease characteristics (e.g., age at onset), and methodological features (e.g., risk of bias). We have already addressed the results of several of these analyses in earlier sections, including those pertaining to subtype method, disease duration, and motor symptom severity. In this section, we focus on several other moderators and confounds of interest. Importantly, with the exception of some of our analyses examining the SMD in UPDRS-III scores, none of our significant moderators or confounds were able to account for all of the variance in effect size magnitude. This provides strong evidence for the utility of motor subtypes for making inferences about an individual’s cognitive profile that cannot be inferred from other information about the patient (e.g., age, disease duration).

#### Demographics and disease characteristics

For both our TD vs. PIGD and ID vs. PIGD comparisons, the SMD in age and the SMD in age at onset emerged as significant confounds. As the SMD in age increased, reflecting older age in the motor subtype with poorer cognition (PIGD), the pooled effect size also *increased*; when the SMD in age increased in the opposite direction, reflecting older age in the motor subtype with better cognition (TD or ID), the pooled effect size *decreased.* This result is suggestive of a negative influence of older age on cognitive performance, irrespective of motor subtype. The same pattern of results was observed for the SMD in age at disease onset and is consistent with older age at onset being associated with more rapid disease progression (Raket et al., 2022).

The difference in cognition between TD and PIGD subtypes was also confounded by the SMD in education years; as the difference in education years between subtypes increased, reflecting more education years in the TD group, the difference in cognitive performance also increased, favouring the TD group. Similarly, pooled mean years of education was found to moderate the difference in cognition between FOG and nFOG groups; as the pooled mean years of education increased, the difference in cognitive performance between subtypes *decreased*. However, the SMD in years of education was not a significant confound, suggesting that the difference in cognition between the two subtypes was not driven by group differences in education level. Within the context of education being a proxy for cognitive reserve, the moderating influence of education among TD/PIGD and FOG/nFOG subtypes underscores the potential protective effects of modifiable lifestyle factors on cognitive decline in PD; in both cases, higher education levels minimised the cognitive impairment observed in the poorer performing motor subtype group (PIGD and FOG).

Finally, we found that the difference in cognition between FOG and nFOG patients was larger when patients were subtyped based on the presence of FOG in the ON *or* OFF medication states compared to when patients were subtyped according to presence of FOG in the ON state alone (Supplementary Material 2, Table S39). The relationship between Parkinsonian medication and FOG is complex; many patients experience more frequent and longer FOG episodes in the OFF state compared with the ON state (Schaafsma et al., 2003), but there are also cases of medication-induced FOG (Espay et al., 2012; Giladi, 2008). In potentially allowing for patients who experience FOG under *either* medication state (ON or OFF) to be classified as belonging to the FOG subtype, studies that assessed motor function under ON or OFF medication states presumably had the lowest likelihood of misclassifying patients with FOG as nFOG. Given this, one interpretation is that the larger pooled effect observed for these studies provides a more precise estimate of the true difference in cognition between FOG and nFOG subtypes. This result has consequences for future FOG/nFOG research, as it highlights the potential importance of conducting FOG assessments both ON and OFF medication for accurate subtype classification.

#### Cognitive domain

One of our review aims was to compare the sensitivity of global and domain-specific measures of cognition in detecting differences between motor subtypes. With the exception of the FOG/nFOG subtypes, the pooled effect size estimates obtained for global and domain-specific measures of cognition did not differ significantly from each other.^18^ This suggests that short-form global measures are as sensitive in detecting cognitive differences between motor subtypes as domain-specific measures, when considered holistically. Importantly, however, these analyses compared a pooled effect for all global measures to a pooled effect for *all* domain-specific measures, meaning that any variation in effect sizes between domains was masked.

To explore variation across cognitive domains, we performed additional moderator analyses where the pooled effect estimates for each domain were considered separately. Relatively few studies administered domain-specific measures, contributing to imprecision in the pooled effects for most domains. It is unlikely that reducing the number of cognitive domains we considered (e.g., having a single long-term memory/learning domain), thereby increasing the number of effects per domain, would have substantially reduced this imprecision; wide confidence intervals were still present for cognitive domains for which we had an ample number of effect sizes (e.g., working memory, processing speed; e.g., Supplementary Material 2, Table S5). This imprecision led to substantial overlap in the confidence intervals surrounding the pooled effects for each domain (e.g., Supplementary Material 2, Fig. S2) and, as a consequence, cognitive domain did not significantly moderate effect size magnitude for any subtype pair. Inspection of the pooled effects for individual domains nevertheless provided some insight into differences between cognitive domains.

Across *all* motor subtype frameworks, differences in cognition were most pronounced for executive functions. This is consistent with research that has found executive dysfunction to be a hallmark of cognitive decline in PD (for reviews, see Dirnberger & Jahanshahi, 2013; Kehagia et al., 2013; Kudlicka et al., 2011), irrespective of motor subtype. However, given the small sample from which they were derived, these results should be considered preliminary. Relative to PIGD patients, TD patients showed marked advantages on measures of cognitive flexibility, response inhibition, and visuospatial reasoning. For our TD vs. ID and PIGD vs. ID comparisons, the pooled effect for cognitive flexibility was significantly larger than that for global cognition, and for TD vs. AR, the pooled effects for cognitive inhibition and working memory were larger than for global cognition. Finally, with respect to our FOG vs. nFOG comparison, the pooled effect sizes were largest for cognitive flexibility, cognitive inhibition, and planning. These results are broadly aligned with those of Monaghan et al.’s (2023) recent meta-analysis, which found the difference between FOG and nFOG patients to be largest on tasks assessing executive function (*d* = 0.5).

#### Dose–effect analyses

Our dose–effect analyses sought to evaluate the SMDs in UPDRS tremor and PIGD subscores as potential moderators of the difference in cognition between the TD, PIGD, and ID motor subtypes. In addition, we also investigated whether the SMD in UPDRS tremor subscore moderated the difference in cognition observed between TD and AR patients. We reasoned that if any of these moderators were significant, this would be evidence of that subscore primarily driving the observed effect, and would point to a more parsimonious subtyping approach (e.g., based on tremor assessment alone) being informative for making inferences about a patient’s cognitive profile.

None of these analyses were statistically significant, suggesting that neither tremor nor PIGD/AR subscore, considered independently, explained a significant amount of variance in effect size magnitude. With respect to differences in cognitive outcomes, therefore, our results indicate that the *relative* severity of tremor to PIGD/AR symptoms captures something distinct that cannot be captured by the severity of a patient’s tremor, PIGD, or AR symptoms alone. However, contrary to these results, our comparison of several univariate motor subtype frameworks (TR-Y/TR-N, BR/no-BR, with/without facial tremor) suggest that subtyping along a single dimension of motor function *can* capture meaningful differences in cognitive performance. It should be acknowledged that all three of these subtype comparisons were based on data collated from few studies (*n* range = 1–2 studies); given this, none provide especially strong evidence for meaningful differences in cognition being elucidated by subtyping frameworks that classify patients along one dimension of motor function.

#### Nonsignificant moderators and confounds

The majority of our moderator and confound analyses returned nonsignificant results. In some cases, these analyses were well powered, and the nonsignificant results were informative. For example, we found that the magnitude of the difference in cognitive performance between TD, PIGD, and ID subtypes did not vary with subtype method. This finding is consequential, as it provides evidence of Stebbins et al.’s (2013) subtyping procedure producing motor subtype groups that are equivalent—at least with respect to cognitive profile—to those produced by Jankovic et al.’s (1990) original procedure. In other cases, however, our nonsignificant results were likely, at least in part, attributable to the limited data available. We would encourage more detailed reporting of potential moderators so that the interactions between motor subtype differences in cognition and these variables may be more thoroughly investigated.

### Strengths

Our review was strengthened by our broad search strategy. Historically, subtyping studies have used vague or inconsistent terms to describe their approaches; for example, rather than “subtype” or “subtyping”, several studies refer to “subgroups”, “phenotypes”, or simply “heterogeneity” (e.g., Graham & Sagar, 1999; Paulus & Jellinger, 1991; Wojtala et al., 2019). Our search strategy was comprehensive and included all of these terms, along with related terms, such as “classification” and “cluster”. Although we made every effort to identify as many eligible studies as possible within a highly heterogenous body of literature, some studies were undoubtedly missed; however, given the large number of included studies, we are confident that we have captured a sizeable, representative portion of the literature.

We also kept our inclusion criteria broad with respect to the reporting of motor and cognitive data. Eligible studies were not limited to those investigating the relationship between motor subtype and cognition; we included all studies that performed motor subtyping and reported administering one or more objective or clinician-rated cognitive assessments, regardless of whether these cognitive data were reported in relation to motor subtype. Although author contact was necessary in 155 cases to obtain the data required to include these studies in our review—and many studies were excluded following unsuccessful data requests—we are confident that this approach helped to reduce publication bias. Indeed, only a small handful of our meta-analyses returned significant Egger’s tests.

In contrast, we employed stringent inclusion criteria with respect to both motor subtyping procedure and cognitive assessment. We excluded studies that used a combination of motor and nonmotor features to subtype patients. This was critical to our review objectives, as the inclusion of subtyping procedures that relied on any nonmotor features would have confounded the relationship between motor function and cognitive performance. We also required all studies to use at least one objective or clinician-rated motor assessment to classify patients. This maximised the likelihood of patients’ motor symptoms being accurately assessed, thereby ensuring that they were assigned to the correct motor subtype. This had especially marked consequences for the number of FOG/nFOG studies that were eligible for inclusion in our review, as most FOG/nFOG studies rely solely on self-reported FOG. Our decision to exclude these studies is, however, justified by research showing poor correlations between self-reported and clinician-rated FOG (Shine et al., 2012). Similarly, we only included studies where cognition was assessed using objective or clinician-rated tasks. As with motor function, this decision was justified by the well-documented disparity between patient- or caregiver-reported cognitive impairment and clinically diagnosed or objectively assessed cognitive impairment in PD (Copeland et al., 2016; Koerts et al., 2011; Siciliano et al., 2021).

Finally, our review was strengthened by our use of multilevel meta-analysis. In conventional meta-analysis, all effect sizes must be independent of one another; any effect size dependency is often overcome by including only one effect size per study or averaging across effect sizes, resulting in information loss and limited investigation of heterogeneity (Gucciardi et al., 2022). Multilevel meta-analysis allows for multiple effect sizes from a single study to be included in the same analysis, resulting in no data loss. In our review, 81 studies with continuous cognitive data reported more than one effect size, often taken from tasks assessing different cognitive domains (e.g., attention, working memory). Taking a multilevel approach to our analysis was therefore highly appropriate, as it allowed us to leverage all available data and investigate heterogeneity with respect to differences across cognitive domains.

### Limitations

#### Cross-sectional data

It is important to recognise several key limitations of our review. First, because our analyses only included cross-sectional data, the temporal order and causal link(s) between motor subtype and cognitive function remain unclear. While our review provides robust evidence for differences in cognition between motor subtypes, our results offer no insight into the neurobiological mechanisms driving these cognitive differences. Moreover, our results do not establish whether the neurobiological processes that give rise to these cognitive differences are integral to—or merely covary with—the mechanisms that determine a patient’s motor subtype; cross-sectional data precludes any inference about casual or temporal precedence.

In the case of TD and PIGD subtypes, for example, it is often presumed that the poorer cognitive performance among PIGD patients is a consequence of one or more features of PD pathology that are more advanced in, or perhaps even unique to, the PIGD subtype. This position is supported by recent research by Vijayakumari and colleagues (2025), who used a data-driven approach to derive two neuroanatomical PD subtypes from structural MRI data. When these neuroanatomical subtypes were compared on motor and cognitive measures four years later, results supported a confluence of poor cognition and PIGD motor symptoms that was specifically observed within patients belonging to the same neuroanatomical subtype (characterised by brain atrophy in frontal and subcortical regions). These findings complement our review and illustrate the potential biological correlates underpinning the motor-cognitive relationship illustrated here. Elucidating these biological correlates and determining their potential causal role has important implications for prognosis and treatment of both motor and cognitive symptoms in PD. We would encourage further research in this area, informed by the motor-cognitive phenotypes substantiated by our review.

With respect to temporal order, the cross-sectional data included in our review and meta-analyses cannot elucidate whether motor subtype presentation precedes cognitive changes (i.e., cognitive impairment in PIGD and FOG), or vice versa. Identifying the temporal order of these two disease dimensions is critical for guiding investigation of their respective (or shared) aetiologies, and for informing how this motor-cognitive relationship might be utilised for prognostic purposes in clinical settings. Historically, PD diagnostic criteria have differentiated PD from Lewy Body Dementia in requiring motor symptoms to emerge *prior* to the onset of cognitive symptoms (Gibb & Lees, 1988); consistent with this, it is often assumed that a patient’s motor subtype precedes the development of cognitive impairment. Thus, while, as abovementioned, it is generally assumed that a common neuropathological process drives both motor phenotype and cognitive status in parallel, motor subtype might also directly contribute to cognitive decline through activity restriction. For example, although TD patients may withdraw from public activities due to social stigma associated with their tremor, those belonging to the PIGD and FOG subtypes experience greater mobility limitations and fall risks, potentially leading to more marked activity restriction (Landers et al., 2017; Thordardottir et al., 2014; for review, see Ahn et al., 2022). This activity restriction, in virtue of negatively affecting engagement in cognitively stimulating activities known to protect against cognitive decline (e.g., work/volunteering, travel, socialising outside of the home; Fratiglioni et al., 2020), could itself contribute to the poorer cognitive outcomes observed in the PIGD and FOG subtypes.

Alternatively, it is possible that individuals with poor cognitive function *prior* to the onset of PD may be those most likely to present with a motor symptom profile consistent with the PIGD subtype. In particular, one possibility is that cognitive function directly influences motor symptom presentation through altered compensation mechanisms (Yogev‐Seligmann et al., 2008). Patients with better preserved executive function, for example, might develop more effective compensatory strategies for managing their motor symptoms. A patient with intact planning abilities might consciously modify their gait or posture to minimise instability, making PIGD symptoms less apparent. Those with impaired executive function might lack this compensatory ability, leading to more pronounced PIGD presentation (Amboni et al., 2013; Heremans et al., 2013; Peterson et al., 2016). Insights into causal direction and temporal order could be gained from studies comparing premorbid cognitive function across motor subtypes, or from longitudinal studies tracking changes in cognition before and after disease onset. Studies investigating the pathophysiology driving these differences in phenotype will also be critical for understanding the underlying causes of motor and cognitive impairment in PD, which may be shared or independent.

Another drawback of relying on cross-sectional data concerns the clinical implications of this review. In all included studies, patients were subtyped using motor assessments administered at roughly the same time as the cognitive assessments on which subtype groups were compared. Our results therefore demonstrate that knowledge of a patient’s motor subtype can be used to make inferences about their cognitive profile at the time of motor subtyping. For time-poor clinicians, motor subtype could be an important decision aid that directs time and resources towards patients most at risk of cognitive impairment, facilitating timely diagnosis and management of PD-MCI and PDD. It would be more impactful, however, for clinicians to be able to identify patients at risk of cognitive impairment *prior* to the onset of this impairment. This would allow for a proactive, preventative approach focused on delaying the onset of cognitive impairment, rather than a reactive approach focused on managing or slowing the progression of existing impairment. Unfortunately, the cross-sectional scope of our review prevents us from establishing whether a patient’s motor subtype at one time point can predict their cognitive profile at *subsequent* time points (i.e., months or years after subtyping).^19^ For this reason, our review should be replicated with longitudinal data. Given that the scarcity of longitudinal research contributed to our decision to restrict our review to cross-sectional data, we would also encourage further primary longitudinal investigations on the progression of cognitive symptoms in different motor subtypes of PD.

#### Other limitations

Using Hedges’ *g* as an effect size measure meant that our meta-analyses of continuous cognitive data only provided information about the *relative* difference in cognition between motor subtypes; we were unable to draw conclusions about the clinical significance of each subtype’s cognitive performance. Fortunately, our synthesis of categorical cognitive data, which included prevalence rates of MCI and dementia, did provide some insight into whether the cognitive deficits shown by motor subtype groups are clinically meaningful. On the basis of these data, we can tentatively conclude that clinically significant cognitive decline is present in all motor subtype groups, albeit to different extents. Our categorical cognitive data had their own limitations, however. When reporting our results, we have referred to dementia rather than PDD. This is because most included studies did not apply formal PDD diagnostic criteria (Emre et al., 2007), but rather used MoCA or MMSE cutoff scores to determine dementia status. Critically, both the MoCA and MMSE are screening tools that should not be used for diagnosis (Mitchell, 2017), and in many cases, the cutoff scores used were not those optimal for use with PD cohorts (Fiorenzato et al., 2024), which may have led dementia rates to be overestimated. In a few cases, DSM diagnostic criteria for dementia were applied; however, these criteria are *not* specific to the presentation of dementia observed in PD (Martinez-Martin et al., 2011a), which is known to differ from that of other dementia types, such as Alzheimer’s disease (Janvin et al., 2006; Noe et al., 2004). To ensure diagnostic accuracy, we encourage future studies to make use of the formal diagnostic criteria available for both PD-MCI (Litvan et al., 2012) and PDD (Emre et al., 2007).

We additionally used meta-analysis to evaluate differences in demographics and disease characteristics (e.g., age, LEDD) between motor subtype groups. In interpreting the results of these analyses, it should be recognised that our inclusion criteria were tailored to our primary aim of investigating cognitive differences between subtypes; as such, these analyses are not exhaustive and likely exclude hundreds of additional studies that report these data. To our knowledge, ours is the only review to date that has sought to meta-analyse motor subtype differences on these features. Thus, while exploratory, these analyses make a valuable contribution towards our understanding of PD motor subtypes.

Our ability to compare the cognitive performance of motor subtype groups within the TD/PIGD/ID and TD/AR/MX subtyping frameworks was limited by the tendency for researchers to exclude ID and MX patients from their analyses. Only 22 of 55 studies applying the TD/PIGD/ID framework and six of 22 studies applying the TD/AR/MX framework reported or provided upon request data for the ID/MX group. As a consequence, we had insufficient data to perform several moderator and confound analyses of interest for our comparisons including the MX and ID groups. Among studies that did report or provide ID/MX data, 7.1% to 58.3% of their samples (median = 12.3%) were classified as ID/MX. These subtypes therefore represent a sizable portion of the PD population that is not being adequately represented in research and whose disease profiles and trajectories are, as a result, less well understood. We would strongly encourage researchers using the TD/PIGD/ID and TD/AR/MX frameworks to be more inclusive in their approach to participant recruitment, reporting, and analysis.

It is also important to note that the pooled effect sizes for our main meta-analytic models were derived from studies that were not homogenous with respect to participants’ medication state at the time of motor and cognitive assessment. Given that dopaminergic medication affects both motor and cognitive function, medication state is consequential for both motor subtyping and cognitive assessment. Ideally, patients’ motor and cognitive function should be assessed OFF medication, to allow for the relationship between motor subtype and cognition to be elucidated in the absence of any medication effects. Unfortunately, reporting of medication status among included studies was poor, and restricting our analyses to only those studies where patients were known to be OFF medication would have severely compromised statistical power (e.g., for our TD vs. PIGD comparison, only five of 50 studies reported that participants were OFF medication for both motor and cognitive assessments). Where feasible (≥ 10 studies), we ran moderation analyses to investigate whether effect size varied with medication state at the time of motor assessment and cognitive assessment, considered separately. Owing to underreporting of medication status, however, these analyses were generally underpowered, and we did not find medication state to be a significant moderator of effect size for any subtype pair. We would strongly recommend increased transparency in the reporting of medication state at the time of both motor and cognitive assessment, so that the potential confounding effects of dopaminergic medication may be more thoroughly investigated.

Owing to the nature of the included studies, some of our review aims could not be achieved. As reported in our protocol (Child et al., 2024), we had intended to compare the results of hypothesis-driven and data-driven subtyping approaches. In hypothesis-driven subtyping, the development of subtype groups is guided by clinical expertise regarding which disease features tend to co-occur. The TD/PIGD/ID framework, for example, was based on clinicians’ observations that strong tremors often do not present alongside severe axial symptoms (e.g., postural instability, gait impairment; Marras, 2015; von Coelln & Shulman, 2016). In contrast, data-driven subtyping approaches employ statistical or machine learning methods (e.g., *k*-means clustering) to identify distinct clusters—or subgroups—of patients that emerge from a specified combination of disease features. Unfortunately, none of the studies screened for inclusion in our review reported on eligible data-driven subtyping frameworks. This is because we restricted our review to studies where subtypes were derived from motor symptoms alone, while data-driven methods tend to be applied to large datasets comprising motor *and* nonmotor variables (e.g., demographics, cognition, biomarkers; Mestre et al., 2018; van Rooden et al., 2010). Nevertheless, one of the advantages of data-driven approaches is that they attempt to capture patterns among patients purely based on the available data and so could reveal new subtypes beyond those currently hypothesised by clinicians. For this reason, we consider the application of data-driven methods to *motor* subtyping in PD to be an important avenue for future research.

We also planned to examine motor assessment type as a potential moderator of effect size, with a specific focus on comparing clinician-rated (e.g., UPDRS) and objective measures (e.g., accelerometers, force plates). As the majority of included studies classified patients into motor subtypes according to clinical judgement, often guided by rating scales, we did not have sufficient data to investigate motor assessment type as a moderator. This was unfortunate, as past research has critiqued the inherent subjectivity of clinician-rated measures, which can lead to inaccurate assessments (Kenny et al., 2024; Post et al., 2005). Use of objective measures would reduce measurement inconsistencies and potentially improve the reliability of subtype classification.

### Implications and future directions

#### Clinical implications

Parkinson’s disease is a highly heterogeneous neurodegenerative disease characterised by both motor and nonmotor symptoms, yet many patients receive “monodisciplinary” care focused on the assessment and treatment of motor symptoms (van der Marck et al., 2009). Nonmotor symptoms, including cognitive impairment, are frequently overlooked during consultations (Chaudhuri et al., 2006, 2010) despite their significant impact on patient and caregiver quality of life (Lawson et al., 2014, 2017; Morley et al., 2012). Although lack of education may contribute to some clinicians’ poor prioritisation of nonmotor symptoms, context also plays a role; operating within an overburdened healthcare system, many clinicians do not have the time and resources to review and address all motor and nonmotor symptoms (Chaudhuri et al., 2010; Ip et al., 2024). Given that motor assessment is necessary for PD diagnosis and remains a focal point of ongoing care, it is worth considering how data collected as part of existing motor assessment might be leveraged to make inferences about other disease aspects. In this way, motor assessment could be used as a decision aid that directs limited time and resources towards the assessment and treatment of nonmotor symptoms in a sub-cohort of patients most likely to experience those symptoms. Moreover, it may also enable clinicians to provide patients with more accurate prognoses regarding their expected disease trajectory.

With this in mind, our meta-analysis and systematic review specifically sought to evaluate how motor symptom heterogeneity, captured by motor subtypes, could be used to infer patients’ cognitive profile. Our review found robust evidence for the TD motor subtype being associated with better preserved cognition in PD, and for the PIGD, AR, and FOG subtypes being associated with poorer cognitive function. From a clinical perspective, patients who are classified as belonging to any of the latter subtypes should be counselled on their increased risk of PD-MCI and PDD. This may serve to improve self- and caregiver-monitoring for early signs of cognitive decline and provide opportunities for patients to engage with preventative strategies (e.g., cognitive training; Giustiniani et al., 2022; Guglietti et al., 2021) or, in future, clinical trials of neuroprotective agents. Where time and resources for cognitive evaluation are limited, patients belonging to the PIGD, AR, and FOG subtypes should be given priority.

An important caveat is that TD status should not be interpreted as conferring guaranteed or indefinite protection against cognitive impairment. Although cross-sectional data consistently show that cognition is relatively preserved in TD patients, a sizeable minority of TD patients nevertheless experience MCI or dementia (Danti et al., 2015). Moreover, longitudinal research indicates that TD subtype membership is not necessarily stable (Alves et al., 2006; Simuni et al., 2016; von Coelln et al., 2021). Patients with TD and ID both frequently convert to a PIGD subtype over time. In one study, 45.7% of patients transitioned from a TD to PIGD subtype over 4 years (Alves et al., 2006), and in another, 46% to 50% of TD patients transitioned to the PIGD or AR subtype (depending on the subtyping procedure applied) after a mean follow-up period of 6.2 years (von Coelln et al., 2021). Thus, while TD motor subtype membership is protective against cognitive decline, TD patients should still be monitored for possible transition to the PIGD subtype, and any signs of subtype transition should be accompanied by increased monitoring for signs of cognitive impairment. With the exception of newly diagnosed, de novo patients (< 12 months disease duration; Simuni et al., 2016), PIGD subtype membership seems to show much stronger stability; it is rare for patients with established PD to transition from PIGD to TD or ID (Alves et al., 2006; von Coelln et al., 2021). Given this, PIGD subtype classification likely warrants ongoing monitoring for cognitive changes.

Our comparison of global and domain-specific measures of cognition revealed that short-form global screening measures (e.g., MMSE, MoCA) perform as well as domain-specific measures, when considered holistically (i.e., a single summary measure of cognition, derived from a comprehensive neuropsychological test battery), in capturing differences in cognition between motor subtypes. This finding should provide reassurance to time-poor clinicians who rely on short-form screening measures over more comprehensive assessments. It should be recognised, however, that these global screening tools are likely insensitive to more marked deficits in specific domains, which may be relevant for informing cognitive remediation or “brain training” strategies. Although our pooled effects for individual cognitive domains lacked precision, our results trended towards all motor subtype frameworks being most sensitive to differences in executive function, indicating that cognitive training and rehabilitation strategies targeting executive dysfunction may be especially beneficial.

#### Recommendations for research

We have already outlined several recommendations for future research in earlier sections. Ultimately, a subtyping framework that captures meaningful differences in disease heterogeneity is only valuable insofar as it can be reproduced in other research cohorts and clinical settings. This reproducibility depends on clear reporting of the subtype classification procedure, something which we found to be lacking in many included studies. We therefore appeal to researchers to be more comprehensive in their reporting. In addition, we encourage a broader exploration of motor and cognitive assessment tools and of subtyping methods. There is considerable potential for objective motor measures to be incorporated into subtyping procedures, as part of either hypothesis-driven or data-driven approaches. Use of domain-specific cognitive assessments should also be prioritised, as reliance on global measures may be masking important domain differences between subtypes.

We have also identified research gaps created by the exclusion of ID and MX patients, and the paucity of research applying the FOG/nFOG subtyping framework to newly diagnosed patients. Both gaps could be addressed through more inclusive patient recruitment. Finally, there is a dearth of longitudinal research documenting differences in the progression of cognitive symptoms in motor subtype groups over time. Such research is critical for determining whether motor subtype at diagnosis can be used to predict patients’ long-term cognitive trajectories, and could lead to important insights into the temporal relationship between motor and cognitive decline in PD.

## Conclusion

Our systematic review is the first to investigate the relationship between motor subtype and cognitive function in PD across multiple motor subtyping frameworks. Whilst several reviews of motor subtyping in PD have been published, these have predominantly been expert reviews concerned with providing a broad overview of motor subtyping frameworks and their classification procedures. Our review instead offers a systematic appraisal of how these motor subtype frameworks can be used to capture heterogeneity in nonmotor symptoms, specifically cognitive impairment.

Our review found robust evidence for the TD motor subtype being a protective factor against cognitive decline in PD, and for the PIGD, AR, and FOG motor subtypes being risk factors for cognitive impairment. On the basis of our multilevel meta-analyses, gait-related subtypes appear to be the most sensitive to differences in cognition; however, further research evaluating their generalisability and application is needed. Importantly, we found little evidence for other demographics or disease characteristics accounting for the differences between motor subtype groups. This indicates that the motor subtype frameworks reviewed here are sensitive to differences in cognitive performance that cannot be captured by other dimensions of PD. In clinical settings, motor subtype classification could provide valuable information about a patient’s likelihood of cognitive decline in a time and resource efficient manner, thereby improving prognostic accuracy and facilitating personalised prevention, management, and treatment plans.

## Supplementary Information

Below is the link to the electronic supplementary material.

Supplementary file1 (PDF 84 KB)

Supplementary file2 (PDF 4289 KB)

Supplementary file3 (PDF 197 KB)

Supplementary file4 (PDF 44 KB)

Supplementary file5 (XLSX 46 KB)

## Data Availability

Data and code used in analyses can be found on OSF (https://osf.io/6ckwe/). Data from the Parkinson’s Progression Markers Initiative (PPMI) are openly available from the PPMI database (www.ppmi-info.org/access-data-specimens/download-data).

## Footnotes

1 This was a deviation from our published protocol (Child et al., 2024), where we had planned to analyse follow-up cognitive data reported in longitudinal studies. Although these data were extracted, no analyses using these data were performed. Such analyses were not deemed feasible, given the large size of our review, and the heterogenous nature of longitudinal data (follow-up length, attrition rates). Of the 123 studies included in our review, 16 studies (including the Parkinson's Progression Markers Initiative [PPMI] dataset as “one study”) reported longitudinal cognitive data (see Supplementary Material 5).

2 There was one exception to this. For two studies using different subtyping methods but with suspected overlapping samples, the corresponding author confirmed overlapping samples and subsequently advised analysing the data of one paper over another. This correspondence took place as part of our request for critical data; the corresponding author subsequently provided the critical data requested for this single study (one subtyping method) only. Because critical data were not reported in the text (or supplementary material) for the other paper, it was not possible to retain this second study in the review.

3 There was one exception to this. Santos-García et al. (2020, 2022) reported on the same sample. Although Santos-García et al. (2020) received a lower quality assessment score, this paper reported on a larger sample than Santos-García et al. (2022) and also reported data for a larger number of cognitive tasks than Santos-García et al. (2022). For these reasons, we elected to retain Santos-García et al. (2020) and exclude Santos-García et al. (2022).

4 In our published protocol, we reported that we would use Cramer’s *V* as the effect size measure for any meta-analyses evaluating the association between motor subtype and cognitive status. Unlike risk ratios, Cramer’s *V* does not capture information regarding the *direction* of the association between variables, only whether there *is* an association. Therefore, we revised our analysis plan to use risk ratios instead of Cramer’s *V*.

5 For all demographics and disease characteristics, effect sizes were calculated by using the total sample size of each motor subtype group (i.e., we assumed no missing data).

6 In this sense, traditional meta-analysis models are also “multilevel,” modelling variance across two levels (variance attributable to sampling error and between-study variance). In the literature, however, the term “multilevel meta-analysis” is usually reserved for models with three or more levels and is used in the same manner here.

7 For example, across the measures that can be extracted from the stop-signal task (e.g., stop-signal reaction time [SSRT], go reaction time, no-go reaction time, number of go errors, number of omissions on go trials), only SSRT captures the cognitive ability being measured by the task (response inhibition) and so was prioritised over other measures.

8 This was a deviation from our published protocol, where we reported that GRADE would be rated for all studies.

9 This was another deviation from our published protocol. At the time of writing the protocol, we were not aware of this adapted framework and reported that we would follow the original GRADE guidelines (Guyatt et al., 2011).

10 Note that the sum of these two values (*n* = 69) exceeds the total number of unique studies (*n* = 62) reporting the presence of cognitive impairment and/or dementia as exclusion criteria. This is because several studies reported both continuous and categorical cognitive outcome data and so appear in both counts.

11 This method is akin to the most commonly used method used to produce the TD and NTD groups; they merely differ with respect to whether the TD or PIGD group is combined with the ID group.

12 Insufficient data were available (*k* = 2) to analyse MDS-UPDRS-III scores separately.

13 Note that Zhang & colleagues (2016) did not report disease characteristics for their motor subtype groups separately, so these characteristics are for all three motor subtype groups (TD, AR, and MX) included in their sample.

14 Indeed, when this analysis was repeated with outliers removed (Supplementary Material 2, Table S29), five of the 12 data points with MDS-UPDRS-III data were identified as outliers; after their removal, SMD in UPDRS-III score (any version) was no longer a significant confound.

15 When this analysis was repeated with outliers removed (identified using residuals), cognitive class only approached statistical significance (*p* =.066; Supplementary Material 2, Table S39).

16 This effect remained when this analysis was repeated with outliers removed (identified using residuals; *p* =.018; Supplementary Material 2, Table S39).

17 Risk of bias summary plots comparing Jankovic et al. (1990) and Stebbins et al.’s (2013) subtyping methods can be found in Supplementary Material 2, Fig. S19, and risk of bias summary plots comparing studies that reported continuous and categorical cognitive data can be found in Supplementary Material 2, Fig. S20.

18 For our FOG vs. nFOG comparison, cognitive class was no longer a significant moderator when this analysis was repeated with outliers removed; as such, this significant result was likely spurious.

19 Moreover, as discussed, the temporal onset of motor subtype and cognitive symptoms in PD remains unclear. It may in fact be that signs of cognitive impairment can be leveraged to predict the subsequent emergence of motor symptoms associated with the PIGD and FOG subtypes.
