# Supplementary material for: Cognitive function in different motor subtypes of Parkinson’s disease: A systematic review and multilevel meta-analysis
Source: Cogn Affect Behav Neurosci. 2025 Dec 17;26(1):218–66. doi: 10.3758/s13415-025-01343-8 (PMC12847103; doi:10.3758/s13415-025-01343-8)
Supplement: Supplementary file 2 — Supplementary file2 (PDF 4289 KB) [file 13415_2025_1343_MOESM2_ESM.pdf]

## Supplementary Material 2


Search Strategy ........................................ 4
Table S1 ........................................ 9
Data Extraction ........................................ 10
Quality Assessment ........................................ 11
Table S2 ........................................ 12
Analysis of Parkinson's Progression Marker Initiative (PPMI) Data ........................................ 13
Results of Tremor-Dominant vs. Postural Instability Gait Disorder Analyses ........................................ 15
Table S3 ........................................ 15
Table S4 ........................................ 17
Table S5 ........................................ 18
Table S6 ........................................ 22
Figure S1 ........................................ 23
Figure S2 ........................................ 24
Figure S3 ........................................ 25
Figure S4 ........................................ 26
Results of Tremor-Dominant vs. Indeterminate Analyses ........................................ 27
Table S7 ........................................ 28
Table S8 ........................................ 30
Table S9 ........................................ 31
Table S10 ........................................ 32
Table S11 ........................................ 34
Figure S5 ........................................ 35
Table S12 ........................................ 36
Figure S6 ........................................ 39
Table S13 ........................................ 40
Results of Postural Instability Gait Disorder vs. Indeterminate Analyses ........................................ 41
Table S14 ........................................ 42
Table S15 ........................................ 44
Table S16 ........................................ 45
Table S17 ........................................ 47
Table S18 ........................................ 48

Figure S7 ... 51
Table S19 ... 53
Figure S8 ... 54
Figure S9 ... 54
Figure S10 ... 55
Results of Predominantly Tremor-Dominant vs. Predominantly Postural Instability Gait Disorder Analyses ... 56
Results of Tremor-Dominant vs. Non-Tremor-Dominant Analyses ... 58
Table S20 ... 58
Table S21 ... 60
Table S22 ... 61
Table S23 ... 63
Results of Postural Instability Gait Disorder vs. Non-Postural Instability Gait Disorder Analyses ... 64
Table S24 ... 64
Results of Tremor-Dominant vs. Akinetic-Rigid Analyses ... 66
Table S25 ... 66
Table S26 ... 68
Figure S11 ... 68
Table S27 ... 69
Table S28 ... 70
Figure S12 ... 73
Table S29 ... 74
Results of Tremor-Dominant vs. Mixed Analyses ... 75
Table S30 ... 75
Table S31 ... 77
Figure S13 ... 78
Results of Akinetic-Rigid vs. Mixed Analyses ... 79
Table S32 ... 80
Table S33 ... 82
Figure S14 ... 83
Results of Tremor vs. Bradykinesia Analyses ... 84
Table S34 ... 85
Table S35 ... 86

Figure S15 .......... 87
Results of Bradykinesia vs. No-Bradykinesia Synthesis .......... 88
Results of Tremor vs. No-Tremor Synthesis .......... 89
Results of With Facial Tremor vs. Without Facial Tremor Synthesis .......... 90
Results of Freezing of Gait vs. Non-Freezing of Gait Analyses .......... 91
Table S36 .......... 91
Figure S16 .......... 93
Table S37 .......... 94
Table S38 .......... 95
Figure S17 .......... 96
Table S39 .......... 97
Table S40 .......... 100
Table S41 .......... 103
Table S42 .......... 104
Figure S18 .......... 105
Results of Poor Gait vs. Good Gait Analyses .......... 106
Table S43 .......... 106
Results of Fallers vs. Non-Fallers Synthesis .......... 107
Risk of Bias .......... 108
Figure S19 .......... 108
Figure S20 .......... 108
Grading of Recommendations, Assessment, Development, and Evaluations (GRADE) .......... 109
Table S44 .......... 110
Reference List: All Studies Included in Review .......... 111
Supplementary Material References .......... 127

**Search Strategy**

**PubMed**

("Parkinson Disease"[mh] OR "Parkinsonian Disorders"[mh] OR Parkinson*[tiab]) AND ("Classification"[mh] OR "Cluster Analysis"[mh] OR "Algorithms*"[mh] OR "Factor Analysis, Statistical"[mh] OR "Population Characteristics"[mh] OR "Genetic Heterogeneity"[mh] OR "Phenotype"[mh] OR Subtyp* OR Cluster* OR Heterogen* OR Subgroup OR Classif* OR Typog* OR Type OR Phenotyp OR "Clinical feature" OR "Tremor-dominant" OR "Tremor dominant" OR "Akinetic-rigid" OR "Akinetic rigid" OR "PIGD" OR "Postural instability gait disorder" OR "Cluster analysis" OR "Factor analysis" OR "Data driven" OR "Data-driven") AND ("Psychomotor Performance"[mh] OR "Motor Disorders"[mh] OR "Motor Skills Disorders"[mh] OR "Tremor"[mh] OR "Hypokinesia"[mh] OR "Muscle Rigidity"[mh] OR "Gait"[mh] OR "Gait Disorders, Neurologic"[mh] OR "Postural Balance"[mh] OR "Dyskinesias"[mh] OR "Disease Progression"[mh] OR Motor OR UPDRS OR "Hoehn and Yahr" OR Tremor OR Bradykinesia OR Rigid* OR "Postural instability" OR Balance OR Gait OR Dyskinesia OR Akinesia OR "Disease duration" OR "Disease severity" OR "Disease progression") AND ("Cognition"[mh] OR "Cognition Disorders"[mh] OR "Cognitive Dysfunction"[mh] OR "Neuropsychology"[mh] OR "Neurologic Examination"[mh] OR "Neuropsychological Tests"[mh] OR "Intelligence"[mh] OR "Aptitude Tests"[mh] OR "Dementia"[mh] OR "Learning"[mh] OR "Attention"[mh] OR "Executive Function"[mh] OR "Inhibition, Psychological"[mh] OR "Visual Perception"[mh] OR "Decision Making"[mh] OR Cogniti* OR Neuropsych* OR Neurocog* OR Dementia OR "Mild cognitive impairment" OR MCI OR Intelligen* OR IQ OR Learn* OR Memory OR Attention OR "Task switching" OR "Executive Function" "Cognitive control" OR "Executive control" OR "Response inhibition" OR "Processing speed" OR "Visuospatial" OR "Reasoning" OR Verbal OR Vocabulary OR Language OR MoCA OR "Montreal Cognitive Assessment" OR MMSE OR "Mini Mental State Examination" OR CANTAB OR "Cambridge Neuropsychological Test Automated Battery" OR WCST OR "Wisconsin Card Sorting Task" OR "Wisconsin Card Sorting Test" OR IGT OR "Iowa Gambling Task" OR "Intra-Extra Dimensional Set Shift" OR "Stop signal" OR "Stop-signal" OR "Paired associates" OR "Digit span" OR N-back OR "N back" OR "Decision making" OR "Decision-making") NOT ("Animals"[mh] NOT "Humans"[mh])

Filters:
Language: English

**PsycINFO**

((exp Parkinsons Disease/ or Parkinson*.ti,ab.) and (exp Disorder Attributes/ or Taxonomies.sh. or "classification (cognitive process)".sh. or Phenotypes.sh. or Cluster Analysis.sh. or Factor Analysis.sh. or Machine Learning Algorithms.sh. or Subtyp*.tw. or Cluster*.tw. or Heterogen*.tw. or Subgroup.tw. or Classif*.tw. or Typo*.tw. or Type.tw. or Phenotyp*.tw. or "Clinical feature".tw. or Tremor-dominant.tw. or "Tremor dominant".tw. or Akinetic-rigid.tw. or "Akinetic rigid".tw. or PIGD.tw. or "Postural instability gait disorder".tw. or "Cluster analysis".tw. or "Factor analysis".tw. or "Data driven".tw. or "Data-driven".tw.) and (exp Motor Processes/ or Tremor.sh. or Bradykinesia.sh. or Equilibrium.sh. or Muscle Contractions.sh. or Motor.tw. or UPDRS.tw. or "Hoehn and Yahr".tw. or Tremor.tw. or Bradykinesia.tw. or Rigid*.tw. or "Postural instability".tw. or Balance.tw. or Gait.tw. or Dyskinesia.tw. or Akinesia.tw. or "Disease duration".tw. or "Disease severity".tw. or "Disease progression".tw.) and (exp Cognitive Processes/ or Cognitive Aging.sh. or exp Learning/ or exp Memory/ or exp Attention/ or Language.sh. or Neurocognition.sh. or exp Neurocognitive Disorders/ or exp Neuropsychological Assessment/ or exp Cognitive Assessment/ or response inhibition.sh. or visuospatial ability.sh. or reasoning.sh. or language.sh. or digit span testing.sh. or Cogniti*.tw. or Neuropsych*.tw. or Neurocog*.tw. or Dementia.tw. or Mild cognitive impairment.tw. or MCI.tw. or Intelligen*.tw. or IQ.tw. or Learn*.tw. or Memory.tw. or Attention.tw. or "Task switching".tw. or "Executive Function".tw. or "Cognitive control".tw. or "Executive control".tw. or "Response inhibition".tw. or "Processing speed".tw. or Visuospatial.tw. or Reasoning.tw. or Verbal.tw. or Vocabulary.tw. or Language.tw. or MoCA.tw. or "Montreal Cognitive Assessment".tw. or MMSE.tw. or "Mini Mental State Examination".tw. or CANTAB.tw. or "Cambridge Neuropsychological Test Automated Battery".tw. or WCST.tw. or "Wisconsin Card Sorting Task".tw. or "Wisconsin Card Sorting Test".tw. or IGT.tw. or "Iowa Gambling Task".tw. or "Intra-Extra Dimensional Set Shift".tw. or "Stop signal".tw. or "Stop-signal".tw. or "Paired associates".tw. or "Digit span".tw. or N-back.tw. or "N back".tw. or "Decision making".tw. or "Decision-making".tw.)) not (Animal not (animal and human)).po.

Filters:
Language: English

**CINAHL**

MH “Parkinson disease” OR TI Parkinson* OR AB Parkinson* AND MH “Cluster analysis+” OR MH “Factor analysis+” OR MH “Classification+” OR MH “Phenotype+” OR Subtyp* OR Cluster* OR Heterogen* OR Subgroup OR Classif* OR Typo* OR Type OR Phenotyp* OR “Clinical feature” OR “Tremor-dominant” OR “Tremor dominant” OR “Akinetic-rigid” OR “Akinetic rigid” OR “PIGD” OR “Postural instability gait disorder” OR "Cluster analysis" OR "Factor analysis" OR "Data driven" OR "Data-driven" AND MH “Psychomotor Performance+” OR MH “Gait+” OR MH “Gait Analysis” OR MH “Gait Disorders, Neurologic” OR MH “Balance, Postural” OR MH “Dyskinesias+” OR MH “Posture” OR MH “Motor Activity” OR MH “Psychomotor Disorders” OR MH “Disease Attributes+” OR MH “Severity of Illness Indices” OR Motor OR UPDRS OR “Hoehn and Yahr” OR Tremor OR Bradykinesia OR Rigid* OR “Postural instability” OR Balance OR Gait OR Dyskinesia OR Akinesia OR "Disease duration" OR "Disease severity" OR "Disease progression" AND MH “Cognition” OR MH “Intelligence” OR MH “Executive Function” OR MH “Learning+” OR MH “Attention+” OR MH “Perception+” OR MH “Language+” OR MH “Vocabulary” OR MH “Language Tests+” OR MH “Psychological Tests+” OR MH “Delirium, Dementia, Amnestic, Cognitive Disorders+” OR Cogniti* OR Neuropsych* OR Neurocog* OR Dementia OR “Mild cognitive impairment” OR MCI OR Intelligen* OR IQ OR Learn* OR Memory OR Attention OR “Task switching” OR “Executive Function” OR “Cognitive control” OR “Executive control” OR “Response inhibition” OR “Processing speed” OR Visuospatial OR Reasoning OR Verbal OR Vocabulary OR Language OR MoCA OR “Montreal Cognitive Assessment” OR MMSE OR “Mini Mental State Examination” OR CANTAB OR “Cambridge Neuropsychological Test Automated Battery” OR WCST OR “Wisconsin Card Sorting Task” OR "Wisconsin Card Sorting Test" OR IGT OR “Iowa Gambling Task” OR “Intra-Extra Dimensional Set Shift” OR “Stop signal” OR "Stop-signal" OR “Paired associates” OR “Digit span” OR N-back OR “N back” OR "Decision making" OR "Decision-making" NOT ((MH animals+ OR MH "animal studies" OR TI "animal model*") NOT (MH Human AND (MH animals+ OR MH "animal studies" OR TI "animal model*")))

Filters:

Language: English

**Scopus**

( TITLE ( parkinson* ) AND TITLE-ABS-KEY ( subtyp* OR cluster* OR heterogen* OR subgroup OR classif* OR typo* OR type OR phenotyp* OR "Clinical feature" OR "Tremor-dominant" OR "Tremor dominant" OR "Akinetic-rigid" OR "Akinetic rigid" OR "PIGD" OR "Postural instability gait disorder" OR "Cluster analysis" OR "Factor analysis" OR "Data driven" OR "Data-driven" ) AND TITLE-ABS-KEY ( motor OR updrs OR "Hoehn and Yahr" OR tremor OR bradykinesia OR rigid* OR "Postural instability" OR balance OR gait OR dyskinesia OR akinesia OR "Disease duration" OR "Disease severity" OR "Disease progression" ) AND TITLE-ABS-KEY ( cogniti* OR neuropsych* OR neurocog* OR dementia OR "Mild cognitive impairment" OR mci OR intelligen* OR iq OR learn* OR memory OR attention OR "Task switching" OR "Executive Function" OR "Cognitive control" OR "Executive control" OR "Response inhibition" OR "Processing speed" OR visuospatial OR reasoning OR verbal OR vocabulary OR language OR moca OR "Montreal Cognitive Assessment" OR mmse OR "Mini Mental State Examination" OR cantab OR "Cambridge Neuropsychological Test Automated Battery" OR wcst OR "Wisconsin Card Sorting Task" OR "Wisconsin Card Sorting Test" OR igt OR "Iowa Gambling Task" OR "Intra-Extra Dimensional Set Shift" OR "Stop signal" OR "Stop-signal" OR "Paired associates" OR "Digit span" OR n-back OR "N back" OR "Decision making" OR "Decision-making" ) )

Filters:

Language: English

Document Type: Article

**Web of Science**

(((TS=("Parkinson* Disease")) AND ALL=(Subtyp* OR Cluster* OR Heterogen* OR Subgroup OR Classif* OR Typo* OR Type OR Phenotyp* OR "Clinical feature" OR "Tremor-dominant" OR "Tremor dominant" OR "Akinetic-rigid" OR "Akinetic rigid" OR "PIGD" OR "Postural instability gait disorder" OR "Cluster analysis" or "Factor analysis" OR "Data driven" OR "Data-driven")) AND ALL=(Motor OR UPDRS OR "Hoehn and Yahr" OR Tremor OR Bradykinesia OR Rigid* OR "Postural instability" OR Balance OR Gait OR Dyskinesia OR Akinesia OR "Disease duration" OR "Disease severity" OR "Disease progression")) AND ALL=(Cogniti* OR Neuropsych* OR Neurocog* OR Dementia OR "Mild cognitive impairment" OR MCI OR Intelligen* OR IQ OR Learn* OR Memory OR

Attention OR "Task switching" OR "Executive Function" OR "Cognitive control" OR "Executive control" OR "Response inhibition" OR  "Processing speed" OR Visuospatial OR Reasoning OR Verbal OR Vocabulary OR Language OR MoCA OR "Montreal Cognitive Assessment" OR MMSE OR "Mini Mental State Examination" OR CANTAB OR "Cambridge Neuropsychological Test Automated Battery" OR WCST OR "Wisconsin Card Sorting Task" OR "Wisconsin Card Sorting Test" OR IGT OR "Iowa Gambling Task" OR "Intra-Extra Dimensional Set Shift" OR "Stop signal" OR Stop-signal OR "Paired associates" OR "Digit span" OR N-back OR "N back" OR "Decision making" OR Decision-making)

Filters:
Language: English
Document Type: Article

**Note**

All database searches were run on 20 July 2022 and then re-run (before the commencement of data synthesis) on 23 January 2024.

When the search was re-run:

- **PubMed**, **PsycINFO**, and **Scopus** did not allow the date range to be restricted to a specific date (i.e., 21 July 2022 – current), but only to a given year. Therefore, these searches were re-run with the added restriction of date 2022-2024/current.
- **CINAHL** did not allow the date range to be restricted to a specific date (i.e., 21 July 2022 – current), but only to a given month. Therefore, this search was re-run with the added restriction of date July 2022 – January 2024.
- **Web of Science** did allow an exact date range to be specified, so this search was re-run with the added timespan restriction of 2022-07-22 to 2024-01-23 (Publication Date).

**Table S1**

*Title and Abstract and Full-Text Screening Interrater Agreement Statistics*

| | Title and Abstract Screening | | | Full-Text | | |
|---|---|---|---|---|---|---|
| | Number of records screened | Proportionate Agreement | Kappa ($\kappa$) | Number of records screened | Proportionate Agreement | Kappa ($\kappa$) |
| **Reviewer** | | BC | | | BC | |
| IS | 7140 | .95 | .48 | 127 | .88 | .66 |
| RDS | 2295 | .93 | .53 | 197 | .89 | .79 |
| BE | - | - | - | 91 | .86 | .72 |
| AM | - | - | - | 61 | .84 | .58 |
| IB | - | - | - | 13 | 1 | 1 |

*Note.* AM = Angus McNamara; BC = Brittany Child; BE = Benjamin Ellul; IB = Irina Baetu; IS = Isaac Saywell; RDS = Robyn da Silva. AM, BE, and IB only served as reviewers at the full-text screening stage.

## Data Extraction

A custom data extraction form comprising 127 items[1] (see Supplementary Material 3) was developed to extract study information pertaining to sample size and characteristics, subtyping methods, and cognitive outcome data. We extracted information on psychiatric comorbidities (anxiety, depression), *post hoc* motor data[2], and longitudinal cognitive data from all studies where these data were reported; however, we did not analyse these data for feasibility reasons. For information on which included studies reported *post hoc* motor data (*n* = 50) and longitudinal cognitive data (*n* = 16), see Supplementary Material 5.

When cognitive outcome data (means and standard deviations or frequencies/proportions, test statistics and *p*-values, or effect sizes) were not reported for motor subtype groups in either the main text or supplementary materials, data were requested from corresponding authors via email. If other desirable but non-essential data were missing (e.g., demographics or disease characteristics, such as disease duration), these were also requested[3]. If cognitive outcome data were reported in full, we did not contact authors to request any missing non-essential data. Up to two attempts at contact were made; if the author did not respond within a 1-week period from the date of the second email being sent[4], or if the requested data were unable to be provided, attempts were made to estimate the values needed to compute an effect size for at least one cognitive measure (see Section 2.7.2 'Effect Size Calculation'). If this was not possible, the study was excluded.

---

[1] An additional two items were added after the publication of our protocol, which included a 125-item data extraction template: one item to extract education (in years) from each motor subtype group separately; and one item to extract the levodopa equivalent daily dose (LEDD) for each motor subtype group separately.
[2] Here, we use '*post hoc*' to refer to any additional objective or clinician-rated motor assessment(s) on which motor subtype groups were compared which were *not* used to classify participants into motor subtype groups. For example, Ehm et al. (2019) classified participants into 'good tandem gait' and 'poor tandem gait' groups using a tandem gait test and then subsequently compared these groups on the freezing item and axial subscore of the Movement Disorder Society Unified Parkinson's Disease Rating Scale (MDS-UPDRS).
[3] Where mean age (at time of study participation) and mean age at disease onset were both reported but mean disease duration was not reported (and any data requests were unsuccessful), we calculated mean disease duration as mean age minus mean age at disease onset. Where mean disease duration and mean age (at time of study participation) were both reported but mean age at disease onset was not reported (and any data requests were unsuccessful), we calculated mean age at onset as mean age minus mean disease duration.
[4] Due to time constraints, we deviated from our protocol, where we reported that we would make up to three attempts at author contact, allowing up to 2 weeks for a response from the date of third email. Note that we accommodated all deadline extension requests. One author (without an extension) responded to our request after the deadline; we included these data in our meta-analysis.

**Quality Assessment**

We adapted from Hayden et al.'s (2013) Quality in Prognosis Studies (QUIPS) tool, which was designed to evaluate validity and bias in studies of prognostic factors. We customised this tool to better suit our review by modifying its 'prognostic factor' and 'outcome measurement' domains to appraise the quality of motor and cognitive measures in our included studies. In addition, we replaced the QUIPS' 'study confounding' domain with a single item and, as we did not analyse any longitudinal data, removed the 'attrition' domain[5]. Within each domain, we altered items to be specific to our review and incorporated several items adapted from Mestre and colleagues' (2021) methodological quality tool, which was specifically developed for appraising PD subtyping studies. We also adopted a numeric scoring approach to increase sensitivity and to allow for greater weight to be given to items considered to be of greater importance. After piloting this tool, we identified cut-off scores for classifying a study's risk of bias as low, moderate, or high for each domain and overall. Where two studies used the same (or overlapping) sample and were collapsed into a single study for the purposes of our review, the quality assessment tool was applied to both studies separately. An average quality assessment score was then calculated for each domain, and these were used to determine a risk of bias judgement (low/moderate/high) for each domain and overall. Similarly, where one study reported multiple subtyping methods eligible for inclusion in our review, the quality assessment tool was completed for each subtyping method separately and the above procedure was used to determine an average score for each domain, followed by a risk of bias judgement for each domain and overall. All quality assessment scores (including averages, where applicable) can be found on the Open Science Framework (OSF; https://osf.io/6ckwe/).

[5] In our published protocol (Child et al., 2024), we reported renaming the 'attrition' domain to 'use of follow-up data' and adapted its items to better suit our review. However, given that we subsequently chose not to analyse any longitudinal cognitive data, we removed this domain from our quality assessment tool altogether.

**Table S2**

*Intraclass Correlations for Quality Assessment*

| Domain | Proportionate Agreement | ICC |
|---|---|---|
| 1. Recruitment and Sample Characteristics | .89 | .57 |
| 2. Motor Function Measurement | .91 | .81 |
| 3. Cognitive Function Measurement | .93 | .87 |
| 4. Statistical Analysis and Reporting | .69 | .65 |
| Overall | .83 | .73 |

*Note.* ICC = intra-class correlation. Quality assessment was completed by two reviewers (BC and IS) on a minimum 20% of included studies, selected at random.

**Analysis of Parkinson's Progression Marker Initiative (PPMI) Data**

The open-access Parkinson's Progression Marker Initiative (PPMI) data repository comprises clinical, imaging, genetic, and biomarker data collected as part of a large longitudinal study investigating Parkinson's disease (PD; Marek et al., 2018). These data have been analysed in hundreds of publications, with research teams applying different participant inclusion criteria and varied analysis techniques to answer specific research questions of interest. Twelve studies analysing PPMI data were eligible for inclusion in our review; given the between-study heterogeneity in participant selection and analysed variables, and the open-access nature of the PPMI repository, we chose to exclude all published PPMI studies and instead analyse the PPMI dataset ourselves applying data filtering methods consistent with our review's inclusion criteria.

Data used in the preparation of this article were obtained on 25 June 2024 from the PPMI database (www.ppmi-info.org/access-data-specimens/download-data), RRID:SCR 006431.

We retained all participants who were *de novo* at baseline and for whom sufficient baseline Movement Disorder Society Unified Parkinson's Disease Rating Scale (MDS-UPDRS; Goetz et al., 2008) data were available to calculate tremor and PIGD scores according to Stebbin et al.'s (2013) subtyping method. We then applied Stebbins et al.'s (2013) subtyping method as per its traditional usage to allocate each participant to a tremor-dominant (TD), postural instability and gait disorder (PIGD), or indeterminate (ID) group. In addition, consistent with other researchers' variations on Stebbins et al.'s (2013) method (e.g., Pelicioni et al., 2021; Yu et al., 2022), we applied two further subtyping classifications: one where participants were classified as either TD or non-TD (NTD; in which the latter group is formed by collapsing the PIGD and ID groups); and one where participants were classified as either PIGD or non-PIGD (in which the latter group is formed by collapsing the TD and ID groups).

For each subtype group (across all three subtyping methods), we calculated means and SDs for all available cognitive tasks administered at baseline (or, for the Montreal Cognitive Assessment [MoCA], at screening or baseline). For cognitive status, the PPMI dataset provides a normal cognition (NC), mild cognitive impairment (PD-MCI), or PD dementia (PDD) classification based on clinical judgement; however, we instead classified participants as either NC or PD-MCI by applying the Movement Disorder Society Task Force's (Litvan et al., 2012) Level II diagnostic criteria for PD-MCI using the task cut-off

scores reported by PPMI (Coffey et al., 2020). Summary statistics (means and standard deviations) for each motor subtype group were also calculated for the following demographics and disease characteristics: age at baseline, proportion of men, disease duration (converted to years), age at disease onset, years of education, MDS-UPDRS-III score, MDS-UPDRS tremor subscore, MDS-UPDRS PIGD subscore. Effect size estimates were then computed using the methods described in the main text (Section 2.7.2).

PPMI – a public-private partnership – is funded by the Michael J. Fox Foundation for Parkinson's Research and funding partners, including 4D Pharma, Abbvie, AcureX, Allergan, Amathus Therapeutics, Aligning Science Across Parkinson's, AskBio, Avid Radiopharmaceuticals, BIAL, BioArctic, Biogen, Biohaven, BioLegend, BlueRock Therapeutics, Bristol-Myers Squibb, Calico Labs, Capsida Biotherapeutics, Celgene, Cerevel Therapeutics, Coave Therapeutics, DaCapo Brainscience, Denali, Edmond J. Safra Foundation, Eli Lilly, Gain Therapeutics, GE HealthCare, Genentech, GSK, Golub Capital, Handl Therapeutics, Insitro, Jazz Pharmaceuticals, Johnson & Johnson Innovative Medicine, Lundbeck, Merck, Meso Scale Discovery, Mission Therapeutics, Neurocrine Biosciences, Neuron23, Neuropore, Pfizer, Piramal, Prevail Therapeutics, Roche, Sanofi, Servier, Sun Pharma Advanced Research Company, Takeda, Teva, UCB, Vanqua Bio, Verily, Voyager Therapeutics, the Weston Family Foundation and Yumanity Therapeutics. For up-to-date information on the study, visit www.ppmi-info.org.

**Results of Tremor-Dominant vs. Postural Instability Gait Disorder Analyses**

**Table S3**

*Meta-Analyses Comparing Tremor-Dominant (TD) and Postural Instability Gait Disorder (PIGD) Motor Subtypes on Demographics and Disease Characteristics*

| Characteristic | *k* (*o*) | Pooled effect size (95% CI) | *p* | $I^2$ (%) | *Q* (*p*) | Egger's test (*p*) |
|---|---|---|---|---|---|---|
| **Age** | | | | | | |
| *All studies* | 47 (9232) | -0.20 (-0.29, -0.12) | **< .001** | 56.0 | 104.58 (**< .001**) | .836 |
| *With outliers removed* | 43 (7935) | -0.18 (-0.24, -0.12) | **< .001** | 20.0 | 52.53 (.128) | .202 |
| **Gender (proportion of men)*** | | | | | | |
| *All studies* | 43 (8705) | 1.05 (1.00, 1.10) | **.049** | 25.7 | 56.55 (.066) | .791 |
| **Years of education*** | | | | | | |
| *All studies* | 27 (4103) | 0.10 (0.01, 0.20) | **.032** | 26.0 | 35.12 (.109) | .321 |
| **Disease duration** | | | | | | |
| *All studies* | 43 (8661) | -0.17 (-0.26, -0.08) | **< .001** | 61.0 | 107.74 (**< .001**) | .222 |
| *With outliers removed* | 40 (8325) | -0.16 (-0.23, -0.09) | **< .001** | 42.6 | 67.89 (**.003**) | .180 |
| **Age at onset** | | | | | | |
| *All studies* | 20 (5925) | -0.17 (-0.34, -0.00) | **.049** | 72.4 | 68.95 (**< .001**) | .650 |
| *With outliers removed* | 18 (5743) | -0.16 (-0.27, -0.06) | **.005** | 48.6 | 33.09 (**.011**) | .549 |
| **LEDD** | | | | | | |
| *All studies* | 30 (6517) | -0.41 (-0.49, -0.32) | **< .001** | 48.9% | 56.77 (**.002**) | .210 |

| | | | | | | |
|---|---|---|---|---|---|---|
| *With outliers removed* | 29 (5997) | -0.45 (-0.53, -0.38) | < **.001** | 21.4 | 35.63 (.152) | **.023** |
| **UPDRS-III total score (original version)** | | | | | | |
| *All studies* | 26 (4580) | -0.34 (-0.49, -0.19) | < **.001** | 70.5 | 84.75 (< **.001**) | .849 |
| *With outliers removed* | 22 (4040) | -0.34 (-0.45, -0.24) | < **.001** | 42.5 | 36.60 (**.019**) | .458 |
| **MDS-UPDRS-III total score** | | | | | | |
| *All studies* | 20 (4575) | -0.11 (-0.25, 0.03) | .118 | 57.9 | 45.13 (< **.001**) | .257 |
| *With outliers removed* | 19 (3500) | -0.08 (-0.21, 0.05) | .203 | 42.0 | 31.01 (**.029**) | .576 |
| **UPDRS-III total score (any version)** | | | | | | |
| *All studies* | 46 (9155) | -0.24 (-0.34, -0.13) | < **.001** | 69.0 | 145.04 (< **.001**) | .955 |
| *With outliers removed* | 40 (8073) | -0.23 (-0.32, -0.15) | < **.001** | 49.4 | 77.01 (< **.001**) | .798 |

*Note.* CI = confidence interval; *k* = number of studies/effect sizes; LEDD = levodopa equivalent daily dose; MDS-UPDRS-III = Movement Disorder Society Unified Parkinson's Disease Rating Scale Part III; *o* = pooled sample size (both groups); UPDRS-III = Unified Parkinson's Disease Rating Scale Part III. Pooled effect size is Hedges' *g* for all variables except gender, which is risk ratio. For Hedges' *g*, negative pooled effect sizes reflect higher values (i.e., older age, longer disease duration) in the postural instability gait disorder (PIGD) group relative to the tremor-dominant group; for risk ratio, values > 1 indicate that tremor-dominant patients have a greater risk of being men relative to postural instability gait disorder (PIGD) patients. $I^2$ is for between-study variance. Egger's test was conducted only when $k \geq 10$ and using Pustejovsky and Rodgers' (2019) revised method. *Results for model with outliers removed not reported as no outliers detected. Significant *p*-values (< .05) are highlighted in bold.

**Table S4**

*Results of Continuous Moderator Analyses for Tremor-Dominant (TD) and Postural Instability Gait Disorder (PIGD) Motor Subtype Groups (Outliers Removed – Residuals Approach)*

| **Moderator** | ***n*** | ***k*** | ***β* (95% CI)** | ***p*** | **Change in Between-Study $I^2$** |
|---|---|---|---|---|---|
| Sample size | 50 | 242 | 0.00 (-0.00, 0.00) | .928 | -0.49 |
| Publication year | 50 | 242 | 0.01 (-0.01, 0.02) | .390 | -0.57 |
| Pooled mean age | 47 | 218 | 0.03 (0.01, 0.04) | **.003** | 10.58 |
| Pooled proportion of men | 44 | 189 | -0.17 (-1.01, 0.67) | .693 | -1.26 |
| Pooled mean years of education | 28 | 169 | -0.02 (-0.05, 0.02) | .330 | -1.33 |
| Pooled mean disease duration | 47 | 209 | -0.00 (-0.03, 0.02) | .721 | -1.24 |
| Pooled mean age at onset | 48 | 223 | 0.02 (0.00, 0.03) | **.013** | 5.90 |
| Pooled mean LEDD | 30 | 152 | -0.00 (-0.00, 0.00) | .584 | -1.92 |
| Pooled mean UPDRS-III (original) | 26 | 126 | 0.01 (-0.00, 0.02) | .297 | 0.17 |
| Pooled mean MDS-UPDRS-III | 20 | 94 | 0.01 (-0.01, 0.03) | .521 | -0.17 |

*Note*. CI = confidence interval; *k* = number of effect sizes; LEDD = levodopa equivalent daily dose; MDS-UPDRS-III = Movement Disorder Society Unified Parkinson’s Disease Rating Scale Part III; *n* = number of unique studies; UPDRS-III = Unified Parkinson’s Disease Rating Scale Part III; $\beta$ = regression coefficient. For $\beta$, positive values reflect a positive association between the moderator and effect size (i.e., larger values of the moderator are associated with larger effect sizes, reflecting better cognitive performance in the tremor-dominant [TD] group relative to the postural instability gait disorder [PIGD] group). Change in $I^2$ is the difference in between-study $I^2$ for the model with and without the moderator (larger positive values correspond to greater between-study variance being accounted for by the moderator; negative values suggest that model fit worsened as a consequence of including the moderator). Significant *p*-values (< .05) are highlighted in bold.

**Table S5**

*Results of Categorical Moderator Analyses for Tremor-Dominant (TD) and Postural Instability Gait Disorder (PIGD) Motor Subtype Groups (Outliers Removed – Residuals Approach)*

| **Moderator** | ***n*** | ***k*** | **Pooled Hedges' *g* (95% CI)** | ***β* (95% CI)** | ***p*** | ***Q* (*p*)** | **ToM (*p*)** |
|---|---|---|---|---|---|---|---|
| **Subtype Method** | | | | | | 407.69 (< **.001**) | 2.03 (.133) |
| *Jankovic** | 26 | 110 | 0.18 (0.10, 0.26) | | **< .001** | | |
| *Stebbins* | 21 | 106 | 0.29 (0.17, 0.41) | 0.11 (-0.04, 0.26) | .149 | | |
| *Other* | 3 | 26 | 0.35 (0.17, 0.54) | 0.17 (-0.03, 0.38) | .094 | | |
| **Cognitive Class** | | | | | | 409.84 (< **.001**) | 0.04 (.844) |
| *Global** | 47 | 67 | 0.24 (0.17, 0.31) | | **< .001** | | |
| *Specific* | 24 | 175 | 0.23 (0.14, 0.32) | -0.01 (-0.09, 0.07) | .844 | | |
| **Cognitive Domain** | | | | | | 371.97 (<**.001**) | 0.73 (.782) |
| *Global Cognitive Function** | 47 | 67 | 0.23 (0.17, 0.30) | | **< .001** | | |
| *Executive Function - Attention* | 5 | 9 | 0.24 (0.03, 0.45) | 0.01 (-0.19, 0.20) | .955 | | |
| *Executive Function – Cognitive Flexibility* | 11 | 15 | 0.25 (0.10, 0.40) | 0.02 (-0.12, 0.15) | .809 | | |
| *Executive Function – Cognitive Inhibition* | 6 | 6 | 0.15 (-0.03, 0.33) | -0.08 (-0.26, 0.10) | .368 | | |
| *Executive Function – Response Inhibition* | 3 | 3 | 0.41 (0.11, 0.72) | 0.18 (-0.12, 0.48) | .230 | | |

| | | | | | |
|---|---|---|---|---|---|
| *Executive Function – Working Memory* | 16 | 23 | 0.27 (0.11, 0.42) | 0.03 (-0.11, 0.17) | .650 |
| *Higher-Order Fluid Abilities - Planning* | 5 | 5 | 0.28 (0.09, 0.47) | 0.04 (-0.15, 0.24) | .645 |
| *Language* | 12 | 19 | 0.24 (0.11, 0.36) | 0.00 (-0.11, 0.12) | .948 |
| *LTM/Learning – Lexical* | 8 | 8 | 0.10 (-0.03, 0.23) | -0.13 (-0.27, 0.01) | .062 |
| *LTM/Learning - Semantic* | 7 | 7 | 0.18 (0.03, 0.32) | -0.05 (-0.20, 0.09) | .457 |
| *LTM/Learning - Visuospatial* | 4 | 4 | 0.11 (-0.09, 0.31) | -0.13 (-0.33, 0.08) | .223 |
| *STM – Lexical* | 8 | 8 | 0.17 (0.02, 0.32) | -0.06 (-0.21, 0.08) | .384 |
| *STM – Numeric* | 5 | 5 | 0.22 (0.04, 0.41) | -0.01 (-0.20, 0.17) | .900 |
| *STM – Visuospatial* | 5 | 10 | 0.17 (0.02, 0.31) | -0.07 (-0.22, 0.08) | .384 |
| *STM – Other* | 3 | 4 | 0.29 (0.03, 0.54) | 0.05 (-0.21, 0.31) | .683 |
| *Processing Speed* | 16 | 25 | 0.24 (0.08, 0.40) | 0.00 (-0.14, 0.15) | .953 |
| *Visuospatial Abilities – Construction* | 3 | 4 | 0.12 (-0.08, 0.33) | -0.11 (-0.32, 0.10) | .292 |
| *Visuospatial Abilities – Perception* | 6 | 6 | 0.23 (0.02, 0.45) | -0.00 (-0.21, 0.21) | 1.000 |
| *Visuospatial Abilities – Reasoning* | 3 | 4 | 0.34 (0.14, 0.54) | 0.11 (-0.10, 0.32) | .307 |

| | | | | | | | |
|---|---|---|---|---|---|---|---|
| **Medication Status for Cognitive Assessment(s)** | | | | | | 262.96 (< **.001**) | 1.41 (.241) |
| *De Novo*[*] | 8 | 53 | 0.23 (0.10, 0.35) | | **< .001** | | |
| *ON* | 10 | 79 | 0.12 (-0.05, 0.28) | -0.11 (-0.31, 0.10) | .297 | | |
| *OFF* | 5 | 34 | 0.37 (0.19, 0.54) | 0.14 (-0.07, 0.35) | .198 | | |
| *ON or OFF* | 1 | 2 | 0.19 (-0.14, 0.53) | -0.03 (-0.39, 0.33) | .857 | | |
| **Medication Status for Motor Assessment(s)** | | | | | | 312.10 (< **.001**) | 0.625 (.600) |
| *De Novo*[*] | 8 | 53 | 0.23 (0.10, 0.35) | | **< .001** | | |
| *ON* | 13 | 66 | 0.33 (0.14, 0.52) | 0.10 (-0.12, 0.33) | .371 | | |
| *OFF* | 15 | 62 | 0.23 (0.10, 0.36) | 0.00 (-0.17, 0.18) | .963 | | |
| *ON or OFF* | 8 | 4 | 0.17 (0.05, 0.30) | -0.05 (-0.23, 0.13) | .561 | | |
| **Cognitive Impairment/Dementia as Exclusion Criterion** | | | | | | 411.36 (< **.001**) | 0.11 (.744) |
| *No*[*] | 27 | 115 | 0.24 (0.17, 0.32) | | **< .001** | | |
| *Yes* | 23 | 127 | 0.22 (0.10, 0.34) | -0.02 (-0.17, 0.12) | .744 | | |
| **Motor Subtypes Compared on Cognition in Paper** | | | | | | 386.14 (< **.001**) | 0.99 (.320) |
| *No*[*] | 15 | 31 | 0.29 (0.14, 0.44) | | **< .001** | | |
| *Yes* | 41 | 198 | 0.22 (0.15, 0.29) | -0.07 (-0.22, 0.07) | .320 | | |

| | | | | | | | |
|---|---|---|---|---|---|---|---|
| **Overall Risk of Bias** | | | | | | 408.98 (< **.001**) | 0.19 (.829) |
| *High*[*] | 20 | 72 | 0.26 (0.10, 0.42) | | **.002** | | |
| *Moderate* | 19 | 98 | 0.21 (0.14, 0.29) | -0.05 (-0.22, 0.13) | .608 | | |
| *Low* | 11 | 72 | 0.24 (0.12, 0.37) | -0.01 (-0.22, 0.19) | .897 | | |

*Note*. CI = confidence interval; *k* = number of effect sizes; LTM = long-term memory; *n* = number of unique studies; STM = short-term memory; ToM = Test of Moderators; $\beta$ = regression coefficient. [*] denotes reference category for model. The $\beta$ coefficients and corresponding *p*-values indicate whether the pooled effect size for that level of the moderator differs significantly from the pooled effect size of the reference category; for the reference category, these values indicate whether the pooled effect size differs significantly from zero. For Hedges' *g*, values > 0 indicate poorer cognitive performance among postural instability gait disorder (PIGD) patients relative to tremor-dominant (TD) patients. Significant *p*-values (< .05) are highlighted in bold.

**Table S6**

*Results of Confound Analyses for Tremor-Dominant (TD) and Postural Instability Gait Disorder (PIGD) Motor Subtype Groups (Outliers Removed – Residuals Approach)*

| Confound | *n* | *k* | ***β*** **(95% CI)** | *p* | **Change in Between-Study** $I^2$ |
|---|---|---|---|---|---|
| SMD age | 47 | 218 | -0.48 (-0.67, -0.29) | **< .001** | 23.75 |
| Difference in proportion of men | 44 | 189 | 0.03 (-0.56, 0.62) | .929 | -1.32 |
| SMD years of education | 28 | 169 | 0.36 (0.07, 0.65) | **.016** | 7.44 |
| SMD disease duration | 44 | 199 | -0.13 (-0.35, 0.10) | .257 | 1.23 |
| SMD age at onset | 20 | 86 | -0.55 (-0.80, -0.29) | **< .001** | 26.73 |
| SMD LEDD | 30 | 152 | -0.22 (-0.53, 0.09) | .161 | 3.64 |
| SMD UPDRS-III (original) | 26 | 126 | -0.37 (-0.54, -0.19) | **< .001** | 30.40 |
| SMD MDS-UPDRS-III | 20 | 94 | -0.20 (-0.60, 0.21) | .333 | 26.52 |
| SMD UPDRS-III (any version) | 46 | 220 | -0.19 (-0.39, 0.00) | .051 | 4.44 |

*Note*. CI = confidence interval; *k* = number of effect sizes; LEDD = levodopa equivalent daily dose; MDS-UPDRS-III = Movement Disorder Society Unified Parkinson's Disease Rating Scale Part III; *n* = number of unique studies; UPDRS-III = Unified Parkinson's Disease Rating Scale Part III; SMD = standardised mean difference (Hedges' *g*); $\beta$ = regression coefficient. For $\beta$, positive values reflect a positive association between the confound (moderator) and effect size (i.e., larger values of the confound [moderator] are associated with larger effect sizes, reflecting better cognitive performance in the tremor-dominant [TD] group relative to the postural instability gait disorder [PIGD] group). Change in $I^2$ is the difference in between-study $I^2$ for the model with and without the confound (moderator; larger positive values correspond to greater between-study variance being accounted for by the confound [moderator]; negative values suggest that model fit worsened as a consequence of including the confound [moderator]). Significant *p*-values (< .05) are highlighted in bold.

**Figure S1**

*Relationships Between Standardised Mean Difference (SMD) in Years of Education and Effect Size for Cognitive Difference Between Tremor-Dominant (TD) and Postural Instability Gait Disorder (PIGD) Motor Subtype Groups*

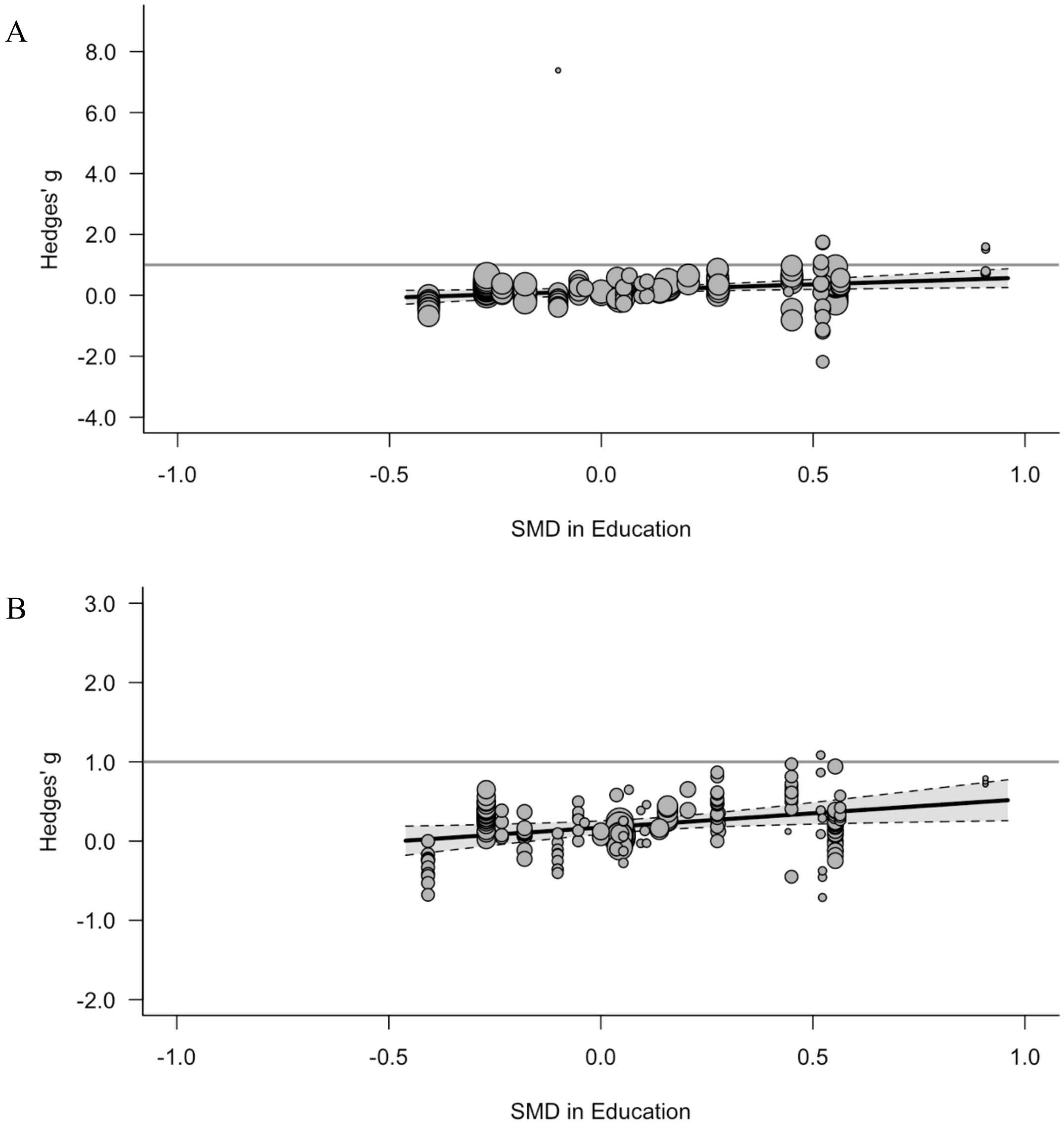


*Note.* A. No outliers removed. B. Outliers removed using residuals approach. For Hedges' *g*, values > 0 indicate better cognitive performance among tremor-dominant (TD) patients relative to postural instability gait disorder (PIGD) patients.

**Figure S2**

*Relationship Between Standardised Mean Difference (SMD) in Age at Disease Onset and Effect Size for Cognitive Difference Between Tremor-Dominant (TD) and Postural Instability Gait Disorder (PIGD) Motor Subtype Groups*

A

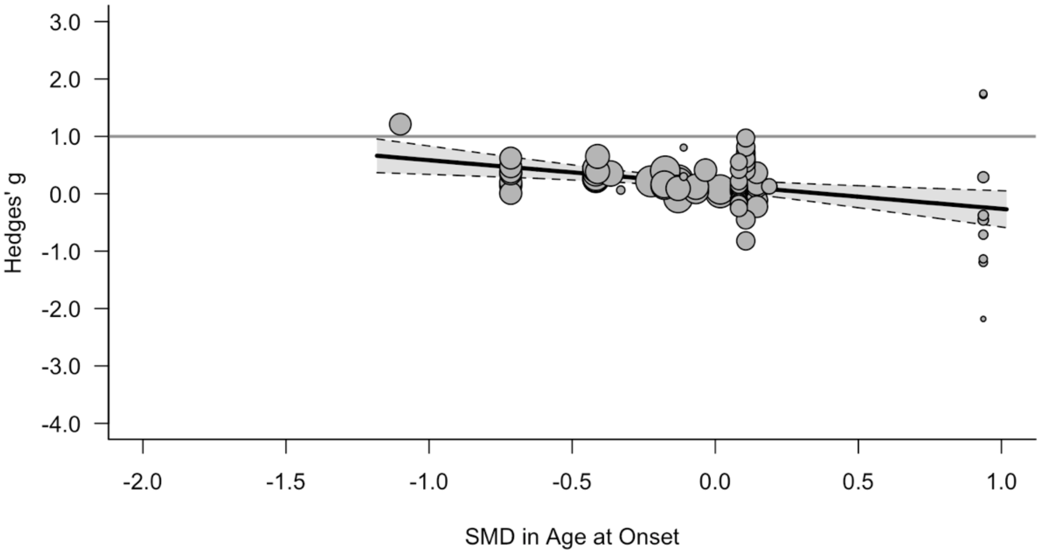


B

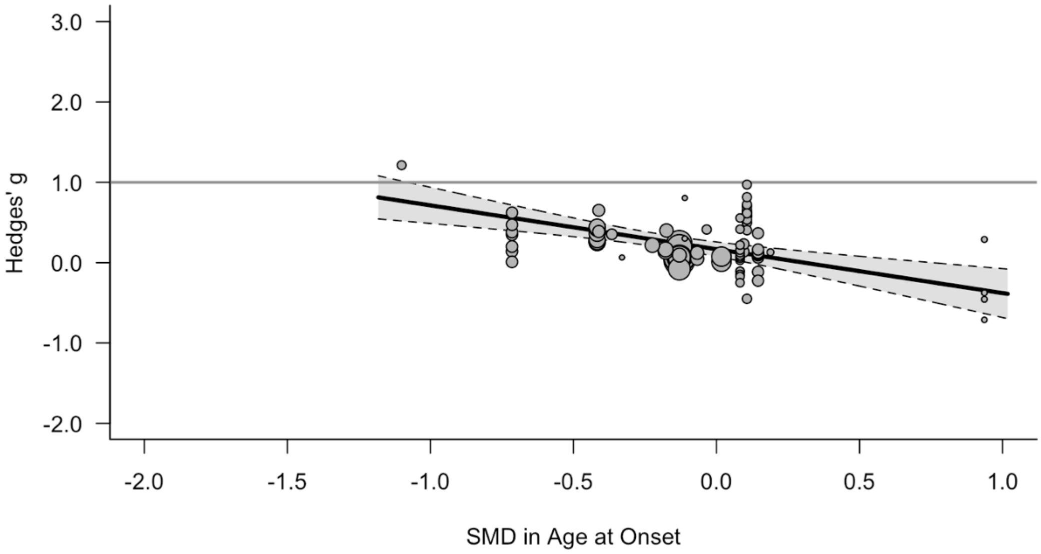


*Note.* A. No outliers removed. B. Outliers removed using residuals approach. For Hedges' *g*, values > 0 indicate better cognitive performance among tremor-dominant (TD) patients relative to postural instability gait disorder (PIGD) patients.

**Figure S3**

*Relationship Between Standardised Mean Difference (SMD) in Age and Effect Size for Cognitive Difference Between Tremor-Dominant (TD) and Postural Instability Gait Disorder (PIGD) Motor Subtype Groups (Outliers Removed – Residuals Approach)*

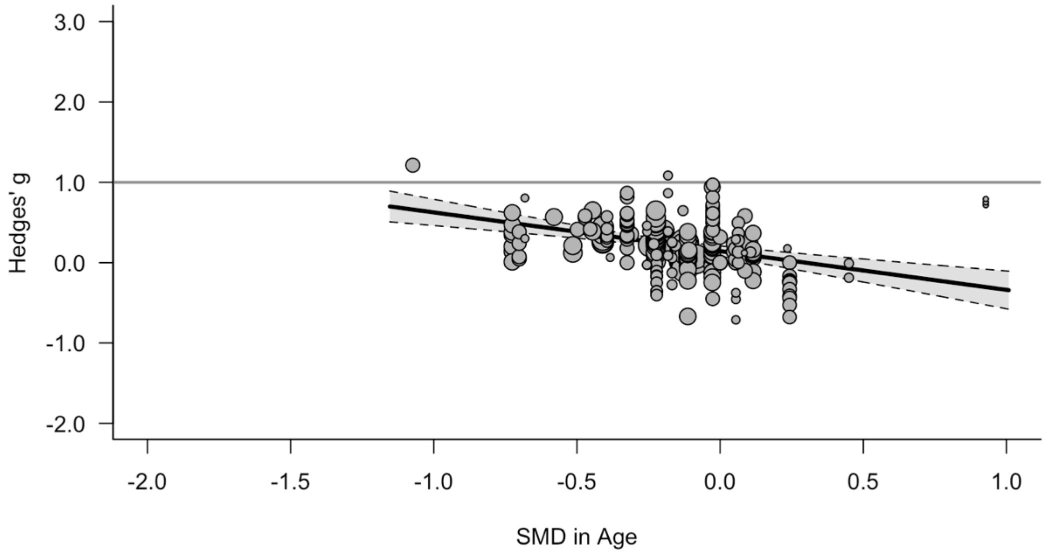


*Note*. SMD = standardised mean difference. For Hedges' *g*, values > 0 indicate better cognitive performance among tremor-dominant (TD) patients relative to postural instability gait disorder (PIGD) patients.

**Figure S4**

*Relationship Between Standardised Mean Difference (SMD) in Unified Parkinson's Disease Rating Scale (UPDRS) Part III Scores (Original Version) and Effect Size for Cognitive Difference Between Tremor-Dominant (TD) and Postural Instability Gait Disorder (PIGD) Motor Subtype Groups (Outliers Removed – Residuals Approach)*

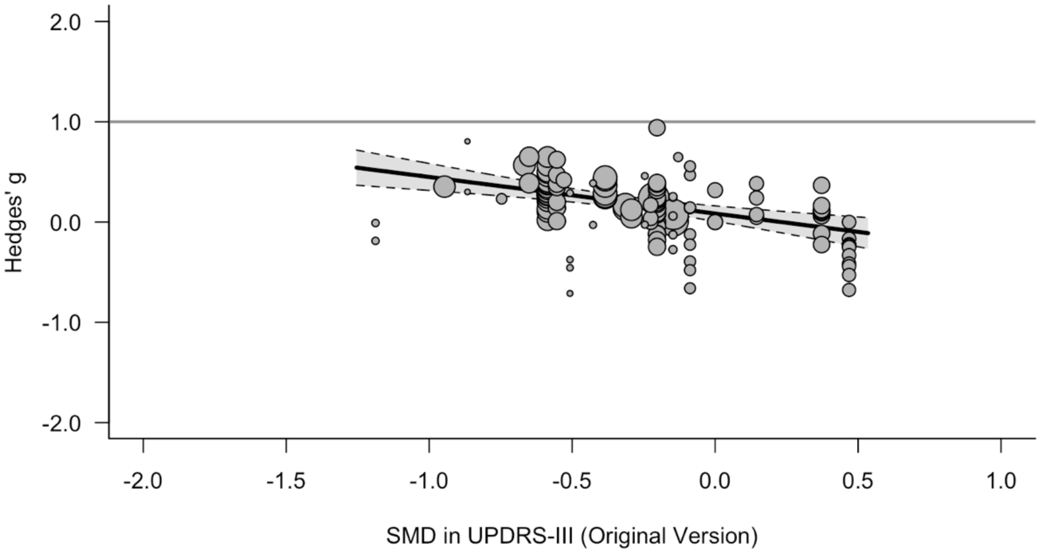


*Note*. SMD = standardised mean difference; UPDRS-III = Unified Parkinson's Disease Rating Scale Part III (original version). For Hedges' *g*, values > 0 indicate better cognitive performance among tremor-dominant (TD) patients relative to postural instability gait disorder (PIGD) patients.

## Results of Tremor-Dominant vs. Indeterminate Analyses

### *Demographics and Disease Characteristics*

The results of traditional meta-analyses comparing TD and ID motor subtype groups on demographics and disease characteristics are reported in Table S7. No significant group differences emerged for gender, years of education, or age at disease onset. All remaining pooled effect sizes indicated negligible-to-small differences between groups; ID patients were marginally older in age (*k* (*o*) = 19 (3642), *g* = 0.12 [0.03-0.21], *p* = .016), had slightly longer disease duration (*k* (*o*) = 17 (3306), *g* = 0.14 [0.03-0.25], *p* = .014), and had a higher LEDD (*k* (*o*) = 13 (2627), *g* = 0.29 [0.22-0.37], $p < .001$). When motor symptom severity was assessed using the original version of the UPDRS-III (Fahn et al., 1987), a small-to-moderate difference was found (*k* (*o*) = 10 (1368), *g* = 0.41 [0.17-0.66], *p* = .004), suggesting greater motor impairment in ID patients relative to TD patients. However, when the MDS-UPDRS-III (Goetz et al., 2008) was used to assess motor symptom severity, this group difference was weaker in magnitude and failed to reach statistical significance (*k* (*o*) = 9 (2274), *g* = 0.21 [0.01-0.42], *p* = .055). This is consistent with our TD vs. PIGD comparison, where a significant pooled effect size for the group difference in motor symptom severity was found for the original UPDRS-III but not the MDS-UPDRS-III.

**Table S7**

*Meta-Analyses Comparing Tremor-Dominant (TD) and Indeterminate (ID) Motor Subtypes on Demographics and Disease Characteristics*

| Characteristic | *k* (*o*) | Pooled effect size (95% CI) | *p* | $I^2$ (%) | *Q* (*p*) | Egger's test (*p*) |
|---|---|---|---|---|---|---|
| **Age*** | | | | | | |
| *All studies* | 19 (3642) | -0.12 (-0.21, -0.03) | **.016** | 11.4 | 20.31 (.315) | **.006** |
| **Gender (proportion of men)*** | | | | | | |
| *All studies* | 18 (3532) | 1.03 (0.97, 1.09) | .364 | 0.0 | 13.28 (.717) | .765 |
| **Years of education*** | | | | | | |
| *All studies* | 9 (1212) | 0.02 (-0.14, 0.19) | .762 | 13.2 | 9.22 (.324) | - |
| **Disease duration*** | | | | | | |
| *All studies* | 17 (3306) | -0.14 (-0.25, -0.03) | **.014** | 25.1 | 21.35 (.166) | .081 |
| **Age at onset*** | | | | | | |
| *All studies* | 13 (2836) | -0.10 (-0.20, 0.00) | .057 | 3.2 | 12.40 (.414) | .193 |
| **LEDD*** | | | | | | |
| *All studies* | 13 (2627) | -0.29 (-0.37, -0.22) | **< .001** | 0.0 | 6.53 (.887) | .535 |
| **UPDRS-III total score (original version)** | | | | | | |
| *All studies* | 10 (1368) | -0.41 (-0.66, -0.17) | **.004** | 61.9 | 23.64 (**.005**) | .402 |
| *With outliers removed* | 9 (1258) | -0.31 (-0.46, -0.15) | **.002** | 9.8 | 8.87 (.353) | - |
| **MDS-UPDRS-III total score*** | | | | | | |
| *All studies* | 9 (2274) | -0.21 (-0.42, 0.01) | .055 | 65.1 | 22.90 (**.004**) | - |

| | | | | | | |
|---|---|---|---|---|---|---|
| **UPDRS-III total score (any version)** | | | | | | |
| *All studies* | 19 | -0.32 | **< .001** | 66.8 | 54.18 | .118 |
| | (3642) | (-0.48, -0.17) | | | (**< .001**) | |
| *With outliers removed* | 18 | -0.26 | **< .001** | 51.7 | 35.22 | .118 |
| | (3532) | (-0.39, -0.14) | | | (**.006**) | |

*Note.* CI = confidence interval; *k* = number of studies/effect sizes; LEDD = levodopa equivalent daily dose; MDS-UPDRS-III = Movement Disorder Society Unified Parkinson's Disease Rating Scale Part III; *o* = pooled sample size (both groups); UPDRS-III = Unified Parkinson's Disease Rating Scale Part III. Pooled effect size is Hedges' *g* for all variables except gender, which is risk ratio. For Hedges' *g*, negative pooled effect sizes reflect higher values (i.e., older age, longer disease duration) in the indeterminate group relative to the tremor-dominant group; for risk ratio, values > 1 indicate that tremor-dominant patients have a greater risk of being men relative to indeterminate patients. $I^2$ is for between-study variance. Egger's test was conducted only when $k \geq 10$ and using Pustejovsky and Rodgers' (2019) revised method. *Results for model with outliers removed not reported as no outliers detected. Significant *p*-values (< .05) reported in bold.

### *Cognition*

**Main Analyses.** Compared to our analysis of TD and PIGD motor subtypes, less than half the number of studies ($n$ = 19) and effect sizes ($k$ = 72) were available for our multi-level meta-analysis comparing cognitive performance in TD and ID patients. A negligible-to-small difference in cognitive performance was observed between TD and ID patients, favouring TD patients (Table S8). Multi-level Egger's tests did not indicate evidence of publication bias; however, removal of outliers did reduce the magnitude of the pooled effect. Of note, sampling error variance was especially high for all three models (range = 88.93-100.00%).

**Table S8**

*Results of Multi-Level Meta-Analyses of Cognitive Performance in Tremor-Dominant (TD) and Indeterminate (ID) Motor Subtype Groups*

| **Subtype Pair** | **Model** | ***n* (*k*)** | **Pooled Hedges' *g* (95% CI)** | ***p*** | **Within-Study $I^2$ (%) (*p*)** | **Between-Study $I^2$ (%) (*p*)** | ***Q* (*p*)** | **Multi-level Egger's test (*p*)** |
|---|---|---|---|---|---|---|---|---|
| TD vs. ID | All studies | 19 (72) | 0.15 (0.08, 0.23) | **< .001** | 11.07 (.162) | 0.00 (.500) | 81.57 (.184) | 0.19 (.665) |
| | With outliers removed – residuals approach | 19 (62) | 0.15 (0.08, 0.22) | **< .001** | 0.00 (.500) | 0.00 (.500) | 43.64 (.955) | 0.00 (.971) |
| | With outliers removed – Cook's distance approach | 13 (60) | 0.13 (0.05, 0.21) | **.003** | 0.00 (.500) | 0.00 (.500) | 49.51 (.806) | 0.40 (.528) |

*Note*. CI = confidence interval; ID = indeterminate; *k* = number of effect sizes; *n* = number of unique studies; TD = tremor-dominant. For Hedges' *g*, values > 0 indicate poorer cognitive performance among indeterminate (ID) patients relative to tremor-dominant (TD) patients. Significant *p*-values (< .05) are highlighted in bold.

The results of our traditional meta-analyses evaluating the prevalence of MCI and dementia in TD and ID patients are presented in Table S9. Compared to TD patients, ID patients had an 18% greater relative risk of MCI and a 41% greater relative risk of dementia. Relative to our multi-level meta-analysis of continuous cognitive data, the number of included studies for these analyses was small ($n$ = 9 and $n$ = 7, respectively), but between-study heterogeneity was low ($p$ > .05 for both $Q$s).

**Table S9**

*Results of Meta-Analyses Comparing Cognitive Status in Tremor-Dominant and Indeterminate (ID) Motor Subtype Groups*

| **Outcome** | ***k*** **(*o*)** | **Pooled risk ratio (95% CI)** | ***p*** | **$I^2$ (%)** | ***Q*** **(*p*)** |
|---|---|---|---|---|---|
| Mild cognitive impairment* | 9 (2301) | 1.18 (0.97, 1.44) | .082 | 0.00 | 7.44 (.490) |
| Dementia* | 6 (1383) | 1.41 (0.84, 2.39) | .149 | 12.6 | 5.72 (.334) |

*Note*. CI = confidence interval; $k$ = number of studies/effect sizes; $o$ = pooled sample size (both groups). For risk ratio, values > 1 indicate that indeterminate patients have a greater risk of mild cognitive impairment/dementia relative to tremor-dominant patients. $I^2$ is for between-study variance. *Results for model with outliers removed not reported as no outliers detected. Egger's test (using Pustejovsky and Rodgers' (2019) revised method) not performed as $k < 10$. Significant $p$-values (< .05) are highlighted in bold.

**Dose-Effect Analyses.** Neither the SMD in UPDRS tremor subscore nor the SMD in UPDRS PIGD subscore were significant moderators of differences in cognition between TD and ID motor subtypes (Table S10).

**Table S10**

*Results of Dose-Effect Analyses for Pairwise Comparisons Between Tremor-Dominant (TD), Postural Instability Gait Disorder (PIGD), and Indeterminate (ID) Motor Subtype Groups*

| **Subtype Pair** | **Moderator** | ***k*** | ***n*** | ***β* (95% CI)** | ***p*** | **Change in Between-Study $I^2$** |
|---|---|---|---|---|---|---|
| TD vs. PIGD (full model) | SMD in UPDRS tremor subscore | 23 | 80 | -0.04 (-0.35, 0.27) | .817 | -2.44 |
| | SMD in UPDRS PIGD subscore | 23 | 80 | 0.28 (-0.13, 0.68) | .178 | 2.75 |
| TD vs. PIGD (outliers removed – residuals approach) | SMD in UPDRS tremor subscore | 23 | 79 | -0.05 (-0.16, 0.07) | .429 | -4.41 |
| | SMD in UPDRS PIGD subscore | 23 | 79 | 0.05 (-0.10, 0.20) | .521 | -0.48 |
| TD vs. ID | SMD in UPDRS tremor subscore | 12 | 42 | 0.03 (-0.09, 0.15) | .630 | 0.00 |
| | SMD in UPDRS PIGD subscore | 12 | 42 | 0.02 (-0.08, 0.12) | .665 | 0.00 |
| PIGD vs. ID | SMD in UPDRS tremor subscore | 12 | 42 | -0.05 (-0.23, 0.13) | .544 | 0.00 |
| | SMD in UPDRS PIGD subscore | 12 | 42 | -0.06 (-0.42, 0.29) | .712 | 0.00 |

*Note*. CI = confidence interval; ID = indeterminate; *k* = number of unique studies; *n* = number of effect sizes; PIGD = postural instability gait disorder; SMD = standardised mean difference (Hedges' *g*); TD = tremor-dominant; UPDRS = Unified Parkinson's Disease Rating Scale (any version); $\beta$ = regression coefficient. For $\beta$, positive values reflect a positive association between the moderator and effect size (i.e., larger values of the moderator are associated with larger effect sizes, reflecting better cognitive performance in the tremor-dominant [TD] group relative to the postural instability gait disorder [PIGD] or indeterminate [ID] group, or better cognitive performance in the PIGD group relative to the ID group). Change in $I^2$ is the difference in between-study $I^2$ for the model with and without the moderator (larger positive values correspond to greater between-study variance being accounted for by the moderator; negative values suggest that model fit worsened as a consequence of including the moderator). Significant *p*-values (< .05) are highlighted in bold.

**Moderator Analyses.** None of our moderators were statistically significant (Tables S11-S12). Among our continuous moderators, publication year did approach significance ($p$ = .059; Table S11). As shown in Figure S5, the two studies published prior to 2010 (Alves et al., 2006; Williams-Gray et al., 2007) reported larger pooled effect sizes that favoured the TD group, consistent with our overall multi-level model (Table S8). In comparison, there was much greater variability in the effect sizes of studies published more recently. These studies tended to report a smaller cognitive advantage for TD patients or report the opposite effect of superior cognitive performance among ID patients relative to TD patients.

Of note, while cognitive domain was not a significant moderator of effect size, the pooled effect size reported for measures of cognitive flexibility ($g$ = 0.38) did differ significantly from the effect size estimate reported for measures of global cognitive function ($g$ = 0.13; Table S12, Figure S6). While this suggests that TD patients have a marked advantage over ID patients on measures of cognitive flexibility, this finding should be interpreted cautiously given that the mean effect size for cognitive flexibility was taken from a limited sample of only three data points ($n$ = 2 unique studies).

**Table S11**

*Results of Continuous Moderator Analyses for Tremor-Dominant (TD) and Indeterminate (ID) Motor Subtype Groups*

| **Moderator** | ***n*** | ***k*** | ***β* (95% CI)** | ***p*** | **Change in Between-Study $I^2$** |
|---|---|---|---|---|---|
| Sample size | 19 | 72 | 0.00 (-0.00, 0.00) | .904 | 0.00 |
| Publication year | 19 | 72 | -0.02 (-0.03, 0.00) | .059 | 0.00 |
| Pooled mean age | 19 | 72 | 0.02 (-0.01, 0.05) | .129 | 0.00 |
| Pooled proportion of men | 18 | 71 | -0.15 (-1.07, 0.76) | .738 | 0.00 |
| Pooled mean disease duration | 19 | 72 | 0.01 (-0.02, 0.05) | .465 | 0.00 |
| Pooled mean age at onset | 19 | 72 | 0.01 (-0.01, 0.04) | .368 | 0.00 |
| Pooled mean LEDD | 13 | 43 | 0.00 (-0.00, 0.00) | .867 | 0.00 |
| Pooled mean UPDRS-III (original) | 10 | 49 | 0.02 (-0.01, 0.04) | .169 | 2.49 |

*Note*. CI = confidence interval; *k* = number of effect sizes; LEDD = levodopa equivalent daily dose; *n* = number of unique studies; UPDRS-III = Unified Parkinson's Disease Rating Scale Part III; *β* = regression coefficient (larger positive values reflect better cognitive performance in the tremor-dominant (TD) group relative to the indeterminate (ID) group). Change in $I^2$ is the difference between between-study $I^2$ for the model with and without the moderator; larger positive values correspond to greater between-study variance being accounted for by the moderator. No moderator analyses performed for pooled mean years of education or pooled mean MDS-UPDRS-III score as $n < 10$ studies. Significant *p*-values (< .05) are highlighted in bold.

**Figure S5**

*Relationship Between Publication Year and Effect Size for Cognitive Difference Between Tremor-Dominant (TD) and Indeterminate (ID) Motor Subtype Groups*

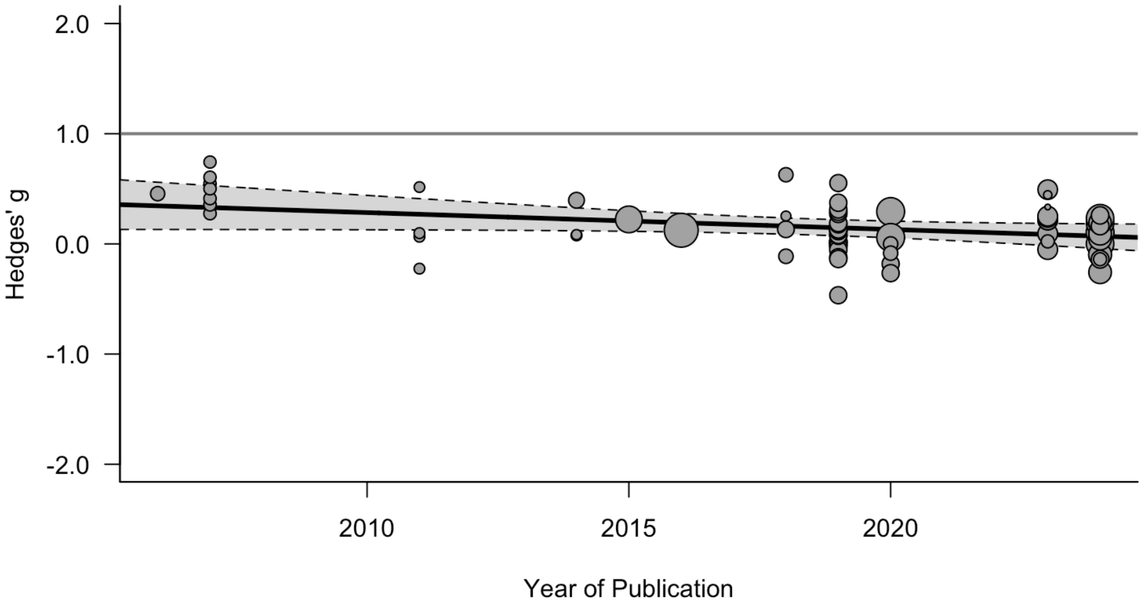


*Note*. For Hedges' *g*, values > 0 indicate better cognitive performance among tremor-dominant (TD) patients relative to indeterminate (ID) patients.

**Table S12**

*Results of Categorical Moderator Analyses for Tremor-Dominant (TD) and Indeterminate (ID) Motor Subtype Groups*

| Moderator | *n* | *k* | Pooled Hedges' *g* (95% CI) | *β* (95% CI) | *p* | *Q* (*p*) | ToM (*p*) |
|---|---|---|---|---|---|---|---|
| **Subtype Method** | | | | | | 54.08 (.254) | 0.77 (.383) |
| *Jankovic** | 9 | 27 | 0.20 (0.07, 0.33) | | **.004** | | |
| *Stebbins* | 9 | 23 | 0.12 (0.02, 0.23) | -0.07 (-0.24, 0.10) | .383 | | |
| **Cognitive Class** | | | | | | 79.77 (.199) | 0.94 (.335) |
| *Global** | 17 | 23 | 0.12 (0.03, 0.22) | | **.011** | | |
| *Specific* | 5 | 49 | 0.19 (0.07, 0.31) | 0.07 (-0.07, 0.22) | .335 | | |
| **Cognitive Domain**† | | | | | | 59.23 (.128) | 1.37 (.228) |
| *Global Cognitive Function** | 17 | 23 | 0.13 | | **.008** | | |
| *Executive Function – Cognitive Flexibility* | 2 | 3 | 0.38 | 0.25 (0.02, 0.48) | **.035** | | |
| *Executive Function – Working Memory* | 3 | 6 | 0.22 | 0.09 (-0.11, 0.28) | .361 | | |
| *Higher-Order Fluid Abilities – Planning* | 2 | 2 | 0.16 | 0.03 (-0.36, 0.41) | .884 | | |
| *Language* | 4 | 8 | 0.16 | 0.03 (-0.14, 0.20) | .745 | | |
| *LTM/Learning – Lexical* | 2 | 2 | 0.26 | 0.13 (-0.09, 0.34) | .245 | | |
| *LTM/Learning – Semantic* | 3 | 3 | 0.19 | 0.06 (-0.18, 0.35) | .526 | | |

| | | | | | | | |
|---|---|---|---|---|---|---|---|
| *LTM/Learning – Visuospatial* | 2 | 2 | 0.15 | 0.02 (-0.32, 0.36) | .918 | | |
| *STM – Lexical* | 3 | 3 | 0.14 | 0.01 (-0.25, 0.27) | .928 | | |
| *Processing Speed* | 3 | 6 | 0.19 | 0.06 (-0.22, 0.34) | .679 | | |
| **Medication Status for Motor Assessment(s)** | | | | | | 51.36 (.239) | 0.86 (.469) |
| *De novo** | 5 | 28 | 0.10 (-0.03, 0.23) | | .134 | | |
| *ON* | 7 | 14 | 0.28 (0.08, 0.48) | 0.18 (-0.05, 0.42) | .127 | | |
| *OFF* | 3 | 4 | 0.17 (-0.01, 0.36) | 0.08 (-0.15, 0.30) | .505 | | |
| *ON or OFF* | 2 | 3 | 0.12 (-0.09, 0.32) | 0.02 (-0.22, 0.26) | .870 | | |
| **Cognitive Impairment/Dementia as Exclusion Criterion** | | | | | | 80.15 (.191) | 1.45 (.232) |
| *No** | 11 | 53 | 0.12 (0.04, 0.21) | | .005 | | |
| *Yes* | 8 | 19 | 0.22 (0.08, 0.36) | 0.10 (-0.06, 0.26) | .232 | | |
| **Motor Subtypes Compared on Cognition in Paper** | | | | | | 63.78 (.250) | 1.49 (.228) |
| *No** | 8 | 22 | 0.22 (0.10, 0.34) | | **< .001** | | |
| *Yes* | 10 | 37 | 0.12 (0.01, 0.23) | -0.10 (-0.26, 0.06) | .228 | | |

| | | | | | | | |
|---|---|---|---|---|---|---|---|
| **Overall Risk of Bias** | | | | | | 80.31 (.166) | 0.74 (.482) |
| *High*[*] | 7 | 20 | 0.21 (0.09, 0.32) | | **< .001** | | |
| *Moderate* | 8 | 31 | 0.11 (-0.05, 0.26) | -0.10 (-0.29, 0.09) | .305 | | |
| *Low* | 4 | 21 | 0.12 (-0.03, 0.26) | -0.09 (-0.27, 0.09) | .337 | | |

*Note*. CI = confidence interval; *k* = number of effect sizes; LTM = long-term memory; *n* = number of unique studies; STM = short-term memory; ToM = Test of Moderators; $\beta$ = regression coefficient. [*] denotes reference category for model. The $\beta$ coefficients and corresponding *p*-values indicate whether the pooled effect size for that level of the moderator differs significantly from the pooled effect size of the reference category; for the reference category, these values indicate whether the pooled effect size differs significantly from zero. For Hedges' *g*, values > 0 indicate poorer cognitive performance among indeterminate (ID) patients relative to tremor-dominant (TD) patients. [†] 95% confidence intervals not reported as model without intercept failed to reach convergence. No moderator analyses performed for medication status for cognitive assessment(s) as *n* < 10 studies. Significant *p*-values (< .05) are highlighted in bold.

**Figure S6**

*Orchard Plot of Effect Sizes for Difference in Cognitive Performance Between Tremor-Dominant (TD) and Indeterminate (ID) Motor Subtype Groups According to Cognitive Domain*

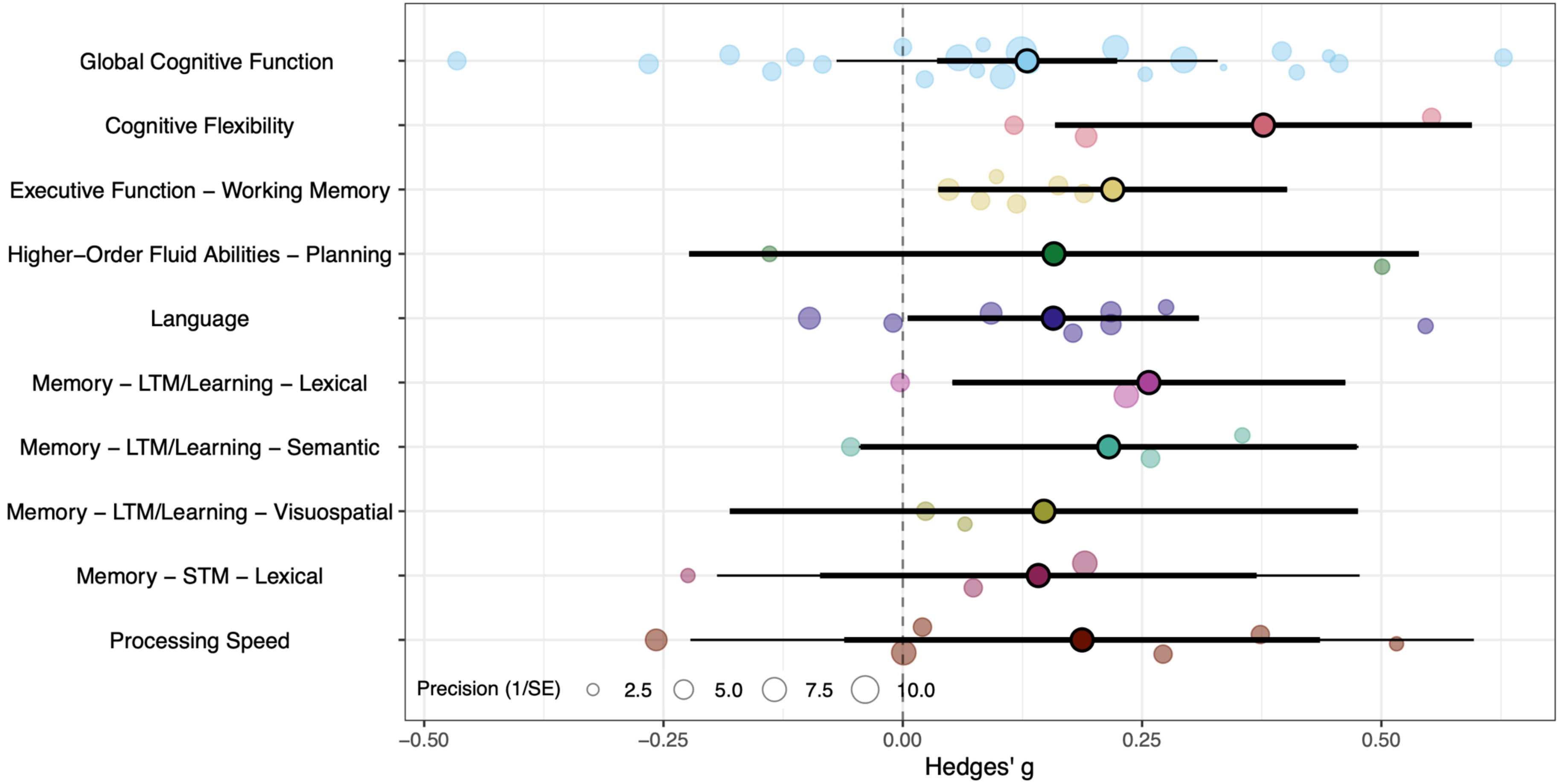


*Note*. For Hedges' *g*, values > 0 indicate better cognitive performance among tremor-dominant (TD) patients relative to indeterminate (ID) patients.

**Confound Analyses.** None of the variables we investigated as possible confounds significantly moderated effect size magnitude for the difference in cognitive performance between TD and ID motor subtype groups (Table S13).

**Table S13**

*Results of Confound Analyses for Tremor-Dominant (TD) and Indeterminate (ID) Motor Subtype Groups*

| **Confound** | ***n*** | ***k*** | ***β* (95% CI)** | ***p*** | **Change in Between-Study $I^2$** |
|---|---|---|---|---|---|
| SMD age | 19 | 72 | -0.10 (-0.48, 0.29) | .618 | 0.00 |
| Difference in proportion of men | 18 | 71 | 0.78 (-0.31, 1.87) | .158 | 0.00 |
| SMD disease duration | 17 | 64 | -0.04 (-0.49, 0.40) | .842 | 0.00 |
| SMD age at onset | 13 | 40 | -0.11 (-0.63, 0.41) | .673 | 0.00 |
| SMD LEDD | 13 | 43 | -0.40 (-1.08, 0.28) | .246 | 0.00 |
| SMD UPDRS-III (original) | 10 | 49 | -0.19 (-0.59, 0.21) | .349 | -1.30 |
| SMD UPDRS-III (any version) | 19 | 72 | -0.09 (-0.34, 0.17) | .498 | 0.00 |

*Note*. CI = confidence interval; *k* = number of effect sizes; LEDD = levodopa equivalent daily dose; UPDRS-III = Unified Parkinson's Disease Rating Scale Part III; SMD = standardised mean difference (Hedges' *g*); *β* = regression coefficient. For *β*, positive values reflect a positive association between the confound (moderator) and effect size (i.e., larger values of the confound [moderator] are associated with larger effect sizes, reflecting better cognitive performance in the tremor-dominant [TD] group relative to the indeterminate [ID] group). Change in $I^2$ is the difference in between-study $I^2$ for the model with and without the confound (moderator; larger positive values correspond to greater between-study variance being accounted for by the confound [moderator]; negative values suggest that model fit worsened as a consequence of including the confound [moderator]). No confound (moderator) analyses performed for SMD in years of education or SMD in Movement Disorder Society (MDS)-UPDRS-III score as $n < 10$ studies. Significant *p*-values (< .05) are highlighted in bold.

## Results of Postural Instability Gait Disorder vs. Indeterminate Analyses

### *Demographics and Disease Characteristics*

The results of traditional meta-analyses comparing PIGD and ID motor subtype groups on demographics and disease characteristics are reported in Table S14. No significant differences were found between subtype groups for gender, years of education, disease duration, or UPDRS-III scores (original or MDS version). All remaining pooled effect sizes indicated negligible-to-small differences between groups. Relative to ID participants, PIGD participants were older at time of disease onset ($k$ ($o$) = 13 (3284), $g$ = 0.14 [0.02-0.26], $p$ = .028) and at time of study participation ($k$ ($o$) = 19 (4885), $g$ = 0.14 [0.03-0.25], $p$ = .012), and reported a higher LEDD ($k$ ($o$) = 13 (3388), $g$ = 0.18 [0.06-0.31], $p$ = .007).

**Table S14**

*Meta-Analyses Comparing Postural Instability and Gait Disorder (PIGD) and Indeterminate (ID) Motor Subtypes on Demographics and Disease Characteristics*

| **Characteristic** | ***k* (*o*)** | **Pooled effect size (95% CI)** | ***p*** | **$I^2$ (%)** | ***Q* (*p*)** | **Egger's test (*p*)** |
|---|---|---|---|---|---|---|
| **Age** | | | | | | |
| *All studies* | 19 (4885) | 0.14 (0.03, 0.25) | **.012** | 32.8 | 26.79 (.083) | .848 |
| *With outliers removed* | 18 (4804) | 0.12 (0.04, 0.21) | **.006** | 0.0 | 16.90 (.461) | .347 |
| **Gender (proportion of men)*** | | | | | | |
| *All studies* | 18 (4592) | 0.97 (0.91, 1.04) | .406 | 0.0 | 17.00 (.454) | .641 |
| **Years of education*** | | | | | | |
| *All studies* | 9 (2346) | -0.06 (-0.19, 0.08) | .348 | 0.0 | 7.25 (.510) | - |
| **Disease duration*** | | | | | | |
| *All studies* | 17 (4571) | 0.07 (-0.01, 0.15) | .071 | 0.0 | 14.35 (.573) | .819 |
| **Age at onset*** | | | | | | |
| *All studies* | 13 (3284) | 0.14 (0.02, 0.26) | **.028** | 32.5 | 17.79 (.122) | .590 |
| **LEDD** | | | | | | |
| *All studies* | 13 (3388) | 0.18 (0.06, 0.31) | **.007** | 23.3 | 15.65 (.208) | .301 |
| *With outliers removed* | 12 (3095) | 0.23 (0.16, 0.31) | **< .001** | 0.0 | 6.48 (.840) | .220 |
| **UPDRS-III total score (original version)*** | | | | | | |
| *All studies* | 10 (2387) | 0.08 (-0.04, 0.19) | .171 | 0.0 | 8.07 (.527) | .719 |

| | | | | | | |
|---|---|---|---|---|---|---|
| **MDS-UPDRS-III total score*** | | | | | | |
| *All studies* | 9 | -0.01 | .892 | 27.5 | 11.03 | - |
| | (2498) | (-0.17, 0.15) | | | (.200) | |
| **UPDRS-III total score (any version)*** | | | | | | |
| *All studies* | 19 | 0.04 | .318 | 9.2 | 19.83 | .392 |
| | (4885) | (-0.04, 0.13) | | | (.343) | |

*Note.* CI = confidence interval; *k* = number of studies/effect sizes; LEDD = levodopa equivalent daily dose; MDS-UPDRS-III = Movement Disorder Society Unified Parkinson's Disease Rating Scale Part III; *o* = pooled sample size (both groups); UPDRS-III = Unified Parkinson's Disease Rating Scale Part III. Pooled effect size is Hedges' *g* for all variables except gender, which is risk ratio. For Hedges' *g*, negative pooled effect sizes reflect higher values (i.e., older age, longer disease duration) in the indeterminate group relative to the postural instability and gait disorder (PIGD) group; for risk ratio, values > 1 indicate that postural instability and gait disorder (PIGD) patients have a greater risk of being men relative to indeterminate patients. $I^2$ is for between-study variance. Egger's test was conducted only when $k \geq 10$ and using Pustejovsky and Rodgers' (2019) revised method. *Results for model with outliers removed not reported as no outliers detected. Significant *p*-values (< .05) are highlighted in bold.

### *Cognition*

**Main Analyses.** Our multi-level meta-analysis of 72 effect sizes from 19 studies revealed a negligible-to-small difference in cognitive performance between PIGD and ID participants, favouring the ID group (Table S15). Removal of outliers resulted in a slight reduction in the pooled effect size estimate, but multi-level Egger's tests did not indicate evidence of publication bias. As with our TD vs. ID comparison, sampling error variance was high across all models (range = 69.85-82.09%).

**Table S15**

*Results of Multi-Level Meta-Analyses of Cognitive Performance in Indeterminate (ID) and Postural Instability Gait Disorder (PIGD) Motor Subtype Groups*

| **Subtype Pair** | **Model** | ***n* (*k*)** | **Pooled Hedges' *g* (95% CI)** | ***p*** | **Within-Study $I^2$ (%) (*p*)** | **Between-Study $I^2$ (%) (*p*)** | ***Q* (*p*)** | **Multi-level Egger's test (*p*)** |
|---|---|---|---|---|---|---|---|---|
| PIGD vs. ID | All studies | 19 (72) | -0.14 (-0.21, -0.06) | **.005** | 24.18 (**.007**) | 5.97 (.358) | 105.44 (**.005**) | 0.08 (.777) |
| | With outliers removed – residuals approach | 16 (65) | -0.12 (-0.19, -0.05) | **.001** | 17.91 (**.046**) | 2.92 (.500) | 72.19 (.226) | 1.27 (.265) |
| | With outliers removed – Cook's distance approach | 14 (63) | -0.13 (-0.21, -0.04) | **.004** | 21.02 (**.021**) | 7.24 (.500) | 78.91 (.073) | 0.49 (.488) |

*Note*. CI = confidence interval; ID = indeterminate; *k* = number of effect sizes; *n* = number of unique studies; PIGD = postural instability gait disorder. For Hedges' *g*, values < 0 indicate poorer cognitive performance among postural instability gait disorder (PIGD) patients relative to indeterminate (ID) patients. Significant *p*-values (< .05) are highlighted in bold.

Our traditional meta-analysis found no statistically significant difference in MCI prevalence between PIGD and ID subtypes (Table S16). While dementia rates did differ considerably between subtypes (RR = 1.53), indicating a higher rate of dementia among PIGD patients, this effect did not reach significance ($p$ = .073). Between-study heterogeneity was low ($I^2$ = 23.6%; $Q$ = 6.54, $p$ = .257).

**Table S16**

*Results of Meta-Analyses Comparing Cognitive Status in Postural Instability Gait Disorder (PIGD) and Indeterminate (ID) Motor Subtype Groups*

| **Outcome** | ***k* (*o*)** | **Pooled risk ratio (95% CI)** | ***p*** | **$I^2$ (%)** | ***Q* (*p*)** |
|---|---|---|---|---|---|
| Mild cognitive impairment* | 9 (2642) | 1.15 (0.92, 1.43) | .195 | 37.0 | 12.69 (.123) |
| Dementia* | 6 (1403) | 1.53 (0.94, 2.48) | .073 | 23.6 | 6.54 (.257) |

*Note*. CI = confidence interval; $k$ = number of studies/effect sizes; $o$ = pooled sample size (both groups). For risk ratio, values > 1 indicate that postural instability and gait disorder patients have a greater risk of mild cognitive impairment/dementia relative to indeterminate patients. $I^2$ is for between-study variance. *Results for model with outliers removed not reported as no outliers detected. Egger's test (using Pustejovsky and Rodgers' (2019) revised method) not performed as $k < 10$. Significant $p$-values (< .05) are highlighted in bold.

**Dose-Effect Analyses.** Neither the SMD in UPDRS tremor subscore nor the SMD in UPDRS PIGD subscore were significant moderators of differences in cognition between PIGD and ID motor subtypes (Table S10).

**Moderator Analyses.** The results of our analyses investigating potential moderators of the difference in cognitive performance between PIGD and ID motor subtype groups are reported in Tables S17 and S18. None of our moderators were statistically significant. Interestingly, although cognitive domain was not a significant moderator of effect size, as per our comparison of TD and ID motor subtypes, we found that the pooled effect size estimate for cognitive flexibility ($g$ = 0.03 [95% CI = -0.16-0.22]) differed significantly from the mean effect size estimate for global cognitive function (Figure S7). Whereas TD patients, relative to ID patients, were found to have a *greater* advantage on measures of cognitive flexibility compared to measures of global cognition, the opposite effect was found for our PIGD vs. ID

comparison; namely, ID patients' cognitive advantage relative to PIGD patients was negligible on measures of cognitive flexibility compared to measures of global cognition. Although this estimate lacked precision (and did not differ significantly from global cognitive function), we also found the difference in performance on measures of cognitive flexibility between TD and PIGD patients to be of moderate magnitude (*g* = 0.24 [95% CI = -0.03-0.51], Table 8 in Main Text). Taken together, these results indicate that preserved (or enhanced) cognitive flexibility may be a distinct feature of TD patients' cognitive profile.

**Table S17**

*Results of Continuous Moderator Analyses for Postural Instability Gait Disorder (PIGD) and Indeterminate (ID) Motor Subtype Groups*

| **Moderator** | ***n*** | ***k*** | ***β* (95% CI)** | ***p*** | **Change in Between-Study $I^2$** |
|---|---|---|---|---|---|
| Sample size | 19 | 72 | 0.00 (-0.00, 0.00) | .208 | 0.72 |
| Publication year | 19 | 72 | -0.01 (-0.03, 0.01) | .413 | -7.15 |
| Pooled mean age | 19 | 72 | -0.02 (-0.04, 0.00) | .127 | 5.85 |
| Pooled proportion of men | 18 | 71 | 0.06 (-1.23, 1.34) | .932 | -4.22 |
| Pooled mean disease duration | 19 | 72 | -0.01 (-0.04, 0.01) | .311 | 5.33 |
| Pooled mean age at onset | 19 | 72 | -0.01 (-0.02, 0.01) | .578 | -4.20 |
| Pooled mean LEDD | 13 | 43 | -0.00 (-0.00, 0.00) | .369 | 1.49 |
| Pooled mean UPDRS-III (original) | 10 | 49 | 0.01 (-0.01, 0.03) | .280 | 1.24 |

*Note*. CI = confidence interval; *k* = number of effect sizes; LEDD = levodopa equivalent daily dose; *n* = number of unique studies; UPDRS-III = Unified Parkinson's Disease Rating Scale Part III; $\beta$ = regression coefficient. For $\beta$, positive values reflect a positive association between the moderator and effect size (i.e., larger values of the moderator are associated with larger effect sizes, reflecting better cognitive performance in the postural instability gait disorder [PIGD] group relative to the indeterminate [ID] group). Change in $I^2$ is the difference in between-study $I^2$ for the model with and without the moderator (larger positive values correspond to greater between-study variance being accounted for by the moderator; negative values suggest that model fit worsened as a consequence of including the moderator). No moderator analyses performed for pooled mean years of education or pooled mean Movement Disorder Society (MDS)-UPDRS Part III score as $n < 10$ studies. Significant *p*-values (< .05) are highlighted in bold.

**Table S18**

*Results of Categorical Moderator Analyses for Postural Instability Gait Disorder (PIGD) and Indeterminate (ID) Motor Subtype Groups*

| **Moderator** | ***n*** | ***k*** | **Pooled Hedges' *g* (95% CI)** | ***β* (95% CI)** | ***p*** | ***Q* (*p*)** | **ToM (*p*)** |
|---|---|---|---|---|---|---|---|
| **Subtype Method** | | | | | | 68.59 (**.027**) | 0.11 (.740) |
| *Jankovic** | 9 | 27 | -0.11 (-0.22, -0.00) | | **.041** | | |
| *Stebbins* | 9 | 23 | -0.15 (-0.33, 0.03) | -0.04 (-0.25, 0.18) | .740 | | |
| **Cognitive Class** | | | | | | 102.75 (**.007**) | 1.89 (.173) |
| *Global** | 17 | 23 | -0.18 (-0.29, -0.07) | | **.002** | | |
| *Specific* | 5 | 49 | -0.09 (-0.19, 0.01) | 0.09 (-0.04, 0.21) | .173 | | |
| **Cognitive Domain** | | | | | | 73.47 (**.010**) | 1.30 (.263) |
| *Global Cognitive Function** | 17 | 23 | -0.18 (-0.29, -0.07) | | **.002** | | |
| *Executive Function – Cognitive Flexibility* | 2 | 3 | 0.03 (-0.16, 0.22) | 0.21 (0.00, 0.41) | **.048** | | |
| *Executive Function – Working Memory* | 3 | 6 | -0.12 (-0.28, 0.04) | 0.06 (-0.11, 0.22) | .504 | | |
| *Higher-Order Fluid Abilities – Planning* | 2 | 2 | -0.09 (-0.46, 0.28) | 0.09 (-0.29, 0.47) | .635 | | |
| *Language* | 4 | 8 | -0.13 (-0.27, 0.01) | 0.05 (-0.11, 0.21) | .571 | | |
| *LTM/Learning – Lexical* | 2 | 2 | 0.07 (-0.12, 0.26) | 0.25 (0.05, 0.44) | **.015** | | |
| *LTM/Learning – Semantic* | 3 | 3 | -0.06 (-0.36, 0.24) | 0.12 (-0.16, 0.39) | .394 | | |

| | | | | | | | |
|---|---|---|---|---|---|---|---|
| *LTM/Learning – Visuospatial* | 2 | 2 | -0.05 (-0.35, 0.24) | 0.13 (-0.16, 0.41) | .376 | | |
| *STM – Lexical* | 3 | 3 | -0.03 (-0.24, 0.17) | 0.14 (-0.05, 0.34) | .149 | | |
| *Processing Speed* | 3 | 6 | -0.17 (-0.31, -0.02) | 0.01 (-0.16, 0.18) | .897 | | |
| **Medication Status for Motor Assessment(s)** | | | | | | 67.07 (**.018**) | 0.21 (.892) |
| *De novo*[*] | 5 | 28 | -0.11 (-0.24, 0.01) | | .074 | | |
| *ON* | 7 | 14 | -0.19 (-0.51, 0.14) | -0.07 (-0.41, 0.27) | .680 | | |
| *OFF* | 3 | 4 | -0.05 (-0.24, 0.14) | 0.06 (-0.17, 0.29) | .578 | | |
| *ON or OFF* | 2 | 3 | -0.09 (-0.32, 0.14) | 0.02 (-0.24, 0.28) | .857 | | |
| **Cognitive Impairment/Dementia as Exclusion Criterion** | | | | | | 105.20 (**.004**) | 0.01 (.935) |
| *No*[*] | 11 | 53 | -0.14 (-0.23, -0.06) | | **.001** | | |
| *Yes* | 8 | 19 | -0.15 (-0.42, 0.11) | -0.01 (-0.29, 0.26) | .935 | | |
| **Motor Subtypes Compared on Cognition in Paper** | | | | | | 74.83 (.057) | 0.01 (.910) |
| *No*[*] | 8 | 22 | -0.19 (-0.40, 0.03) | | .094 | | |
| *Yes* | 10 | 37 | -0.17 (-0.27, -0.07) | 0.01 (-0.23, 0.25) | .910 | | |

| | | | | | | | |
|---|---|---|---|---|---|---|---|
| **Overall Risk of Bias** | | | | | | 102.22 (**.006**) | 1.03 (.362) |
| *High*[*] | 7 | 20 | -0.18 (-0.42, 0.06) | | .146 | | |
| *Moderate* | 8 | 31 | -0.20 (-0.32, -0.08) | -0.02 (-0.29, 0.25) | .883 | | |
| *Low* | 4 | 21 | -0.06 (-0.21, 0.08) | 0.11 (-0.17, 0.39) | .424 | | |

*Note*. CI = confidence interval; *k* = number of effect sizes; LTM = long-term memory; *n* = number of unique studies; STM = short-term memory; ToM = Test of Moderators; $\beta$ = regression coefficient. [*] denotes reference category for model. The $\beta$ coefficients and corresponding *p*-values indicate whether the pooled effect size for that level of the moderator differs significantly from the pooled effect size of the reference category; for the reference category, these values indicate whether the pooled effect size differs significantly from zero. For Hedges' *g*, values < 0 indicate poorer cognitive performance among postural instability gait disorder (PIGD) patients relative to indeterminate (ID) patients. No moderator analysis performed for medication status at time of cognitive assessment(s) as *n* < 10 studies. Significant *p*-values (< .05) are highlighted in bold.

**Figure S7**

*Orchard Plot of Effect Sizes for Difference in Cognitive Performance Between Postural Instability Gait Disorder (PIGD) and Indeterminate (ID) Motor Subtype Groups According to Cognitive Domain*

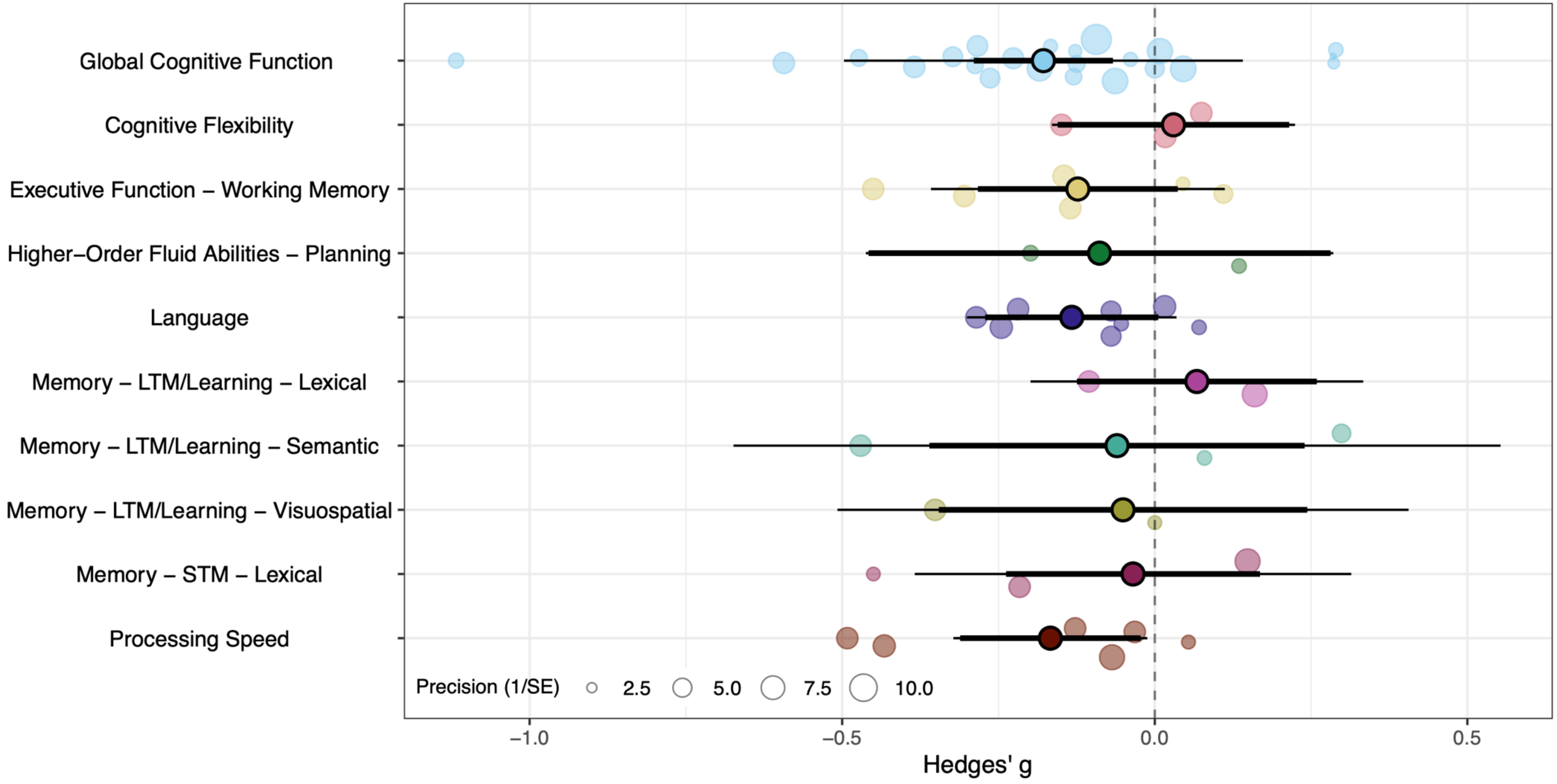


*Note*. For Hedges' *g*, values < 0 indicate better cognitive performance among indeterminate (ID) patients relative to postural instability gait disorder (PIGD) patients.

**Confound Analyses.** The results of our confound analyses are presented in Table S19. The standardised mean difference (SMD) in age, age at disease onset, and UPDRS-III score (any version) between PIGD and ID motor subtype groups were all significant moderators of the difference in cognitive performance between subtypes.

As shown in Figures S8 and S9, the SMD in age and age at time of disease onset had a similar influence on the difference in cognitive performance between PIGD and ID groups. As the magnitude of the SMD in mean age (or mean age at onset) increased above zero, reflecting older age (or older age at onset) in the PIGD group relative to the ID group, the (absolute) magnitude of the difference in cognitive performance also increased, favouring better cognition in the ID group. In contrast, as the magnitude of the SMD in mean age (or mean at age onset) increased below zero, reflecting older age (or older age at onset) in the ID group relative to the PIGD group, the magnitude of the difference in cognitive performance also increased, favouring better cognition in the PIGD group. Stated more simply, relatively older age and older age at onset are associated with worse cognitive performance in both motor subtype groups. Inspection of Figures S8 and S9 does, however, indicate that these trends are predominantly driven by a small handful of extreme values for the SMD in age and age at onset, respectively; thus, these results should be interpreted with caution[6].

We found evidence of a similar deleterious effect of motor symptom severity on the cognitive performance of both PIGD and ID motor subtype groups. As shown in Figure S10, as the SMD in UPDRS-III scores (any version) increased above zero, reflecting worse motor function in the PIGD group relative to the ID group, the (absolute) magnitude of the difference in cognitive performance also increased, reflecting poorer cognition among PIGD patients relative to ID patients. Similarly, as the SMD in UPDRS-III scores (any version) increased below zero, reflecting worse motor function in the ID group relative to the PIGD group, the magnitude of the difference in cognitive performance increased in favour of the PIGD group. These results provide evidence for an association between motor symptom severity and cognitive impairment in PD; however, it should be acknowledged that this pattern seems to be predominantly driven by a small handful of extreme values for the SMD in UPDRS-III scores (Figure S10), and so results should be interpreted cautiously. For all three significant confounds, between-study $I^2$ neared zero (between-study $I^2$ < 0.00%).

---

[6] It is also pertinent to note that these extreme values for the SMD in age and the SMD in age at onset have been reported by the *same* studies (Petrijan et al., 2023; Shen et al., 2023), indicating that the moderating effects of both characteristics may in fact be confounded with each other.

**Table S19**

*Results of Confound Analyses for Postural Instability Gait Disorder (PIGD) and Indeterminate (ID) Motor Subtype Groups*

| Confound | *n* | *k* | *β* (95% CI) | *p* | Change in Between-Study $I^2$ |
|---|---|---|---|---|---|
| SMD age | 19 | 72 | -0.59 (-0.97, -0.21) | **.003** | 5.97 |
| Difference in proportion of men | 18 | 71 | 0.30 (-0.76, 1.36) | .570 | -7.96 |
| SMD disease duration | 17 | 64 | -0.12 (-0.59, 0.34) | .603 | 1.51 |
| SMD age at onset | 13 | 40 | -0.58 (-1.06, -0.10) | **.019** | 12.54 |
| SMD LEDD | 13 | 43 | -0.22 (-0.73, 0.30) | .398 | -0.50 |
| SMD UPDRS-III (original) | 10 | 49 | -0.50 (-1.05, 0.04) | .069 | 3.84 |
| SMD UPDRS-III (any version) | 19 | 72 | -0.38 (-0.76, -0.00) | **.048** | 5.97 |

*Note*. CI = confidence interval; *k* = number of effect sizes; LEDD = levodopa equivalent daily dose; *n* = number of unique studies; UPDRS-III = Unified Parkinson's Disease Rating Scale Part III; SMD = standardised mean difference (Hedges' *g*); *β* = regression coefficient. For *β*, negative values reflect a positive association between the confound (moderator) and effect size (i.e., larger negative values of the confound [moderator] are associated with larger negative effect sizes, reflecting better cognitive performance in the indeterminate [ID] group relative to the postural instability gait disorder [PIGD] group). Change in $I^2$ is the difference in between-study $I^2$ for the model with and without the confound (moderator; larger positive values correspond to greater between-study variance being accounted for by the confound [moderator]; negative values suggest that model fit worsened as a consequence of including the confound [moderator]). No confound (moderator) analyses performed for SMD in years of education or SMD in Movement Disorder Society (MDS)-UPDRS-III score as $n < 10$ studies. Significant *p*-values (< .05) are highlighted in bold.

**Figure S8**

*Relationship Between Standardised Mean Difference (SMD) in Age and Effect Size for Cognitive Difference Between Postural Instability Gait Disorder (PIGD) and Indeterminate (ID) Motor Subtype Groups*

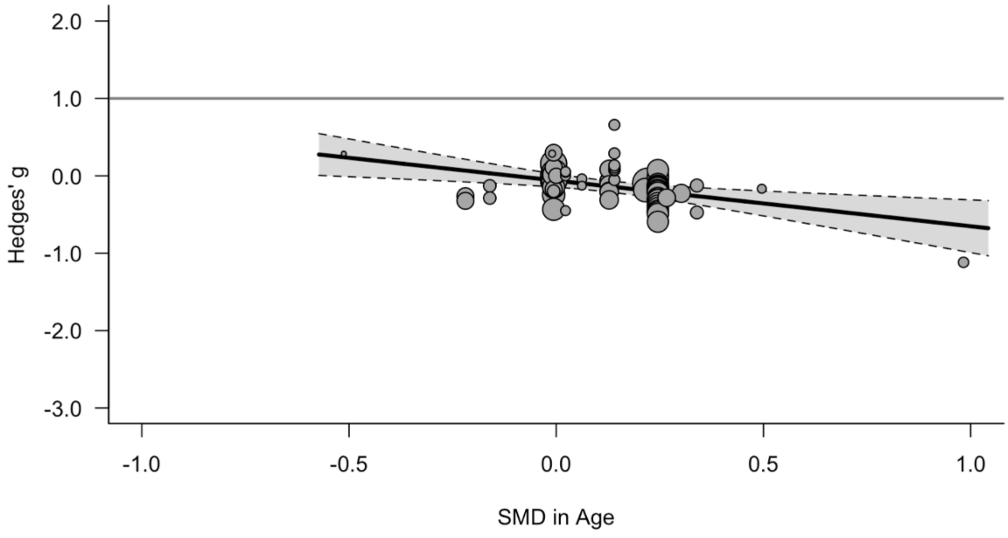


*Note*. SMD = standardised mean difference. For Hedges' *g*, values < 0 indicate better cognitive performance among indeterminate (ID) patients relative to postural instability gait disorder (PIGD) patients.

**Figure S9**

*Relationship Between Standardised Mean Difference (SMD) in Age at Disease Onset and Effect Size for Cognitive Difference Between Postural Instability Gait Disorder (PIGD) and Indeterminate (ID) Motor Subtype Groups*

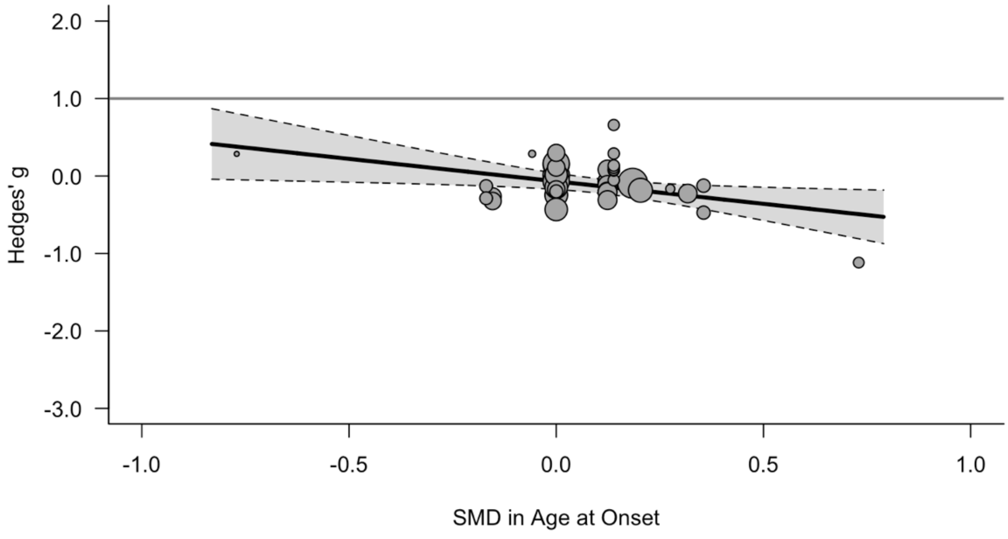


*Note*. SMD = standardised mean difference. For Hedges' *g*, values < 0 indicate better cognitive performance among indeterminate (ID) patients relative to postural instability gait disorder (PIGD) patients.

**Figure S10**

*Relationship Between Standardised Mean Difference (SMD) in Unified Parkinson's Disease Rating Scale (UPDRS) Part III Score (Any Version) and Effect Size for Cognitive Difference Between Postural Instability Gait Disorder (PIGD) and Indeterminate (ID) Motor Subtype Groups*

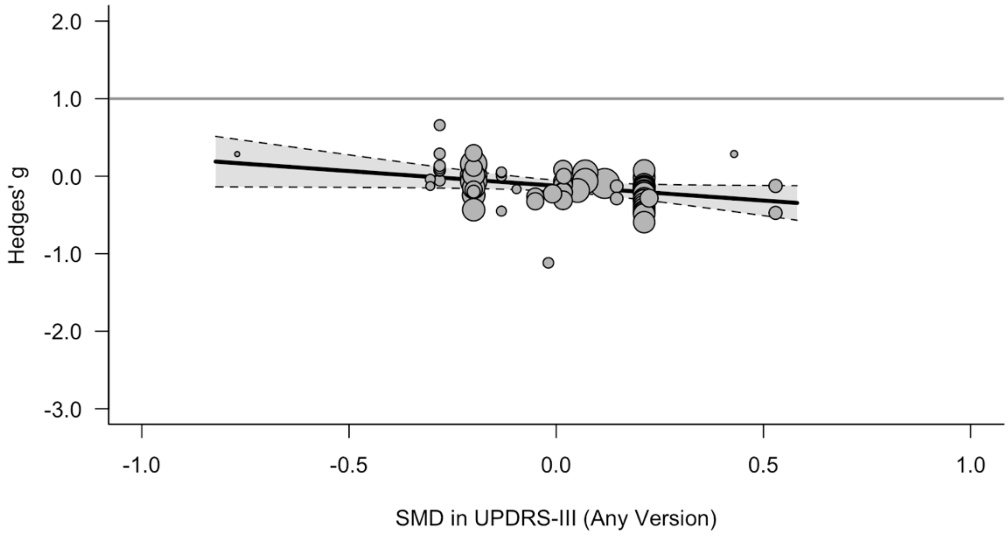


*Note*. SMD = standardised mean difference. For Hedges' *g*, values < 0 indicate better cognitive performance among indeterminate (ID) patients relative to postural instability gait disorder (PIGD) patients.

**Results of Predominantly Tremor-Dominant vs. Predominantly Postural Instability Gait Disorder Analyses**

Two studies (Herman et al., 2015; Ren et al., 2020) compared predominantly tremor-dominant (p-TD) and predominantly postural instability gait disorder (p-PIGD) motor subtype groups on continuous measures of cognition. No cognitive status (rates of MCI and/or dementia) data were reported for this motor subtype pair. Of note, Ren and colleagues (2020) compared p-TD and p-PIGD subtypes among a sample of *de novo* patients with a mean disease duration of 2.3 years whilst Herman and colleagues (2015) compared these subtypes among medicated patients with a mean disease duration of 5.5 years.

Both studies used a similar subtyping method (first reported in Herman et al., 2014), where either Jankovic et al. (1990) or Stebbins et al.'s (2013) subtyping method is first used to classify patients into TD, PIGD, and ID groups. Following this, more stringent criteria are then applied to exclude (1) ID patients; (2) TD patients with especially high PIGD subscores or low tremor subscores; and (3) PIGD patients with especially low PIGD subscores or high tremor subscores, irrespective of tremor to PIGD subscore ratio. This produces constrained TD and PIGD groups that only include patients with either severe tremor symptoms (p-TD) or severe PIGD symptoms (p-PIGD).

Given that the p-TD and p-PIGD subtype groups are intended to capture the most extreme cases of TD and PIGD, it is unsurprising that group differences were qualitatively similar to those observed for TD and PIGD groups (see Section 3.3 of main text). Group differences in age, gender, years of education, and age at disease onset were all negligible-to-small, and Herman et al. (2015) reported greater LEDD among p-PIGD patients relative to p-TD patients ($g = 0.33$). While Ren and colleagues (2020) reported much greater overall motor impairment among their *de novo* p-PIGD patients relative to their *de novo* p-TD patients ($g = 0.55$), Herman et al. (2015) reported a negligible effect among their medicated sample ($g = 0.06$). Interestingly, Herman and colleagues (2015) also reported longer disease duration among p-TD patients relative to p-PIGD patients ($g = 0.32$), contrary to the general observation that the PIGD subtype is associated with later disease stages (Fereshtehnejad & Postuma, 2017; Nutt, 2016).

Both Herman et al. (2015) and Ren et al. (2020) administered the MMSE and MoCA; Herman et al. (2015) also administered a series of domain-specific tasks, including the Trail Making Test (Parts A [TMT-A] and B [TMT-B]) and NeuroTrax Mindstreams computerised

cognitive battery. Group differences on the MMSE and MoCA differed markedly between studies; whilst Herman and colleagues (2015) reported moderate effect sizes (MMSE $g$ = 0.49; MoCA $g$ = 0.30) indicating better cognition among p-TD patients, Ren and colleagues (2020) reported negligible group differences on both screening measures (MMSE $g$ = 0.03; MoCA $g$ = 0.01). Among the domain-specific tasks administered by Herman et al. (2015), similar small-to-moderate effect sizes favouring p-TD patients were observed for all domains except for cognitive flexibility ($g$ = 0.14), where p-PIGD patients demonstrated marginally superior performance, and working memory ($g$ = 0.02), where no significance group difference was found.

**Results of Tremor-Dominant vs. Non-Tremor-Dominant Analyses**

**Table S20**

*Meta-Analyses Comparing Tremor-Dominant (TD) and Non-Tremor-Dominant (NTD) Motor Subtypes on Demographics and Disease Characteristics*

| **Characteristic** | ***k* (*o*)** | **Pooled effect size (95% CI)** | ***p*** | **$I^2$ (%)** | ***Q* (*p*)** | **Egger's test (*p*)** |
|---|---|---|---|---|---|---|
| **Age*** | | | | | | |
| *All studies* | 10 (2655) | -0.12 (-0.22, -0.01) | **.034** | 0.0 | 8.81 (.455) | .909 |
| **Gender (proportion of men)*** | | | | | | |
| *All studies* | 9 (2578) | 1.05 (0.98, 1.13) | .171 | 0.0 | 6.83 (.555) | - |
| **Years of education*** | | | | | | |
| *All studies* | 3 (1369) | 0.04 (-0.55, 0.63) | .794 | 51.4 | 4.11 (.128) | - |
| **Disease duration** | | | | | | |
| *All studies* | 8 (2515) | -0.02 (-0.15, 0.11) | .700 | 32.8 | 10.41 (.167) | - |
| *With outliers removed* | 7 (2498) | -0.03 (-0.13, 0.07) | .464 | 0.0 | 4.62 (.594) | - |
| **Age at onset*** | | | | | | |
| *All studies* | 5 (2269) | -0.10 (-0.24, 0.05) | .131 | 8.3 | 4.36 (.359) | - |
| **LEDD*** | | | | | | |
| *All studies* | 5 (1132) | -0.31 (-0.50, -0.13) | **.009** | 0.0 | 3.59 (.465) | - |
| **UPDRS-III total score (original version)** | | | | | | |
| *All studies* | 7 (1342) | -0.11 (-0.62, 0.40) | .622 | 72.7 | 21.97 (**.001**) | - |
| *With outliers removed* | 6 (1325) | -0.20 (-0.50, 0.10) | .153 | 57.6 | 11.80 (**.038**) | - |

| | | | | | | |
|---|---|---|---|---|---|---|
| **UPDRS-III total score (any version)** | | | | | | |
| *All studies* | 9 | -0.17 | .246 | 73.8 | 30.53 | - |
| | (2592) | (-0.49, 0.15) | | | (< **.001**) | |
| *With outliers removed* | 8 | -0.23 | **.041** | 63.5 | 19.19 | - |
| | (2575) | (-0.44, -0.01) | | | (**.008**) | |

*Note.* CI = confidence interval; *k* = number of studies/effect sizes; LEDD = levodopa equivalent daily dose; *o* = pooled sample size (both groups); UPDRS-III = Unified Parkinson's Disease Rating Scale Part III. Pooled effect size is Hedges' *g* for all variables except gender, which is risk ratio. For Hedges' *g*, negative pooled effect sizes reflect higher values (i.e., older age, longer disease duration) in the non-tremor-dominant (NTD) group relative to the tremor-dominant (TD) group; for risk ratio, values > 1 indicate that tremor-dominant (TD) patients have a greater risk of being men relative to non-tremor-dominant (NTD) patients. $I^2$ is for between-study variance. Egger's test was conducted only when $k \geq 10$ and using Pustejovsky and Rodgers' (2019) revised method. [*]Results for model with outliers removed not reported as no outliers detected. No meta-analyses performed for Movement Disorder Society Unified Parkinson's Disease Rating Scale Part III as $k < 3$ studies. Significant *p*-values (< .05) are highlighted in bold.

**Table S21**

*Results of Continuous Moderator Analyses for Tremor-Dominant (TD) and Non-Tremor-Dominant (NTD) Motor Subtype Groups*

| **Moderator** | ***n*** | ***k*** | ***β* (95% CI)** | ***p*** | **Change in Between-Study $I^2$** |
|---|---|---|---|---|---|
| Sample size | 10 | 25 | 0.00 (-0.00, 0.00) | .800 | 0.00 |
| Publication year | 10 | 25 | 0.01 (-0.02, 0.03) | .520 | -3.69 |
| Pooled mean age | 10 | 25 | -0.00 (-0.04, 0.04) | .920 | 0.00 |

*Note*. CI = confidence interval; *k* = number of effect sizes; *n* = number of unique studies; *β* = regression coefficient. For *β*, positive values reflect a positive association between the moderator and effect size (i.e., larger values of the moderator are associated with larger effect sizes, reflecting better cognitive performance in the tremor-dominant [TD] group relative to the non-tremor-dominant [NTD] group). Change in $I^2$ is the difference in between-study $I^2$ for the model with and without the moderator (larger positive values correspond to greater between-study variance being accounted for by the moderator; negative values suggest that model fit worsened as a consequence of including the moderator). No moderator analyses performed for pooled proportion of men, pooled mean years of education, pooled mean disease duration, pooled mean age at onset, pooled mean levodopa equivalent daily dose (LEDD), pooled mean Unified Parkinson's Disease Rating Scale (UPDRS) Part III score, or pooled mean Movement Disorder Society (MDS)-UPDRS Part III score as $n < 10$ studies. Significant *p*-values (< .05) are highlighted in bold.

**Table S22**

*Results of Categorical Moderator Analyses for Tremor-Dominant (TD) and Non-Tremor-Dominant (NTD) Motor Subtype Groups*

| Moderator | *n* | *k* | Pooled Hedges' *g* (95% CI) | *β* (95% CI) | *p* | *Q* (*p*) | ToM (*p*) |
|---|---|---|---|---|---|---|---|
| **Subtype Method** | | | | | | 30.99 (.096) | 0.48 (.623) |
| *Jankovic** | 4 | 4 | 0.09 (-0.05, 0.23) | | | | |
| *Stebbins* | 2 | 14 | 0.09 (-0.00, 0.18) | 0.00 (-0.17, 0.17) | 1.000 | | |
| *Other* | 4 | 7 | -0.22 (-0.87, 0.43) | -0.31 (-0.97, 0.35) | .344 | | |
| **Cognitive Class** | | | | | | 32.31 (.094) | 1.70 (.205) |
| *Global** | 9 | 10 | 0.08 (-0.01, 0.17) | | .076 | | |
| *Specific* | 3 | 15 | -0.42 (-1.23, 0.38) | -0.50 (-1.30, 0.29) | .205 | | |
| **Cognitive Domain** | | | | | | 21.59 (**.042**) | 1.73 (.219) |
| *Global Cognitive Function** | 9 | 10 | 0.08 (-0.01, 0.18) | | .792 | | |
| *Language* | 2 | 3 | -0.91 (-3.23, 1.42) | -0.99 (-3.31, 1.33) | .370 | | |
| *LTM/Learning – Semantic* | 2 | 2 | -0.42 (-1.29, 0.44) | -0.51 (-1.37, 0.35) | .224 | | |
| **Cognitive Impairment/ Dementia as Exclusion Criterion** | | | | | | 32.42 (.092) | 0.05 (.816) |
| *No** | 5 | 17 | 0.08 (-0.00, 0.15) | | .054 | | |
| *Yes* | 5 | 8 | 0.04 (-0.22, 0.31) | -0.03 (-0.31, 0.25) | .816 | | |

*Note*. CI = confidence interval; *k* = number of effect sizes; LTM = long-term memory; *n* = number of unique studies; STM = short-term memory; ToM = Test of Moderators; *β* = regression coefficient. * denotes reference category for model. The *β* coefficients and corresponding *p*-values indicate whether the pooled effect size for that level of the

moderator differs significantly from the pooled effect size of the reference category; for the reference category, these values indicate whether the pooled effect size differs significantly from zero. For Hedges' $g$, values > 0 indicate poorer cognitive performance among non-tremor-dominant (NTD) patients relative to tremor-dominant (TD) patients. No moderator analyses performed for medication status at time of cognitive assessment(s), for medication status at time of motor assessment(s), for motor subtypes compared on cognition in paper, or for overall risk of bias as $n < 10$ studies. Significant $p$-values (< .05) are highlighted in bold.

**Table S23**

*Results of Confound Analyses for Tremor-Dominant (TD) and Non-Tremor-Dominant (NTD) Motor Subtype Groups*

| **Confound** | ***n*** | ***k*** | ***β* (95% CI)** | ***p*** | **Change in Between-Study $I^2$** |
|---|---|---|---|---|---|
| SMD age | 10 | 25 | 0.07 (-0.61, 0.76) | .828 | 0.00 |

*Note*. CI = confidence interval; *k* = number of effect sizes; *n* = number of unique studies; SMD = standardised mean difference (Hedges' *g*); *β* = regression coefficient. For *β*, positive values reflect a positive association between the confound (moderator) and effect size (i.e., larger values of the confound [moderator] are associated with larger effect sizes, reflecting better cognitive performance in the tremor-dominant [TD] group relative to the non-tremor-dominant [NTD] group). Change in $I^2$ is the difference in between-study $I^2$ for the model with and without the confound (moderator; larger positive values correspond to greater between-study variance being accounted for by the confound [moderator]; negative values suggest that model fit worsened as a consequence of including the confound [moderator]). No confound (moderator) analyses performed for raw difference in proportion of men, SMD in years of education, SMD in disease duration, SMD in age at disease onset, SMD in levodopa equivalent daily dose (LEDD), SMD in Unified Parkinson's Disease Rating Scale (UPDRS) Part III scores, or SMD in Movement Disorder Society (MDS)-UPDRS Part III scores, or SMD in UPDRS Part III scores (any version) as $n < 10$ studies. Significant *p*-values (< .05) are highlighted in bold.

**Results of Postural Instability Gait Disorder vs. Non-Postural Instability Gait Disorder Analyses**

**Table S24**

*Meta-Analyses Comparing Postural Instability Gait Disorder (PIGD) and Non-Postural Instability Gait Disorder (non-PIGD) Motor Subtypes on Demographics and Disease Characteristics*

| **Characteristic** | ***k* (*o*)** | **Pooled effect size (95% CI)** | ***p*** | **$I^2$ (%)** | ***Q* (*p*)** |
|---|---|---|---|---|---|
| **Age** | | | | | |
| *All studies* | 7 (1750) | -0.01 (-0.22, 0.03) | .100 | 0.2 | 6.01 (.422) |
| **Gender (proportion of men)** | | | | | |
| *All studies* | 7 (1750) | 1.09 (1.03, 1.15) | **.009** | 0.0 | 2.17 (.903) |
| **Disease duration*** | | | | | |
| *All studies* | 7 (1750) | -0.29 (-0.51, -0.06) | **.021** | 71.8 | 21.25 (**.002**) |
| **Age at onset*** | | | | | |
| *All studies* | 4 (1377) | -0.10 (-0.32, 0.12) | .245 | 34.9 | 4.61 (.203) |
| **LEDD*** | | | | | |
| *All studies* | 4 (451) | -0.35 (-1.06, 0.37) | .223 | 81.9 | 16.61 (**<.001**) |
| **UPDRS-III total score (original version)*** | | | | | |
| *All studies* | 4 (307) | -0.21 (-0.75, 0.32) | .295 | 40.6 | 5.05 (.168) |
| **UPDRS-III total score (any version)*** | | | | | |
| *All studies* | 6 (1686) | -0.22 (-0.40, -0.04) | **.027** | 30.1 | 7.15 (.210) |

*Note.* CI = confidence interval; *k* = number of studies/effect sizes; LEDD = levodopa equivalent daily dose; *o* = pooled sample size (both groups); UPDRS-III = Unified Parkinson's Disease Rating Scale Part III. Pooled effect size is Hedges' *g* for all variables except gender, which is risk ratio. For Hedges' *g*, negative pooled effect sizes reflect higher values (i.e., older age, longer disease duration) in the postural instability gait disorder (PIGD)

group relative to the non-PIGD group; for risk ratio, values > 1 indicate that non-PIGD patients have a greater risk of men relative to PIGD patients. $I^2$ is for between-study variance. Egger's test not conducted as $k < 10$ for all analyses. *Results for model with outliers removed not reported as no outliers detected. No meta-analyses performed for years of education or Movement Disorder Society (MDS)-UPDRS-III as $k < 3$ studies. Significant *p*-values (< .05) are highlighted in bold.

**Results of Tremor-Dominant vs. Akinetic-Rigid Analyses**

**Table S25**

*Meta-Analyses Comparing Tremor-Dominant (TD) and Akinetic-Rigid (AR) Motor Subtypes on Demographics and Disease Characteristics*

| **Characteristic** | ***k* (*o*)** | **Pooled effect size (95% CI)** | ***p*** | **$I^2$ (%)** | ***Q* (*p*)** | **Egger's test (*p*)** |
|---|---|---|---|---|---|---|
| **Age** | | | | | | |
| *All studies* | 19 (2944) | -0.12 (-0.38, 0.15) | .362 | 87.8 | 147.52 (< **.001**) | .053 |
| *With outliers removed* | 17 (2409) | -0.05 (-0.16, 0.06) | .375 | 0.0 | 15.46 (.491) | .337 |
| **Gender (proportion of men)** | | | | | | |
| *All studies* | 16 (2824) | 1.06 (0.91, 1.23) | .461 | 45.0 | 27.29 (**.027**) | .953 |
| *With outliers removed* | 15 (2734) | 1.08 (0.95, 1.24) | .222 | 33.9 | 21.19 (.097) | .857 |
| **Years of education** | | | | | | |
| *All studies* | 15 (2039) | -0.05 (-0.21, 0.10) | .487 | 63.0 | 37.80 (< **.001**) | .759 |
| *With outliers removed* | 14 (1546) | 0.01 (-0.14, 0.15) | .913 | 32.8 | 19.34 (.113) | .170 |
| **Disease duration** | | | | | | |
| *All studies* | 18 (2441) | -0.11 (-0.28, 0.05) | .169 | 53.2 | 36.35 (**.004**) | .533 |
| *With outliers removed* | 17 (2388) | -0.06 (-0.12, 0.07) | .343 | 37.7 | 25.69 (.059) | .734 |
| **Age at onset** | | | | | | |
| *All studies* | 9 (1934) | -0.18 (-0.67, 0.31) | .422 | 86.8 | 60.62 (< **.001**) | - |
| *With outliers removed* | 8 (1831) | 0.03 (-0.10, 0.15) | .615 | 0.0 | 4.28 (.747) | - |

| | | | | | | |
|---|---|---|---|---|---|---|
| **LEDD** | | | | | | |
| *All studies* | 12 (1147) | -0.38 (-0.77, 0.02) | .058 | 83.4 | 66.22 (< **.001**) | .367 |
| *With outliers removed* | 11 (1044) | -0.24 (-0.47, -0.01) | **.042** | 59.1 | 24.43 (**.007**) | .096 |
| **UPDRS-III total score (original version)*** | | | | | | |
| *All studies* | 17 (2396) | -0.29 (-0.37, -0.20) | **< .001** | 0.0 | 8.59 (.929) | .446 |
| **UPDRS-III total score (any version)†** | | | | | | |
| *All studies* | 19 (3321) | -0.89 (-1.90, 0.11) | .078 | 98.4 | 1128.08 (< **.001**) | .085 |

*Note.* CI = confidence interval; *k* = number of studies/effect sizes; LEDD = levodopa equivalent daily dose; MDS-UPDRS-III = Movement Disorder Society Unified Parkinson's Disease Rating Scale Part III; *o* = pooled sample size (both groups); UPDRS-III = Unified Parkinson's Disease Rating Scale Part III. Pooled effect size is Hedges' *g* for all variables except gender, which is risk ratio. For Hedges' *g*, negative pooled effect sizes reflect higher values (i.e., older age, longer disease duration) in the akinetic-rigid group relative to the tremor-dominant group; for risk ratio, values > 1 indicate that tremor-dominant patients have a greater risk of being men relative to akinetic-rigid patients. $I^2$ is for between-study variance. Egger's test was conducted only when $k \geq 10$ and using Pustejovsky and Rodgers' (2019) revised method. *Results for model with outliers removed not reported as no outliers detected. †Results for model with outliers removed not reported as outliers were both studies reporting MDS-UPDRS-III and no other studies reported MDS-UPDRS-III, so model with outliers removed was identical to model for UPDRS-III (original version). No meta-analyses were performed for Movement Disorder Society Unified Parkinson's Disease Rating Scale Part III as $k < 3$ studies. Significant *p*-values (< .05) are highlighted in bold.

**Table S26**

*Results of Dose-Effect Analysis for Pairwise Comparison Between Tremor-Dominant (TD) and Akinetic-Rigid (AR) Motor Subtype Groups*

| Moderator | *n* | *k* | *β* (95% CI) | *p* | Change in Between-Study $I^2$ |
|---|---|---|---|---|---|
| SMD in UPDRS tremor subscore | 6 | 35 | -0.06 (-0.51, 0.39) | .782 | 0.00 |

*Note*. CI = confidence interval; *k* = number of effect sizes; *n* = number of unique studies; SMD = standardised mean difference (Hedges' *g*); UPDRS = Unified Parkinson's Disease Rating Scale (any version); $\beta$ = regression coefficient. For $\beta$, positive values reflect a positive association between the moderator and effect size (i.e., larger values of the moderator are associated with larger effect sizes, reflecting better cognitive performance in the tremor-dominant [TD] group relative to the akinetic-rigid [AR] group). Change in $I^2$ is the difference in between-study $I^2$ for the model with and without the moderator (larger positive values correspond to greater between-study variance being accounted for by the moderator; negative values suggest that model fit worsened as a consequence of including the moderator). No results reported for SMD in UPDRS AR subscore as $n < 10$ studies. No results are reported for SMD in UPDRS tremor subscore when outliers removed (identified from the full model, using a residuals approach) as none of the studies with outliers reported UPDRS tremor subscore data; consequently, the results of our dose-effect analysis were the same regardless of whether these outliers were retained or removed from the data set. Significant *p*-values (< .05) are highlighted in bold.

**Figure S11**

*Relationship Between Sample Size and Effect Size for Cognitive Difference Between Tremor-Dominant (TD) and Akinetic-Rigid (AR) Motor Subtype Groups*

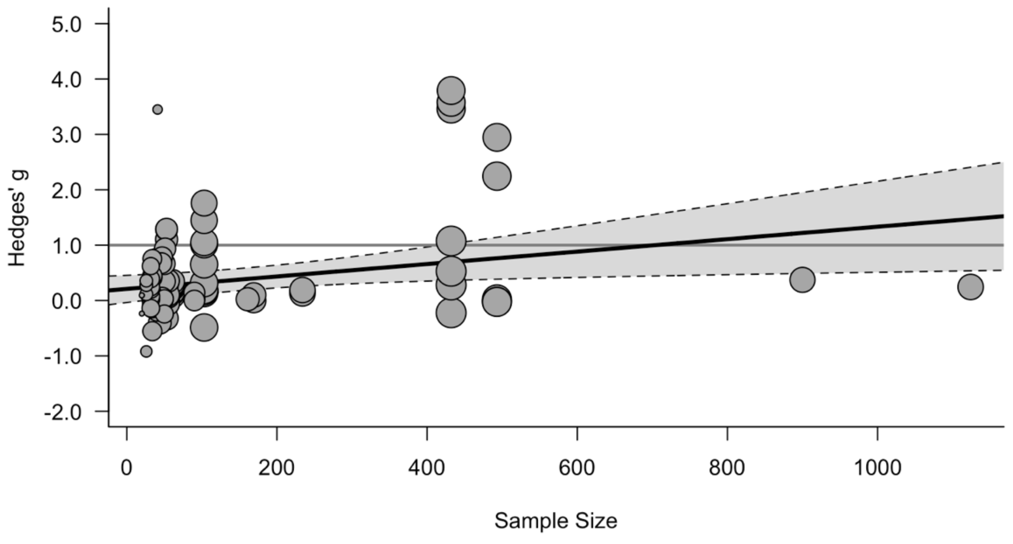


*Note*. For Hedges' *g*, values > 0 indicate better cognitive performance among tremor-dominant (TD) patients relative to akinetic-rigid (AR) patients.

**Table S27**

*Results of Continuous Moderator Analyses for Tremor-Dominant (TD) and Akinetic-Rigid (AR) Motor Subtype Groups (Outliers Removed – Residuals Approach)*

| **Moderator** | ***n*** | ***k*** | ***β* (95% CI)** | ***p*** | **Change in Between-Study $I^2$** |
|---|---|---|---|---|---|
| Sample size | 20 | 108 | 0.00 (-0.00, 0.00) | .798 | 0.00 |
| Publication year | 20 | 108 | -0.01 (-0.03, 0.02) | .624 | 0.00 |
| Pooled mean age | 18 | 98 | 0.01 (-0.02, 0.04) | .419 | 0.00 |
| Pooled proportion of men | 15 | 73 | 0.59 (-0.63, 1.81) | .340 | 0.00 |
| Pooled mean years of education | 15 | 85 | -0.00 (-0.05, 0.04) | .899 | 0.00 |
| Pooled mean disease duration | 18 | 101 | 0.02 (-0.01, 0.05) | .200 | 0.00 |
| Pooled mean age at onset | 18 | 101 | 0.00 (-0.02, 0.02) | .845 | 0.00 |
| Pooled mean LEDD | 12 | 74 | 0.00 (-0.00, 0.00) | .186 | 0.00 |
| Pooled mean UPDRS-III (original) | 17 | 92 | -0.00 (-0.01, 0.01) | .931 | 0.00 |

*Note*. CI = confidence interval; *k* = number of effect sizes; *n* = number of unique studies; UPDRS-III = Unified Parkinson's Disease Rating Scale Part III; *β* = regression coefficient. For *β*, positive values reflect a positive association between the moderator and effect size (i.e., larger values of the moderator are associated with larger effect sizes, reflecting better cognitive performance in the tremor-dominant [TD] group relative to the akinetic-rigid [AR] group). Change in $I^2$ is the difference in between-study $I^2$ for the model with and without the moderator (larger positive values correspond to greater between-study variance being accounted for by the moderator; negative values suggest that model fit worsened as a consequence of including the moderator). No moderator analyses performed for pooled mean Movement Disorder Society (MDS)-UPDRS Part III score as *n* < 10 studies. Significant *p*-values (< .05) are highlighted in bold.

**Table S28**

*Results of Categorical Moderator Analyses for Tremor-Dominant (TD) and Akinetic-Rigid (AR) Motor Subtype Groups (Outliers Removed – Residuals Approach)*

| **Moderator** | ***n*** | ***k*** | **Pooled Hedges' *g* (95% CI)** | ***β* (95% CI)** | ***p*** | ***Q* (*p*)** | **ToM (*p*)** |
|---|---|---|---|---|---|---|---|
| **Subtype Method** | | | | | | 361.46 (< **.001**) | 0.482 (.489) |
| *Kang** | 11 | 64 | 0.17 (0.03, 0.31) | | **.014** | | |
| *Other* | 9 | 44 | 0.25 (0.08, 0.41) | 0.07 (-0.14, 0.29) | .489 | | |
| **Cognitive Class** | | | | | | 339.19 (< **.001**) | 0.783 (.378) |
| *Global** | 20 | 30 | 0.16 (0.03, 0.29) | | **.019** | | |
| *Specific* | 12 | 78 | 0.25 (0.10, 0.39) | 0.08 (-0.11, 0.28) | .378 | | |
| **Cognitive Domain** | | | | | | 256.59 (< **.001**) | 1.53 (.120) |
| *Global Cognitive Function** | 20 | 30 | 0.16 (0.04, 0.28) | | **.009** | | |
| *Executive Function – Cognitive Flexibility* | 4 | 4 | 0.29 (-0.07, 0.65) | 0.13 (-0.24, 0.51) | .489 | | |
| *Executive Function – Cognitive Inhibition* | 4 | 4 | 0.40 (0.06, 0.73) | 0.24 (-0.10, 0.58) | .170 | | |
| *Executive Function – Working Memory* | 5 | 8 | 0.36 (0.08, 0.64) | 0.20 (-0.10, 0.50) | .181 | | |
| *Composite IQ* | 2 | 2 | 0.36 (-0.16, 0.88) | 0.21 (-0.32, 0.73) | .439 | | |
| *Language* | 3 | 3 | 0.58 (-0.14, 1.30) | 0.42 (-0.31, 1.15) | .252 | | |
| *LTM – Lexical* | 2 | 2 | 0.09 (-0.44, 0.62) | -0.07 (-0.61, 0.47) | .791 | | |

| | | | | | | | |
|---|---|---|---|---|---|---|---|
| *LTM – Semantic* | 2 | 2 | 0.86 (0.38, 1.33) | 0.70 (0.21, 1.18) | **.005** | | |
| *LTM – Visuospatial* | 4 | 5 | 0.30 (-0.05, 0.65) | 0.14 (-0.22, 0.50) | .441 | | |
| *STM – Lexical* | 3 | 3 | 0.05 (-0.36, 0.47) | -0.11 (-0.53, 0.32) | .621 | | |
| *STM – Numerical* | 2 | 2 | -0.38 (-0.80, 0.05) | -0.53 (-0.97, -0.10) | **.018** | | |
| *STM – Visuospatial* | 4 | 7 | 0.04 (-0.26, 0.33) | -0.12 (-0.43, 0.19) | .434 | | |
| *Processing Speed* | 7 | 14 | 0.25 (-0.02, 0.51) | 0.09 (-0.20, 0.38) | .548 | | |
| *Visuospatial Abilities – Construction* | 2 | 2 | 0.23 (-0.26, 0.72) | 0.08 (-0.42, 0.57) | .764 | | |
| *Visuospatial Abilities – Perception* | 2 | 2 | 0.48 (-0.05, 1.02) | 0.32 (-0.22, 0.86) | .236 | | |
| **Medication Status for Cognitive Assessment(s)** | | | | | | 328.76 (< **.001**) | 0.54 (.656) |
| *De Novo*[*] | 4 | 17 | 0.25 (0.05, 0.46) | | **.016** | | |
| *ON* | 5 | 37 | 0.26 (0.04, 0.47) | 0.01 (-0.29, 0.30) | .965 | | |
| *OFF* | 4 | 18 | 0.05 (-0.23, 0.34) | -0.20 (-0.55, 0.15) | .263 | | |
| *ON or OFF* | 3 | 17 | 0.25 (-0.04, 0.53) | -0.00 (-0.36, 0.35) | .978 | | |
| **Medication Status for Motor Assessment(s)** | | | | | | 330.86 (< **.001**) | 1.29 (.283) |
| *De Novo*[*] | 4 | 17 | 0.25 (0.06, 0.45) | | **.011** | | |
| *ON* | 4 | 27 | 0.30 (0.06, 0.53) | 0.04 (-0.26, 0.35) | .789 | | |
| *OFF* | 7 | 38 | 0.05 (-0.13, 0.23) | -0.20 (-0.47, 0.06) | .125 | | |
| *ON or OFF* | 3 | 17 | 0.24 (-0.04, 0.52) | -0.01 (-0.35, 0.33) | .945 | | |

| | | | | | | | |
|---|---|---|---|---|---|---|---|
| **Cognitive Impairment/Dementia as Exclusion Criterion** | | | | | | 360.99 (< **.001**) | 1.13 (.290) |
| *No** | 10 | 35 | 0.26 (0.12, 0.41) | | **.001** | | |
| *Yes* | 10 | 73 | 0.15 (0.01, 0.29) | -0.11 (-0.31, 0.09) | .290 | | |
| **Motor Subtypes Compared on Cognition in Paper** | | | | | | 348.23 (< **.001**) | 2.75 (.100) |
| *No** | 3 | 13 | 0.49 (0.13, 0.85) | | **.009** | | |
| *Yes* | 19 | 95 | 0.17 (0.06, 0.28) | -0.312 (-0.69, 0.06) | .100 | | |
| **Overall Risk of Bias** | | | | | | 356.59 (< **.001**) | 0.26 (.769) |
| *High** | 9 | 41 | 0.20 (0.02, 0.37) | | **.026** | | |
| *Moderate* | 6 | 26 | 0.26 (0.08, 0.44) | 0.06 (-0.18, 0.31) | .604 | | |
| *Low* | 5 | 41 | 0.17 (-0.02, 0.36) | -0.03 (-0.29, 0.23) | .837 | | |

*Note*. CI = confidence interval; IQ = intelligence quotient; *k* = number of effect sizes; LTM = long-term memory; *n* = number of unique studies; STM = short-term memory; ToM = Test of Moderators; $\beta$ = regression coefficient. * denotes reference category for model. The $\beta$ coefficients and corresponding *p*-values indicate whether the pooled effect size for that level of the moderator differs significantly from the pooled effect size of the reference category; for the reference category, these values indicate whether the pooled effect size differs significantly from zero. For Hedges' *g*, values > 0 indicate poorer cognitive performance among akinetic-rigid (AR) patients relative to tremor-dominant (TD) patients. Significant *p*-values (< .05) are highlighted in bold.

**Figure S12**

*Relationship Between Standardised Mean Difference (SMD) in Unified Parkinson's Disease Rating Scale Part III (Any Version) and Effect Size for Cognitive Difference Tremor-Dominant (TD) and Akinetic-Rigid (AR) Motor Subtype*

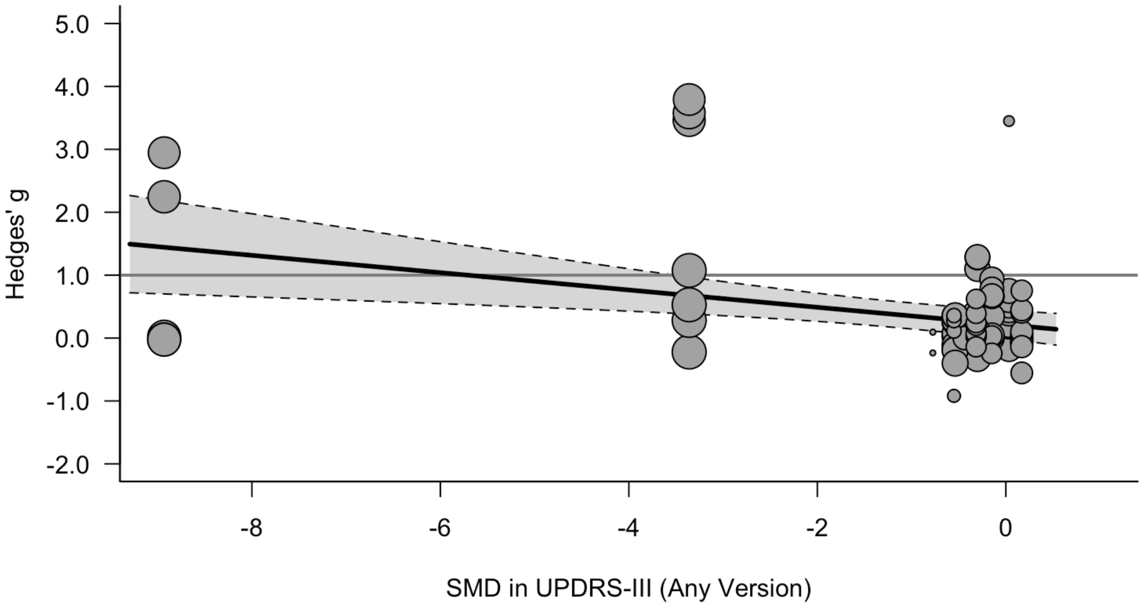


*Note.* SMD = standardised mean difference (Hedges' *g*). UPDRS-III = Unified Parkinson's Disease Rating Scale. For Hedges' *g*, values > 0 indicate poorer cognitive performance among akinetic-rigid (AR) patients relative to tremor-dominant (TD) patients.

**Table S29**

*Results of Confound Analyses for Tremor-Dominant (TD) and Akinetic-Rigid (AR) Motor Subtype Groups (Outliers Removed – Residuals Approach)*

| **Confound** | ***n*** | ***k*** | ***β* (95% CI)** | ***p*** | **Change in Between-Study $I^2$** |
|---|---|---|---|---|---|
| SMD age | 18 | 98 | -0.04 (-0.19, 0.12) | .620 | 0.00 |
| Difference in proportion of men | 15 | 73 | -0.35 (-1.09, 0.39) | .349 | 0.00 |
| SMD years of education | 15 | 85 | 0.32 (-0.02, 0.66) | .069 | 0.00 |
| SMD disease duration | 17 | 92 | -0.19 (-0.46, 0.08) | .170 | 0.00 |
| SMD LEDD | 12 | 74 | -0.12 (-0.30, 0.07) | .214 | 0.00 |
| SMD UPDRS-III (original) | 17 | 92 | 0.12 (-0.36, 0.60) | .621 | 0.00 |
| SMD UPDRS-III (any version) | 19 | 99 | 0.01 (-0.02, 0.05) | .518 | 0.00 |

*Note*. CI = confidence interval; *k* = number of effect sizes; LEDD = levodopa equivalent daily dose; *n* = number of unique studies; UPDRS-III = Unified Parkinson's Disease Rating Scale Part III; SMD = standardised mean difference (Hedges' *g*); *β* = regression coefficient. For *β*, positive values reflect a positive association between the confound (moderator) and effect size (i.e., larger values of the confound [moderator] are associated with larger effect sizes, reflecting better cognitive performance in the tremor-dominant [TD] group relative to the akinetic-rigid [AR] group). Change in $I^2$ is the difference in between-study $I^2$ for the model with and without the confound (moderator; larger positive values correspond to greater between-study variance being accounted for by the confound [moderator]; negative values suggest that model fit worsened as a consequence of including the confound [moderator]). No confound (moderator) analyses performed for SMD in age at disease onset or SMD in Movement Disorder Society (MDS)-UPDRS Part III scores as $n < 10$ studies. Significant *p*-values (< .05) are highlighted in bold.

## Results of Tremor-Dominant vs. Mixed Analyses

### *Demographics and Disease Characteristics*

Our traditional meta-analyses comparing tremor-dominant (TD) and mixed (MX) motor subtype groups on demographics and disease characteristics revealed no significant differences (Table S30).

**Table S30**

*Meta-Analyses Comparing Tremor-Dominant (TD) and Mixed (MX) Motor Subtypes on Demographics and Disease Characteristics*

| Characteristic | *k* (*o*) | Pooled effect size (95% CI) | *p* | $I^2$ (%) | *Q* (*p*) |
|---|---|---|---|---|---|
| **Age*** | | | | | |
| *All studies* | 5 (984) | -0.07 (-0.32, 0.19) | .512 | 0.0 | 3.52 (.476) |
| **Gender (proportion of men)*** | | | | | |
| *All studies* | 5 (984) | 0.95 (0.83, 1.08) | .299 | 0.0 | 1.34 (.854) |
| **Years of education*** | | | | | |
| *All studies* | 3 (261) | -0.18 (-1.03, 0.68) | .469 | 33.4 | 3.00 (.223) |
| **Disease duration*** | | | | | |
| *All studies* | 5 (984) | -0.19 (-0.62, 0.24) | .282 | 57.1 | 9.33 (.053) |
| **Age at onset*** | | | | | |
| *All studies* | 4 (949) | 0.12 (-0.06, 0.30) | .119 | 0.0 | 0.90 (.824) |
| **UPDRS-III total score (original version)*** | | | | | |
| *All studies* | 5 (984) | -0.46 (-1.25, 0.33) | .183 | 73.0 | 14.82 (**.005**) |

*Note.* CI = confidence interval; *k* = number of studies/effect sizes; *o* = pooled sample size (both groups); UPDRS-III = Unified Parkinson's Disease Rating Scale Part III. Pooled effect size is Hedges' *g* for all variables except gender, which is risk ratio. For Hedges' *g*, negative pooled effect sizes reflect higher values (i.e., older age, longer disease duration) in the mixed group relative to tremor-dominant group; for risk ratio, values > 1 indicate that

tremor-dominant patients have a greater risk of being men relative to mixed patients. $I^2$ is for between-study variance. Egger's test not conducted as $k < 10$ for all analyses. [*]Results for model with outliers removed not reported as no outliers detected. No meta-analyses performed for LEDD as $k < 3$ studies. No meta-analyses performed for MDS-UPDRS-III total score or UPDRS-III total score (any version) as no studies reported MDS-UPDRS-III data. Significant *p*-values (< .05) are highlighted in bold.

### *Cognition*

**Main Analyses.** Our multi-level meta-analysis of 17 effect sizes from 5 studies produced a non-significant, negligible pooled effect size estimate, indicating no difference in cognitive performance between TD and MX motor subtype groups (Table S31). Removal of outliers (using both methods) resulted in a slight increase in the pooled effect size estimate, but it remained non-significant and of negligible magnitude. Inspection of sunset (power-enhanced) funnel plots (Figure S13) indicated that all included studies were highly underpowered (median power = 5.5% for full model) to detect an effect. No dose-effect, moderator, or confound analyses were performed due to an insufficient number of studies ($n < 10$). With respect to cognitive status, one study (Zhang et al., 2016) compared rates of MCI among TD and MX patients; this study found comparable rates of MCI among both subtype groups, with a slightly higher rate observed in the MX group (60.00%) relative to the TD group (50.00%).

**Table S31**

*Results of Multi-Level Meta-Analyses of Cognitive Performance in Tremor-Dominant (TD) and Mixed (MX) Motor Subtype Groups*

| Subtype Pair | Model | *n* (*k*) | Pooled Hedges' *g* (95% CI) | *p* | Within-Study $I^2$ (%) (*p*) | Between-Study $I^2$ (%) (*p*) | *Q* (*p*) | Multi-level Egger's test (*p*) |
|---|---|---|---|---|---|---|---|---|
| TD vs. MX | All studies | 5 (17) | 0.09 (-0.14, 0.31) | .419 | 1.29 (.500) | 18.05 (.204) | 13.71 (.620) | .366 |
| | With outliers removed – residuals approach | 5 (14) | 0.11 (-0.09, 0.31) | .267 | 4.64 (.500) | 10.13 (.329) | 8.37 (.819) | .451 |
| | With outliers removed – Cook's distance approach† | 5 (15) | 0.10 (-0.09, 0.29) | .277 | 3.59 (.500) | 6.24 (.500) | 8.37 (.869) | - |

*Note*. CI = confidence interval; *k* = number of effect sizes; MX = mixed; *n* = number of unique studies; TD = tremor-dominant. For Hedges' *g*, values > 0 indicate poorer cognitive performance among mixed (MX) patients relative to tremor-dominant (TD) patients. † Multi-level Egger's test not reported as model failed to reach convergence. Significant *p*-values (< .05) are highlighted in bold.

**Figure S13**

*Sunset (Power-Enhanced) Funnel Plots for Difference in Cognitive Performance Between Tremor-Dominant (TD) and Mixed (MX) Motor Subtype Groups*

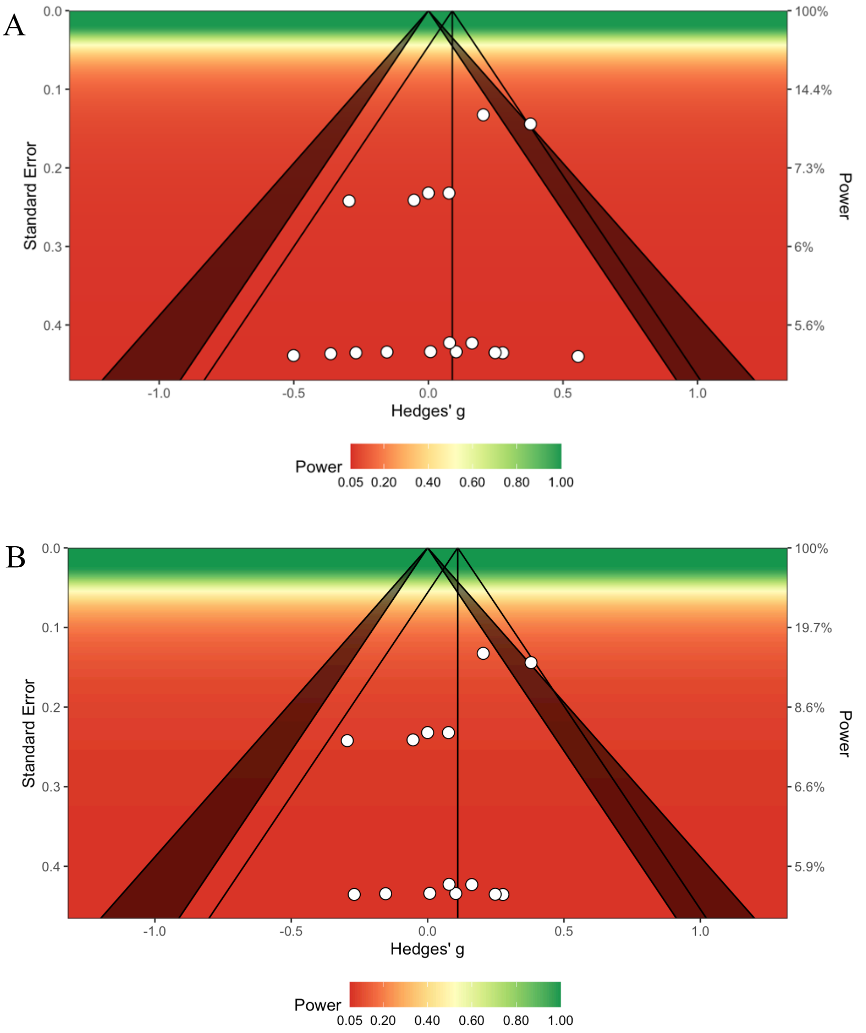


*Note.* A: Full model (no outliers removed). B. Model with outliers removed (residuals approach). For Hedges' *g*, values > 0 indicate poorer cognitive performance among mixed (MX) patients relative to tremor-dominant (TD) patients.

## Results of Akinetic-Rigid vs. Mixed Analyses

### *Demographics and Disease Characteristics*

Results of our traditional meta-analyses comparing akinetic-rigid (AR) and mixed (MX) motor subtype groups on demographics and disease characteristics are reported in Table S32. With the exception of age and age at time of disease onset, all meta-analyses were non-significant. The pooled effect sizes for both age and age at time of disease onset were of negligible magnitude but reflected older age (at time of disease onset and at time of study participation) among AR patients relative to MX patients.

**Table S32**

*Meta-Analyses Comparing Akinetic-Rigid (AR) and Mixed (MX) Motor Subtypes on Demographics and Disease Characteristics*

| **Characteristic** | ***k*** **(*o*)** | **Pooled effect size (95% CI)** | ***p*** | $I^2$ **(%)** | ***Q*** **(*p*)** |
|---|---|---|---|---|---|
| **Age*** | | | | | |
| *All studies* | 5 (2093) | 0.13 (0.06, 0.21) | **.009** | 0.0 | 1.33 (.856) |
| **Gender (proportion of men)*** | | | | | |
| *All studies* | 5 (2093) | 0.87 (0.57, 1.30) | .381 | 54.6 | 8.81 (.066) |
| **Years of education*** | | | | | |
| *All studies* | 3 (307) | -0.18 (-1.19, 0.83) | .524 | 46.9 | 3.77 (.152) |
| **Disease duration*** | | | | | |
| *All studies* | 5 (2093) | 0.02 (-0.07, 0.12) | .542 | 0.0 | 2.18 (.702) |
| **Age at onset*** | | | | | |
| *All studies* | 4 (2052) | 0.10 (0.05, 0.15) | **.009** | 0.0 | 0.33 (.954) |
| **UPDRS-III total score (original version)*** | | | | | |
| *All studies* | 5 (2093) | -0.07 (-0.60, 0.46) | .734 | 67.0 | 12.12 (**.017**) |

*Note.* CI = confidence interval; *k* = number of studies/effect sizes; *o* = pooled sample size (both groups); UPDRS-III = Unified Parkinson's Disease Rating Scale Part III. Pooled effect size is Hedges' *g* for all variables except gender, which is risk ratio. For Hedges' *g*, negative pooled effect sizes reflect higher values (i.e., older age, longer disease duration) in the mixed group relative to the akinetic-rigid group; for risk ratio, values > 1 indicate that akinetic-rigid patients have a greater risk of being men relative to mixed patients. $I^2$ is for between-study variance. Egger's test not conducted as $k < 10$ for all analyses. *Results for model with outliers removed not reported as no outliers detected. No meta-analyses performed for LEDD as $k < 3$ studies. No meta-analyses performed for MDS-UPDRS-III total score or UPDRS-III total score (any version) as no studies reported MDS-UPDRS-III data. Significant *p*-values (< .05) are highlighted in bold.

### *Cognition*

**Main Analyses.** Our multi-level meta-analysis of 17 effect sizes from 5 studies produced a non-significant, negligible pooled effect size estimate, suggesting no difference in cognitive performance between AR and MX motor subtype groups (Table S33). Removal of outliers identified using residuals resulted in a slight decrease in the (absolute) magnitude of the pooled effect. Of note, whilst the pooled effect size estimate remained of negligible magnitude following removal of outliers identified using Cook's distance, the estimate for this model was in the opposite direction to our other models. Inspection of sunset (power-enhanced) funnel plots (Figure S14) indicated that all included studies were highly underpowered (median power = 5.1% for full model) to detect an effect. No dose-effect, moderator, or confound analyses were performed due to an insufficient number of studies ($n < 10$). With regards to cognitive status, only one study (Zhang et al., 2016) examined rates of MCI between AR and MX subtype groups and reported a negligible difference in MCI prevalence between AR (57.44%) and MX (60.00%) patients.

**Table S33**

*Results of Multi-Level Meta-Analyses of Cognitive Performance in Akinetic-Rigid (AR) and Mixed (MX) Motor Subtype Groups*

| **Subtype Pair** | **Model** | ***n* (*k*)** | **Pooled Hedges' *g* (95% CI)** | ***p*** | **Within-Study $I^2$ (%) (*p*)** | **Between-Study $I^2$ (%) (*p*)** | ***Q* (*p*)** | **Multi-level Egger's test (*p*)** |
|---|---|---|---|---|---|---|---|---|
| AR vs. MX | All studies | 5 (17) | -0.04 (-0.13, 0.05) | .330 | 5.09 (.500) | 4.63 (.500) | 10.74 (.825) | .582 |
| | With outliers removed – residuals approach | 5 (13) | -0.04 (-0.13, 0.05) | .351 | 1.11 (.500) | 6.49 (.500) | 4.75 (.966) | .662 |
| | With outliers removed – Cook's distance approach | 4 (15) | 0.11 (-0.34, 0.12) | .323 | 4.11 (.500) | 5.55 (.500) | 9.55 (.794) | .856 |

*Note*. AR = akinetic-rigid; CI = confidence interval; *k* = number of effect sizes; MX = mixed; *n* = number of unique studies. For Hedges' *g*, values < 0 indicate poorer cognitive performance among akinetic-rigid (AR) patients relative to mixed (MX) patients. Significant *p*-values (< .05) are highlighted in bold.

**Figure S14**

*Sunset (Power-Enhanced) Funnel Plots for Difference in Cognitive Performance Between Akinetic-Rigid (AR) and Mixed (MX) Motor Subtype Groups*

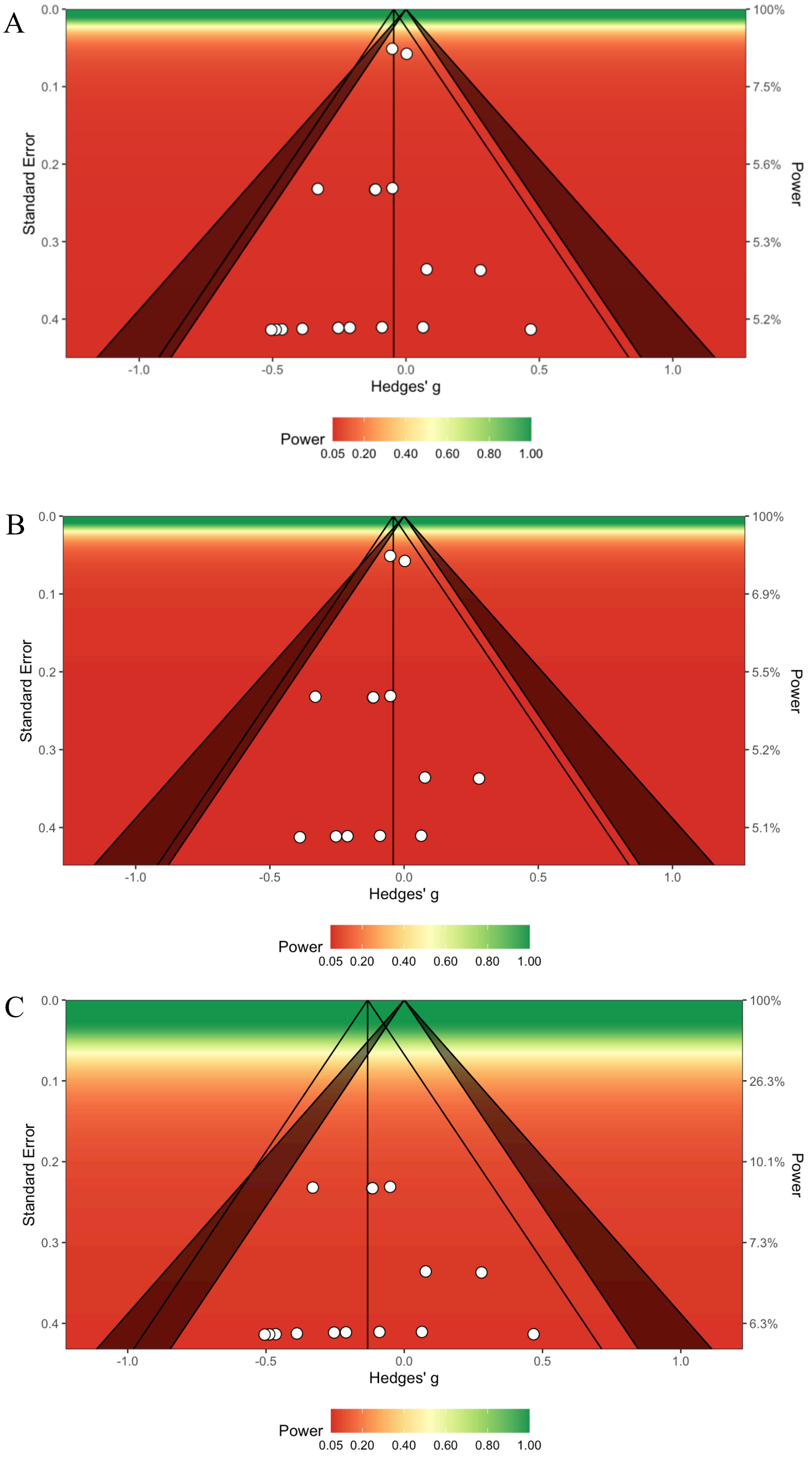


*Note.* A: Full model (no outliers removed). B. Model with outliers removed (residuals approach). C. Model with outliers removed (Cook's distance approach). For Hedges' *g*, values < 0 indicate poorer cognitive performance among akinetic-rigid (AR) patients relative to mixed (MX) patients.

**Results of Tremor vs. Bradykinesia Analyses**

Five studies compared the cognitive performance of a tremor (TR) motor subtype to that of a bradykinesia (BR) motor subtype. In three studies (Dewey et al., 2012; Erro et al., 2013; Katzen et al., 2006), patients were subtyped according to whether their *initial* motor symptom was tremor or bradykinesia, as determined via medical records alone or patient-report verified by chart review. In another study (Vakil & Herishanu-Naaman, 1998), patients were classified by a senior neurologist according to whether tremor or bradykinesia was their most predominant motor symptom at the time of study participation. Only one study (Huber et al., 1991) used a more rigorous approach, wherein UPDRS scores (original version; Fahn et al., 1987) were used to classify patients as either tremor-predominant (≥2 on rest tremor and ≤1 on rigidity and bradykinesia items) or rigid/bradykinetic (≥2 on rigidity and bradykinesia items and ≤1 on rest tremor).

None of our traditional meta-analyses comparing TR and BR subtypes on demographics and disease characteristics reached statistical significance (Table S34). Wide 95% confidence intervals were observed around all pooled effect sizes, indicating a high level of imprecision in these estimates, likely due to the low number of included studies ($k = 4$) and small pooled sample size ($o = 260$). With respect to cognitive function, all five studies reported data from continuous cognitive measures; none reported cognitive status data. All of our multi-level meta-analyses (both with and without outliers removed) comparing cognitive function between the TR and BR subtypes produced negligible, non-significant pooled effect size estimates with wide confidence intervals (Table S35). Multi-level Egger's tests indicated presence of publication bias in all three models. Inspection of sunset (power-enhanced) funnel plots (Supplementary Material 2, Figure S15) revealed that all included studies were highly underpowered (median power = 9.6% for full model) to detect an effect. No dose-effect, moderator, or confound analyses were performed due to an insufficient number of studies ($n < 10$).

**Table S34**

*Meta-Analyses Comparing Tremor (TR) and Bradykinesia (BR) Motor Subtypes on Demographics and Disease Characteristics*

| Characteristic | *k* (*o*) | Pooled effect size (95% CI) | *p* | $I^2$ (%) | *Q* (*p*) |
|---|---|---|---|---|---|
| **Age*** | | | | | |
| *All studies* | 4 (260) | 0.24 (-0.41, 0.89) | .320 | 49.9 | 5.99 (.112) |
| **Gender (proportion of men)*** | | | | | |
| *All studies* | 4 (260) | 1.00 (0.82, 1.21) | .956 | 0.0 | 1.02 (.798) |
| **Years of education*** | | | | | |
| *All studies* | 4 (260) | 0.07 (-0.31, 0.45) | .611 | 0.0 | 2.39 (.495) |
| **Disease duration*** | | | | | |
| *All studies* | 4 (260) | -0.01 (-0.59, 0.56) | .952 | 41.0 | 5.08 (.166) |

*Note.* CI = confidence interval; *k* = number of studies/effect sizes; LEDD = levodopa equivalent daily dose; MDS-UPDRS-III = Movement Disorder Society Unified Parkinson's Disease Rating Scale Part III; *o* = pooled sample size (both groups); UPDRS-III = Unified Parkinson's Disease Rating Scale Part III. Pooled effect size is Hedges' *g* for all variables except gender, which is risk ratio. For Hedges' *g*, negative pooled effect sizes reflect higher values (i.e., older age, longer disease duration) in the bradykinesia (BR) group relative to the tremor (TR) group; for risk ratio, values > 1 indicate that tremor (TR) patients have a greater risk of being men relative to bradykinesia (BR) patients. $I^2$ is for between-study variance. Egger's test was conducted only when $k \geq 10$ and using Pustejovsky and Rodgers' (2019) revised method. * Results for model with outliers removed are not reported as no outliers were detected. Significant *p*-values (< .05) are highlighted in bold.

**Table S35**

*Results of Multi-Level Meta-Analyses of Cognitive Performance in Tremor (TR) and Bradykinesia (BR) Motor Subtype Groups*

| **Subtype Pair** | **Model** | ***n* (*k*)** | **Pooled Hedges' *g* (95% CI)** | ***p*** | **Within-Study $I^2$ (%) (*p*)** | **Between-Study $I^2$ (%) (*p*)** | ***Q* (*p*)** | **Multi-level Egger's test (*p*)** |
|---|---|---|---|---|---|---|---|---|
| TR vs. BR | All studies | 5 (49) | 0.13 (-0.18, 0.44) | .395 | 47.02 (< **.001**) | 25.60 (.052) | 156.86 (< **.001**) | **.001** |
| | With outliers removed – residuals approach | 5 (44) | 0.08 (-0.19, 0.34) | .575 | 19.41 (**.019**) | 33.80 (**.048**) | 74.97 (**.002**) | **.003** |
| | With outliers removed – Cook's distance approach | 5 (39) | 0.15 (-0.19, 0.48) | .380 | 5.65 (.500) | 58.41 (**.002**) | 40.12 (.377) | **.018** |

*Note*. BR = bradykinesia; CI = confidence interval; *k* = number of effect sizes; *n* = number of unique studies; TR = tremor. For Hedges' *g*, values > 0 indicate poorer cognitive performance among bradykinesia (BR) patients relative to tremor (TR) patients. Significant *p*-values (< .05) are highlighted in bold.

**Figure S15**

*Sunset (Power-Enhanced) Funnel Plots for Difference in Cognitive Performance Between Tremor (TR) and Bradykinesia (BR) Motor Subtype Groups*

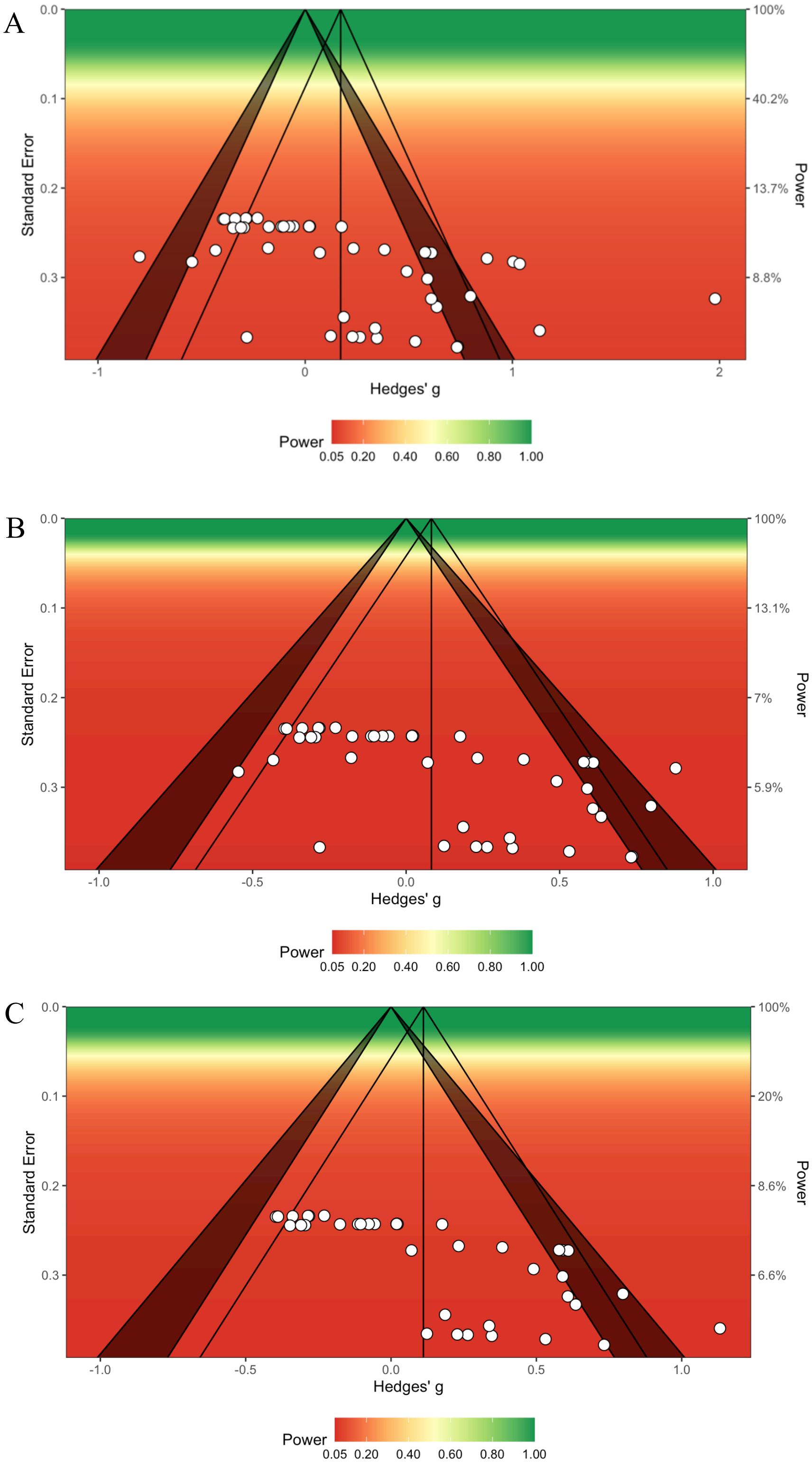


*Note.* A: Full model (no outliers removed). B. Model with outliers removed (residuals approach). C. Model with outliers removed (Cook's distance approach). For Hedges' *g*, values > 0 indicate better cognitive performance among tremor (TR) patients relative to bradykinesia (BR) patients.

**Results of Bradykinesia vs. No-Bradykinesia Synthesis**

One study (Tomer et al., 2002) compared the cognitive performance of PD patients with and without bradykinesia on a single task, the Wisconsin Card Sorting Test (WCST). The authors reported that patients were classified into bradykinesia and no-bradykinesia motor subtype groups according to UPDRS scores (original version; Fahn et al., 1987), but they did not specify the specific item(s) used. In general, no-bradykinesia patients performed better than bradykinesia patients on the WCST. Compared to patients with bradykinesia, patients without bradykinesia achieved more categories ($g = 0.53$) and made fewer total errors on the task ($g = 0.44$), but no difference was observed on set loss ($g = 0.05$). Of note, a large effect size was reported for number of perseverative errors ($g = 0.82$), indicating that bradykinetic patients may experience particularly marked problems with cognitive perseveration.

**Results of Tremor vs. No-Tremor Synthesis**

Two studies (Poletti et al., 2012; Rana et al., 2012) formed motor subtype groups based on the presence (TR-Y) or absence (TR-N) of tremor. In Poletti et al. (2012), the presence of tremor was determined using items 20 and 21 of the UPDRS-III, administered at the time of study participation, while Rana et al. (2012) based their subtype classification on retrospective chart analysis of each patient's medical records. Demographics and disease characteristics were only available for one study (Poletti et al., 2012); these data indicated negligible-to-small subtype differences between TR-Y and TR-N groups. Of note, consistent with our analyses of other subtype pairs including a tremor-based subtype group, a small effect size was found, indicating greater overall motor impairment (as assessed by UPDRS-III total score) in the TR-N group relative to the TR-Y group ($g = 0.261$).

With respect to cognitive function, Poletti et al. (2012) administered a comprehensive cognitive assessment battery comprising 15 tasks, reporting the results from these individual tasks as well as MCI rates based on task performance (defined as scores on at least two tasks falling below 1.5 standard deviations of the mean). Rana et al. (2012) reported dementia rates in both subtype groups, as diagnosed by a neurologist according to Diagnostic and Statistical Manual of Mental Disorders criteria (DSM-IV; American Psychiatric Association, 1994). Among Poletti and colleagues' (2012) tasks, two global (Mini-Mental State Examination [MMSE], Frontal Assessment Battery [FAB]) and 13 domain-specific (e.g., digit span, phonemic fluency) tasks were administered. Effect sizes for the global measures ranged from $g = 0.14$ (MMSE) to $g = 0.22$ (FAB), with TR-Y patients outperforming TR-N patients on both tasks. Consistent with this, both studies' cognitive status data indicated greater cognitive impairment among TR-N patients relative to TR-Y patients; compared to TR-N patients, TR-Y patients had 70.0% relative reduced risk of MCI (Poletti et al., 2012) and 57.2% relative reduced risk of dementia (Rana et al., 2012).

Notably, effect sizes for the domain-specific tasks indicated that whilst TR-Y patients outperformed TR-N patients on most measures (largest $g = 0.58$, digit span), this advantage did not extend to all domains. Relative to TR-Y patients, TR-N patients demonstrated superior semantic long-term memory/learning ($g = -0.28$), and no significant subtype differences were observed for executive function measures of cognitive inhibition and working memory (all absolute $g < 0.10$). Moreover, no consistent advantage emerged across measures of language; although TR-Y patients performed better on the Boston Naming Test – Short Form ($g = 0.31$), no difference between subtypes was found on a task of phonemic

fluency (*g* = 0.06). Across all other cognitive domains assessed, however, the TR-Y subtype did outperform the TR-N subtype, showing superior performance on measures of cognitive flexibility (*g* range = 0.20 – 0.27), processing speed (*g* = 0.38), and both short- and long-term memory (*g* range = 0.40 – 0.58). Of note, there was consistent evidence for TR-Y patients having superior visuospatial abilities (reasoning, construction, and perception; *g* range = 0.20 – 0.30) relative to TR-N patients, which extended to measures of short- and long-term memory in the visuospatial modality (*g* range = 0.42 – 0.45).

**Results of With Facial Tremor vs. Without Facial Tremor Synthesis**

One study (Ou et al., 2021) subtyped participants according to the presence (*n* = 403) or absence (*n* = 1821) of facial tremor, as determined by clinical judgement using UPDRS-III item 20A (tremor at rest: face, lips, and chin). In this study, participants with facial tremor were significantly older at time of study participation (*g* = 0.39) and at time of disease onset (*g* = 0.23); had a longer disease duration (*g* = 0.42); were less educated (*g* = 0.15); and were less likely to be men (RR = 0.77) than participants without facial tremor. Participants with facial tremor also had greater overall motor symptom severity (*g* = 0.29), as indicated by significantly higher UPDRS-III total scores (excluding item 20A). Both of the global cognitive measures administered to participants (MoCA, FAB) indicated a small effect size (*g* = 0.23 and *g* = 0.18, respectively) favouring better cognition among patients without facial tremor relative to those with facial tremor.

**Results of Freezing of Gait vs. Non-Freezing of Gait Analyses**

**Table S36**

*Meta-Analyses Comparing Freezing of Gait (FOG) and Non-Freezing of Gait (nFOG) Motor Subtypes on Demographics and Disease Characteristics*

| Characteristic | *k* (*o*) | Pooled effect size (95% CI) | *p* | $I^2$ (%) | *Q* (*p*) | Egger's test (*p*) |
|---|---|---|---|---|---|---|
| **Age*** | | | | | | |
| *All studies* | 19 (1632) | 0.14 (0.03, 0.25) | **.015** | 0.0 | 17.54 (.486) | .633 |
| **Gender (proportion of men)*** | | | | | | |
| *All studies* | 17 (1574) | 1.12 (1.07, 1.19) | **< .001** | 0.0 | 6.51 (.982) | .103 |
| **Years of education*** | | | | | | |
| *All studies* | 9 (837) | -0.14 (-0.31, 0.03) | .087 | 0.0 | 7.83 (.451) | - |
| **Disease duration** | | | | | | |
| *All studies* | 20 (1706) | 0.52 (0.37, 0.68) | **< .001** | 38.8 | 31.06 (**.040**) | .295 |
| *With outliers removed* | 19 (1631) | 0.50 (0.36, 0.64) | **< .001** | 28.0 | 24.99 (.125) | .116 |
| **Age at onset*** | | | | | | |
| *All studies* | 3 (456) | -0.18 (-0.50, 0.13) | .129 | 0.0 | 1.19 (.553) | - |
| **LEDD** | | | | | | |
| *All studies* | 15 (1442) | 0.46 (0.24, 0.68) | **< .001** | 57.8 | 33.18 (**.003**) | .603 |
| *With outliers removed* | 14 (1367) | 0.41 (0.23, 0.58) | **< .001** | 41.0 | 22.03 (.055) | .177 |
| **UPDRS-III total score (original version)*** | | | | | | |
| *All studies* | 10 (979) | 0.59 (0.36, 0.83) | **< .001** | 50.8 | 18.28 (**.032**) | .481 |
| **MDS-UPDRS-III total score*** | | | | | | |
| *All studies* | 10 | 0.73 | **< .001** | 46.8 | 16.91 | .285 |

| | | | | | | |
|---|---|---|---|---|---|---|
| | (727) | (0.46, 1.00) | | | (.050) | |
| **UPDRS-III total score (any version)*** | | | | | | |
| *All studies* | 20 | 0.66 | < **.001** | 50.4 | 38.27 | .499 |
| | (1706) | (0.49, 0.82) | | | (**.006**) | |

*Note.* CI = confidence interval; *k* = number of studies/effect sizes; LEDD = levodopa equivalent daily dose; MDS-UPDRS-III = Movement Disorder Society Unified Parkinson's Disease Rating Scale Part III; *o* = pooled sample size (both groups); UPDRS-III = Unified Parkinson's Disease Rating Scale Part III. Pooled effect size is Hedges' *g* for all variables except gender, which is risk ratio. For Hedges' *g*, positive pooled effect sizes reflect higher values (i.e., older age, longer disease duration) in the freezing of gait (FOG) group relative to the non-freezing of gait (nFOG) group; for risk ratio, values > 1 indicate that freezing of gait (FOG) patients have a greater risk of being men relative to non-freezing of gait (nFOG) patients. $I^2$ is for between-study variance. Egger's test was conducted only when $k \geq 10$ and using Pustejovsky and Rodgers' (2019) revised method. *Results for model with outliers removed not reported as no outliers detected. Significant *p*-values (< .05) are highlighted in bold.

**Figure S16**

*Sunset (Power-Enhanced) Funnel Plots for Difference in Cognitive Performance Between Freezing of Gait (FOG) and Non-Freezing of Gait (nFOG) Motor Subtype Groups*

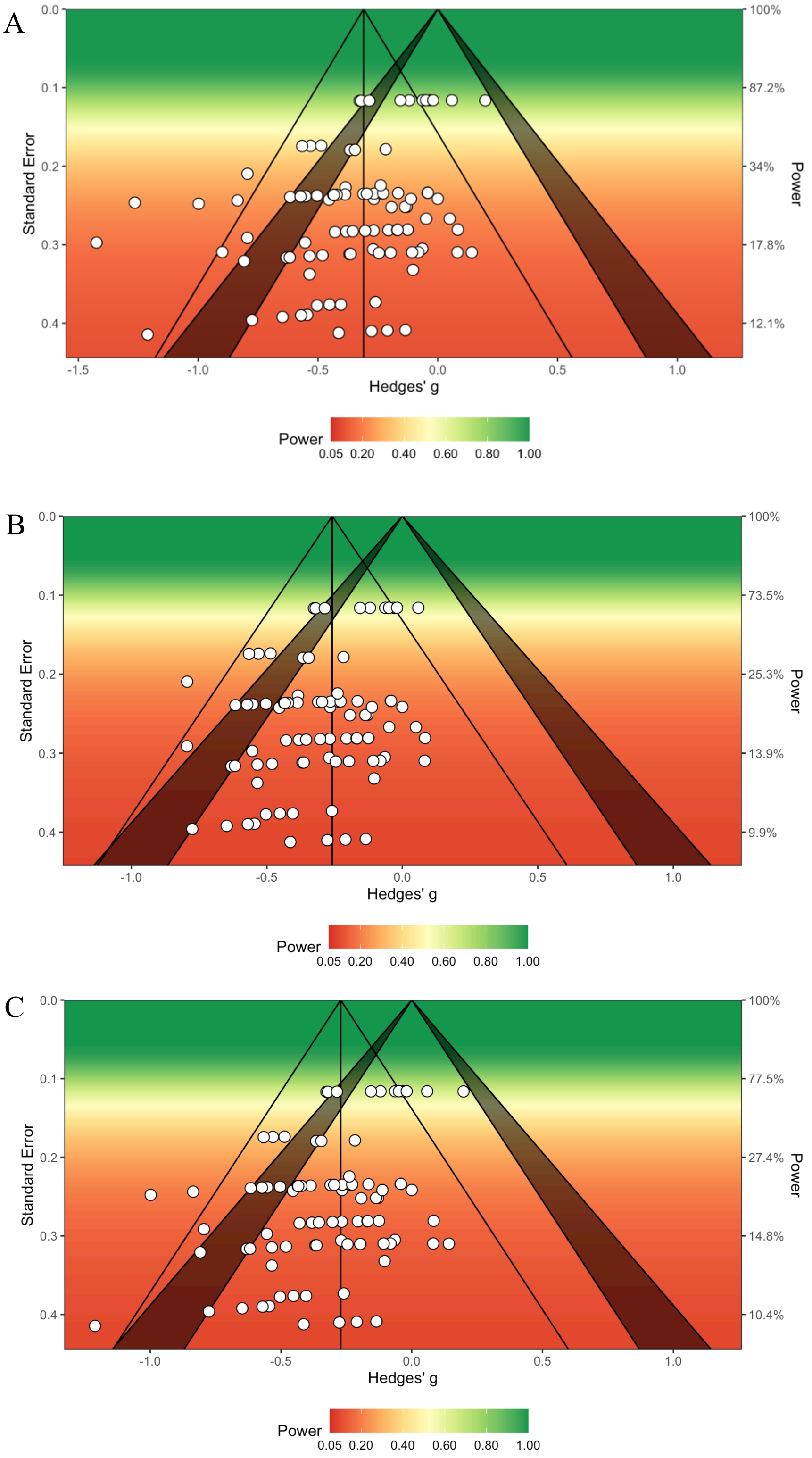


*Note.* A: Full model (no outliers removed). B. Model with outliers removed (residuals approach). C. Model with outliers removed (Cook's distance approach). For Hedges' *g*, values < 0 indicate poorer cognitive performance among freezing of gait (FOG) patients relative to non-freezing of gait (nFOG) patients.

**Table S37**

*Results of Continuous Moderator Analyses for Freezing of Gait (FOG) and Non-Freezing of Gait (nFOG) Motor Subtype Groups (No Outliers Removed)*

| **Moderator** | ***n*** | ***k*** | **$\beta$ (95% CI)** | ***p*** | **Change in Between-Study $I^2$** |
|---|---|---|---|---|---|
| Sample size | 20 | 93 | 0.00 (-0.00, 0.00) | .131 | 8.10 |
| Publication year | 20 | 93 | 0.01 (-0.02, 0.04) | .404 | -1.34 |
| Pooled mean age | 19 | 75 | 0.01 (-0.03, 0.05) | .628 | -1.94 |
| Pooled proportion of men | 17 | 67 | 0.06 (-1.04, 1.16) | .908 | -2.01 |
| Pooled mean years of education | 10 | 51 | 0.08 (-0.04, 0.20) | .175 | 4.89 |
| Pooled mean disease duration | 20 | 93 | 0.01 (-0.04, 0.06) | .669 | 0.08 |
| Pooled mean age at onset | 20 | 93 | 0.00 (-0.03, 0.03) | .806 | -2.58 |
| Pooled mean LEDD | 15 | 79 | 0.00 (-0.00, 0.00) | .470 | -0.47 |
| Pooled mean UPDRS-III (original) | 10 | 48 | -0.01 (-0.03, 0.00) | .827 | 8.87 |
| Pooled mean MDS-UPDRS-III | 10 | 45 | -0.01 (-0.03, 0.01) | .330 | 0.00 |

*Note*. CI = confidence interval; *k* = number of effect sizes; MDS-UPDRS-III = Movement Disorder Society Unified Parkinson's Disease Rating Scale Part III; *n* = number of unique studies; UPDRS-III = Unified Parkinson's Disease Rating Scale Part III; $\beta$ = regression coefficient. For $\beta$, positive values reflect a negative association between the moderator and effect size (i.e., larger values of the moderator are associated with effect sizes approaching zero, reflecting a smaller difference in cognitive performance between the freezing of gait [FOG] group and the non-freezing of gait [nFOG] group). Change in $I^2$ is the difference in between-study $I^2$ for the model with and without the moderator (larger positive values correspond to greater between-study variance being accounted for by the moderator; negative values suggest that model fit worsened as a consequence of including the moderator). Significant *p*-values (< .05) are highlighted in bold.

**Table S38**

*Results of Continuous Moderator Analyses for Freezing of Gait (FOG) and Non-Freezing of Gait (nFOG) Motor Subtype Groups (Outliers Removed – Residuals Approach)*

| **Moderator** | ***n*** | ***k*** | ***β* (95% CI)** | ***p*** | **Change in Between-Study $I^2$** |
|---|---|---|---|---|---|
| Sample size | 20 | 84 | 0.00 (-0.00, 0.00) | .474 | 0.85 |
| Publication year | 20 | 84 | 0.01 (-0.01, 0.04) | .335 | -1.37 |
| Pooled mean age | 19 | 68 | -0.01 (-0.05, 0.03) | .627 | -1.00 |
| Pooled proportion of men | 17 | 61 | 0.45 (-0.50, 1.40) | .351 | 3.35 |
| Pooled mean years of education | 10 | 46 | 0.10 (0.00, 0.19) | **.040** | 17.52 |
| Pooled mean disease duration | 20 | 84 | 0.01 (-0.03, 0.05) | .637 | 0.69 |
| Pooled mean age at onset | 20 | 84 | -0.01 (-0.03, 0.02) | .594 | 0.37 |
| Pooled mean LEDD | 15 | 71 | 0.00 (-0.00, 0.00) | .608 | -0.88 |
| Pooled mean UPDRS-III (original) | 10 | 41 | -0.01 (-0.02, 0.01) | .415 | -0.64 |
| Pooled mean MDS-UPDRS-III* | 10 | 43 | -0.01 (-0.03, 0.01) | .404 | 0.00 |

*Note*. CI = confidence interval; *k* = number of effect sizes; MDS-UPDRS-III = Movement Disorder Society Unified Parkinson's Disease Rating Scale Part III; *n* = number of unique studies; UPDRS-III = Unified Parkinson's Disease Rating Scale Part III; *β* = regression coefficient. For *β*, positive values reflect a negative association between the moderator and effect size (i.e., larger values of the moderator are associated with effect sizes approaching zero, reflecting a smaller difference in cognitive performance between the freezing of gait [FOG] group and the non-freezing of gait [nFOG] group). Change in $I^2$ is the difference in between-study $I^2$ for the model with and without the moderator (larger positive values correspond to greater between-study variance being accounted for by the moderator; negative values suggest that model fit worsened as a consequence of including the moderator). Significant *p*-values (< .05) are highlighted in bold.

**Figure S17**

*Relationship Between Pooled Mean Years of Education and Effect Size for Cognitive Difference Between Freezing of Gait (FOG) and Non-Freezing of Gait (nFOG) Motor Subtype Groups*

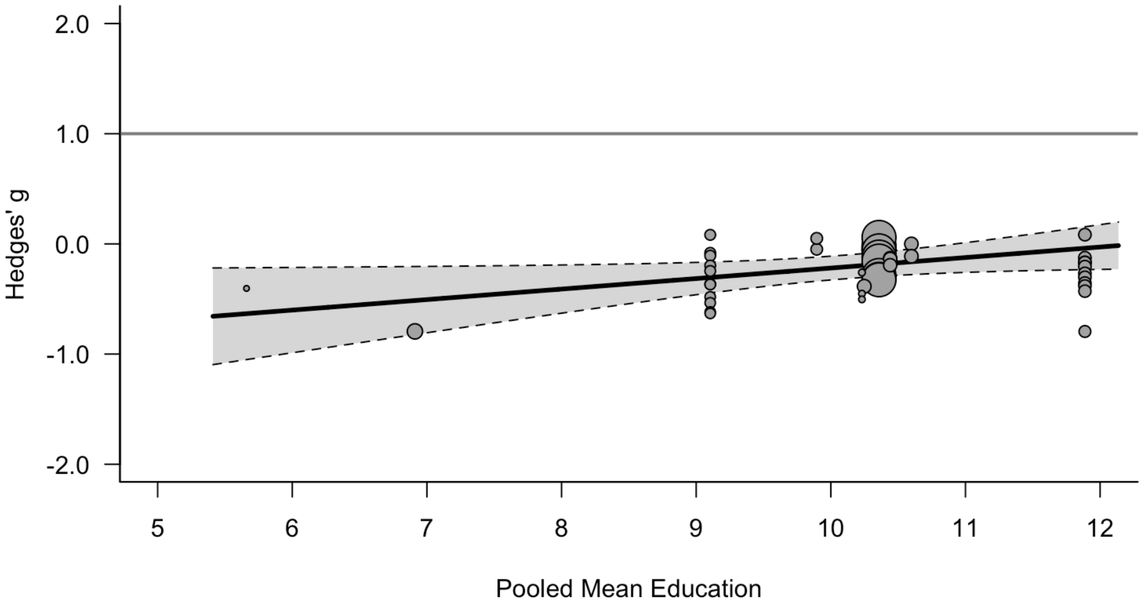


*Note*. For Hedges' *g*, values < 0 indicate better cognitive performance among non-freezing of gait (nFOG) patients relative to freezing of gait (FOG) patients.

**Table S39**

*Results of Categorical Moderator Analyses for Freezing of Gait (FOG) and Non-Freezing of Gait (nFOG) Motor Subtype Groups (No Outliers Removed)*

| **Moderator** | ***n*** | ***k*** | **Pooled Hedges' *g* (95% CI)** | ***β* (95% CI)** | ***p*** | ***Q* (*p*)** | **ToM (*p*)** |
|---|---|---|---|---|---|---|---|
| **Cognitive Class** | | | | | | 138.37 (**.001**) | 7.56 (**.007**) |
| *Global** | 19 | 34 | -0.42 (-0.54, -0.30) | | **< .001** | | |
| *Specific* | 9 | 59 | -0.24 (-0.35, -0.12) | 0.18 (0.05, 0.31) | **.007** | | |
| **Cognitive Domain** | | | | | | 113.42 (**.001**) | 1.16 (.325) |
| *Global Cognitive Function** | 19 | 34 | -0.43 (-0.55, -0.30) | | **< .001** | | |
| *Executive Function – Cognitive Flexibility* | 4 | 6 | -0.28 (-0.46, -0.10) | 0.14 (-0.05, 0.34) | .144 | | |
| *Executive Function – Cognitive Inhibition* | 3 | 3 | -0.46 (-0.73, -0.19) | -0.03 (-0.29, 0.23) | .802 | | |
| *Executive Function – Working Memory* | 5 | 8 | -0.26 (-0.45, -0.06) | 0.17 (-0.03, 0.37) | .087 | | |
| *Higher-Order Fluid Abilities – Planning* | 2 | 2 | -0.37 (-0.77, 0.03) | 0.06 (-0.35, 0.46) | .784 | | |
| *Language* | 3 | 4 | -0.31 (-0.57, -0.05) | 0.12 (-0.13, 0.37) | .346 | | |
| *LTM/Learning – Lexical* | 3 | 3 | -0.12 (-0.43, 0.20) | 0.31 (-0.02, 0.63) | .061 | | |
| *LTM/Learning – Semantic* | 3 | 3 | -0.26 (-0.51, -0.00) | 0.17 (-0.08, 0.42) | .177 | | |

| | | | | | | | |
|---|---|---|---|---|---|---|---|
| *LTM/Learning – Visuospatial* | 3 | 3 | -0.14 (-0.45, 0.16) | 0.28 (-0.01, 0.58) | .058 | | |
| *STM – Lexical* | 3 | 3 | -0.01 (-0.32, 0.30) | 0.41 (0.10, 0.74) | **.013** | | |
| *STM – Numerical* | 2 | 2 | -0.27 (-0.64, 0.11) | 0.16 (-0.22, 0.54) | .402 | | |
| *STM – Visuospatial* | 3 | 4 | -0.19 (-0.50, 0.13) | 0.24 (-0.09, 0.56) | .149 | | |
| *Processing Speed* | 4 | 7 | -0.34 (-0.52, -0.16) | 0.09 (-0.11, 0.28) | .384 | | |
| *Visuospatial Abilities – Perception* | 2 | 2 | -0.12 (-0.49, 0.25) | 0.30 (-0.07, 0.68) | .111 | | |
| *Visuospatial Abilities – Reasoning* | 2 | 3 | -0.17 (-1.08, 0.74) | 0.26 (-0.64, 1.16) | .566 | | |
| **Medication Status for Cognitive Assessment(s)** | | | | | | 105.96 (**.002**) | 0.43 (.515) |
| *ON or OFF** | 2 | 5 | -0.47 (-0.71, -0.24) | | < **.001** | | |
| *ON* | 13 | 65 | -0.38 (-0.54, -0.23) | 0.09 (-0.19, 0.38) | .515 | | |
| **Medication Status for Motor Assessment(s)** | | | | | | 106.76 (**.008**) | 0.61 (.546) |
| *ON or OFF** | 2 | 5 | -0.48 (-0.71, -0.24) | | | | |
| *ON* | 10 | 42 | -0.32 (-0.48, -0.16) | 0.16 (-0.13, 0.44) | < **.001** | | |
| *OFF* | 6 | 30 | -0.40 (-0.62, -0.17) | 0.08 (-0.25, 0.41) | .626 | | |

| | | | | | | | |
|---|---|---|---|---|---|---|---|
| **Cognitive Impairment/Dementia as Exclusion Criterion** | | | | | | 149.48 (**< .001**) | 0.12 (.731) |
| *No*$^{*}$ | 7 | 24 | -0.39 (-0.61, -0.17) | | **.001** | | |
| *Yes* | 13 | 69 | -0.35 (-0.46, -0.23) | 0.04 (-0.20, 0.29) | .731 | | |
| **Overall Risk of Bias** | | | | | | 140.93 (**< .001**) | 1.54 (.220) |
| *High*$^{*}$ | 11 | 27 | -0.45 (-0.61, -0.30) | | **< .001** | | |
| *Moderate* | 6 | 33 | -0.35 (-0.53, -0.16) | 0.10 (-0.14, 0.34) | .394 | | |
| *Low* | 3 | 33 | -0.23 (-0.43, -0.04) | 0.22 (0.03, 0.47) | .084 | | |

*Note*. CI = confidence interval; *k* = number of effect sizes; LTM = long-term memory; *n* = number of unique studies; STM = short-term memory; ToM = Test of Moderators; $\beta$ = regression coefficient. $^{*}$ denotes reference category for model. For Hedges' *g*, values < 0 reflect indicate poorer cognitive performance among freezing of gait (FOG) patients relative to non-freezing of gait (FOG) patients. The $\beta$ coefficients and corresponding *p*-values indicate whether the pooled effect size for that level of the moderator differs significantly from the pooled effect size of the reference category; for the reference category, these values indicate whether the pooled effect size differs significantly from zero. For Hedges' *g*, values < 0 indicate poorer cognitive performance among freezing of gait (FOG) patients relative to non-freezing of gait (FOG) patients. No moderator analyses performed for subtyping method as all effect sizes belonged to the same moderator level ('Other'). No moderator analyses performed for motor subtypes compared on cognition in paper as only *k* = 1 for 'No' level of moderator. Significant *p*-values (< .05) are highlighted in bold.

**Table S40**

*Results of Categorical Moderator Analyses for Freezing of Gait (FOG) and Non-Freezing of Gait (nFOG) Motor Subtype Groups (Outliers Removed – Residuals Approach)*

| Moderator | *n* | *k* | Pooled Hedges' *g* (95% CI) | *β* (95% CI) | *p* | *Q* (*p*) | ToM (*p*) |
|---|---|---|---|---|---|---|---|
| **Cognitive Class** | | | | | | 68.73 (.852) | 3.46 (.066) |
| *Global** | 19 | 30 | -0.34 (-0.44, -0.23) | | **< .001** | | |
| *Specific* | 9 | 54 | -0.24 (-0.34, -0.13) | 0.10 (-0.01, 0.21) | .066 | | |
| **Cognitive Domain** | | | | | | 53.96 (.784) | 1.01 (.453) |
| *Global Cognitive Function** | 19 | 30 | -0.34 (-0.45, -0.24) | | **< .001** | | |
| *Executive Function – Cognitive Flexibility* | 4 | 6 | -0.29 (-0.44, -0.14) | 0.05 (-0.10, 0.20) | .494 | | |
| *Executive Function – Cognitive Inhibition* | 3 | 3 | -0.47 (-0.77, -0.17) | -0.13 (-0.40, 0.15) | .358 | | |
| *Executive Function – Working Memory* | 5 | 8 | -0.27 (-0.47, -0.08) | 0.07 (-0.11, 0.25) | .437 | | |
| *Higher-Order Fluid Abilities – Planning* | 2 | 2 | -0.39 (-0.76, -0.03) | -0.05 (-0.42, 0.32) | .794 | | |
| *Language* | 3 | 3 | -0.28 (-0.52, -0.03) | 0.07 (-0.16, 0.29) | .548 | | |
| *LTM/Learning – Lexical* | 2 | 2 | -0.06 (-0.49, 0.37) | 0.29 (-0.13, 0.70) | .170 | | |
| *LTM/Learning – Semantic* | 3 | 3 | -0.26 (-0.47, -0.04) | 0.08 (-0.12, 0.29) | .410 | | |

| | | | | | | | |
|---|---|---|---|---|---|---|---|
| *LTM/Learning – Visuospatial* | 2 | 2 | -0.23 (-0.68, 0.22) | 0.11 (-0.34, 0.57) | .621 | | |
| *STM – Lexical* | 3 | 3 | 0.06 (-0.28, 0.40) | 0.40 (0.07, 0.73) | **.018** | | |
| *STM – Numerical* | 2 | 2 | -0.19 (-0.65, 0.26) | 0.15 (-0.29, 0.59) | .497 | | |
| *STM – Visuospatial* | 3 | 4 | -0.17 (-0.48, 0.15) | 0.18 (-0.13, 0.48) | .250 | | |
| *Processing Speed* | 4 | 6 | -0.28 (-0.43, -0.12) | 0.07 (-0.10, 0.24) | .431 | | |
| *Visuospatial Abilities – Perception* | 2 | 2 | -0.15 (-0.49, 0.20) | 0.20 (-0.15, 0.54) | .254 | | |
| *Visuospatial Abilities – Reasoning* | 2 | 2 | -0.18 (-0.61, 0.25) | 0.17 (-0.27, 0.60) | .448 | | |
| **Medication Status for Cognitive Assessment(s)** | | | | | | 56.06 (.688) | 1.78 (.187) |
| *ON or OFF* * | 2 | 5 | -0.48 (-0.70, -0.26) | | **< .001** | | |
| *ON* | 13 | 59 | -0.31 (-0.44, -0.18) | 0.17 (-0.08, 0.42) | .187 | | |
| **Medication Status for Motor Assessment(s)** | | | | | | 56.77 (.832) | 2.41 (.097) |
| *ON or OFF* * | 2 | 5 | -0.48 (-0.69, -0.26) | | **< .001** | | |
| *ON* | 10 | 39 | -0.23 (-0.34, -0.12) | 0.25 (0.01, 0.49) | **.043** | | |
| *OFF* | 6 | 27 | -0.37 (-0.60, -0.15) | 0.11 (-0.21, 0.42) | .502 | | |
| **Cognitive Impairment/Dementia as Exclusion Criterion** | | | | | | 72.93 (.753) | 0.84 (.363) |
| *No* * | 7 | 22 | -0.38 (-0.58, -0.18) | | **< .001** | | |
| *Yes* | 13 | 62 | -0.27 (-0.38, -0.16) | -0.11 (-0.12, 0.34) | .363 | | |

| | | | | | | | |
|---|---|---|---|---|---|---|---|
| **Overall Risk of Bias** | | | | | | 68.73 (.833) | 0.68 (.508) |
| *High*[*] | 11 | 23 | -0.31 (-0.47, -0.16) | | **< .001** | | |
| *Moderate* | 6 | 31 | -0.35 (-0.55, -0.15) | -0.04 (-0.29, 0.21) | .753 | | |
| *Low* | 3 | 30 | -0.22 (-0.37, -0.06) | 0.10 (-0.12, 0.31) | .379 | | |

*Note*. CI = confidence interval; *k* = number of effect sizes; LTM = long-term memory; *n* = number of unique studies; STM = short-term memory; ToM = Test of Moderators; $\beta$ = regression coefficient. [*] denotes reference category for model. For Hedges' *g*, values < 0 reflect indicate poorer cognitive performance among freezing of gait (FOG) patients relative to non-freezing of gait (FOG) patients. The $\beta$ coefficients and corresponding *p*-values indicate whether the pooled effect size for that level of the moderator differs significantly from the pooled effect size of the reference category; for the reference category, these values indicate whether the pooled effect size differs significantly from zero. For Hedges' *g*, values < 0 indicate poorer cognitive performance among freezing of gait (FOG) patients relative to non-freezing of gait (FOG) patients. No moderator analyses performed for subtyping method as all effect sizes belonged to the same moderator level ('Other'). No moderator analyses performed for motor subtypes compared on cognition in paper as only *k* = 1 for 'No' level of moderator. Significant *p*-values (< .05) are highlighted in bold.

**Table S41**

*Results of Confound Analyses for Freezing of Gait (FOG) and Non-Freezing of Gait (nFOG) Motor Subtype Groups (No Outliers Removed)*

| **Confound** | ***n*** | ***k*** | ***β* (95% CI)** | ***p*** | **Change in Between-Study $I^2$** |
|---|---|---|---|---|---|
| SMD age | 19 | 75 | -0.02 (-0.48, 0.44) | .945 | -2.01 |
| Difference in proportion of men | 17 | 67 | 0.58 (-1.13, 2.28) | .503 | 1.90 |
| SMD years of education | 10 | 51 | 0.31 (-0.48, 1.11) | .433 | -0.21 |
| SMD disease duration | 20 | 93 | -0.03 (-0.38, 0.32) | .851 | -1.96 |
| SMD LEDD | 15 | 79 | -0.01 (-0.36, 0.33) | .945 | -2.12 |
| SMD UPDRS-III (original) | 10 | 48 | -0.09 (-0.64, 0.46) | .734 | -2.12 |
| SMD MDS-UPDRS-III | 10 | 45 | -0.15 (-0.54, 0.23) | .431 | 0.00 |
| SMD UPDRS-III (any version) | 20 | 93 | -0.13 (-0.43, 0.17) | .400 | 3.08 |

*Note*. CI = confidence interval; *k* = number of effect sizes; LEDD = levodopa equivalent daily dose; MDS-UPDRS-III = Movement Disorder Society Unified Parkinson's Disease Rating Scale Part III; *n* = number of unique studies; UPDRS-III = Unified Parkinson's Disease Rating Scale Part III; SMD = standardised mean difference (Hedges' *g*); *β* = regression coefficient. For *β*, positive values reflect a negative association between the confound (moderator) and effect size (i.e., larger values of the confound [moderator] are associated with effect sizes approaching zero, reflecting a smaller difference in cognitive performance between the freezing of gait [FOG] group and the non-freezing of gait [nFOG] group). Change in $I^2$ is the difference in between-study $I^2$ for the model with and without the confound (moderator; larger positive values correspond to greater between-study variance being accounted for by the confound [moderator]; negative values suggest that model fit worsened as a consequence of including the confound [moderator]). No confound (moderator) analyses performed for SMD in age at disease onset as $n < 10$ studies. Significant *p*-values (< .05) are highlighted in bold.

**Table S42**

*Results of Confound Analyses for Freezing of Gait (FOG) and Non-Freezing of Gait (nFOG) Motor Subtype Groups (Outliers Removed – Residuals Approach)*

| **Confound** | ***n*** | ***k*** | ***β* (95% CI)** | ***p*** | **Change in Between-Study $I^2$** |
|---|---|---|---|---|---|
| SMD age | 19 | 68 | 0.00 (-0.42, 0.42) | .995 | -2.03 |
| Difference in proportion of men | 17 | 61 | 0.09 (-1.49, 1.67) | .912 | -2.46 |
| SMD years of education | 10 | 46 | 0.37 (-0.23, 0.97) | .220 | 8.67 |
| SMD disease duration | 20 | 84 | -0.10 (-0.42, 0.22) | .539 | -2.27 |
| SMD LEDD | 15 | 71 | -0.07 (-0.39, 0.25) | .676 | -2.58 |
| SMD UPDRS-III (original) | 10 | 41 | -0.39 (-0.72, -0.06) | **.021** | 29.44 |
| SMD MDS-UPDRS-III | 10 | 43 | -0.15 (-0.52, 0.23) | .437 | 0.00 |
| SMD UPDRS-III (any version) | 20 | 84 | -0.28 (-0.51, -0.05) | **.018** | 16.65 |

*Note*. CI = confidence interval; *k* = number of effect sizes; LEDD = levodopa equivalent daily dose; MDS-UPDRS-III = Movement Disorder Society Unified Parkinson's Disease Rating Scale Part III; *n* = number of unique studies; UPDRS-III = Unified Parkinson's Disease Rating Scale Part III; SMD = standardised mean difference (Hedges' *g*); *β* = regression coefficient. For *β*, positive values reflect a negative association between the confound (moderator) and effect size (i.e., larger values of the confound [moderator] are associated with effect sizes approaching zero, reflecting a smaller difference in cognitive performance between the freezing of gait [FOG] group and the non-freezing of gait [nFOG] group). Change in $I^2$ is the difference in between-study $I^2$ for the model with and without the confound (moderator; larger positive values correspond to greater between-study variance being accounted for by the confound [moderator]; negative values suggest that model fit worsened as a consequence of including the confound [moderator]). No confound (moderator) analyses performed for SMD in age at disease onset as $n < 10$ studies. Significant *p*-values (< .05) are highlighted in bold.

**Figure S18**

*Relationship Between Standardised Mean Difference (SMD) in Unified Parkinson's Disease Rating Scale Part III and Effect Size for Cognitive Difference Between Freeing of Gait (FOG) and Non-Freezing of Gait (nFOG) Motor Subtype Groups*

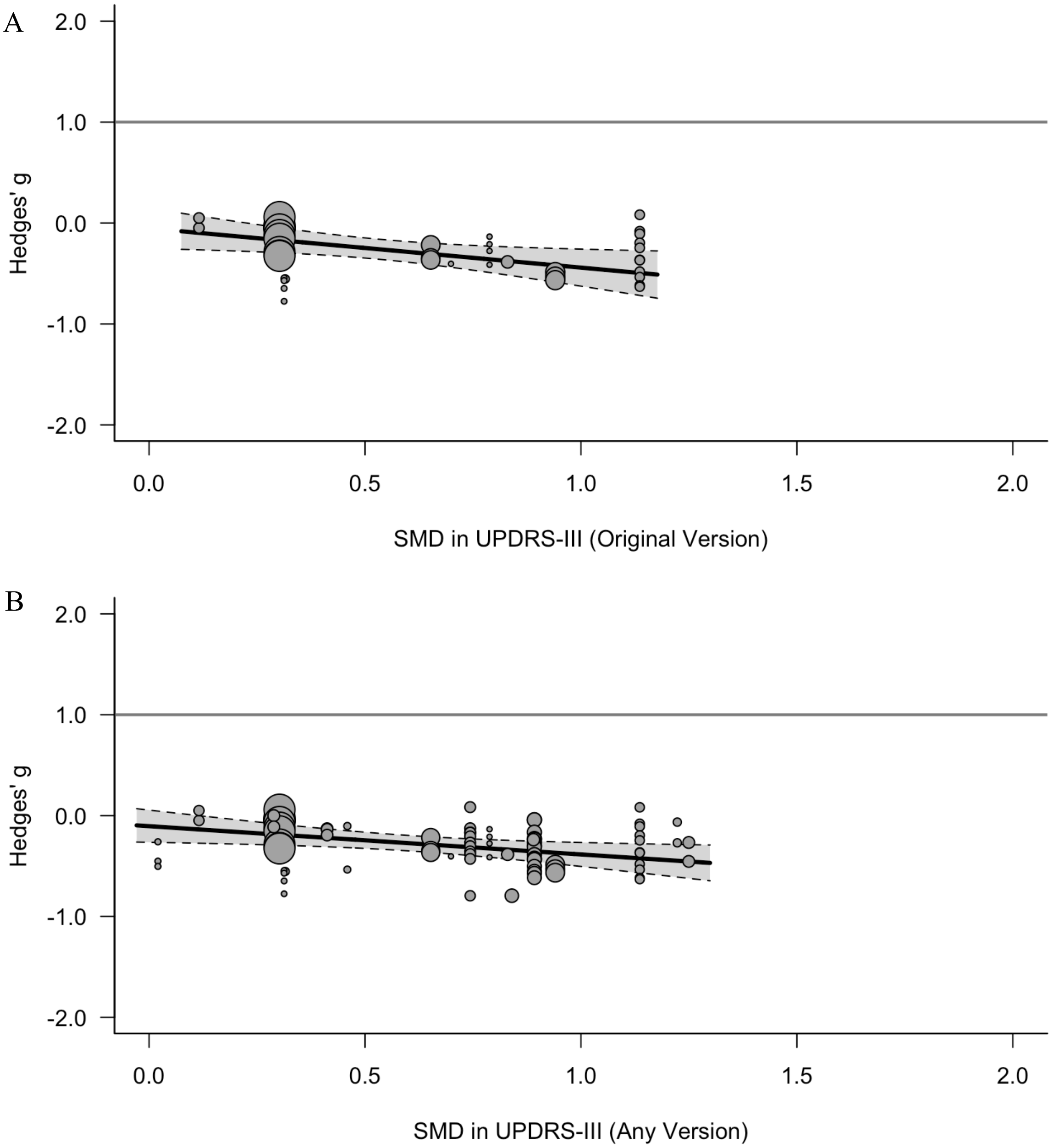


*Note.* A. Unified Parkinson's Disease Rating Scale Part III – original version. B. Unified Parkinson's Disease Rating Scale Part III – any version. SMD = standardised mean difference; UPDRS-III = Unified Parkinson's Disease Rating Scale Part III. For Hedges' *g*, values < 0 indicate better cognitive performance among non-freezing of gait (nFOG) patients relative to freezing of gait (FOG) patients.

**Results of Poor Gait vs. Good Gait Analyses**

**Table S43**

*Meta-Analyses Comparing Poor Gait and Good Gait Motor Subtypes on Demographics and Disease Characteristics*

| Characteristic | *k* (*o*) | Pooled effect size (95% CI) | *p* | $I^2$ (%) | *Q* (*p*) |
|---|---|---|---|---|---|
| **Age*** | | | | | |
| *All studies* | 4 (263) | 0.14 (-1.20, 1.48) | .759 | 89.4 | 28.33 (< **.001**) |
| **Gender (proportion of men)*** | | | | | |
| *All studies* | 4 (263) | 0.95 (0.87, 1.04) | .165 | 0.00 | 0.23 (.972) |
| **Disease duration*** | | | | | |
| *All studies* | 4 (263) | 0.58 (0.26, 0.91) | **.011** | 0.00 | 1.79 (.618) |
| **LEDD*** | | | | | |
| *All studies* | 4 (263) | 0.12 (-1.39, 1.63) | .814 | 90.0 | 30.03 (< **.001**) |

*Note.* CI = confidence interval; *k* = number of studies/effect sizes; LEDD = levodopa equivalent daily dose; *o* = pooled sample size (both groups); UPDRS-III = Unified Parkinson's Disease Rating Scale Part III. Pooled effect size is Hedges' *g* for all variables except gender, which is risk ratio. For Hedges' *g*, positive pooled effect sizes reflect higher values (i.e., older age, longer disease duration) in the poor gait group relative to the good gait group; for risk ratio, values > 1 indicate that poor gait patients have a greater risk of being men relative to good gait patients. $I^2$ is for between-study variance. Egger's test was conducted only when $k \geq 10$ and using Pustejovsky and Rodgers' (2019) revised method. * Results for model with outliers removed are not reported as no outliers were detected. Significant *p*-values (< .05) are highlighted in bold.

**Results of Fallers vs. Non-Fallers Synthesis**

One study (Altmann et al., 2023) subtyped patients ($o$ = 1810) according to whether they had at least one fall during a two-week inpatient stay at a PD clinic. A fall event or its outcome had to be observed and recorded by a clinician. Consistent with our previously reported subtype comparisons that included a gait-related subtype, fallers had a longer disease duration than non-fallers ($g$ = 0.36) and greater overall motor impairment ($g$ = 0.60). Altmann and colleagues (2023) compared fallers and non-fallers on two global measures of cognition and found non-fallers to have significantly better cognitive function relative to fallers ($g$ = 0.74 and $g$ = 0.69 for MoCA and FAB, respectively).

## Risk of Bias

### Figure S19

*Risk of Bias Summary Plots for Jankovic et al. (1990) and Stebbins et al. (2013) Motor Subtyping Methods*

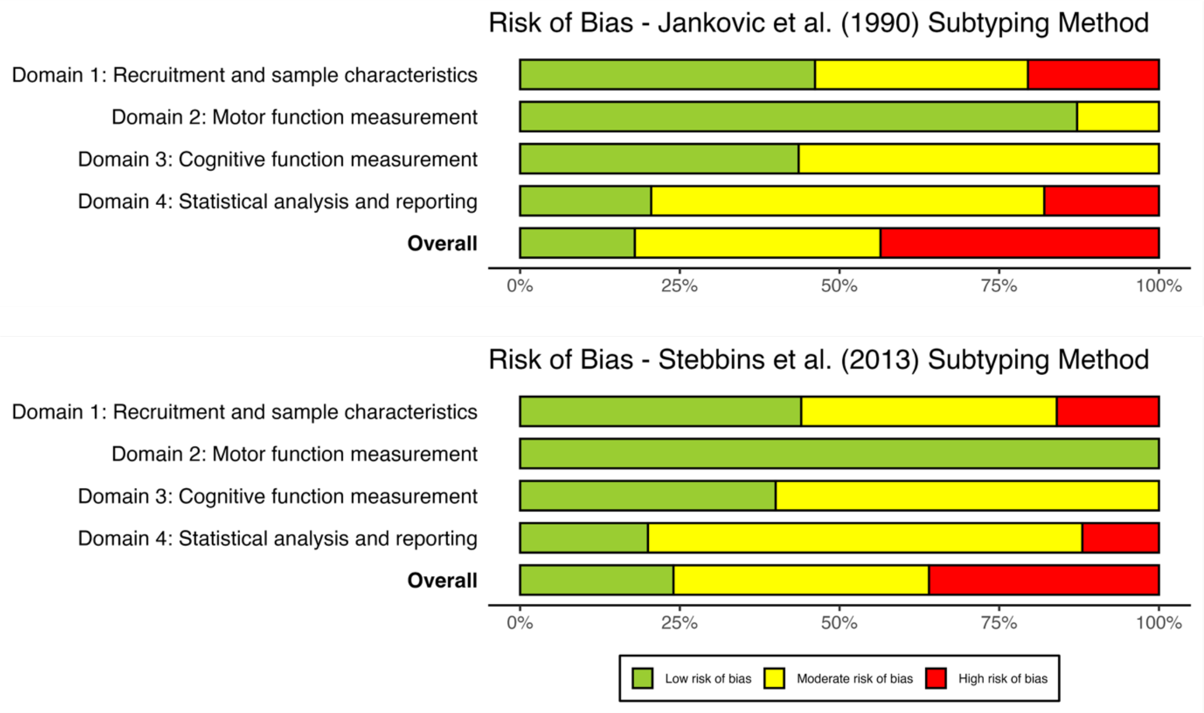


### Figure S20

*Risk of Bias Summary Plots for Studies Reporting Continuous and Categorical Cognitive Outcome Data*

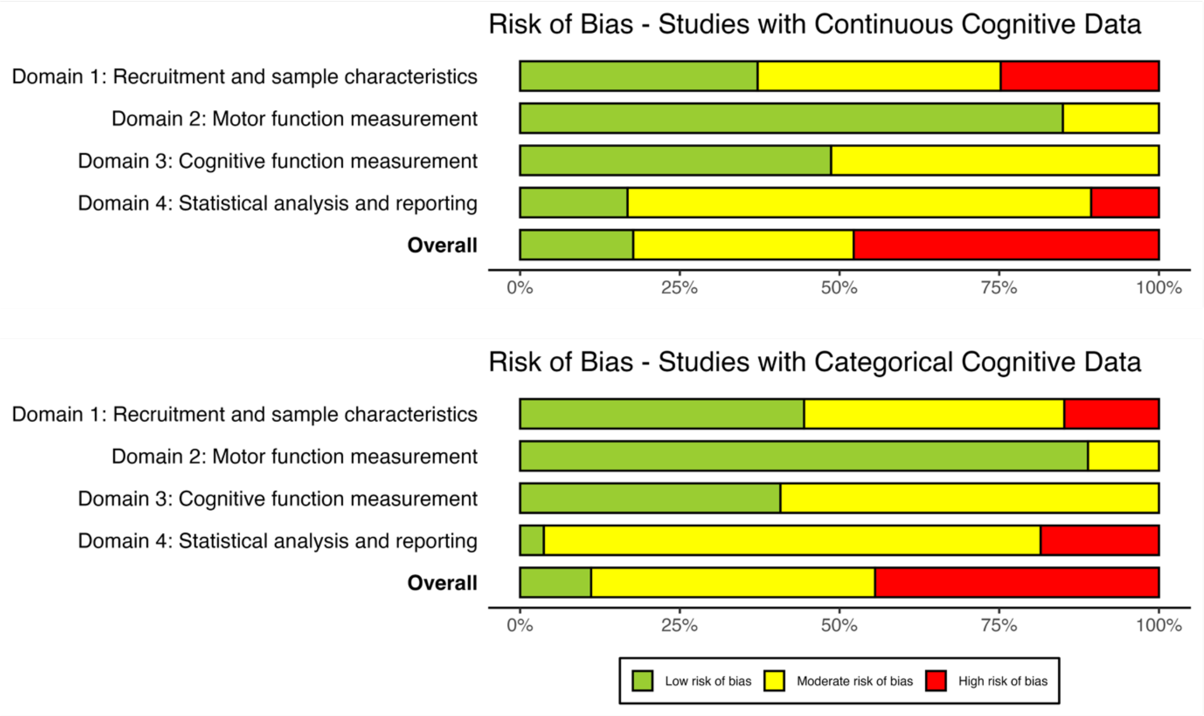

**Grading of Recommendations, Assessment, Development, and Evaluations (GRADE)**

Table S44 provides a summary of the GRADE framework used to evaluate pooled effects for motor subtype pairs with continuous cognitive outcome data available for at least 10 studies. Quality of evidence for all motor subtype pairs was deemed low or very low. All motor subtype pairs were downgraded due to study limitations, which resulted from most included studies being assessed as having a moderate or high risk of bias. TD/PIGD and all motor subtype pairs (TD/AR, TD/NTD, FOG/nFOG) not belonging to the TD/PIGD/ID framework were also downgraded for evidence of publication bias. Quality of evidence was strongest for the subtype pairs produced from the TD/PIGD/ID subtyping framework. This is likely, at least in part, due to the large number of available studies and consistency in subtyping procedures used to classify patients into these groups, which contributed to more precise estimates and allowed for a more comprehensive coverage of cognitive domains (directness).

**Table S44**

*Grading of Recommendations, Assessment, Development, and Evaluation (GRADE) Summary of Findings*

| | GRADE Factor | | | | | | | |
|---|---|---|---|---|---|---|---|---|
| **Subtype Pair** | Study limitations | Inconsistency | Indirectness | Imprecision | Publication bias | Moderate/ large effect size | Dose-effect | Overall certainty of evidence |
| TD / PIGD | × | × | ✓ | ✓ | × | × | × | + |
| TD / ID | × | ✓ | ✓ | ✓ | ✓ | × | × | ++ |
| PIGD / ID | × | ✓ | ✓ | ✓ | ✓ | × | × | ++ |
| TD / AR | × | × | ✓ | ✓ | × | × | × | + |
| TD / NTD | × | ✓ | × | ✓ | ✓ | × | unclear | + |
| FOG / nFOG | × | ✓ | ✓ | ✓ | × | × | unclear | + |

*Note.* Dose-effect = exposure-gradient response. For GRADE factors: ✓ = no serious limitations; × = serious limitations (or not present for moderate/large effect sizes, dose-effect); unclear = unable to rate item based on available information. For overall quality of evidence: + = very low; ++ = low; +++ = moderate; ++++ = high.

**Reference List: All Studies Included in Review**

Allan, L. M., Ballard, C. G., Burn, D. J., & Kenny, R. A. (2005). Prevalence and severity of gait disorders in Alzheimer's and non-Alzheimer's dementias. *Journal of the American Geriatrics Society*, *53*(10), 1681–1687. https://doi.org/10.1111/j.1532-5415.2005.53552.x

Altmann, C. F., Koschel, J., & Jost, W. H. (2023). Predictors of falls in Parkinson's disease, progressive supranuclear palsy, and multiple system atrophy: A retrospective study. *Neurologia i Neurochirurgia Polska*, *57*(3), 297–304. https://doi.org/10.5603/PJNNS.a2023.0036

Alves, G., Larsen, J. P., Emre, M., Wentzel-Larsen, T., & Aarsland, D. (2006). Changes in motor subtype and risk for incident dementia in Parkinson's disease. *Movement Disorders*, *21*(8), 1123–1130. https://doi.org/10.1002/mds.20897

Alves, G., Pedersen, K. F., Bloem, B. R., Blennow, K., Zetterberg, H., Borm, G. F., Dalaker, T. O., Beyer, M. K., Aarsland, D., Andreasson, U., Lange, J., Tysnes, O.-B., Zivadinov, R., & Larsen, J. P. (2013). Cerebrospinal fluid amyloid- and phenotypic heterogeneity in de novo Parkinson's disease. *Journal of Neurology, Neurosurgery & Psychiatry*, *84*(5), 537–543. https://doi.org/10.1136/jnnp-2012-303808

Arie, L., Herman, T., Shema-Shiratzky, S., Giladi, N., & Hausdorff, J. M. (2017). Do cognition and other non-motor symptoms decline similarly among patients with Parkinson's disease motor subtypes? Findings from a 5-year prospective study. *Journal of Neurology*, *264*(10), 2149–2157. https://doi.org/10.1007/s00415-017-8605-x

Assogna, F., Pellicano, C., Cravello, L., Savini, C., Pierantozzi, M., Mercuri, B., Caltagirone, C., Pontieri, F. E., Spalletta, G., & Stefani, A. (2018). Psychiatric profile of motor subtypes of de novo drug-naïve Parkinson's disease patients. *Brain and Behavior*, *8*(10), e01094. https://doi.org/10.1002/brb3.1094

Baig, F., Lawton, M., Rolinski, M., Ruffmann, C., Nithi, K., Evetts, S. G., Fernandes, H. R., Ben-Shlomo, Y., & Hu, M. T. M. (2015). Delineating nonmotor symptoms in early Parkinson's disease and first-degree relatives. *Movement Disorders*, *30*(13), 1759–1766. https://doi.org/10.1002/mds.26281

Basaia, S., Agosta, F., Francia, A., Cividini, C., Balestrino, R., Stojkovic, T., Stankovic, I., Markovic, V., Sarasso, E., Gardoni, A., De Micco, R., Albano, L., Stefanova, E., Kostic, V. S., & Filippi, M. (2022). Cerebro-cerebellar motor networks in clinical

subtypes of Parkinson’s disease. *Npj Parkinson's Disease*, *8*(1), 113. https://doi.org/10.1038/s41531-022-00377-w

Bohnen, N. I., Frey, K. A., Studenski, S., Kotagal, V., Koeppe, R. A., Constantine, G. M., Scott, P. J. H., Albin, R. L., & Müller, M. L. T. M. (2014). Extra-nigral pathological conditions are common in Parkinson’s disease with freezing of gait: An in vivo positron emission tomography study. *Movement Disorders*, *29*(9), 1118–1124. https://doi.org/10.1002/mds.25929

Burn, D. J., Rowan, E. N., Allan, L. M., Molloy, S., O’Brien, J. T., & McKeith, I. G. (2006). Motor subtype and cognitive decline in Parkinson’s disease, Parkinson’s disease with dementia, and dementia with Lewy bodies. *Journal of Neurology, Neurosurgery & Psychiatry*, *77*(5), 585–589. https://doi.org/10.1136/jnnp.2005.081711

Burn, D. J., Rowan, E. N., Minett, T., Sanders, J., Myint, P., Richardson, J., Thomas, A., Newby, J., Reid, J., O’Brien, J. T., & McKeith, I. G. (2003). Extrapyramidal features in Parkinson’s disease with and without dementia and dementia with Lewy bodies: A cross-sectional comparative study. *Movement Disorders*, *18*(8), 884–889. https://doi.org/10.1002/mds.10455

Cancela, J. M., Nascimento, C. M., Varela, S., Seijo-Martínez, M., Lorenzo-López, L., Millán-Calenti, J. C., Domínguez-Vivero, C., & Ayán, C. (2018). Influence of cognitive impairment on the freezing of gait in non demented people with Parkinson’s disease. *Revista de Neurología*, *66*, 289–296. https://doi.org/10.33588/rn.6609.2017289

Che, N.-N., Jiang, Q.-H., Chen, S., Chen, S.-Y., Zhao, Z.-X., Li, X., Ma, J.-J., Zhang, J.-W., Malik, R. A., & Yang, H.-Q. (2023). The severity of corneal nerve loss differentiates motor subtypes in patients with Parkinson’s disease. *Therapeutic Advances in Neurological Disorders*, *16*, 1–10. https://doi.org/10.1177/17562864231165561

Chen, J., Jiang, X., Wu, J., Wu, H., Zhou, C., Guo, T., Bai, X., Liu, X., Wen, J., Cao, Z., Gu, L., Yang, W., Pu, J., Guan, X., Xu, X., Zhang, B., & Zhang, M. (2022). Gray and white matter alterations in different predominant side and type of motor symptom in Parkinson’s disease. *CNS Neuroscience & Therapeutics*, *28*(9), 1372–1379. https://doi.org/10.1111/cns.13877

Choi, S.-M., Kim, B. C., Cho, B.-H., Kang, K. W., Choi, K.-H., Kim, J.-T., Lee, S.-H., Park, M.-S., Kim, M.-K., & Cho, K.-H. (2018). Comparison of two motor subtype classifications in de novo Parkinson’s disease. *Parkinsonism & Related Disorders*, *54*, 74–78. https://doi.org/10.1016/j.parkreldis.2018.04.021

Danti, S., Toschi, N., Diciotti, S., Tessa, C., Poletti, M., Del Dotto, P., & Lucetti, C. (2015). Cortical thickness in de novo patients with Parkinson disease and mild cognitive impairment with consideration of clinical phenotype and motor laterality. *European Journal of Neurology*, *22*(12), 1564–1572. https://doi.org/10.1111/ene.12785

Das, D., Biswas, A., Roy, A., Sauerbier, A., & Bhattacharyya, K. (2016). Cognitive impairment in idiopathic Parkinson's disease. *Neurology India*, *64*(3), 419. https://doi.org/10.4103/0028-3886.181533

de Almeida, F. O., Ugrinowitsch, C., Brito, L. C., Milliato, A., Marquesini, R., Moreira-Neto, A., Barbosa, E. R., Horak, F. B., Mancini, M., & Silva-Batista, C. (2021). Poor sleep quality is associated with cognitive, mobility, and anxiety disability that underlie freezing of gait in Parkinson's disease. *Gait & Posture*, *85*, 157–163. https://doi.org/10.1016/j.gaitpost.2021.01.026

De Lucia, N., De Rosa, A., Perillo, S., Sperandeo, R., De Michele, G., & Maldonato, N. M. (2023). Introversion and neuroticism in akinetic-rigid Parkinson's disease: Association with frontal-executive dysfunction. *The Journal of Neuropsychiatry and Clinical Neurosciences*, *35*(3), 228–235. https://doi.org/10.1176/appi.neuropsych.20220115

Dewey, R. B., Taneja, A., McClintock, S. M., Munro Cullum, C., Dewey, R. B., Bernstein, I., & Husain, M. M. (2012). Motor symptoms at onset of Parkinson disease and risk for cognitive impairment and depression. *Cognitive and Behavioral Neurology*, *25*(3), 115–120. https://doi.org/10.1097/WNN.0b013e31826dfd62

Di Battista, M. E., Cova, I., Rubino, A., Papi, C. P., Alampi, G., Purcaro, C., Vanacore, N., Pascale, E., Locuratolo, N., Fattapposta, F., Mariani, C., Pomati, S., & Meco, G. (2018). Intercepting Parkinson disease non-motor subtypes: A proof-of-principle study in a clinical setting. *Journal of the Neurological Sciences*, *388*, 186–191. https://doi.org/10.1016/j.jns.2018.03.024

D'Iorio, A., Maggi, G., Vitale, C., Amboni, M., Di Meglio, D., Trojano, L., & Santangelo, G. (2019). Prospective memory in Parkinson's disease: The role of the motor subtypes. *Journal of Neurology*, *266*(10), 2505–2511. https://doi.org/10.1007/s00415-019-09448-0

Domellöf, M. E., Elgh, E., & Forsgren, L. (2011). The relation between cognition and motor dysfunction in drug-naive newly diagnosed patients with Parkinson's disease. *Movement Disorders*, *26*(12), 2183–2189. https://doi.org/10.1002/mds.23814

Duncan, G. W., Khoo, T. K., Yarnall, A. J., O'Brien, J. T., Coleman, S. Y., Brooks, D. J., Barker, R. A., & Burn, D. J. (2014). Health-related quality of life in early Parkinson's disease: The impact of nonmotor symptoms. *Movement Disorders*, *29*(2), 195–202. https://doi.org/10.1002/mds.25664

Ehm, G., Lee, W.-W., Jin Jung, Y., Kim, H.-J., & Jeon, B. (2019). Clinical differences in patients with Parkinson's disease according to tandem gait performance. *Journal of Clinical Neuroscience*, *60*, 93–95. https://doi.org/10.1016/j.jocn.2018.09.022

Erro, R., Santangelo, G., Picillo, M., Vitale, C., Amboni, M., Longo, K., Giordano, F., Moccia, M., Barone, P., & Pellecchia, M. T. (2013). Side of onset does not influence cognition in newly diagnosed untreated Parkinson's disease patients. *Parkinsonism & Related Disorders*, *19*(2), 256–259. https://doi.org/10.1016/j.parkreldis.2012.10.020

Fernandez-Baizan, C., Paula Fernandez Garcia, M., Diaz-Caceres, E., Menendez-Gonzalez, M., Arias, J. L., & Mendez, M. (2020). Patients with Parkinson's disease show alteration in their visuospatial abilities and in their egocentric and allocentric spatial orientation measured by card placing tests. *Journal of Parkinson's Disease*, *10*(4), 1807–1816. https://doi.org/10.3233/JPD-202122

Freitas, T. B., Bonuzzi, G. M. G., Nuvolini, R. A., Silva, K. G., Palma, G. C. S., Freudenheim, A. M., Pompeu, J. E., & Torriani-Pasin, C. (2023). Do motor subtypes of Parkinson's disease impact the learning of motor tasks? *Brazilian Journal of Motor Behavior*, *17*(4), 99–107. https://doi.org/10.20338/bjmb.v17i4.361

Gan, Y., Xie, H., Qin, G., Wu, D., Shan, M., Hu, T., Yin, Z., An, Q., Ma, R., Wang, S., Zhang, Q., Zhu, G., & Zhang, J. (2023). Association between cognitive impairment and freezing of gait in patients with Parkinson's disease. *Journal of Clinical Medicine*, *12*(8), 2799. https://doi.org/10.3390/jcm12082799

Herman, T., Weiss, A., Brozgol, M., Wilf-Yarkoni, A., Giladi, N., & Hausdorff, J. M. (2015). Cognitive function and other non-motor features in non-demented Parkinson's disease motor subtypes. *Journal of Neural Transmission*, *122*(8), 1115–1124. https://doi.org/10.1007/s00702-014-1349-1

Hu, J., Xiao, C., Gong, D., Qiu, C., Liu, W., & Zhang, W. (2019). Regional homogeneity analysis of major Parkinson's disease subtypes based on functional magnetic resonance imaging. *Neuroscience Letters*, *706*, 81–87. https://doi.org/10.1016/j.neulet.2019.05.013

Hu, X., Jiang, Y., Jiang, X., Zhang, J., Liang, M., Li, J., Zhang, Y., Yao, D., Luo, C., & Wang, J. (2017). Altered functional connectivity density in subtypes of Parkinson's disease. *Frontiers in Human Neuroscience*, *11*, 458. https://doi.org/10.3389/fnhum.2017.00458

Huber, S., Christy, J., & Paulson, G. (1991). Cognitive heterogeneity associated with clinical subtypes of Parkinson's disease. *Neuropsychiatry, Neuropsychology, and Behavioural Neurology*, *4*(2), 147–157.

Hurt, C. S., Alkufri, F., Brown, R. G., Burn, D. J., Hindle, J. V., Landau, S., Wilson, K. C., Samuel, M., & on behalf of the PROMS-PD study group. (2014). Motor phenotypes, medication and mood: Further associations with impulsive behaviours in Parkinson's disease. *Journal of Parkinson's Disease*, *4*(2), 245–254. https://doi.org/10.3233/JPD-130314

Jeong, S. H., Kim, S. H., Park, C. W., Lee, H. S., Lee, P. H., Kim, Y. J., Sohn, Y. H., Jeong, Y., & Chung, S. J. (2023). Differential implications of cerebral hypoperfusion and hyperperfusion in Parkinson's disease. *Movement Disorders*, *38*(10), 1881–1890. https://doi.org/10.1002/mds.29565

Jin, C., Qi, S., Yang, L., Teng, Y., Li, C., Yao, Y., Ruan, X., & Wei, X. (2023). Abnormal functional connectivity density involvement in freezing of gait and its application for subtyping Parkinson's disease. *Brain Imaging and Behavior*, *17*(4), 375–385. https://doi.org/10.1007/s11682-023-00765-7

Johnson, A. R., Bucks, R. S., Kane, R. T., Thomas, M. G., Gasson, N., & Loftus, A. M. (2016). Motor subtype as a predictor of future working memory performance in idiopathic Parkinson's disease. *PLOS ONE*, *11*(3), e0152534. https://doi.org/10.1371/journal.pone.0152534

Karunanayaka, P. R., Lee, E.-Y., Lewis, M. M., Sen, S., Eslinger, P. J., Yang, Q. X., & Huang, X. (2016). Default mode network differences between rigidity- and tremor-predominant Parkinson's disease. *Cortex*, *81*, 239–250. https://doi.org/10.1016/j.cortex.2016.04.021

Katzen, H. L., Levin, B. E., & Weiner, W. (2006). Side and type of motor symptom influence cognition in Parkinson's disease. *Movement Disorders*, *21*(11), 1947–1953. https://doi.org/10.1002/mds.21105

Keener, A. M., Paul, K. C., Folle, A., Bronstein, J. M., & Ritz, B. (2018). Cognitive impairment and mortality in a population-based Parkinson's disease cohort. *Journal of Parkinson's Disease*, *8*(2), 353–362. https://doi.org/10.3233/JPD-171257

Klotzbier, T., Schott, N., & Almeida, Q. (2022). Profiles of motor-cognitive interference in Parkinson's disease—The Trail-Walking Test to discriminate between motor phenotypes. *Brain Sciences*, *12*(9), 1217.

Kwon, K., Lee, E. J., Lee, M., Ju, H., & Im, K. (2021). Impact of motor subtype on non-motor symptoms and fall-related features in patients with early Parkinson's disease. *Geriatrics & Gerontology International*, *21*(5), 416–420. https://doi.org/10.1111/ggi.14156

Lally, H., Hart, A. R., Bay, A. A., Kim, C., Wolf, S. L., & Hackney, M. E. (2020). Association between motor subtype and visuospatial and executive function in mild-moderate Parkinson disease. *Archives of Physical Medicine and Rehabilitation*, *101*(9), 1580–1589. https://doi.org/10.1016/j.apmr.2020.05.018

Li, J., Zhang, Y., Huang, Z., Jiang, Y., Ren, Z., Liu, D., Zhang, J., La Piana, R., & Chen, Y. (2022). Cortical and subcortical morphological alterations in motor subtypes of Parkinson's disease. *Npj Parkinson's Disease*, *8*(1), 167. https://doi.org/10.1038/s41531-022-00435-3

Lichter, D. G., Benedict, R. H. B., & Hershey, L. A. (2018). Importance of balance-gait disorder as a risk factor for cognitive impairment, dementia and related non-motor symptoms in Parkinson's disease. *Journal of Parkinson's Disease*, *8*(4), 539–552. https://doi.org/10.3233/JPD-181375

Lichter, D. G., Benedict, R. H. B., & Hershey, L. A. (2021). Freezing of gait in Parkinson's disease: Risk factors, their interactions, and associated nonmotor symptoms. *Parkinson's Disease*, *2021*, 1–12. https://doi.org/10.1155/2021/8857204

Liu, X., Liu, X., Liu, Y., Yang, B., Li, Y., Li, F., Qian, K., Zu, J., Zhang, W., Zhou, S., Zhang, T., Liu, J., Cui, G., & Xu, C. (2024). Utility of serum neurofilament light chain and glial fibrillary acidic protein as diagnostic biomarkers of freezing of gait in Parkinson's disease. *Brain Research*, *1822*, 148660. https://doi.org/10.1016/j.brainres.2023.148660

Lord, S., Galna, B., Coleman, S., Yarnall, A., Burn, D., & Rochester, L. (2014). Cognition and gait show a selective pattern of association dominated by phenotype in incident Parkinson's disease. *Frontiers in Aging Neuroscience*, *6*, 249. https://doi.org/10.3389/fnagi.2014.00249

Lord, S. R., Bindels, H., Ketheeswaran, M., Brodie, M. A., Lawrence, A. D., Close, J. C. T., Whone, A. L., Ben-Shlomo, Y., & Henderson, E. J. (2020). Freezing of gait in

people with Parkinson's disease: Nature, occurrence, and risk factors. *Journal of Parkinson's Disease*, *10*(2), 631–640. https://doi.org/10.3233/JPD-191813

Luo, C., Gao, Y., Hu, N., Wei, X., Xiao, Y., Wang, W., Lui, S., & Gong, Q. (2021). Distinct hippocampal subfield atrophy in Parkinson's disease regarding motor subtypes. *Parkinsonism & Related Disorders*, *93*, 66–70. https://doi.org/10.1016/j.parkreldis.2021.11.011

Lyros, E., Messinis, L., & Papathanasopoulos, P. (2008). Does motor subtype influence neurocognitive performance in Parkinson's disease without dementia? *European Journal of Neurology*, *15*(3), 262–267. https://doi.org/10.1111/j.1468-1331.2007.02046.x

Maggi, G., D'Iorio, A., Di Meglio, D., Vinciguerra, A., Amboni, M., Vitale, C., & Santangelo, G. (2020). The role of the motor subtypes on the relationship between anxiety and cognitive dysfunctions in Parkinson's disease. *Journal of Neural Transmission*, *127*(6), 893–898. https://doi.org/10.1007/s00702-020-02179-x

Maidan, I., Bernad-Elazari, H., Giladi, N., Hausdorff, J. M., & Mirelman, A. (2017). When is higher level cognitive control needed for locomotor tasks among patients with Parkinson's disease? *Brain Topography*, *30*(4), 531–538. https://doi.org/10.1007/s10548-017-0564-0

Malek, N., Lawton, M. A., Swallow, D. M. A., Grosset, K. A., Marrinan, S. L., Bajaj, N., Barker, R. A., Burn, D. J., Hardy, J., Morris, H. R., Williams, N. M., Wood, N., Ben-Shlomo, Y., Grosset, D. G., & on behalf of the PRoBaND Clinical Consortium. (2016). Vascular disease and vascular risk factors in relation to motor features and cognition in early Parkinson's disease. *Movement Disorders*, *31*(10), 1518–1526. https://doi.org/10.1002/mds.26698

Mao, C. J., Xiong, Y. T., Wang, F., Yang, Y. P., Yuan, W., Zhu, C., Chen, J., & Liu, C. F. (2018). Motor subtypes and other risk factors associated with drooling in Parkinson's disease patients. *Acta Neurologica Scandinavica*, *137*(5), 509–514. https://doi.org/10.1111/ane.12893

Marek, K., Chowdhury, S., Siderowf, A., Lasch, S., Coffey, C. S., Caspell-Garcia, C., Simuni, T., Jennings, D., Tanner, C. M., Trojanowski, J. Q., Shaw, L. M., Seibyl, J., Schuff, N., Singleton, A., Kieburtz, K., Toga, A. W., Mollenhauer, B., Galasko, D., Chahine, L. M., … the Parkinson's Progression Markers Initiative. (2018). The Parkinson's Progression Markers Initiative (PPMI) – Establishing a PD biomarker cohort. *Annals*

*of Clinical and Translational Neurology*, *5*(12), 1460–1477. https://doi.org/10.1002/acn3.644

Margolesky, J., Bette, S., Shpiner, D. S., Jordan, E. A., Dong, C., Rundek, T., Luca, C. C., Moore, H., & Singer, C. (2019). Tandem gait abnormality in Parkinson disease: Prevalence and implication as a predictor of fall risk. *Parkinsonism & Related Disorders*, *63*, 83–87. https://doi.org/10.1016/j.parkreldis.2019.02.034

Minibajeva, O., Zeltiņa, E., Karelis, G., Kurjāne, N., & Ķēniņa, V. (2023). Clinical symptoms influencing Parkinson's patients' quality of life in Latvia: A single-center cohort study. *Medicina*, *59*(5), 935. https://doi.org/10.3390/medicina59050935

Moretti, R., Caruso, P., Monguzzi, G., Sala, A., Dal Ben, M., & Gazzin, S. (2020). Is Parkinson's disease an unique clinical entity? Rigid or tremor dominant PD: Two faces of the same coin. *Journal of Clinical Neuroscience*, *74*, 18–24. https://doi.org/10.1016/j.jocn.2020.01.068

Moretti, R., Milner, V., Caruso, P., Gazzin, S., & Rumiati, R. (2017). Frontal tasks and behavior in rigid or tremor-dominant Parkinson disease. *American Journal of Alzheimer's Disease & Other Dementias*, *32*(5), 300–306. https://doi.org/10.1177/1533317517714887

Moretti, R., Torre, P., Antonello, R. M., Rosin, M. V., Esposito, F., Furman, M. R., & Bellini, G. (2012). Apathy: A complex symptom specific to the clinical pattern of presentation of Parkinson's disease? *American Journal of Alzheimer's Disease & Other Dementias*, *27*(3), 196–201. https://doi.org/10.1177/1533317512445502

Moustafa, A. A., Bell, P., Eissa, A. M., & Hewedi, D. H. (2013). The effects of clinical motor variables and medication dosage on working memory in Parkinson's disease. *Brain and Cognition*, *82*(2), 137–145. https://doi.org/10.1016/j.bandc.2013.04.001

Moustafa, A. A., Krishna, R., Eissa, A. M., & Hewedi, D. H. (2013). Factors underlying probabilistic and deterministic stimulus-response learning performance in medicated and unmedicated patients with Parkinson's disease. *Neuropsychology*, *27*(4), 498–510. https://doi.org/10.1037/a0032757

Mun, J. K., Youn, J., Cho, J. W., Oh, E.-S., Kim, J. S., Park, S., Jang, W., Park, J. S., Koh, S.-B., Lee, J. H., Park, H. K., Kim, H.-J., Jeon, B. S., Shin, H.-W., Choi, S.-A., Kim, S. J., Choi, S.-M., Park, J.-Y., Kim, J. Y., … Kwon, D.-Y. (2016). Weight change is a characteristic non-motor symptom in drug-naïve Parkinson's disease patients with non-tremor dominant subtype: A nation-wide observational study. *PLOS ONE*, *11*(9), e0162254. https://doi.org/10.1371/journal.pone.0162254

Nantel, J., McDonald, J. C., Tan, S., & Bronte-Stewart, H. (2012). Deficits in visuospatial processing contribute to quantitative measures of freezing of gait in Parkinson's disease. *Neuroscience*, *221*, 151–156. https://doi.org/10.1016/j.neuroscience.2012.07.007

Nazmuddin, M., Van Dalen, J.-W., Borra, R. J. H., Stormezand, G. N., Van Der Horn, H. J., Van Der Zee, S., Boertien, J., & Van Laar, T. (2021). Postural and gait symptoms in de novo Parkinson's disease patients correlate with cholinergic white matter pathology. *Parkinsonism & Related Disorders*, *93*, 43–49. https://doi.org/10.1016/j.parkreldis.2021.11.010

Ng, A. S. L., Tan, Y. J., Yong, A. C. W., Saffari, S. E., Lu, Z., Ng, E. Y., Ng, S. Y. E., Chia, N. S. Y., Choi, X., Heng, D., Neo, S., Xu, Z., Keong, N. C. H., Tay, K. Y., Au, W. L., Tan, L. C. S., & Tan, E.-K. (2020). Utility of plasma neurofilament light as a diagnostic and prognostic biomarker of the postural instability gait disorder motor subtype in early Parkinson's disease. *Molecular Neurodegeneration*, *15*(1), 33. https://doi.org/10.1186/s13024-020-00385-5

Nyberg, E. M., Tanabe, J., Honce, J. M., Krmpotich, T., Shelton, E., Hedeman, J., & Berman, B. D. (2015). Morphologic changes in the mesolimbic pathway in Parkinson's disease motor subtypes. *Parkinsonism & Related Disorders*, *21*(5), 536–540. https://doi.org/10.1016/j.parkreldis.2015.03.008

Önder, H., & Ozyurek, O. (2021). The impact of distinct cognitive dual-tasks on gait in Parkinson's disease and the associations with the clinical features of Parkinson's disease. *Neurological Sciences*, *42*(7), 2775–2783. https://doi.org/10.1007/s10072-020-04874-9

Ortelli, P., Ferrazzoli, D., Cian, V., Zarucchi, M., Palamara, G., Giobbia, A., Frazzitta, G., Maestri, R., & Canesi, M. (2019). How cognition and motivation "freeze" the motor behavior in Parkinson's disease. *Frontiers in Neuroscience*, *13*, 1302. https://doi.org/10.3389/fnins.2019.01302

Ou, R., Wei, Q., Hou, Y., Zhang, L., Liu, K., Lin, J., Jiang, Z., Zhao, B., Cao, B., & Shang, H. (2021). Facial tremor in patients with Parkinson's disease: Prevalence, determinants and impacts on disease progression. *BMC Neurology*, *21*(1), 86. https://doi.org/10.1186/s12883-021-02105-y

Paulus, W., & Jellinger, K. (1991). The neuropathologic basis of different clinical subgroups of Parkinson's disease. *Journal of Neuropathology & Experimental Neurology*, *50*(6), 743–755. https://doi.org/10.1097/00005072-199111000-00006

Pelicioni, P. H. S., Menant, J. C., Henderson, E. J., Latt, M. D., Brodie, M. A., & Lord, S. R. (2021). Mild and marked executive dysfunction and falls in people with Parkinson's disease. *Brazilian Journal of Physical Therapy*, *25*(4), 437–443. https://doi.org/10.1016/j.bjpt.2020.11.005

Pelicioni, P. H. S., Menant, J. C., Latt, M. D., & Lord, S. R. (2019). Falls in Parkinson's disease subtypes: Risk factors, locations and circumstances. *International Journal of Environmental Research and Public Health*, *16*(12), 2216. https://doi.org/10.3390/ijerph16122216

Pereira, M. P., Batistela, R. A., Rocha Dos Santos, P. C., Simieli, L., & Gobbi, L. T. B. (2019). The dual-tasking overload on functional mobility is related to specific cognitive domains in different subtypes of Parkinson's disease. *Topics in Geriatric Rehabilitation*, *35*(2), 119–124. https://doi.org/10.1097/TGR.0000000000000220

Petrijan, T., Zmazek, J., & Menih, M. (2023). Parkinson's disease non-motor subtypes classification in a group of Slovenian patients: Actuarial vs. data-driven approach. *Journal of Clinical Medicine*, *12*(23), 7434. https://doi.org/10.3390/jcm12237434

Pietracupa, S., Suppa, A., Upadhyay, N., Giannì, C., Grillea, G., Leodori, G., Modugno, N., Di Biasio, F., Zampogna, A., Colonnese, C., Berardelli, A., & Pantano, P. (2018). Freezing of gait in Parkinson's disease: Gray and white matter abnormalities. *Journal of Neurology*, *265*(1), 52–62. https://doi.org/10.1007/s00415-017-8654-1

Poletti, M., Frosini, D., Pagni, C., Baldacci, F., Nicoletti, V., Tognoni, G., Lucetti, C., Del Dotto, P., Ceravolo, R., & Bonuccelli, U. (2012). Mild cognitive impairment and cognitive-motor relationships in newly diagnosed drug-naive patients with Parkinson's disease. *Journal of Neurology, Neurosurgery & Psychiatry*, *83*(6), 601–606. https://doi.org/10.1136/jnnp-2011-301874

Pongmala, C., Roytman, S., Van Emde Boas, M., Dickinson, O., Kanel, P., & Bohnen, N. I. (2023). Composite measures of motor performance and self-efficacy are better determinants of postural instability and gait difficulties than individual clinical measures in Parkinson's disease. *Parkinsonism & Related Disorders*, *107*, 105251. https://doi.org/10.1016/j.parkreldis.2022.105251

Pötter-Nerger, M., Dutke, J., Lezius, S., Buhmann, C., Schulz, R., Gerloff, C., Kuhle, J., & Choe, C. (2022). Serum neurofilament light chain and postural instability/gait difficulty (PIGD) subtypes of Parkinson's disease in the MARK-PD study. *Journal of Neural Transmission*, *129*(3), 295–300. https://doi.org/10.1007/s00702-022-02464-x

Raffo De Ferrari, A., Lagravinese, G., Pelosin, E., Pardini, M., Serrati, C., Abbruzzese, G., & Avanzino, L. (2015). Freezing of gait and affective theory of mind in Parkinson disease. *Parkinsonism & Related Disorders*, *21*(5), 509–513. https://doi.org/10.1016/j.parkreldis.2015.02.023

Rana, A. Q., Vaid, H. M., Edun, A., Dogu, O., & Rana, M. A. (2012). Relationship of dementia and visual hallucinations in tremor and non-tremor dominant Parkinson's disease. *Journal of the Neurological Sciences*, *323*(1–2), 158–161. https://doi.org/10.1016/j.jns.2012.09.007

Ren, J., Hua, P., Li, Y., Pan, C., Yan, L., Yu, C., Zhang, L., Xu, P., Zhang, M., & Liu, W. (2020). Comparison of three motor subtype classifications in de novo Parkinson's disease patients. *Frontiers in Neurology*, *11*, 601225. https://doi.org/10.3389/fneur.2020.601225

Santos-García, D., De Deus-Fonticoba, T., Suárez Castro, E., M Aneiros Díaz, Á., Feal-Painceiras, M. J., Paz-González, J. M., García-Sancho, C., Jesús, S., Mir, P., Planellas, L., García-Caldentey, J., Caballol, N., Legarda, I., Hernández-Vara, J., González-Aramburu, I., Ávila-Rivera, M. A., Catalán, M. J., Nogueira, V., Álvarez-Sauco, M., … on behalf of the COPPADIS Study Group. (2020). The impact of freezing of gait on functional dependency in Parkinson's disease with regard to motor phenotype. *Neurological Sciences*, *41*(10), 2883–2892. https://doi.org/10.1007/s10072-020-04404-7

Sarasso, E., Basaia, S., Cividini, C., Stojkovic, T., Stankovic, I., Piramide, N., Tomic, A., Markovic, V., Stefanova, E., Kostic, V. S., Filippi, M., & Agosta, F. (2022). MRI biomarkers of freezing of gait development in Parkinson's disease. *Npj Parkinson's Disease*, *8*(1), 158. https://doi.org/10.1038/s41531-022-00426-4

Sawada, M., Wada-Isoe, K., Hanajima, R., & Nakashima, K. (2019). Clinical features of freezing of gait in Parkinson's disease patients. *Brain and Behavior*, *9*(4), e01244. https://doi.org/10.1002/brb3.1244

Shen, B., Pan, Y., Jiang, X., Wu, Z., Zhu, J., Dong, J., Zhang, W., Xu, P., Dai, Y., Gao, Y., Xiao, C., & Zhang, L. (2020). Altered putamen and cerebellum connectivity among different subtypes of Parkinson's disease. *CNS Neuroscience & Therapeutics*, *26*(2), 207–214. https://doi.org/10.1111/cns.13259

Shen, D., Cao, L., Ling, Y., Li, D., Ren, K., Shi, W., Chen, Z., Zhou, H., & Liu, J. (2023). Bilateral globus pallidus interna deep brain stimulation in Parkinson's disease: Therapeutic effects and motor outcomes prediction in a short-term follow up.

*Frontiers in Human Neuroscience*, *16*, 1023917. https://doi.org/10.3389/fnhum.2022.1023917

Shkodina, A. D., Tarianyk, K. A., Boiko, D. I., Zehravi, M., Akter, S., Md. Ashraf, G., & Rahman, Md. H. (2022). Cognitive and affective disturbances in patients with Parkinson's disease: Perspectives for classifying of motor/neuropsychiatric subtypes. *Neuroscience Letters*, *781*, 136675. https://doi.org/10.1016/j.neulet.2022.136675

Si, Q.-Q., Yuan, Y.-S., Zhi, Y., Tong, Q., Zhang, L., & Zhang, K. (2018). Plasma transferrin level correlates with the tremor-dominant phenotype of Parkinson's disease. *Neuroscience Letters*, *684*, 42–46. https://doi.org/10.1016/j.neulet.2018.07.004

Song, C., Shen, Q., Tan, C., Li, J., Zhou, F., Wang, T., Zhang, L., Wang, M., Liu, Y., Yuan, J., Cai, S., & Liao, H. (2023). Distinct changes in global brain synchronization in different motor subtypes of Parkinson's disease. *Frontiers in Neuroscience*, *17*, 1170225. https://doi.org/10.3389/fnins.2023.1170225

Stegemöller, E. L., Vallabhajosula, S., Haq, I., Hwynn, N., Hass, C. J., & Okun, M. S. (2013). Selective use of low frequency stimulation in Parkinson's disease based on absence of tremor. *NeuroRehabilitation*, *33*(2), 305–312. https://doi.org/10.3233/NRE-130960

Stojkovic, T., Stefanova, E., Soldatovic, I., Markovic, V., Stankovic, I., Petrovic, I., Agosta, F., Galantucci, S., Filippi, M., & Kostic, V. (2018). Exploring the relationship between motor impairment, vascular burden and cognition in Parkinson's disease. *Journal of Neurology*, *265*(6), 1320–1327. https://doi.org/10.1007/s00415-018-8838-3

Sunwoo, M. K., Cho, K. H., Hong, J. Y., Lee, J. E., Sohn, Y. H., & Lee, P. H. (2013). Thalamic volume and related visual recognition are associated with freezing of gait in non-demented patients with Parkinson's disease. *Parkinsonism & Related Disorders*, *19*(12), 1106–1109. https://doi.org/10.1016/j.parkreldis.2013.07.023

Surova, Y., Lampinen, B., Nilsson, M., Lätt, J., Hall, S., Widner, H., Swedish BioFINDER study, Van Westen, D., & Hansson, O. (2016). Alterations of diffusion kurtosis and neurite density measures in deep grey matter and white matter in Parkinson's disease. *PLOS ONE*, *11*(6), e0157755. https://doi.org/10.1371/journal.pone.0157755

Tang, X., Zhang, Y., Liu, D., Hu, Y., Jiang, L., & Zhang, J. (2021). Association of gyrification pattern, white matter changes, and phenotypic profile in patients with Parkinson disease. *Neurology*, *96*(19), e2387–e2394. https://doi.org/10.1212/WNL.0000000000011894

Taylor, J.-P., Rowan, E. N., Lett, D., O'Brien, J. T., McKeith, I. G., & Burn, D. J. (2008). Poor attentional function predicts cognitive decline in patients with non-demented Parkinson's disease independent of motor phenotype. *Journal of Neurology, Neurosurgery & Psychiatry*, *79*(12), 1318–1323. https://doi.org/10.1136/jnnp.2008.147629

Tolleson, C., Turchan, M., Van Wouwe, N., Isaacs, D., Phibbs, F., & Wylie, S. (2017). Parkinson's disease subtypes show distinct tradeoffs between response initiation and inhibition latencies. *Journal of the International Neuropsychological Society*, *23*(8), 665–674. https://doi.org/10.1017/S1355617717000467

Tomer, R., & Aharon-Peretz, J. (2002). Dissociation between spontaneous and reactive flexibility in early Parkinson's disease. *Neuropsychiatry, Neuropsychology and Behavioral Neurology*, *15*(2), 106–112.

Vakil, E., & Herishanu-Naaman, S. (1998). Declarative and procedural learning in Parkinson's disease patients having tremor or bradykinesia as the predominant symptom. *Cortex*, *34*(4), 611–620. https://doi.org/10.1016/S0010-9452(08)70518-5

van Nuland, A. J., Helmich, R. C., Dirkx, M. F., Zach, H., Toni, I., Cools, R., & den Ouden, H. E. M. (2020). Effects of dopamine on reinforcement learning in Parkinson's disease depend on motor phenotype. *Brain*, *143*(11), 3422–3434. https://doi.org/10.1093/brain/awaa335

Vercruysse, S., Devos, H., Munks, L., Spildooren, J., Vandenbossche, J., Vandenberghe, W., Nieuwboer, A., & Heremans, E. (2012). Explaining freezing of gait in Parkinson's disease: Motor and cognitive determinants. *Movement Disorders*, *27*(13), 1644–1651. https://doi.org/10.1002/mds.25183

Vervoort, G., Bengevoord, A., Nackaerts, E., Heremans, E., Vandenberghe, W., & Nieuwboer, A. (2015). Distal motor deficit contributions to postural instability and gait disorder in Parkinson's disease. *Behavioural Brain Research*, *287*, 1–7. https://doi.org/10.1016/j.bbr.2015.03.026

Wan, Y., Hu, W., Gan, J., Song, L., Wu, N., Chen, Y., & Liu, Z. (2019). Exploring the association between cerebral small-vessel diseases and motor symptoms in Parkinson's disease. *Brain and Behavior*, *9*(4), e01219. https://doi.org/10.1002/brb3.1219

Wang, F., Sun, L., Zhang, X., Jia, J., Liu, Z., Huang, X., Yu, S., Zuo, L., Cao, C., Wang, X., & Zhang, W. (2015). Effect and potential mechanism of electroacupuncture add-on

treatment in patients with Parkinson's disease. *Evidence-Based Complementary and Alternative Medicine*, *2015*, 1–11. https://doi.org/10.1155/2015/692795

Wang, J., Bi, Q., Gong, W., Zhang, H., Deng, M., Chen, L., & Wang, B. (2023). Histogram analysis of diffusion kurtosis imaging of deep brain nuclei in Parkinson's disease with different motor subtypes. *Clinical Radiology*, *78*(12), e966–e974. https://doi.org/10.1016/j.crad.2023.09.008

Wang, J., Shen, Y., Peng, J., Wang, A., Wu, X., Chen, X., Liu, J., Wei, M., Zou, D., Han, Y., & Cheng, O. (2021). Different functional connectivity modes of the right fronto-insular cortex in akinetic-rigid and tremor-dominant Parkinson's disease. *Neurological Sciences*, *42*(7), 2937–2946. https://doi.org/10.1007/s10072-020-04917-1

Wang, L., Gan, C., Sun, H., Ji, M., Zhang, H., Cao, X., Wang, M., Yuan, Y., & Zhang, K. (2023). Impaired structural and reserved functional topological organizations of brain networks in Parkinson's disease with freezing of gait. *Quantitative Imaging in Medicine and Surgery*, *13*(1), 66–79. https://doi.org/10.21037/qims-22-351

Wang, L., Ji, M., Sun, H., Gan, C., Zhang, H., Cao, X., Yuan, Y., & Zhang, K. (2022). Reduced short-latency afferent inhibition in Parkinson's disease patients with L-dopa-unresponsive freezing of gait. *Journal of Parkinson's Disease*, *12*(8), 2507–2518. https://doi.org/10.3233/JPD-223498

Wang, L., Yan, Y., Zhang, L., Liu, Y., Luo, R., & Chang, Y. (2021). Substantia nigra neuromelanin magnetic resonance imaging in patients with different subtypes of Parkinson disease. *Journal of Neural Transmission*, *128*(2), 171–179. https://doi.org/10.1007/s00702-020-02295-8

Wang, Y., Li, D., Chen, Y., Zhu, S., Jiang, X., Jiang, Y., Gu, R., Shen, B., Zhu, J., Pan, Y., Yan, J., & Zhang, L. (2023). Clinical features of minor hallucinations in different phenotypes of Parkinson's disease: A cross-sectional study. *Frontiers in Neurology*, *14*, 1158188. https://doi.org/10.3389/fneur.2023.1158188

Wang, Y., Tang, B., Yan, X., Chen, Z., Xu, Q., Liu, Z., Li, K., Wang, K., & Guo, J. (2015). A neurophysiological profile in Parkinson's disease with mild cognitive impairment and dementia in China. *Journal of Clinical Neuroscience*, *22*(6), 981–985. https://doi.org/10.1016/j.jocn.2014.11.030

Wang, Z., & You, Z. (2023). Impacts of motor phenotype on cognitive function in patients with Parkinson's disease 1 year after subthalamic-nucleus deep brain stimulation. *Geriatrics & Gerontology International*, *23*(2), 85–90. https://doi.org/10.1111/ggi.14524

Williams-Gray, C. H., Foltynie, T., Brayne, C. E. G., Robbins, T. W., & Barker, R. A. (2007). Evolution of cognitive dysfunction in an incident Parkinson's disease cohort. *Brain*, *130*(7), 1787–1798. https://doi.org/10.1093/brain/awm111

Wojtala, J., Heber, I. A., Neuser, P., Heller, J., Kalbe, E., Rehberg, S. P., Storch, A., Linse, K., Schneider, C., Gräber, S., Berg, D., Dams, J., Balzer-Geldsetzer, M., Hilker-Roggendorf, R., Oberschmidt, C., Baudrexel, S., Witt, K., Schmidt, N., Deuschl, G., … Reetz, K. (2019). Cognitive decline in Parkinson's disease: The impact of the motor phenotype on cognition. *Journal of Neurology, Neurosurgery & Psychiatry*, *90*(2), 171–179. https://doi.org/10.1136/jnnp-2018-319008

Wolters, A. F., Michielse, S., Kuijf, M. L., Defebvre, L., Lopes, R., Dujardin, K., & Leentjens, A. F. G. (2022). Brain network characteristics and cognitive performance in motor subtypes of Parkinson's disease: A resting state fMRI study. *Parkinsonism & Related Disorders*, *105*, 32–38. https://doi.org/10.1016/j.parkreldis.2022.10.027

Wu, D.-D., Su, W., He, J., Li, S.-H., Li, K., & Chen, H.-B. (2022). Nonmotor symptoms and quality of life in Parkinson's disease with different motor subtypes. *Zeitschrift Für Gerontologie Und Geriatrie*, *55*(6), 496–501. https://doi.org/10.1007/s00391-021-01950-3

Wu, Y., Guo, X.-Y., Wei, Q.-Q., Ou, R.-W., Song, W., Cao, B., Zhao, B., & Shang, H.-F. (2016). Non-motor symptoms and quality of life in tremor dominant vs postural instability gait disorder Parkinson's disease patients. *Acta Neurologica Scandinavica*, *133*(5), 330–337. https://doi.org/10.1111/ane.12461

Wylie, S. A., Van Den Wildenberg, W., Ridderinkhof, K. R., Claassen, D. O., Wooten, G. F., & Manning, C. A. (2012). Differential susceptibility to motor impulsivity among functional subtypes of Parkinson's disease. *Journal of Neurology, Neurosurgery & Psychiatry*, *83*(12), 1149–1154. https://doi.org/10.1136/jnnp-2012-303056

Yang, Q., Nanivadekar, S., Taylor, P. A., Dou, Z., Lungu, C. I., & Horovitz, S. G. (2021). Executive function network's white matter alterations relate to Parkinson's disease motor phenotype. *Neuroscience Letters*, *741*, 135486. https://doi.org/10.1016/j.neulet.2020.135486

Yang, X., Li, Z., Bai, L., Shen, X., Wang, F., Han, X., Zhang, R., Li, Z., Zhang, J., Dong, M., Wang, Y., Cao, T., Zhao, S., Chu, C., Liu, C., & Zhu, X. (2022). Association of plasma and electroencephalography markers with motor subtypes of Parkinson's disease. *Frontiers in Aging Neuroscience*, *14*, 911221. https://doi.org/10.3389/fnagi.2022.911221

Yu, Q., Zou, X., Quan, F., Dong, Z., Yin, H., Liu, J., Zuo, H., Xu, J., Han, Y., Zou, D., Li, Y., & Cheng, O. (2022). Parkinson's disease patients with freezing of gait have more severe voice impairment than non-freezers during "ON state." *Journal of Neural Transmission*, *129*(3), 277–286. https://doi.org/10.1007/s00702-021-02458-1

Yu, R., Wu, R., Tai, C., Lin, C., & Hua, M. (2010). Feeling-of-knowing in episodic memory in patients with Parkinson's disease with various motor symptoms. *Movement Disorders*, *25*(8), 1034–1039. https://doi.org/10.1002/mds.23017

Yu, Y., Yan, W., Xu, X., Zhang, K., Si, L., Liu, X., Wang, J., Song, J., Sun, H., & Li, X. (2022). Response times for reflexive saccades correlate with cognition in Parkinson's disease, not disease severity or duration. *Frontiers in Neurology*, *13*, 945201. https://doi.org/10.3389/fneur.2022.945201

Zhang, S., Ou, R., Chen, X., Yang, J., Zhao, B., Yuan, X., Wei, Q., Cao, B., & Shang, H.-F. (2016). Correlative factors of cognitive dysfunction in PD patients: A cross-sectional study from Southwest China. *Neurological Research*, *38*(5), 434–440. https://doi.org/10.1080/01616412.2016.1139320

Zhou, H.-Y., Huang, P., Sun, Q., Du, J.-J., Cui, S.-S., Tan, Y.-Y., Hu, Y.-Y., Zhan, W.-W., Wang, Y., Xiao, Q., Liu, J., & Chen, S.-D. (2018). Substantia nigra echogenicity associated with clinical subtypes of Parkinson's disease. *Journal of Parkinson's Disease*, *8*(2), 333–340. https://doi.org/10.3233/JPD-171264

Zuo, L.-J., Piao, Y.-S., Li, L.-X., Yu, S.-Y., Guo, P., Hu, Y., Lian, T.-H., Wang, R.-D., Yu, Q.-J., Jin, Z., Wang, Y.-J., Wang, X.-M., Chan, P., Chen, S.-D., Wang, Y.-J., & Zhang, W. (2017). Phenotype of postural instability/gait difficulty in Parkinson disease: Relevance to cognitive impairment and mechanism relating pathological proteins and neurotransmitters. *Scientific Reports*, *7*(1), 44872. https://doi.org/10.1038/srep44872

**Supplementary Material References**


Altmann, C. F., Koschel, J., & Jost, W. H. (2023). Predictors of falls in Parkinson's disease, progressive supranuclear palsy, and multiple system atrophy: A retrospective study. *Neurologia i Neurochirurgia Polska*, *57*(3), 297–304. https://doi.org/10.5603/PJNNS.a2023.0036

Alves, G., Larsen, J. P., Emre, M., Wentzel-Larsen, T., & Aarsland, D. (2006). Changes in motor subtype and risk for incident dementia in Parkinson's disease. *Movement Disorders*, *21*(8), 1123–1130. https://doi.org/10.1002/mds.20897

American Psychiatric Association. (1994). *Diagnostic and statistical manual of mental disorders* (4th ed.).

Child, B., Saywell, I., Da Silva, R., Collins-Praino, L., & Baetu, I. (2024). Cognitive function in different motor subtypes of Parkinson's disease: A systematic review protocol. *Health Science Reports*, *7*(5), e2092. https://doi.org/10.1002/hsr2.2092

Coffey, C. S., Caspell-Garcia, C. J., & Foster, E. D. (2020, May 7). *Parkinson's Progression Markers Initiative: Variable Definitions and Score Calculations*. Parkinson's Progression Markers Initiative.

Dewey, R. B. Jr., Taneja, A., McClintock, S. M., Cullum, C. M., Dewey, R. B. I., Bernstein, I., & Husain, M. M. (2012). Motor symptoms at onset of Parkinson disease and risk for cognitive impairment and depression. *Cognitive and Behavioral Neurology*, *25*(3), 115–120. https://doi.org/10.1097/WNN.0b013e31826dfd62

Ehm, G., Lee, W.-W., Jin Jung, Y., Kim, H.-J., & Jeon, B. (2019). Clinical differences in patients with Parkinson's disease according to tandem gait performance. *Journal of Clinical Neuroscience*, *60*, 93–95. Scopus. https://doi.org/10.1016/j.jocn.2018.09.022

Erro, R., Santangelo, G., Picillo, M., Vitale, C., Amboni, M., Longo, K., Giordano, F., Moccia, M., Barone, P., & Pellecchia, M. T. (2013). Side of onset does not influence

cognition in newly diagnosed untreated Parkinson's disease patients. *Parkinsonism & Related Disorders*, *19*(2), 256–259. https://doi.org/10.1016/j.parkreldis.2012.10.020

Fahn, S., Elton, R., & Members of the UPRDS Development Committee. (1987). The Unified Parkinson's Disease Rating Scale. In S. Fahn, C. D. Marsden, M. Goldstein, & D. B. Calne, *Recent developments in Parkinson's disease* (Vol. 2, pp. 153–163). Macmillan Health Care Information.

Fereshtehnejad, S.-M., & Postuma, R. B. (2017). Subtypes of Parkinson's disease: What do they tell us about disease progression? *Current Neurology and Neuroscience Reports*, *17*(4), 34. https://doi.org/10.1007/s11910-017-0738-x

Goetz, C. G., Tilley, B. C., Shaftman, S. R., Stebbins, G. T., Fahn, S., Martinez-Martin, P., Poewe, W., Sampaio, C., Stern, M. B., Dodel, R., Dubois, B., Holloway, R., Jankovic, J., Kulisevsky, J., Lang, A. E., Lees, A., Leurgans, S., LeWitt, P. A., Nyenhuis, D., … for the Movement Disorder Society UPDRS Revision Task Force. (2008). Movement Disorder Society-sponsored revision of the Unified Parkinson's Disease Rating Scale (MDS-UPDRS): Scale presentation and clinimetric testing results. *Movement Disorders*, *23*(15), 2129–2170. https://doi.org/10.1002/mds.22340

Hayden, J. A., van der Windt, D. A., Cartwright, J. L., Côté, P., & Bombardier, C. (2013). Assessing bias in studies of prognostic factors. *Annals of Internal Medicine*, *158*(4), 280. https://doi.org/10.7326/0003-4819-158-4-201302190-00009

Herman, T., Weiss, A., Brozgol, M., Giladi, N., & Hausdorff, J. M. (2014). Gait and balance in Parkinson's disease subtypes: Objective measures and classification considerations. *Journal of Neurology*, *261*(12), 2401–2410. https://doi.org/10.1007/s00415-014-7513-6

Herman, T., Weiss, A., Brozgol, M., Wilf-Yarkoni, A., Giladi, N., & Hausdorff, J. M. (2015). Cognitive function and other non-motor features in non-demented Parkinson's disease

motor subtypes. *Journal of Neural Transmission*, *122*(8), 1115–1124. https://doi.org/10.1007/s00702-014-1349-1

Huber, S. J., Christy, J. A., & Paulson, G. W. (1991). Cognitive heterogeneity associated with clinical subtypes of Parkinson's disease. *Neuropsychiatry, Neuropsychology, & Behavioral Neurology*, *4*(2), 147–157.

Jankovic, J., McDermott, M., Carter, J., Gauthier, S., Goetz, C., Golbe, L., Huber, S., Koller, W., Olanow, C., Shoulson, I., Stern, M., Tanner, C., Weiner, W., & Parkinson Study Group. (1990). Variable expression of Parkinson's disease: A base-line analysis of the DATATOP cohort. *Neurology*, *40*(10), 1529–1529. https://doi.org/10.1212/WNL.40.10.1529

Katzen, H. L., Levin, B. E., & Weiner, W. (2006). Side and type of motor symptom influence cognition in Parkinson's disease. *Movement Disorders*, *21*(11), 1947–1953. https://doi.org/10.1002/mds.21105

Litvan, I., Goldman, J. G., Tröster, A. I., Schmand, B. A., Weintraub, D., Petersen, R. C., Mollenhauer, B., Adler, C. H., Marder, K., Williams-Gray, C. H., Aarsland, D., Kulisevsky, J., Rodriguez-Oroz, M. C., Burn, D. J., Barker, R. A., & Emre, M. (2012). Diagnostic criteria for mild cognitive impairment in Parkinson's disease: Movement Disorder Society Task Force guidelines. *Movement Disorders*, *27*(3), 349–356. https://doi.org/10.1002/mds.24893

Marek, K., Chowdhury, S., Siderowf, A., Lasch, S., Coffey, C. S., Caspell-Garcia, C., Simuni, T., Jennings, D., Tanner, C. M., Trojanowski, J. Q., Shaw, L. M., Seibyl, J., Schuff, N., Singleton, A., Kieburtz, K., Toga, A. W., Mollenhauer, B., Galasko, D., Chahine, L. M., … the Parkinson's Progression Markers Initiative. (2018). The Parkinson's Progression Markers Initiative (PPMI) – Establishing a PD biomarker cohort. *Annals*

*of Clinical and Translational Neurology*, *5*(12), 1460–1477. https://doi.org/10.1002/acn3.644

Mestre, T. A., Fereshtehnejad, S.-M., Berg, D., Bohnen, N. I., Dujardin, K., Erro, R., Espay, A. J., Halliday, G., van Hilten, J. J., Hu, M. T., Jeon, B., Klein, C., Leentjens, A. F. G., Marinus, J., Mollenhauer, B., Postuma, R., Rajalingam, R., Rodríguez-Violante, M., Simuni, T., … Marras, C. (2021). Parkinson's disease subtypes: Critical appraisal and recommendations. *Journal of Parkinson's Disease*, *11*(2), 395–404. https://doi.org/10.3233/JPD-202472

Nutt, J. G. (2016). Motor subtype in Parkinson's disease: Different disorders or different stages of disease? *Movement Disorders*, *31*(7), 957–961. https://doi.org/10.1002/mds.26657

Ou, R., Wei, Q., Hou, Y., Zhang, L., Liu, K., Lin, J., Jiang, Z., Zhao, B., Cao, B., & Shang, H. (2021). Facial tremor in patients with Parkinson's disease: Prevalence, determinants and impacts on disease progression. *BMC Neurology*, *21*(1), 86. https://doi.org/10.1186/s12883-021-02105-y

Pelicioni, P. H. S., Menant, J. C., Henderson, E. J., Latt, M. D., Brodie, M. A., & Lord, S. R. (2021). Mild and marked executive dysfunction and falls in people with Parkinson's disease. *Brazilian Journal of Physical Therapy*, *25*(4), 437–443. c8h. https://doi.org/10.1016/j.bjpt.2020.11.005

Petrijan, T., Zmazek, J., & Menih, M. (2023). Parkinson's disease non-motor subtypes classification in a group of Slovenian patients: Actuarial vs. data-driven approach. *Journal of Clinical Medicine*, *12*(23), 7434. https://doi.org/10.3390/jcm12237434

Poletti, M., Frosini, D., Pagni, C., Baldacci, F., Nicoletti, V., Tognoni, G., Lucetti, C., Del Dotto, P., Ceravolo, R., & Bonuccelli, U. (2012). Mild cognitive impairment and cognitive-motor relationships in newly diagnosed drug-naive patients with

Parkinson's disease. *Journal of Neurology, Neurosurgery & Psychiatry*, *83*(6), 601–606. https://doi.org/10.1136/jnnp-2011-301874

Pustejovsky, J. E., & Rodgers, M. A. (2019). Testing for funnel plot asymmetry of standardized mean differences. *Research Synthesis Methods*, *10*(1), 57–71. https://doi.org/10.1002/jrsm.1332

Rana, A. Q., Vaid, H. M., Edun, A., Dogu, O., & Rana, M. A. (2012). Relationship of dementia and visual hallucinations in tremor and non-tremor dominant Parkinson's disease. *Journal of the Neurological Sciences*, *323*(1–2), 158–161. https://doi.org/10.1016/j.jns.2012.09.007

Ren, J., Hua, P., Li, Y., Pan, C., Yan, L., Yu, C., Zhang, L., Xu, P., Zhang, M., & Liu, W. (2020). Comparison of three motor subtype classifications in de novo Parkinson's disease patients. *Frontiers in Neurology*, *11*, 601225. https://doi.org/10.3389/fneur.2020.601225

Shen, D., Cao, L., Ling, Y., Li, D., Ren, K., Shi, W., Chen, Z., Zhou, H., & Liu, J. (2023). Bilateral globus pallidus interna deep brain stimulation in Parkinson's disease: Therapeutic effects and motor outcomes prediction in a short-term follow up. *Frontiers in Human Neuroscience*, *16*, 1023917. https://doi.org/10.3389/fnhum.2022.1023917

Stebbins, G. T., Goetz, C. G., Burn, D. J., Jankovic, J., Khoo, T. K., & Tilley, B. C. (2013). How to identify tremor dominant and postural instability/gait difficulty groups with the movement disorder society unified Parkinson's disease rating scale: Comparison with the unified Parkinson's disease rating scale. *Movement Disorders*, *28*(5), 668–670. https://doi.org/10.1002/mds.25383

Tomer, R., Fisher, T., Giladi, N., & Aharon-Peretz, J. (2002). Dissociation between spontaneous and reactive flexibility in early Parkinson's disease. *Neuropsychiatry, Neuropsychology, & Behavioral Neurology*, *15*(2), 106–112.

Vakil, E., & Herishanu-Naaman, S. (1998). Declarative and procedural learning in Parkinson's disease patients having tremor or bradykinesia as the predominant symptom. *Cortex*, *34*(4), 611–620. https://doi.org/10.1016/S0010-9452(08)70518-5

Williams-Gray, C. H., Foltynie, T., Brayne, C. E. G., Robbins, T. W., & Barker, R. A. (2007). Evolution of cognitive dysfunction in an incident Parkinson's disease cohort. *Brain*, *130*(7), 1787–1798. https://doi.org/10.1093/brain/awm111

Yu, Y., Yan, W., Xu, X., Zhang, K., Si, L., Liu, X., Wang, J., Song, J., Sun, H., & Li, X. (2022). Response times for reflexive saccades correlate with cognition in Parkinson's disease, not disease severity or duration. *Frontiers in Neurology*, *13*, 945201. https://doi.org/10.3389/fneur.2022.945201

Zhang, S., Ou, R., Chen, X., Yang, J., Zhao, B., Yuan, X., Wei, Q., Cao, B., & Shang, H.-F. (2016). Correlative factors of cognitive dysfunction in PD patients: A cross-sectional study from Southwest China. *Neurological Research*, *38*(5), 434–440. https://doi.org/10.1080/01616412.2016.1139320
