# Supplementary material for: Cognitive function in different motor subtypes of Parkinson’s disease: A systematic review and multilevel meta-analysis
Source: Cogn Affect Behav Neurosci. 2025 Dec 17;26(1):218–66. doi: 10.3758/s13415-025-01343-8 (PMC12847103; doi:10.3758/s13415-025-01343-8)
Supplement: Supplementary file 3 — Supplementary file3 (PDF 197 KB) [file 13415_2025_1343_MOESM3_ESM.pdf]

Item	Instructions to Reviewers	Response Choices (left blank for free text items)
1. Study ID	Record study ID in the format [first author surname, year of publication]. Where there are two papers with the same first author and year of publication, use letters (a, b, etc.) placed immediately after the year of publication to distinguish the papers from each others, as per APA referencing style. The assignment of letters should be based on the alphabetical ordering of the references for the papers, based on the title of the paper.	
2. Study IDs of related studies	Record the study IDs (in the format [first author surname, year of publication]) for any related studies. These might be studies that report on the same dataset, or studies reporting on follow-up data for the same sample.	
3. Reviewer	Choose initials of person completing the form (e.g., BC for Brittany Child)	BC / IS / RD / BE / IB
4. Date form completed	Record date form completed in the format dd/mm/yy	
5. Publication year	Record publication year of article in the format yyyy.	
6. Country	Record country where the research was conducted. If multiple countries, list all countries separated by a semi-colon.	
7. Author contact details	Record contact details for corresponding author, as reported in article.	
8. Possible conflicts of interest	List any possible conflicts of interest, as reported in article.	
9. Study funding source	Select study funding source(s), as reported in article.	Government / University / Industry / Philanthropic / None / Not reported or unclear / Other (specify)
	**Study Design and Recruitment**	
10. Study design	Indicate if study was cross-sectional or longitudinal.	Cross-sectional / Longitudinal
11. Open access dataset	If the study has used an open access dataset, record the name of this dataset. For example, PPMI, which refers to the Parkinson's Progression Markers Initiative open access dataset. If not applicable, write 'NA'.	
12. Recruitment source	Record where participants were sourced from - for example, from the community or from a clinic (either as outpatients or inpatients).	Outpatients / Inpatients / Community / Other (specify)
13. Recruitment method	Describe any other details pertaining to the study's recruitment method. For example, how contact was made with participants (e.g., posters, GP referral, etc.).	
14. Sampling method	Specify the sampling method used to recruit participants.	Convenience / Consecutive / Random / Not reported or unclear / Other (specify)
15. Study setting	Record whether participants were recruited from a single location/centre or if they were recruited across multiple locations/centres.	Single centre / Multi-centre / Not reported or unclear
	**Sample Characteristics**	
16. Diagnosis method	Record the diagnostic method used for inclusion in the study, if reported. For example, diagnosis by a neurologist or post-mortem diagnosis. If not reported, write 'Not reported'.	
17. Diagnostic criteria	Select the diagnostic criteria used.	UK Brain Bank Criteria (Gibb and Lees, 1988) / Gelb et al. (1999) / Calne et al. (1992) / Larsen et al. (1994) / MDS-PD criteria (Postuma et al., 2015) / No formal criteria used / Not reported or unclear / Other (specify)
18. Sample size	Record the total sample size, N	
19. Inclusion and exclusion criteria	Record any inclusion and exclusion criteria used. For example, if sample was restricted to those who have received DBS surgery, those with an early age of disease onset, or de novo patients.	
20. Number of withdrawals/exclusions	Record the number of participants who withdrew or who were excluded from the study (separately, where possible). If none, write '0'.	

21. Reason(s) for withdrawals/exclusions	Where reported, record the reason(s) for participant attrition and exclusion. If not reported, write 'Not reported'. If not applicable (none withdrew or were excluded), write 'NA'.	
22. Differences between retained and lost participants	Record any data reported relating to comparisons between participants who were retained and lost (e.g., significant differences in disease duration, age, etc.). Identify whether these data indicate any systematic differences between the participants who were retained and those who were lost.	
23. Sample size after attrition/exclusion*	Record the sample size after participant attrition and exclusion. If unchanged, report sample size from Item 18.	
24. Comparison groups	Record any other comparison groups included in the study (e.g., healthy controls). Do not include these participants in the reporting of sample size, sociodemographic characteristics, or any other data extraction entry fields, as they are not of interest to the current review.	
25. Minimum age (years)	Record the minimum age of participants included in the final sample, if reported (this may be different to the age range specified in the inclusion criteria); otherwise, write 'NA'.	
26. Maximum age (years)	Record the maximum age of participants included in the final sample, if reported (this may be different to the age range specified in the inclusion criteria); otherwise, write 'NA'.	
27. Mean age (years)*	Record the mean age of the final sample, in years. If the mean age of the total final sample is not reported, but the mean age of each subtype is, calculate and record the mean age of the total final sample by weighting the mean age of each subtype by its sample size. If not reported, write 'NA'.	
28. SD age (years)*	Record the standard deviation (SD) of age for the final sample, in years. If SD age for the total final sample is not reported, but SD age of each subtype is, calculate and record SD age of the total final sample by weighting SD age of each subtype by its sample size. If standard error (SE) is reported but not standard deviation (SD), convert SE to SD using the equation given here: https://handbook-5-1.cochrane.org/chapter_7/7_7_3_2_obtaining_standard_deviations_from_standard_errors_and.htm If not reported, write 'NA'.	
29. Number of males*	Report the number of males included in the final sample. If a percentage or proportion is reported, calculate the raw number of males using the total sample size. If number of males in the total final sample is not reported, but number of males in each subtype is, calculate and record the number of males in the total final sample by summing across the subtypes. If not reported, write 'NA'.	
30. Number of females*	Report the number of females included in the final sample. If a percentage or proportion is reported, calculate the raw number of females using the total sample size. If number of females in the total final sample is not reported, but number of females in each subtype is, calculate and record the number of females in the total final sample by summing across the subtypes. If not reported, write 'NA'.	
31. Race/ethnicity	Record any summary data (e.g., counts or proportions/percentages) pertaining to the race/ethnicity of the sample, if reported; otherwise, write 'NA'.	
32. Other relevant sociodemographic characteristics	Record any summary data (e.g., means and SDs, counts or proportions/percentages) pertaining to any other relevant sociodemographic charactersitics reported by the study authors. For example, education status. If no other relevant sociodemographic characteristics reported, write 'NA'.	
	**Disease Characteristics**	
33. Minimum age at onset (years)	Record the minimum age at onset of participants included in the final sample, if reported (this may be different to the age at onset specified in the inclusion criteria); otherwise, write 'NA'.	
34. Maximum age at onset (years)	Record the maximum age at onset of participants included in the final sample, if reported (this may be different to the age at onset range specified in the inclusion criteria); otherwise, write 'NA'.	
35. Mean age at onset (years)*	Record the mean age at onset of the final sample, in years. If the mean age at onset of the total final sample is not reported, but the mean age at onset of each subtype is, calculate and record the mean age at onset of the total final	

	sample by weighting the mean age at onset of each subtype by its sample size. If not reported, write 'NA'.	
36. SD age at onset (years)*	Record the standard deviation (SD) of age at onset for the final sample, in years. If SD age at onset for the total final sample is not reported, but SD age at onset of each subtype is, calculate and record SD age at onset of the total final sample by weighting SD age at onset of each subtype by its sample size. If standard error (SE) is reported but not standard deviation (SD), convert SE to SD using the equation given here: https://handbook-5-1.cochrane.org/chapter_7/7_7_3_2_obtaining_standard_deviations_from_standard_errors_and.htm If not reported, write 'NA'.	
37. Minimum disease duration (years)	Record the minimum disease duration of participants included in the final sample, if reported (this may be different to the disease duration range specified in the inclusion criteria); otherwise, write 'NA'.	
38. Maximum disease duration (years)	Record the maximum disease duration of participants included in the final sample, if reported (this may be different to the disease duration range specified in the inclusion criteria); otherwise, write 'NA'.	
39. Mean disease duration (years)*	Record the mean disease duration of the final sample, in years. If the mean disease duration of the total final sample is not reported, but the mean disease duration of each subtype is, calculate and record the mean disease duration of the total final sample by weighting the mean disease duration of each subtype by its sample size. If not reported, write 'NA'.	
40. SD disease duration (years)*	Record the standard deviation (SD) of disease duration for the final sample, in years. If SD disease duration for the total final sample is not reported, but SD disease duration for each subtype is, calculate and record SD disease duration for the total final sample by weighting SD disease duration of each subtype by its sample size. If standard error (SE) is reported but not standard deviation (SD), convert SE to SD using the equation given here: https://handbook-5-1.cochrane.org/chapter_7/7_7_3_2_obtaining_standard_deviations_from_standard_errors_and.htm If not reported, write 'NA'.	
41. Minimum Hoehn and Yahr stage	Record the minimum Hoehn and Yahr stage of participants included in the final sample, if reported (this may be different to the Hoehn and Yahr stage range specified in the inclusion criteria); otherwise, write 'NA'.	
42. Maximum Hoehn and Yahr stage	Record the maximum Hoehn and Yahr stage of participants included in the final sample, if reported (this may be different to the Hoehn and Yahr stage range specified in the inclusion criteria); otherwise, write 'NA'.	
43. Mean Hoehn and Yahr stage*	Record the mean Hoehn and Yahr stage of the final sample, in years. If the mean Hoehn and Yahr stage of the total final sample is not reported, but the mean Hoehn and Yahr stage of each subtype is, calculate and record the mean Hoehn and Yahr stage of the total final sample by weighting the mean Hoehn and Yahr stage of each subtype by its sample size. If not reported, write 'NA'.	
44. SD Hoehn and Yahr stage*	Record the standard deviation (SD) of Hoehn and Yahr stage for the final sample, in years. If SD Hoehn and Yahr stage for the total final sample is not reported, but SD Hoehn and Yahr stage for each subtype is, calculate and record SD Hoehn and Yahr stage of the total final sample by weighting SD Hoehn and Yahr stage of each subtype by its sample size. If standard error (SE) is reported but not standard deviation (SD), convert SE to SD using the equation given here: https://handbook-5-1.cochrane.org/chapter_7/7_7_3_2_obtaining_standard_deviations_from_standard_errors_and.htm If not reported, write 'NA'.	
45. Other relevant disease characteristics	Record any summary data (e.g., means and SDs, counts or proportions/percentages) pertaining to any other relevant disease characteristics reported by the study authors. If no other relevant disease characteristics reported, write 'NA'.	
	**Medication and Comorbidities**	
46. Medication status*	Identify the medication status of participants (e.g., ON or OFF medication) at time of assessment.	ON medication / OFF medication / ON and OFF medication / De novo (newly diagnosed, never medicated) / Not reported or unclear
47. Medication type and dosage	Record the medication type (e.g., dopaminergic drugs) and summary data regarding dosage (e.g., mean and SD, median, range) for participants, where reported. If not reported, write 'NA'.	

48. Other relevant information about medication/treatment	Record any other relevant information pertaining to participants' medication or treatment not recorded elsewhere. If no other relevant information about medication/treatment reported, write 'NA'.	
49. Comorbidities	List any comorbidities reported, including number of participants affected (as count or proportion/percentage). If authors declare no comoribidities, write 'No comorbidities'. If comorbidities not reported on, write 'Not reported'.	
	**Subtyping Method**	
50. Type of subtyping*	Specify the type of subtyping used as 'Hypothesis-driven', 'Data-driven', 'Both', 'Other' (specify).	Hypothesis-driven / Data-driven / Both / Other (specify)
51. Measures of interest used to define groups*	List the measure(s) used to assess the motor variable(s) that were used to define the subtype groups. For example, UPDRS tremor score, UPDRS-III total score.	
52. Number of measures used to define groups	Record the number of measures used to define the subtype groups. This should be the sum of all the variables listed in Item 51. For example, if subtyping was based on UPDRS-III total scores only, the number of measures used to define the subtypes is 1; if the subtyping was based on UPDRS-III total scores and a tapping test score, the number of measures used to define subtypes is 2. Note that multiple measures belonging to the same assessment (e.g., motor subscales of the UPDRS-III) should be treated as separate measures for this item.	
53. Motor features used to define groups*	List the motor features used to define groups. The features used should be determined based on the variables used to define groups, which should each fit into one of the following categories: general motor, tremor, bradykinesia/akinesia (slowness of movement), muscle rigidity, gait/postural instability, other.	General motor (e.g., UPDRS-III total score) / Tremor / Bradykinesia/akinesia (slowness of movement) / Muscle rigidity / Gait / Other (specify)
54. Number of motor features used to define groups	Record the number of motor features used to define groups. This can be calculated from the number of domains selected in the previous data entry field.	
55. Missing data for variables used to define groups	Record whether there were any missing data for any of the variables used to define the subtype groups. If there were missing data, specify the number of participants for whom there was missing data and identify the variables for which data are missing. If the reason(s) for missing data is known, provide reason(s).	
56. How were missing data handled?	If applicable, describe how any missing data were handled. For example, were participants with missing data excluded, or were missing data imputed? If not reported or unclear, write 'Not reported or unclear'. If not applicable, write 'NA'.	Demographic (e.g., gender, age) / Motor / Cognitive / Psychiatric / Autonomic / Other non-motor / Treatment / Disability / Quality of life / Non-clinical biomarkers / Time-defined measures (e.g., disease duration, age at onset) / Other (specify)
57. If data-driven, how was number of groups decided?*	If a data-driven approach was used, identify the method(s) used to decide on the number of subtype groups. For example, silhouette method, elbow method. If not applicable, write 'NA'.	
58. If data-driven, what data pre-processing took places before variables were used for subtyping?*	If a data-driven approach was used, describe any data pre-processing (e.g., data transformations like converting to z-scores) that took place before the variables were used for subtyping. If none, write 'None'. If not reported or unclear, write 'Not reported or unclear'. If not applicable, write 'NA'.	
59. If data-driven, identify statistical method(s) used to derive groups*	If a data-driven approach was used, describe the statistical method(s) used to derive the subtypes. For example, k-means clustering, hierarchical clustering. If not applicable, write 'NA'.	
60. If hypothesis-driven, describe method used to subtype*	If a hypothesis-driven approach was used, describe the method used to produce the subtypes. If not applicable, write 'NA'.	
	**Results**	
61. Number of groups defined*	Record the number of groups defined by the subtyping approach (e.g., 2, 3, 4).	
62. Sample size of each group*	Record the sample size of each subtyping group.	
	Results on variables used to define subtypes	

63. Class of motor measure(s) used to define subtypes*	Record whether the motor measure(s) used to define subtypes were single-domain (domain-specific) or multi-domain (global), or a combination of both. The UPDRS-III, treated as an overall score, would be considered a multi-domain measure. Subscale scores of the UPDRS-III treated separately (e.g., tremor score, PIGD score) would be considered single-domain.	Single domain / Multi-domain / Combination of both
64. Group means for motor measure(s) used to define subtypes*	Record each group's mean on all motor measures used to define groups (e.g., UPDRS-III score, PIGD score). If not reported, write 'Not reported'.	
65. Group SDs for motor measure(s) used to define subtypes*	Record each group's standard deviation (SD) on all motor measures used to define groups (e.g., UPDRS-III total score, PIGD score). If standard error (SE) is reported but not standard deviation (SD), convert SE to SD using the equation given here: https://handbook-5-1.cochrane.org/chapter_7/7_7_3_2_obtaining_standard_deviations_from_standard_errors_and.htm If not reported, write 'Not reported'.	
66. Effect size(s) for motor measure(s) used to define subtypes*	Record effect size(s) between groups on all motor measures used to define groups (e.g., UPDRS-III total score, PIGD score). If effect size(s) have not been reported but p-value(s) have, record p-value(s) instead. If neither reported, write 'Not reported'.	
67. Type of effect size*	Specify the type of effect size recorded in the previous field. If previous field was not applicable, select 'NA'.	Cohen's d / Hedges' g / Eta-squared / Partial eta-squared / R-squared / Adjusted R-squared / No effect size(s) reported, but p-value(s) reported / Neither effect size(s) nor p-value(s) reported / NA / Other (specify)
68. Qualitative description of groups on each variable used to define subtypes	Provide a brief qualitative description of the groups, based on the variables used to define the groups. For example, 'Group 1 is characterised by strong tremor and good postural stability'.	
	<u>Results on variables used to compare groups post hoc</u>	
69. Measures of interest used to compare groups post hoc*	List the measure(s) used to assess cognitive variable(s) that were used to compare the subtype groups. For example, MMSE, digit span score.	
70. Other measures used to compare groups post hoc	List the measure(s) used to assess any other variable(s) on which the subtypes a were compared post hoc. These are any measures used to assess any non-cognitive variables - e.g., age, disease duration, RSBDQ to assess REM sleep behaviour disorder. If none, write 'None'.	
71. Domains used to compare groups post hoc	List the domains on which the subtype groups were compared post hoc. The domains used should be determined based on the variables used to compare groups post hoc, which should each fit into one of the following pre-defined domain categories (adapted from Mestre et al., 2021): demographic, motor, cognitive, psychiatric, autonomic, other non-motor, treatment, disability, quality of life, genetics, non-clinical biomarkers, time-defined measures (e.g., disease duration), other.	Demographic (e.g., age) / Motor / Cognitive / Psychiatric / Autonomic / Other non-motor / Treatment / Disability / Quality of life / Genetics / Non-clinical biomarkers / Time-defined measures (e.g., disease duration, age at onset) / Other (specify)
72. Number of domains used to compare groups post hoc	Record the number of domains used to compare groups post hoc. This can be calculated from the number of domains selected in the previous data entry field.	
73. Missing data for variables used to compare groups post hoc	Record whether there were any missing data for any cognitive measure(s) used to compare subtype groups post hoc. If there were missing data, specify the number of participants for whom there was missing data and identify the variables for which data are missing. If the reason(s) for missing data is known, provide reason(s).	
74. How were missing data handled?	If applicable, describe how any missing data were handled. For example, were participants with missing data excluded, or were missing data imputed? If not reported or unclear, write 'Not reported or unclear'. If not applicable, write 'NA'.	
75. Mean age for subtypes*	Record each group's mean age (in years). If not reported, write 'Not reported'.	

76. SD age for subtypes*	Record each group's standard deviation (SD) for age (in years). If standard error (SE) is reported but not standard deviation (SD), convert SE to SD using the equation given here: https://handbook-5-1.cochrane.org/chapter_7/7_7_3_2_obtaining_standard_deviations_from_standard_errors_and.htm If not reported, write 'Not reported'.	
77. Age differences between subtypes	Record if there were any significant differences in mean age between subtypes. Record effect size(s) (and specify type of effect size(s)). If effect size(s) have not been reported but p-value(s) have, record p-value(s) instead. If no significant differences, write 'No significant differences'. If neither reported, write 'Not reported'.	
78. Number of males*	Report the number of males included in each subtype group. If a percentage or proportion is reported, calculate the raw number of males using the total sample size of each subtype group.	
79. Number of females*	Report the number of females included in each subtype group. If a percentage or proportion is reported, calculate the raw number of females using the total sample size of each subtype group.	
80. Gender differences between subtypes	Record if there were any significant differences in gender composition between subtypes. Record effect size(s) (and specify type of effect size(s)). If effect size(s) have not been reported but p-value(s) have, record p-value(s) instead. If no significant differences, write 'No significant differences'. If neither reported, write 'Not reported'.	
81. Mean disease duration for subtypes*	Record each group's mean disease duration (in years). If not reported, write 'Not reported'.	
82. SD disease duration for subtypes*	Record each group's standard deviation (SD) for disease duration (in years). If standard error (SE) is reported but not standard deviation (SD), convert SE to SD using the equation given here: https://handbook-5-1.cochrane.org/chapter_7/7_7_3_2_obtaining_standard_deviations_from_standard_errors_and.htm If not reported, write 'Not reported'.	
83. Disease duration differences between subtypes	Record if there were any significant differences in mean disease duration between subtypes. Record effect size(s) (and specify type of effect size(s)). If effect size(s) have not been reported but p-value(s) have, record p-value(s) instead. If no significant differences, write 'No significant differences'. If neither reported, write 'Not reported'.	
84. Mean age at onset for subtypes*	Record each group's mean age at onset (in years). If not reported, write 'Not reported'.	
85. SD age at onset for subtypes*	Record each group's standard deviation (SD) for age at onset (in years). If standard error (SE) is reported but not standard deviation (SD), convert SE to SD using the equation given here: https://handbook-5-1.cochrane.org/chapter_7/7_7_3_2_obtaining_standard_deviations_from_standard_errors_and.htm If not reported, write 'Not reported'.	
86. Age at onset differences between subtypes	Record if there were any significant differences in mean age at onset between subtypes. Record effect size(s) (and specify type of effect size(s)). If effect size(s) have not been reported but p-value(s) have, record p-value(s) instead. If no significant differences, write 'No significant differences'. If neither reported, write 'Not reported'.	
87. Class of cognitive measure(s) used to compare subtypes post hoc*	Record whether the cognitive measure(s) used to compare the subtypes post hoc were single-domain (domain-specific) or multi-domain (global), or a combination of both. Digit span would be an example of a single-domain cognitive measure, as it assesses the domain of working memory. The MMSE would be an example of a multi-domain cognitive measure.	Single domain / Multi-domain / Combination of both
88. Specific cognitive domain(s) used to compare subtypes post hoc	Specify the cognitive domain(s) used to compare subtypes post hoc. Examples of tasks belonging to each domain are as follows: General/global cognition - MoCA, MMSE; Language - verbal fluency; Reasoning ability - Tower of London, Raven's Matrices; Visuospatial - mental rotation task; Visual processing speed - symbol digit, TMT-A, inspection time; Working memory - digit span, n-back; Response inhibition - Stroop task, stop-signal task, SART; Learning/memory/decision-making - Iowa Gambling Task, verbal learning; Attention - TMT-B	General/global cognition / Language / Reasoning ability / Visuospatial / Visual processing speed / Working memory / Response inhibition / Learning/memory/decision-making / Attention / Other (specify)
89. Group means for cognitive measure(s) used to compare	Record each group's mean on all cognitive measures used to compare groups post hoc (e.g., MMSE, MoCA). If not reported, write 'Not reported'.	

subtypes post hoc*		
90. Group SDs for cognitive measure(s) used to compare subtypes post hoc*	Record each group's standard deviation (SD) on all cognitive measures used to compare groups post hoc (e.g., MMSE, MoCA). If standard error (SE) is reported but not standard deviation (SD), convert SE to SD using the equation given here: https://handbook-5-1.cochrane.org/chapter_7/7_7_3_2_obtaining_standard_deviations_from_standard_errors_and.htm If not reported, write 'Not reported'.	
91. Effect size(s) for cognitive measure(s) used to compare subtypes post hoc*	Record effect size(s) between groups on all cognitive measures used to compare groups post hoc (e.g., MMSE, MoCA). If effect size(s) have not been reported but p-value(s) have, record p-value(s) instead. If neither reported, write 'Not reported'.	
92. Type of effect size*	Specify the type of effect size recorded in the previous field. If previous field was not applicable, select 'NA'.	Cohen's d / Hedges' g / Eta-squared / Partial eta-squared / R-squared / Adjusted R-squared / No effect size(s) reported, but p-value(s) reported / Neither effect size(s) nor p-value(s) reported / NA / Other (specify)
93. Class of motor measure(s) used to compare subtypes post hoc*	Record whether the motor measure(s) used to compare subtypes post hoc were single-domain (domain-specific) or multi-domain (global), or a combination of both. If no motor measure(s) were used to compare the subtypes post hoc, choose 'NA'. The UPDRS-III, treated as an overall score, would be considered a multi-domain measure. Subscale scores of the UPDRS-III treated separately (e.g., tremor score, PIGD score) would be considered single-domain.	Single domain / Multi-domain / Combination of both / NA
94. Group means for motor measure(s) used to compare subtypes post hoc*	Record each group's mean on any motor measures used to compare groups post hoc (e.g., UPDRS-III, PIGD score). If not reported, write 'Not reported'. If no motor measures used to compare subtypes post hoc, write 'NA'.	
95. Group SDs for motor measure(s) used to compare subtypes post hoc*	Record each group's standard deviation (SD) on any motor measures used to compare groups post hoc (e.g., UPDRS-III, PIGD score). If standard error (SE) is reported but not standard deviation (SD), convert SE to SD using the equation given here: https://handbook-5-1.cochrane.org/chapter_7/7_7_3_2_obtaining_standard_deviations_from_standard_errors_and.htm If not reported, write 'Not reported'. If no motor measures used to compare subtypes post hoc, write 'NA'.	
96. Effect size(s) for motor measure(s) used to compare subtypes post hoc*	Record effect size(s) between groups on any motor measures used to compare groups post hoc (e.g., UPDRS-III, PIGD score). If effect size(s) have not been reported but p-value(s) have, record p-value(s) instead. If neither reported, write 'Not reported'. If no motor measures used to compare subtypes post hoc, write 'NA'.	
97. Type of effect size*	Specify the type of effect size recorded in the previous field. If previous field was not applicable, select 'NA'.	Cohen's d / Hedges' g / Eta-squared / Partial eta-squared / R-squared / Adjusted R-squared / No effect size(s) reported, but p-value(s) reported / Neither effect size(s) nor p-value(s) reported / NA / Other (specify)
98. Qualitative description of groups on cognitive (and motor) variable(s) used to compare subtypes post hoc	Provide a brief qualitative description of the groups, based on the cognitive variables used to compare groups post hoc. For example, 'Patients belonging to Group 1 were more likely to have dementia compared to patients in Groups 2 and 3'. If the subtypes were compared on any motor variables post hoc (i.e., on motor variables OTHER than those used to derive the subtypes), also add a qualitative description of these group differences here.	
	Selective reporting	
99. Is there evidence of selective reporting?*	Indicate whether there seems to be evidence of selective reporting of results.	Yes / No / Unsure
100. If yes or unsure, provide details.	If the response to the previous item was 'Yes' or 'Unsure', provide details about what data/analyses seem to have been selectively reported.	
	**Longitudinal Data**	
101. Number of follow-ups*	If the study used a longitudinal design, indicate how many follow-up(s) were completed (e.g., 1, 2, 3). If not applicable, write 'NA'.	

102. Duration of follow-up(s)*	If the study used a longitudinal design, indicate the timepoints at which the follow-up(s) were completed. If the length of time was variable, reported the mean length of follow-up and any other relevant descriptive statistics (e.g., SD, range) that have been reported by the study authors. If not applicable, write 'NA'.	
103. Number of participants retained at follow-up(s)*	Record how many participants, n, were retained at each follow-up point. If not applicable, write 'NA'.	
104. Reason(s) for participant attrition at follow-up(s)	List the reasons given for participant attrition at each follow-up point (and where possible, report the number of participants lost due to each reason separately). If no reason(s) given, write 'Not reported'. If not applicable, write 'NA'.	
105. Differences between retained and lost participants at follow-up	Record any data reported relating to comparisons between participants who were retained and lost (e.g., significant differences in disease duration, age, etc.). Identify whether these data indicate any systematic differences between the participants who were retained and those who were lost. If not reported, write 'Not reported'. If not applicable, write 'NA'.	
106. Domains measured at follow-up(s)	List the domains measured at follow-up(s). The domains used should be determined based on the variables measured at follow-up(s), which should each fit into one of the following pre-defined domain categories (adapted from Mestre et al., 2021): demographic, motor, cognitive, psychiatric, autonomic, other non-motor, treatment, disability, quality of life, genetics, non-clinical biomarkers, time-defined measures (e.g., disease duration), other. If not applicable, choose 'NA'.	Demographic (e.g., age) / Motor / Cognitive / Psychiatric / Autonomic / Other non-motor / Treatment / Disability / Quality of life / Genetics / Non-clinical biomarkers / Time-defined measures (e.g., disease duration, age at onset) / NA / Other (specify)
107. Number of domains measured at follow-up(s)	Record the number of domains measured at follow-up(s). This can be calculated from the number of domains recorded in the previous data entry field. If not applicable, write 'NA'.	
108. Cognitive and/or motor measure(s) used at follow-up(s)*	List the cognitive and/or motor measure(s) used at follow-up(s). For example, MMSE, UPDRS tremor score, digit span score. If not applicable, write 'NA'.	
109. Baseline data	Indicate whether the baseline data are reported for the first time in this study, or whether the baseline data were initially reported as part of an earlier study. Provide a 'Yes', 'No', or 'Unsure' response. If 'No', give the study ID - in the format [first author surname, year of publication] - for the original study (or studies). If not applicable, write 'NA'.	
110. Use of follow-up data	Describe how the follow-up data were used in the subtyping analyses. For instance, were follow-up data used to derive the subtypes, either alongside, or independent of, baseline data? Were follow-up data used to compare subtypes derived from baseline data? Were follow-up data used to reproduce subtypes or examine subtype stability over time? If not applicable, write 'NA'.	
111. Statistical analyses using follow-up data*	Describe the statistical analyses used with the follow-up data, if separate to the main analyses recorded in earlier data entry fields. For example, 'The authors re-ran their hierarchical clustering analysis, this time using UPDRS data gathered at 5-years follow-up instead of baseline data.' If not applicable, write 'NA'.	
112. Results of analyses using follow-up data*	Record the results of any analyses conducted using follow-up data, if separate to the main analyses recorded in earlier data entry fields. For example. 'At 5 years follow-up, only 10% of patients classified as belonging to the tremor-dominant subtype at baseline had developed PDD, compared to 88% of patients classified as belonging to the akinetic-rigid subtype at baseline.' If not applicable, write 'NA'.	
113. Selective reporting of longitudinal data*	Indicate whether there seems to be evidence of selective reporting of results of follow-ups.	Yes / No / Unsure
114. If yes or unsure, provide details.	If the response to the previous item was 'Yes' or 'Unsure', provide details about what data/analyses seem to have been selectively reported.	
	**Reporting Quality**	
115. Reporting of subtype procedure/algorithm*	Did the authors provide a detailed procedure/algorithm for classifying individuals into groups? For a 'Yes' response, the level of detail provided must be sufficient for a reader to be capable of using the subtyping procedure/algorithm to classify a novel sample of patients.	Yes / No / Unsure

116. Subtype procedure/algorithm*	If the response to the previous data entry field was 'Yes', specify the procedure or algorithm used to classify individuals into subtypes. For hypothesis-driven studies, this may be a procedure that makes use of scoring thresholds on some assessment tool (e.g., UPDRS); for data-driven studies, this will likely be an algorithm. If not reported, write 'Not reported'.	
117. Subtype validation*	Did the authors attempt to validate their subtyping? For example, have they attempted to externally validate their defined subtypes (e.g., using a separate sample) or internally validate their defined subtypes (e.g., using test set taken from original sample). This validation may take the form of classifying individuals (taken from a new sample or a test set) into the subtypes already defined and testing whether the same post hoc differences between groups emerge. For data-driven studies, this validation may also take the form of attempting to reproduce subtypes that are qualitatively and/or quantitatively similar to the subtypes derived in the original sample when applying the same statistical method. For hypothesis-driven studies, this validation may also take the form of replicating well-established differences between groups on variables known to co-vary with the derived subtypes (e.g., demonstrating that akinetic-rigid patients are, on average, older than tremor-dominant patients, a finding that has been consistently reported elsewhere).	Yes / No / Unsure
118. Subtype validation method and results*	If the response to the previous data entry field was 'Yes', provide details on the validation method(s) used and the results obtained. For example, did the authors conduct internal validation (cross-validation) or external validation (application of subtype procedure/algorithm to a novel sample)? If not applicable, write 'NA'.	
119. Subtype stability*	Did the authors evaluate subtype stability over time?	Yes / No / Unsure
120. Subtype stability methods and results*	If the response to the previous data entry field was 'Yes', provide details on the methods used to evaluate subtype stability and the results of these analyses. If not applicable, write 'NA'.	
	**Re-Analysis and Author Contact**	
121. Re-analysis required	Is re-analysis of data required for inclusion in review?	Yes / No / Unsure
122. Description of re-analysis required	If the response to the previous data entry field was 'Yes' or 'Unsure', provide details of the re-analyses that are (or may be) required (e.g., calculating effect sizes).	
123. Author contact required*	Do the study authors need to be contacted for further information or data?	Yes / No / Unsure
124. Additional information/data required from authors	If the response to the previous data entry field was 'Yes' or 'Unsure', specify the exact data that needs (or may need) to be requested from the corresponding author. For example, list the variable name(s) and the data required (e.g., raw data, means, SDs, percentages, etc.) for each variable.	
	**Other Comments**	
125. Other comments	Record any other comments pertaining to this study that might be of relevance to the current review, or any problems encountered whilst extracting data from this study.	
	**Additional Items**	
126. Mean and standard deviation years of education for subtypes*	Record each group's mean and standard deviation (SD) for years of education. If standard error (SE) is reported but not standard deviation (SD), convert SE to SD using the equation given here: https://handbook-5-1.cochrane.org/chapter_7/7_7_3_2_obtaining_standard_deviations_from_standard_errors_and.htm If not reported, write 'Not reported'.	
127. Mean and standard deviation levodopa equivalent daily dose (LEDD) for subtypes*	Record each group's mean and standard deviation (SD) for levodopa equivalent daily dose (LEDD). If standard error (SE) is reported but not standard deviation (SD), convert SE to SD using the equation given here: https://handbook-5-1.cochrane.org/chapter_7/7_7_3_2_obtaining_standard_deviations_from_standard_errors_and.htm If not reported, write 'Not reported'.	
