# Supplementary material for: Cognitive function in different motor subtypes of Parkinson’s disease: A systematic review and multilevel meta-analysis
Source: Cogn Affect Behav Neurosci. 2025 Dec 17;26(1):218–66. doi: 10.3758/s13415-025-01343-8 (PMC12847103; doi:10.3758/s13415-025-01343-8)
Supplement: Supplementary file 4 — Supplementary file4 (PDF 44 KB) [file 13415_2025_1343_MOESM4_ESM.pdf]

Domain	Item	Scoring Range	Instructions to Reviewer
Domain 1: Recruitment and sample characteristics	1.1 Recruitment method	0-2	2 points are awarded if the sample is community- or population-based and/or the sample was recruited across multiple sites/locations. 1 point is awarded if the sample is clinic-based and sourced from a single site/location. To receive either 1 or 2 points, the authors must also clearly describe how participants were recruited and how long recruitment took (dates and length of time).
	1.2 Inclusion and exclusion criteria	0-1	1 point is awarded if inclusion and/or exclusion criteria are described (i.e., whether participants undergoing deep brain stimulation [DBS] therapy were eligible, whether there was a specific disease duration requirement, etc.).
	1.3 Sample characteristics	0-1	1 point is given if basic sample demographics are described, consisting of at least age, gender, and disease duration or age of onset.
	1.4 Participant medication	0-1	1 point is awarded if medication use and type are recorded alongside the dosage for participants. 0 points are awarded for only mentioning that participants are taking medication (without specifying type and/or dosage) or for not reporting medication use.
	Domain 1 quality	0-5	Sum of scores on all items belonging to Domain 1: Recruitment and sample characteristics (Items 1.1-1.4). Scored out of 5.
	Domain 1 risk of bias	Low / Moderate / High	Rate as low when domain quality score ≥4; rate as moderate when domain quality score = 3; rate as high when domain quality score ≤2.
Domain 2: Motor function measurement	2.1 Definition of motor feature(s) being measured	0-1	1 point is awarded if the specific motor assessment being used is described in detail; if a general assessment of overall motor function is used, then a general description of the assessment tool is sufficient. Note: Where a single study uses multiple motor assessments, the assessment with the highest rating on Item 2.2 (Valid and reliable measurement of motor feature(s)) should be used to assess all other Domain 2 items. In your supporting text, please specify which motor assessment tool you based your Domain 2 ratings on.
	2.2 Valid and reliable meaurement of motor feature(s)	0-3	3 points are awarded if a study uses at least one objective, specific assessment of each motor feature of interest (e.g., tapping test for bradykinesia, gait analysis for gait). 2 points are awarded if a study uses at least one clinical assessment for each motor feature of interest (e.g., tremor item score on Unified Parkinson's Disease Rating Scale [UPDRS]-III). 1 point is awarded if an appropriate general/global assessment of overall motor function is used (e.g., total UPDRS-III score). 0 points are awarded if the measure is not suitable to assess the construct of interest. Note: Where a single study uses multiple motor assessments, the assessment with the highest rating on Item 2.2 (Valid and reliable measurement of motor feature(s)) should be used to assess all other Domain 2 items. In your supporting text, please specify which motor assessment tool you based your Domain 2 ratings on.
	2.3 Continuous measurement of motor feature(s)	0-1	1 point is awarded if the motor function(s) are measured on a continuous scale (including any combination of UPDRS-III items) as opposed to a categorical measure (regardless of whether the authors analysed it as a continuous measure in the results section). Note: Where a single study uses multiple motor assessments, the assessment with the highest rating on Item 2.2 (Valid and reliable measurement of motor feature(s)) should be used to assess all other Domain 2 items. In your supporting text, please specify which motor assessment tool you based your Domain 2 ratings on.
	2.4 Method and setting of motor measure(s)	0-1	1 point is awarded if the authors administered their motor assessment(s) under the same assessment conditions (e.g., quiet room, under timed conditions) for all participants. Note: Where a single study uses multiple motor assessments, the assessment with the highest rating on Item 2.2 (Valid and reliable measurement of motor feature(s)) should be used to assess all other Domain 2 items. In your supporting text, please specify which motor assessment tool you based your Domain 2 ratings on.
	2.5 Proportion of data on motor measure(s) available for analysis	0-1	1 point is awarded when at least 80% of the sample has complete motor function data. Note: Where a single study uses multiple motor assessments, the assessment with the highest rating on Item 2.2 (Valid and reliable measurement of motor feature(s)) should be used to assess all other Domain 2 items. In your supporting text, please specify which motor assessment tool you based your Domain 2 ratings on.
	Domain 2 quality	0-7	Sum of scores on all items belonging to Domain 2: Motor function measurement (Items 2.1-2.5). Scored out of 7.
	Domain 2 risk of bias	Low / Moderate / High	Rate as low when domain quality score ≥6; rate as moderate when domain quality score >3 and <6; rate as high when domain quality score ≤3.
Domain 3: Cognitive function measurement	3.1 Definition of cognitive function(s) being measured	0-1	1 point is awarded if the specific cognitive assessment being used is described in detail; if a general assessment of overall cognitive function is used, then a general description of the assessment tool is sufficient. Note: Where a single study uses multiple cognitive assessments, the assessment with the highest rating on Item 3.2 (Valid and reliable measurement of cognitive function(s)) should be used to assess all other Domain 3 items. In your supporting text, please specify which cognitive assessment tool you based your Domain 3 ratings on.
	3.2 Valid and reliable	0-3	3 points are awarded if a study uses at least one objective, specific assessment of each cognitive feature of interest (e.g., digit span for working

	measurement of cognitive function(s)		memory, stop-signal task for response inhibition). 2 points are awarded if a study uses at least one recognised clinician-scored assessment for each cognitive feature of interest (e.g., interview-based assessments of cognitive function). 1 point is awarded if an appropriate global assessment of overall cognitive function is used (e.g., Mini-Mental State Examination [MMSE], Montreal Cognitive Assessment [MoCA]). 0 points are awarded if the measure is not suitable to assess the construct of interest. Note: Where a single study uses multiple cognitive assessments, the assessment with the highest rating on Item 3.2 (Valid and reliable measurement of cognitive function(s)) should be used to assess all other Domain 3 items. In your supporting text, please specify which cognitive assessment tool you based your Domain 3 ratings on.
	3.3 Continuous measurement of cognitive function(s)	0-1	1 point is awarded if the cognitive function(s) are measured on a continuous scale as opposed to a categorical measure (regardless of whether the authors analysed it as a continuous measure in the results section). Note: Where a single study uses multiple cognitive assessments, the assessment with the highest rating on Item 3.2 (Valid and reliable measurement of cognitive function(s)) should be used to assess all other Domain 3 items. In your supporting text, please specify which cognitive assessment tool you based your Domain 3 ratings on.
	3.4 Method and setting of cognitive measure(s)	0-1	1 point is awarded if the authors administered their cognitive assessment(s) under the same assessment conditions (e.g., quiet room, under timed conditions) for all participants. Note: Where a single study uses multiple cognitive assessments, the assessment with the highest rating on Item 3.2 (Valid and reliable measurement of cognitive function(s)) should be used to assess all other Domain 3 items. In your supporting text, please specify which cognitive assessment tool you based your Domain 3 ratings on.
	3.5 Proportion of data on cognitive measure(s) available for analysis	0-1	1 point is awarded when at least 80% of the sample has complete cognitive function data. Note: Where a single study uses multiple cognitive assessments, the assessment with the highest rating on Item 3.2 (Valid and reliable measurement of cognitive function(s)) should be used to assess all other Domain 3 items. In your supporting text, please specify which cognitive assessment tool you based your Domain 3 ratings on.
	Domain 3 quality	0-7	Sum of scores on all items belonging to Domain 3: Cognitive function measurement (Items 3.1-3.5). Scored out of 7.
	Domain 3 risk of bias	Low / Moderate / High	Rate as low when domain quality score ≥6; rate as moderate when domain quality score >3 and <6; rate as high when domain quality score ≤3.
Domain 4: Statistical analysis and reporting	4.1 Subtyping method	0-2	2 points are awarded if the method used to define subtypes is appropriate and described in sufficient detail. For both hypothesis-driven and data-driven studies, the variables used to derive the subtypes should be identified. For hypothesis-driven methods, the procedure used to classify individuals into the defined subtypes should be identified. For data-driven methods, at least one appropriate statistical method (e.g., 'elbow curve' method, silhouette analysis) must have been used to determine a suitable number of subtypes and the specific statistical analyses used to produce the subtypes should be identified and described (e.g., class of clustering method used), along with any data pre-processing undertaken. 1 point is awarded if the method used to define subtypes is appropriate and an attempt has been made to describe the subtyping process, but important detail (e.g., cut-off scores for hypothesis-driven subtyping, specific class of clustering method applied) is lacking. 0 points are awarded if the subtyping method used is not described or is inappropriate. Note: Where studies apply multiple subtyping methods to the same dataset, each subtyping method should be scored separately on all Domain 4 items. The average score will be taken to determine Domain 4 risk of bias. Please use the Covidence items to score a single subtyping method and then score any remaining subtyping methods in the supporting text for each Domain 4 item. Please make it clear which method you have scored using the Covidence items and which remaining method(s) you have scored in the supportive text.
	4.2 Motor characteristics of subtypes	0-2	2 points are awarded if a detailed quantitative description of the differences between subgroups is provided for all motor measures. This quantitative description must include reporting of relevant descriptive statistics (e.g., mean, standard deviation, percentages) and the reporting of statistical significance. 1 point is awarded if a quantitative description is provided, but lacks sufficient detail (e.g., statistical significance is not reported, selective reporting of only some motor measures). 0 points are awarded if no quantitative description is provided (e.g., only qualitative description given). Note: Where studies apply multiple subtyping methods to the same dataset, each subtyping method should be scored separately on all Domain 4 items. The average score will be taken to determine Domain 4 risk of bias. Please use the Covidence items to score a single subtyping method and then score any remaining subtyping methods in the supporting text for each Domain 4 item. Please make it clear which method you have scored using the Covidence items and which remaining method(s) you have scored in the supportive text.
	4.3 Cognitive characteristics of subtypes	0-2	2 points are awarded if a detailed quantitative description of the differences between subgroups is provided for all cognitive measures. This quantitative description must include reporting of relevant descriptive statistics (e.g., mean, standard deviation, percentages) and the reporting of statistical significance. 1 point is awarded if a quantitative description is provided, but lacks sufficient detail (e.g., statistical significance is not reported, selective reporting of only some cognitive measures). 0 points are awarded if no quantitative description is provided (e.g., only qualitative description given). Note: Where studies apply multiple subtyping methods to the same dataset, each subtyping method should be scored separately on all Domain 4 items. The average score will be taken to determine Domain 4 risk of bias. Please use the Covidence items to score a single subtyping method and then score any remaining subtyping methods in the supporting text for each Domain 4 item. Please make it clear which method you have scored using the Covidence items and which remaining method(s) you have scored in the supportive text.

	4.4 Effect sizes	0-2	2 points are awarded if effect sizes are clearly reported (in any format) for all motor and cognitive variables of interest. 1 point is awarded if sufficient data is provided for calculation of effect sizes without author contact. 0 points are awarded if author contact is necessary to obtain the data required for effect size calculation. Note: Where studies apply multiple subtyping methods to the same dataset, each subtyping method should be scored separately on all Domain 4 items. The average score will be taken to determine Domain 4 risk of bias. Please use the Covidence items to score a single subtyping method and then score any remaining subtyping methods in the supporting text for each Domain 4 item. Please make it clear which method you have scored using the Covidence items and which remaining method(s) you have scored in the supportive text.
	4.5 Appropriate accounting for effect of important confounders	0-1	1 point is awarded if an attempt has been made to statistically account for the effect of any other important sociodemographic and disease characteristics such as age, gender, and disease duration when reporting on the relationship between motor subtype and cognition. Note: Where studies apply multiple subtyping methods to the same dataset, each subtyping method should be scored separately on all Domain 4 items. The average score will be taken to determine Domain 4 risk of bias. Please use the Covidence items to score a single subtyping method and then score any remaining subtyping methods in the supporting text for each Domain 4 item. Please make it clear which method you have scored using the Covidence items and which remaining method(s) you have scored in the supportive text.
	4.6 Subtype reproducibility	0-1	For data-driven studies, 1 point is awarded if the algorithm used to classify individuals into the defined subtypes is reported, the authors have provided access to code, or the authors cite another paper that report the algorithm used. For hypothesis-driven studies, 1 point is awarded if the procedure used to classify individuals into the defined subtypes is reported or the authors cite another paper that details this procedure. For both types of studies, the amount of detail provided must be sufficient for it to be possible for new individuals to be classified into the defined subtypes based on the information provided without author contact required. Note: Where studies apply multiple subtyping methods to the same dataset, each subtyping method should be scored separately on all Domain 4 items. The average score will be taken to determine Domain 4 risk of bias. Please use the Covidence items to score a single subtyping method and then score any remaining subtyping methods in the supporting text for each Domain 4 item. Please make it clear which method you have scored using the Covidence items and which remaining method(s) you have scored in the supportive text.
	4.7 Subtype validation	0-2	2 points are awarded if the authors have attempted to externally validate their defined subtypes (e.g., using a separate sample). 1 point is awarded if the authors have attempted to internally validate their defined subtypes (e.g., using test set taken from original sample). This validation may take the form of classifying individuals (taken from a new sample [2 points] or a test set [1 point]) into the subtypes already defined and testing whether the same post hoc differences between groups emerge. For data-driven studies, this validation may also take the form of attempting to reproduce subtypes that are qualitatively and/or quantitatively similar to the subtypes derived in the original sample when applying the same statistical method. For hypothesis-driven studies, this validation may also take the form of replicating well-established differences between groups on variables known to co-vary with the derived subtypes (e.g., demonstrating that akinetic-rigid patients are, on average, older than tremor-dominant patients, a finding that has been consistently reported elsewhere). Note: Where studies apply multiple subtyping methods to the same dataset, each subtyping method should be scored separately on all Domain 4 items. The average score will be taken to determine Domain 4 risk of bias. Please use the Covidence items to score a single subtyping method and then score any remaining subtyping methods in the supporting text for each Domain 4 item. Please make it clear which method you have scored using the Covidence items and which remaining method(s) you have scored in the supportive text.
	4.8 Selective reporting	0-1	1 point is awarded if authors have not seemingly selectively removed data from their analyses or omitted select analyses or results when reporting study findings. Note: Where studies apply multiple subtyping methods to the same dataset, each subtyping method should be scored separately on all Domain 4 items. The average score will be taken to determine Domain 4 risk of bias. Please use the Covidence items to score a single subtyping method and then score any remaining subtyping methods in the supporting text for each Domain 4 item. Please make it clear which method you have scored using the Covidence items and which remaining method(s) you have scored in the supportive text.
	Domain 4 quality	0-13	Sum of scores on all items belonging to Domain 4: Statistical analysis and reporting (Items 4.1-4.8). Scored out of 13. Note: Where studies apply multiple subtyping methods to the same dataset, each subtyping method should be scored separately on all Domain 4 items. The average score will be taken to determine Domain 4 risk of bias. To do this, add up all the individual item scores for Domain 4 and then divide by the number of methods. For example, if two different subtyping methods were used, all eight Domain 4 items will have been scored twice, resulting in a total of 16 items. Sum the scores of all items and divide by two to get the Domain 4 overall quality score. Where necessary, round up or down to the nearest whole number.
	Domain 4 risk of bias	Low / Moderate / High	Rate as low when domain quality score ≥10; rate as moderate when domain quality score = >4 and <10; rate as high when domain quality score ≤4.
Domain 5: Follow-up data in longitudinal studies	5.1 Number of follow-up timepoints	0-1, NA	1 point is awarded if an attempt was made to assess participants at more than one follow-up timepoint. 0 points are awarded if the participants were only followed up at a single timepoint.

	5.2 Length of longest follow-up	0-1, NA	1 point is awarded if the amount of time between assessment at baseline and the longest follow-up timepoint is at least 12 months.
	5.3 Completeness of follow-up	0-2, NA	2 points are awarded if at least 75% of participants assessed at baseline are retained across all follow-up timepoints. 1 point is awarded if 50-74% of participants assessed at baseline are retained across all follow-up timepoints. 0 points are awarded if fewer than 50% of participants assessed at baseline are retained across all follow-up timepoints, or if the completeness of follow-up is not reported.
	5.4 Reasons for participant attrition	0-1, NA	1 point is awarded if the number of participants lost at follow-up is reported and the reasons for participant attrition are described. 0 points are awarded if the reasons for participant attrition are not reported.
	5.5 Comparison of participants retained and lost at follow-up	0-1, NA	1 point is awarded if the participants retained and lost at follow-up are compared on key characteristics (e.g., age, gender, disease duration). 0 points are awarded if the key characteristics of participants lost at follow-up are not reported.
	Domain 5 quality	0-6, NA	Sum of scores on all items belonging to Domain 5: Follow-up data in longitudinal studies (Items 5.1-5.5). Scored out of 6.
	Domain 5 risk of bias	Low / Moderate / High / NA	Rate as low when domain quality score ≥5; rate as moderate when domain quality score = >2 and <5; rate as high when domain quality score ≤2.
Overall	Overall risk of bias	Low / Moderate / High	Classify as low if reviewers deem all applicable domains as having low risk of bias, or up to one domain as having moderate risk of bias; classify as moderate if study does not meet the classification of low or high risk of bias; classify as high if reviewers deem one or more domains as having high risk of bias, or three or more domains as having moderate risk of bias.
